# The ALFA (Activity Log Files Aggregation) Toolkit: A Method for Precise Observation of the Consultation

**DOI:** 10.2196/jmir.1080

**Published:** 2008-09-08

**Authors:** Simon de Lusignan, Pushpa Kumarapeli, Tom Chan, Bernhard Pflug, Jeremy van Vlymen, Beryl Jones, George K Freeman

**Affiliations:** ^3^University for Health SciencesMedical Informatics and Technology (UMIT)Eduard Wallnofer Zentrum 16060 Hall in TirolAustria; ^2^Faculty of ComputingInformation Systems and MathematicsKingston UniversityKingston Upon ThamesSurreyUK; ^1^Biomedical InformaticsDivision of Community Health SciencesSt. George’s University of LondonLondonUK

**Keywords:** Video recordings, process assessment, observation, attitude to computer, professional-patient relations, general practice, family practice, decision modeling, process assessment, medical informatics, computers, medical records systems, computerized, electronic patient record (EPR), electronic medical record (EMR), evaluation methodologies, usability

## Abstract

**Background:**

There is a lack of tools to evaluate and compare Electronic patient record (EPR) systems to inform a rational choice or development agenda.

**Objective:**

To develop a tool kit to measure the impact of different EPR system features on the consultation.

**Methods:**

We first developed a specification to overcome the limitations of existing methods. We divided this into work packages: (1) developing a method to display multichannel video of the consultation; (2) code and measure activities, including computer use and verbal interactions; (3) automate the capture of nonverbal interactions; (4) aggregate multiple observations into a single navigable output; and (5) produce an output interpretable by software developers. We piloted this method by filming live consultations (n = 22) by 4 general practitioners (GPs) using different EPR systems. We compared the time taken and variations during coded data entry, prescribing, and blood pressure (BP) recording. We used nonparametric tests to make statistical comparisons. We contrasted methods of BP recording using Unified Modeling Language (UML) sequence diagrams.

**Results:**

We found that 4 channels of video were optimal. We identified an existing application for manual coding of video output. We developed in-house tools for capturing use of keyboard and mouse and to time stamp speech. The transcript is then typed within this time stamp. Although we managed to capture body language using pattern recognition software, we were unable to use this data quantitatively. We loaded these observational outputs into our aggregation tool, which allows simultaneous navigation and viewing of multiple files. This also creates a single exportable file in XML format, which we used to develop UML sequence diagrams. In our pilot, the GP using the EMIS LV (Egton Medical Information Systems Limited, Leeds, UK) system took the longest time to code data (mean 11.5 s, 95% CI 8.7-14.2). Nonparametric comparison of EMIS LV with the other systems showed a significant difference, with EMIS PCS (Egton Medical Information Systems Limited, Leeds, UK) (*P* = .007), iSoft Synergy (iSOFT, Banbury, UK) (*P* = .014), and INPS Vision (INPS, London, UK) (*P* = .006) facilitating faster coding. In contrast, prescribing was fastest with EMIS LV (mean 23.7 s, 95% CI 20.5-26.8), but nonparametric comparison showed no statistically significant difference. UML sequence diagrams showed that the simplest BP recording interface was not the easiest to use, as users spent longer navigating or looking up previous blood pressures separately. Complex interfaces with free-text boxes left clinicians unsure of what to add.

**Conclusions:**

The ALFA method allows the precise observation of the clinical consultation. It enables rigorous comparison of core elements of EPR systems. Pilot data suggests its capacity to demonstrate differences between systems. Its outputs could provide the evidence base for making more objective choices between systems.

## Introduction

### Electronic Patient Record (EPR) Systems Vary, and These Differences Provide Opportunities to Make Comparisons

Information and communications technology is ever more widely used in health care [1,2]; however, most EPR systems have grown organically, rather than being based on development specifications. Most countries have started with multiple small vendors developing EPR systems to meet the needs of the GP customers. Subsequently, commercial and regulatory pressures have reduced that number over time [3]; however, even within the same health system, the interfaces and functionalities clinicians use vary [4], as is the way they integrate the computer into the consultation [5]. Health systems are moving toward introducing new enterprise-wide information systems, which provide the opportunity for improved efficiency and patient safety through data sharing across the health system, so-called systemic interoperability [6]. The implementation of these new systems provides an opportunity to improve the interface and functionality, or, at the very least, have a rational reason for adopting the best design features of the existing systems.

### Using Video to Record the Impact of the EPR on the Clinical Consultation

For nearly a decade, we have been developing a video-based method to measure the influence of technology on the clinical consultation. We started with a single channel video, but found that, without simultaneously displaying the clinical system screen and closely questioning the clinician about their objectives behind interactions, it was impossible to interpret the video [7]. Trying to measure the precise length of interactions was also challenging.

We recognized that analogue video (which did not have an accurate time stamp), and using a stopwatch to time events in the consultation, had major limitations. Our next development was to record 3 channels of video: (1) wide-angle view of the consultation, (2) view of clinician’s head and upper body, and (3) screen capture. We used professional video recording tools to do this, as we needed an accurate time stamp to synchronize the videos. Although we produced useful output, the expense and the setup meant that this was not going to be a readily deployable technique [8].

Therefore, we set out to develop a recording method that would enable precise and objective measurement of consultation activities. The system would have to meet the following objectives: (1) can be readily set up in real consulting rooms or clinics in less than an hour; (2) be reliable and could be readily set up by others in a range of settings; (3) provide objective time stamps of activities within the consultation, allowing the synchronization and subsequent simultaneous viewing of multiple measures; and (4) produce an output that could be used by computer software engineers to develop better systems.

### Lack of Readily Available Applications to Compare EPR Applications

We initially reviewed existing applications that we could use to meet these specifications but found none. We looked at applications widely used for (1) qualitative research, (2) transcription and analysis of audio or video recordings, (3) usability testing, and (4) screen casting for demonstrations or training materials. Their shortcomings, compared with our requirements, are shown in Table 1.

There are well established applications used in qualitative research, such as ATLAS.ti (ATLAS.ti Scientific Software Development GmbH, Berlin, Germany) and QSR NVIVO (QSR International Pty Ltd, Melbourne, Australia) which allow detailed analysis and coding of text and multimedia data. They are not designed, however, to incorporate the precise monitoring of computer use that we require or to produce an output that can be exported into a package to develop UML diagrams. Transana (Wisconsin Center for Education Research, University of Wisconsin-Madison, Madison, WI, USA) provides facilities to perform a greater level of analysis by incorporating transcriptions; however its main analysis approach (which is based on the use of keyword, annotations, or their groupings) is not suitable to classify and measure doctor-computer interactions, which often include series of small durations or overlaps with patient interactions.

Widely used usability tools, such as Morae (TechSmith Corporation, Okemos, MI, USA), record observational data about computer use from multiple aspects. Due to the merged outputs they produce, they cannot be flexibly adopted according to research needs and are less helpful to obtain separate quantifiable measures for different combinations of interactions. Camtasia (TechSmith Corporation, Okemos, MI, USA), Adobe Captivate (Adobe Systems Incorporated, San Jose, CA, USA), and BB Flash Back (Blueberry Consultants Ltd, Birmingham, UK) are examples of screen-casting applications. While providing greater details about computer use, they are not optimized to classify interactions in a meaningful way. Focus of their outputs is too narrow to identify the effect of computer use on the overall consultation.

**Table 1:** Existing applications investigated

Expected features	Qualitative research		Transcription and analysis	Usability testing	Screen casting		
	ATLAS.ti	NVivo	Transana	Morae	Camtasia	Adobe Captivate	BB FlashBack
Handles input from 3 cameras or combined video	1 video file. Limited view	1 video file. Limited view	1 video file. Limited view	1 webcam	No	No	1 webcam
Computer screen capture	No	No	No	Yes	Yes	Yes	Yes
Fast setup for recording and data export	No recording element	No recording element	No recording element	Complex setup	Moderate setup, large data file	Moderate setup, large data file	Moderate setup, large data file
Coding and measuring of interactions	Codes segments. Manually measure.	Codes segments. Manually measure.	Codes video clip. No measure.	Codes video frame. Manually measure.	No coding. No measure.	No coding. No measure.	No coding. Manually measure.
Simultaneous viewing of multiple observations	Limited view. All in one channel	Limited view. Multiple channels	Limited view. All in one channel	Limited view. All in one channel	No	No	3 observations only
Easy to compare observational data	Using network diagrams	Using nodes or networks	Using codes, collections	Using tables, graphs	No	No	No
Standard output for UML diagrams	No	Need processing	No	Need processing	No	No	Only computer interactions

### Rationale for This Development

In the absence of any suitable off-the-shelf application, we commenced our own development process to produce a set of applications that would enable researchers to capture the complexity of the computer-mediated consultation.

## Methods

### Developing a Specification

We developed a specification for our development program based on our objectives and on our experiential learning about the limitations of existing techniques. We recognized that our technique should be extendible, to combine a number of monitoring methods which, at that time, we would not be able to define. At the time, we identified: (1) an indeterminate number of video channels; (2) a transcript of the consultation, captured with a precise time stamp, possibly using voice recognition software; (3) output from pattern recognition software [9] and other change recognition technologies [10]; (4) aggregate log files from observation techniques that we could not anticipate, as elements of our specification.

### Developing Separate Work Packages

We converted this work schedule into small work packages, which we developed separately on a largely opportunistic basis, as we had not received any consistent funding. The elements of this were:

1. To determine the optimal number of video channels and a low-cost way of recording. This should have time stamps to allow synchronization with other video channels and methods of data collection.

2. To find a reliable way to code the video footage, so we could navigate directly to particular activities in the consultation and measure their durations.

3. To automate the capture of body language and eye contact, using pattern recognition and gaze detection direction technologies.

4. To aggregate all these elements into a single navigable analysis output.

5. To introduce the ability to export data in a format that could readily be utilized by software engineers to improve systems.

### Multichannel Video

**Figure 1:**
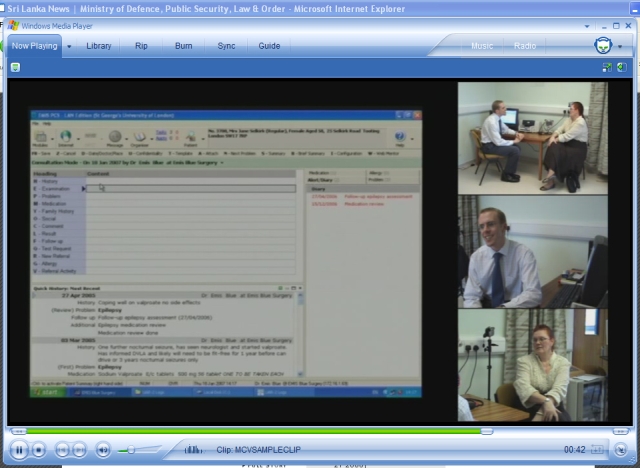
The multichannel video output, combined recordings of clinical computer system screen and 3 views of consultation

 We explored using 3, 4, and 5 channels of video, mixed onto a single screen, as well as a 4-channel version where clicking on a screen would enlarge that window to full screen (Figure 1). The additional channels experimented with since the 3-channel stage are the cameras focused on the patient’s upper body and the clinician’s facial view. We showed example consultations to experienced educationalists and academics accustomed to assessing video consultations, and we conducted semi-structured interviews to elicit their opinions [11].

 We also needed to identify low-cost methods of filming the consultation, ideally using unobtrusive tools, which recorded sound and video with a digital time signal so that precise synchronization was possible [12].

### Capturing and Coding Consultation Activity

**Figure 2:**
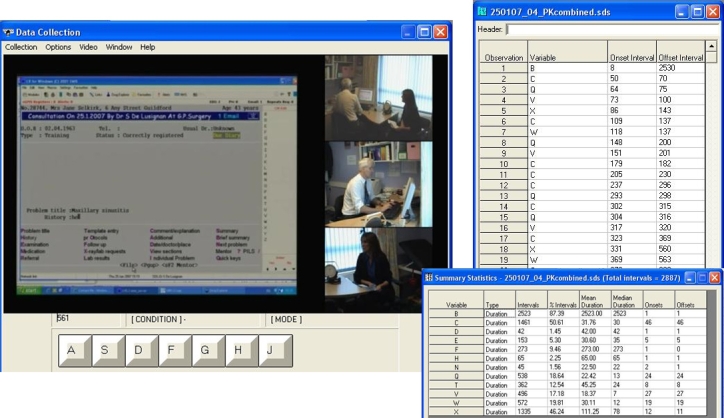
Observational data capture using ObsWin, rating interface and outputs with summary statistics

 We needed to be able to code interactions in the consultation so that we could readily navigate to a particular activity (eg, prescribing) and also identify its duration. We selected a flexible software called “ObsWin” (Antam Ltd, London, UK) to do this [13] (Figure 2). We conducted reliability tests of our manual coding method using multiple observers coding simulated blood pressure management follow-up consultations. We used intra-class correlation coefficient as an index of reliability [14]. Subsequently, we compared the manual coding time for prescribing activities with frame-by-frame analysis of the video to further assess the reliability of our approach.

**Figure 3:**
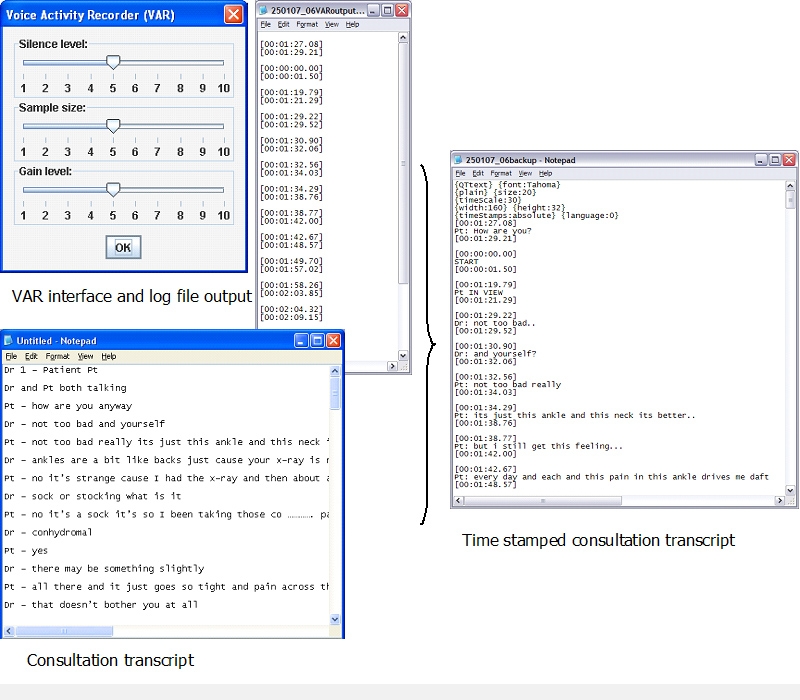
Time-stamped consultation transcript creation using VAR

Wherever possible, we set out to automate the time stamps for the start and end of activities in the consultation. We developed a User Action Recording (UAR) application to measure the precise time stamp of keyboard use (each key depression is recorded and time stamped), as well as all mouse clicks and coordinates. We also produced a Voice Activity Recorder (VAR), which detects and time stamps the start and end of speech (Figure 3).

### Automated Capture of Body Language

**Figure 4:**
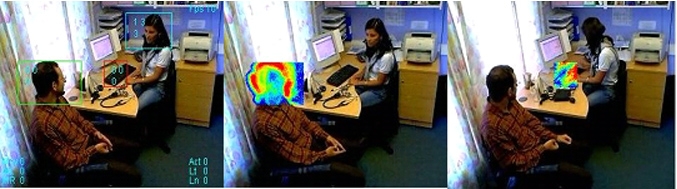
Measurement of nonverbal interactions using PRS, patient’s head nodding and doctor’s keyboard use

We automated the capture of body language to interpret nonverbal interactions and the direction of gaze to infer eye contact between clinician and patient. We experimented with Algol, an experimental pattern recognition software (PRS) not released as a commercial product  (Main Highway Services, Winchester, UK), exploring correlation between movements detected with the software and manually detected activity [15] (Figure 4). We explored the possibility of obtaining software that measured the direction of gaze.

### Aggregation and Navigation Application

**Figure 5:**
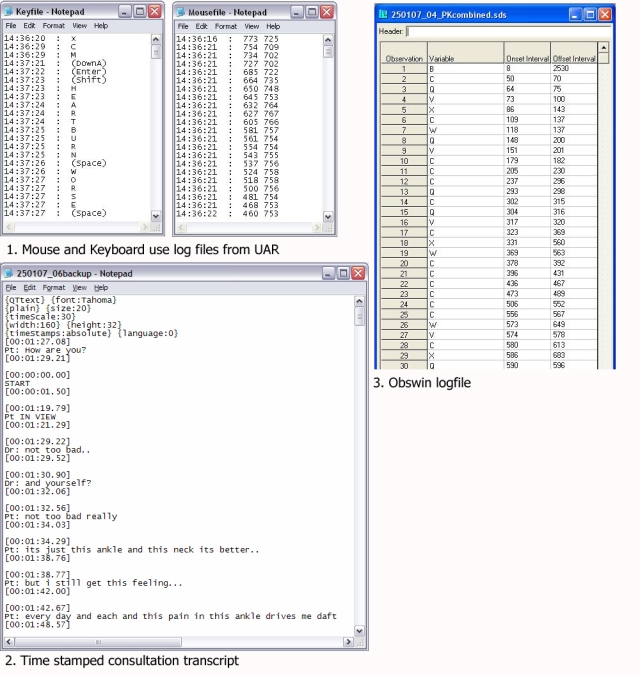
Time stamped log files created by three different consultation activity observation methods.

We needed to aggregate the output from multiple data collection systems (Figure 5) into a single application that would be readily navigable. It needed to be able to flexibly load any number of input files and produce outputs that could be readily utilized in other applications. Unsuccessful effort to identify an appropriate proprietary application resulted in the in-house development of the Log Files Aggregation (LFA) application [16].

### Output That Could Facilitate Better Clinical Computer System Development

We wanted to produce an output that would be readily interpretable by software engineers, so that our findings had a utility beyond the health care community. We specified our aggregation tool to export the combined log files in XML (extensible mark-up language) format, so they can be readily imported and interpreted by other applications. Process models of consultation tasks created using the UML, a standard modeling and specification notation widely used in software engineering, was chosen as our main mechanism for representing the use and impact of clinical system features within the consultation.

### Pilot Recording of Consultations

We developed our method using simulated consultations between clinicians and actor patients within a simulated clinical environment. We initially developed the technique using standard consultations (eg, follow-up blood pressure checks [14]) and then a wider range of clinical problems.

We needed to know whether our technique was practical to set up within a standard consulting room and could cope with background noise, variable lighting including window position, and room size. We next tested our technique using actor patients in GP surgery premises. We found that audio recording from 1 camera was satisfactory; modern cameras coped well with variations in lighting, and 2 people could set up the cameras and install the other data-capture methods in less than 20 minutes. We found that the cameras and other data-capture tools could capture more than an hour’s data, but that it was prudent to remove screen capture and video data in a pause between consultations after 45 minutes.

We next developed a protocol that included our technical method, obtaining proper consent from patients and securing the data. We wanted to obtain pilot data from the 4 different most used brands of GP EPR systems, so we could make comparisons. These 4 brands are: (1) EMIS LV, the longest established and, at the time of the study, the most used system; (2) EMIS PCS, a more modern version from the same manufacturer; (3) INPS Vision; and (4) iSoft Synergy. EMIS LV is largely character user interface (CHUI) driven, whereas the other 3 have graphical user interfaces (GUI).

In our pilot analysis, we only included coding carried out using the picking list or other routine coding tools. We did not include data entry forms or templates that could facilitate more rapid data entry. The 4 GPs we filmed had used their current computer system for at least 3 years and had not routinely consulted with paper records for at least this period.

### Statistical Methods

We planned to compare the time taken to carry out clinical coding, prescribing, and other routine tasks in the clinical consultation. We expected data from a small pilot to not have a normal distribution. This expectation is for 2 reasons: (1) we have a small sample and (2) we expected a skewed distribution because sometimes these tasks take a long time, but they always take a minimum time. We used box whisker plots to visually compare actions that were frequently recorded. We also used nonparametric tests (Mann-Whitney U test) to differentiate between EMIS LV (the then most used brand of GP EPR system) with the other systems. We next used the Krushkal-Wallis to explore any statistically significant difference in mean ranking. We used SPSS version 15 to carry out these analyses.

### Ethical Considerations

We obtained ethical approval for the pilot recording of live consultations via the National Health Services Central Office for Research Ethics Committees (COREC). The protocol included making proper provision for the secure transport and storage of media and limiting access.

We used a 3-step process to obtain consent from patients to be video recorded. First, the video sessions were marked as such in participating practices, so that patients who booked into these sessions knew they were going to have their consultation video taped by 3 cameras as part of a research project. Second, they signed consent at the start of the consultation and were told that, if they did not want the video used after the consultation, they were free to say so. Finally, they and the clinician signed consent after the consultation stating that they remained willing for the consultation data to be used in research.

## Results

### Technical and Pilot Investigation Results

The results initially report a summary of our final technical method and then the results of our pilot study. The full description of the technical process is contained in Appendix 1.

### Number of Video Channels Optimal for Analysis

We found 4 video channels to be optimal for observing the consultation. Our 3-channel video method, which provides an overview of the consultation, the clinician’s upper body, and screen capture, overcame most of the problems associated with single-channel observation [17]; however, a qualitative investigation suggested a fourth channel filming the head and upper torso of the patient was essential to capture the patient’s body language [11] (see Multimedia Appendix 3). In 2006, we found we could source the necessary hardware for 3-channel video around 1100 Euros [18] (or 1500 Euros for 4 channels).

### Coding Consultation Activity

We used our in-house–developed UAR to capture mouse and key movement and VAR to time stamp the start and end of speech. We have piloted the use of UAR to compare the time taken to code a new problem and to issue a single acute prescription on 2 different GP computer systems [19].

The use of VAR overcame the limitations of manual coding of the start and end of speech. Prior to using VAR, we found that training manual raters could reliably code simulated consultations [14], but when presented with a heterogeneous mixture of real consultations, some activities were less reliably coded. The VAR also enables us to identify who initiates and terminates silence. We have observed how the clinician sometimes makes purposeless use of the IT to initiate silence to control the consultation [20].

### Automated Capture of Body Language

We have extensively tested pattern recognition software to see if we can automate the capture of body language and movements such as affirmative head nodding; however, limitations in this technology, and our ability to process it, have left us unable to correlate this with the output from our manual observations.

### The Log File Aggregation (LFA) Tool for Synchronizing and Simultaneous Viewing of Log Files

**Figure 6:**
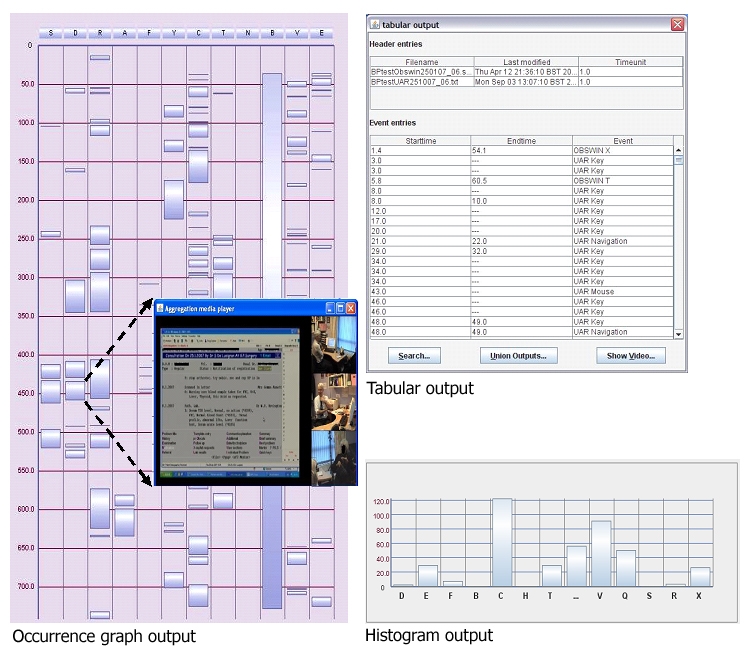
Analyzable outputs of the ALFA tool after aggregation

The LFA tool combines any number of time-stamped log files of different formats. The data imported into LFA can be viewed as histograms or occurrence graphs (Figure 6). The power of this tool in analysis is that clicking on a rectangle representing a specific variable takes the user directly to the appropriate spot in the multichannel video (see Multimedia Appendix 4). This enables users to navigate into any spot in the consultation they wish to study and simultaneously view all the log files relating to that point in time. Reader programs could successfully interpret XML output from the ALFA tool.

### Output That Can Be Used by Software Developers

**Figure 7:**
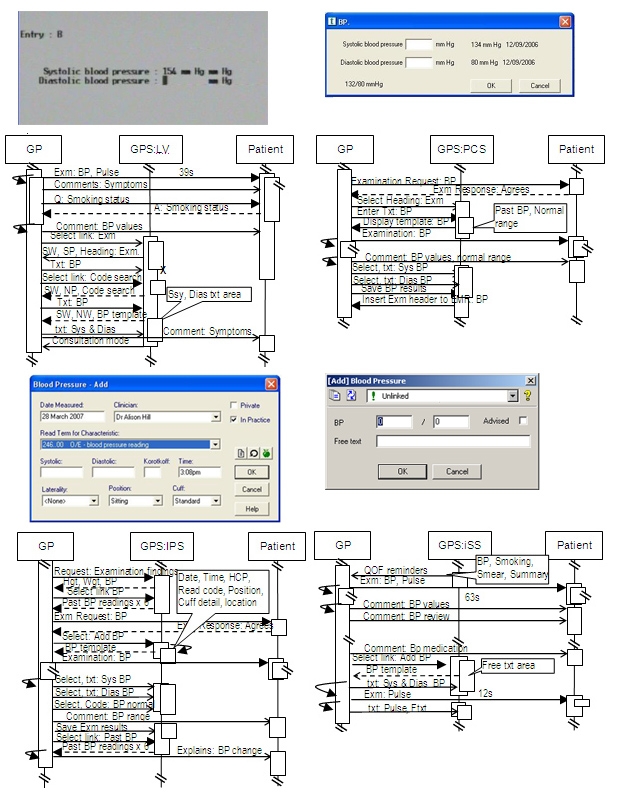
Blood pressure data recording interfaces of 4 different EPR systems and sequence diagrams for the interactions observed

UML sequence diagrams demonstrated the clinicians’ use of EPR system components within the consultation. They contrasted the variations of computer use and how this might be related to interface features. Software developers could examine these process models to evaluate the use and performance of design characteristics within a consultation. We have used the UML outputs to contrast the definition of the presenting problem, prescribing [21], past encounter reviewing, and BP data entry stages (Figure 7) [22]. Examples for design features that we could identify as having an impact on the consultation are: (1) navigation method (use of icons, function, or arrow keys), (2) structure of the main interface (single, sub, or tab-separated windows, (3) display of alerts or prompts, (4) mechanism for searching coded data, (5) retrieving of historical data, etc. The output from LFA automatically creates the framework of a UML sequence diagram. It takes approximately an hour to manually annotate the remaining sequences in a 10-minute consultation.

### Pilot Data

There is considerable heterogeneity of computer use between consultations. We collected initial data from 22 consultations from 4 practices. Each computer system was only used by 1 GP. The GPs generally coded between 1 and 3 items per consultation, though 12 items were coded in one iSoft consultation, and the GP using EMIS LV appeared to code more. The summary of the coding carried out in each consultation is shown in Table 2. Only 2 of the 22 patients seen in this pilot asked to have the cameras switched off. No patients or clinicians withdrew consent for video material to be used post consultation.

**Table 2:** Coding carried out in the pilot consultations

EPRsystem	Consultation ID	No. of items coded	Acute prescriptions (Rx) issued	Repeat prescriptions (Rx) issued	BP measured	Other coded/prescription related computer use
**EMIS LV**	EL1	2	1	0	0	
	EL2	1	0	1	0	
	EL3	3	1	0	1	
	EL4	1	3	0	0	
Total	4	7	5	1	1	
**EMIS PCS**	EP1	5	1	0	0	Prescription restarted
	EP2	1	1	0	0	
	EP3	2	1	0	0	
	EP4	4	2	0	0	
	EP5	7	2	0	0	
	EP6	1	0	0	0	
	EP7	4	0	0	1	
Total	7	24	7	0	1	
**INPS Vision**	IV1	3	2	2	1	
	IV2	1	1	1	1	
	IV3	3	1	0	1	Weight
	IV4	1	0	0	0	
	IV5	1	2	0	1	Weight, Rx cancelled
	IV6	3	2	1	0	
	IV7	2	1	0	1	Drug allergy, Rx cancelled
Total	7	14	9	4	5	
**iSoft Synergy**	IS1	1	0	0	2	BMI, Rx cancelled
	IS2	12	0	0	1	
	IS3	2	0	0	0	
	IS4	3	0	0	0	
Total	4	18	0	0	3	

**Figure 8:**
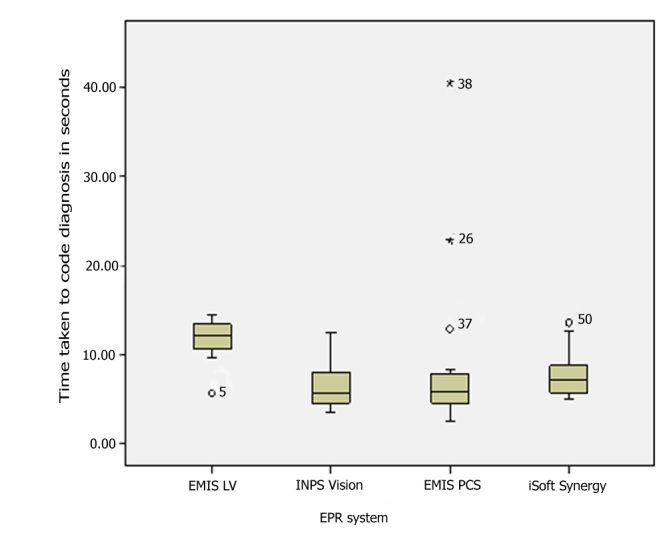
Box-whisker plot comparing coding times with different brands of GP EPR systems

**Figure 9:**
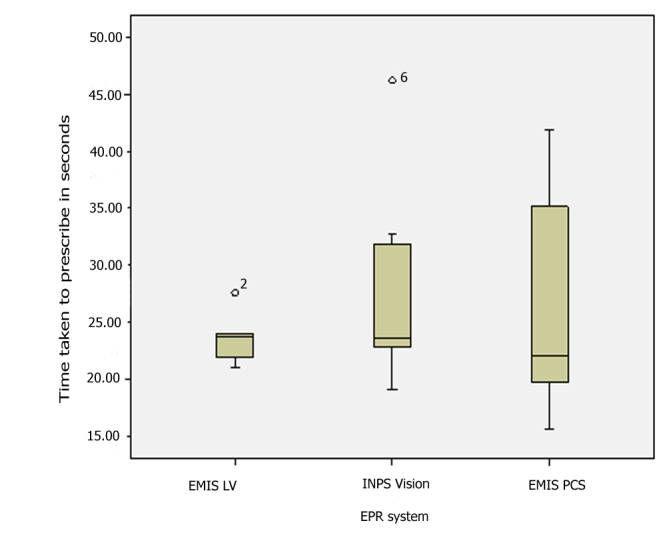
Box-whisker plot comparing prescribing times with different brands of GP EPR systems

We observed differences in time taken to code data, prescribe, and repeat prescribe into the computer systems, though we only had sufficient episodes of coding data and acute (new) prescribing to make any sort of statistical comparison. The descriptive findings are shown in Table 3, and the coding and repeat prescribing data are illustrated using box-whisker plots (Figure 8 and Figure 9.) The clinician using EMIS LV (the CHUI interface) appears to take longer to code items than users of other systems. Their mean ranking (Kruskal-Wallis test) was in the following order: EMIS LV, slowest (highest median); then iSoft Synergy was second slowest to code data; the fastest two were INPS Vision and EMIS PCS, having similar medians. The difference in medians was statistically significant (*P* = .007). Nonparametric (Mann-Whitney U) tests showed that they were all statistically significantly faster than EMIS LV; for EMIS PCS and INPS *P* < .01 and for iSoft Synergy *P* < .05 (Table 3).

Acute prescribing appears to be faster with EMIS LV; however, although the EMIS LV prescriber was consistently at the faster end of prescribing time, there is overlap with the other systems shown in the box-whisker plots. Not surprisingly, the difference in medians was not statistically significantly different from the other two systems for which we have acute prescribing data (*P* = .71 and *P* = .64).

**Table 3:** Comparison between EMIS LV (EL), EMIS PCS (EP), INPS Vision (IV), and iSoft Synergy (iS) of time taken to code data, prescribe, and record BP data

	Coded Data Entry				Acute Prescribing			Repeat Prescribing		BP Recording			
	EL	EP	IV	iS	EL	EP	IV	EL	IV	EL	EP	IV	iS
N	7	24	14	18	5	7	9	1	4	1	1	5	3
Mean (SD)	11.5 (3.0)	8.1 (8.0)	6.8 (2.9)	7.9 (2.5)	23.7 (2.5)	27.1 (10.1)	27.5 (8.5)	21 -	8.4 (3.2)	7.1 -	9 -	9.8 (3.4)	6.7 (1.3)
95% CI	8.7 - 14.2	4.7 - 11.5	5.1 - 8.5	6.6 - 9.2	20-5 - 26.8	17.7 - 36.5	20.9 - 34.0	-	3.3 - 13.5	-	-	5.6 - 13.9	3.5 - 9.8
Median (IQR)	12.1 (2.8)	5.9 (3.2)	5.7 (3.3)	7.2 (2.7)	23.8 (2.1)	22.1 (15.4)	23.6 (9)	21 -	9.4 (3.8)	7.1 -	9 -	8.8 (1)	7.3 (1.1)
MIN	5.7	2.5	3.6	5.1	21	15.7	19.1	21	4	7.1	9	6.7	5.2
MAX	14.4	40.5	12.5	13.6	27.6	41.9	46.2	21	10.7	7.1	9	15.5	7.5
NPAR^*^ P		0.007	0.006	0.012		0.71 (NS)	0.64 (NS)						

^*^NPAR = nonparametric test compared with EMIS LV; exact statistical significance is shown for the Mann-Whitney U test (2-tailed). NS = not significant.

## Discussion

### Principal Findings

The ALFA toolkit allows greater precision of observation of the clinical consultation than other techniques. The current toolkit allows multiple video channels including screen capture, the consultation transcript, computer use, and speech to be precisely synchronized, timed, and navigated through. There is enough scope to add other input as required. Its output can be used to create models that software engineers could use to develop better EPR systems.

The multichannel filming appears to be acceptable to patients; however, the 4 practices involved were teaching and training practices where both medical students and trainee doctors regularly video tape themselves.

Our pilot data shows how the method allows small, but statistically significant, differences between clinical systems and users to be measured. Although these differences in time per coded item and prescription are relatively short, when multiplied up through a clinician’s day, better interfaces might result in a considerable time saving.

The UML models of BP recording show how having the previous reading readily available positively influences the clinical process and provide insights into how the new computer interfaces might be developed in the future. This principal could be carried forward into the recording of other common clinical information, for example, recording a smoking habit or adverse reactions to medication.

### Implications

We developed this tool to meet our aspirations to evaluate the impact of technology on the consultation. Its precise time stamps could be used to compare clinical computer systems or to contrast the time taken with paper systems versus computerization. Comparative analysis of computer use and clinician-patient interactions could determine the common tasks and be used to develop theoretical models for computer-mediated consultation. We hope that our UML sequence diagrams will enable the clinical system designers to evaluate existing systems and also develop and evaluate new features.

The ALFA toolkit can also be used to measure the performance of the clinician or the reaction of the patient. Colleagues who have seen this technique have suggested that remedial doctors assessed in simulated surgeries could be given multichannel videos of their performance as a tool for reflection; if we could automate measures of body language, then this might be used as a formative assessment of communication skills.

The full set of tools created by the team and their source codes are freely available under a GNU General Public License (GNU GPL). Instructions for download, set up, sample files, and links to other related resources are made available as Web resources [23].

### Limitations of the Method

Some of the parts of our development failed. We were unable to use the log file from the motion recognition software effectively. As yet, we have not been able to achieve a transcript from a voice-recognition system; these technologies still require training and are unable to recognize differing patients’ voices. We have not been able to access suitable methods to measure direction of gaze; commonly available tool kits are intrusive.

We have run this development as a series of small-scale components, rather than as a comprehensive program.

Our pilot data only used one clinician per system. More data are needed to discover if these differences were clinician-related or system-related.

### Comparison With the Literature

We are unaware of any similar technique that provides such precision of observation (see Multimedia Appendix 2). Table 4 compares the features of ALFA against popular existing techniques. Although the study of human computer interaction (HCI) is a well developed discipline, it focuses on the interaction between 1 or more individuals and 1 or more computer systems [24]. In HCI, the user-computer interaction has primacy; we wanted instead to develop a toolkit to capture the complex social interaction of the consultation, within which the clinician-patient activity is pre-eminent. 

**Table 4:** Comparison of ALFA toolkit features with existing tools

ALFA element and comparable functionality	Existing tools								ALFA tool kit
	Qualitative research		Transcription and analysis	Usability testing	Screen casting				
	ATLAS.ti	NVivo	Transana	Morae	Camtasia	Adobe Captivate	BB F’Back		
**1. Multichannel Video (MCV) recording**									
Screen capture	N/A	N/A	N/A	Yes	No	No	Yes		Yes
Video capture	N/A	N/A	N/A	1 camera	No	No	1 camera		3 cameras
Audio capture	N/A	N/A	N/A	Yes	Yes	Yes	Yes		Yes
**2. Observational Data Capture (ODC)**									
Multimedia import	Yes	Yes	Yes	Yes	No	No	No		Yes
Sufficient video display	Yes	Yes	No	No	N/A	N/A	N/A		Flexible
Video controls	Yes	Yes	Yes	Limited	N/A	N/A	N/A		Yes
Exports durations directly	No	No	No	No	N/A	N/A	No		Yes
Method of coding for interactions	Codes, Memos	Codes, Memos, Nodes	Keywords, Comments	Markers	No	No	No		duration variables
Interaction durations	No	Graphical	No	Yes	No	No	Graphical		Direct export
**3. User Activity Recording (UAR)**									
Keyboard use	N/A	N/A	N/A	Yes	No	No		Yes	Direct export
Mouse use	N/A	N/A	N/A	Yes	Yes	Yes		Yes	Direct export
Interaction durations	No	No	No	No	No	No		No	No
Lightweight to install	N/A	N/A	N/A	No	No	No		No	Yes
**4. Voice Activity Recording (VAR) and transcription**									
Indicates voice levels	No	No	Yes	No	No	No		Yes	Yes
Measures verbal interactions	No	Manual	Manual	No	No	No		Manual	Direct export
Import/create transcriptions	Yes	Yes	Yes	No	No	No		No	Yes
Time-stamped transcriptions	No	Yes	Yes	No	No	No		No	Yes.
**5. Log File Aggregation**									
Combine data from different tools	video and transcript’s	video and transcripts	video and transcript’s	Screen capture and video	No	No		No	Up to 10. Can extend further
Single exportable file	Yes	No	No	No	Yes	Yes		Yes	Yes, many formats
XML output	Yes	No	No	No	No	No		No	Yes
**6. Occurrence graphs**									
Time lines for interaction	No, Network diagrams	Yes, small display	No, Clips organised with labels	1 timeline	No	1 timeline		mouse, keyboard and voice	Multiple timelines. Large display
Interactions mapped to video	Yes	Yes	Yes	Yes	No	No, to screen capture		No, to screen capture	Yes
Interaction durations linked to video	No	Yes	No	Yes	No	No, linked to frame		No, linked to frame	No
**7. UML process modeling**									
Use for UML validation	Limited	Limited	No	Limited	No	No		No	No
Indicates interactions and durations in channels	No	Yes, limited by display area	No	Yes, 1 at a time	No	No		Only for mouse, keyboard and voice	Yes, multiple channels of interactions
Shows interaction type directly	No, Using codes	Yes	No, Using labels	No, Using markers	No	No		Only mouse, keyboard and voice	Yes.

Evaluation methods in software engineering combine multiple techniques for observation [25, 26]. The analyzable products of these are often a data file stream combining visual or audio representations of sequence of activities in sensory channels [27]. We are not aware, however, of any application that enables such a range of log files to be aggregated, synchronized, and, where needed, exported into other applications. Some keyboard listening or spyware applications could identify the sequence of keyboard activities. Voice spectrum analyzer applications that can present visual data about sound levels also exist. Unlike UAR and VAR applications, these are not capable of timing the computer use or verbal interactions in an analyzable format.

This method examines the impact of the computer on the consultation from a broad sociotechnical perspective, as advocated by Coiera [28], rather than from a purely technical perspective. Rigorous and broadly acceptable evaluation frameworks of IT in health care should be capable of identifying problems, suitable tools for evaluation, and methods for applying them sensibly [29]. It potentially helps fill some of the gaps in the methods for evaluation of health care systems [30].

### Call for Further Research

More research is needed on how to automate data collection regarding the impact of technology on the consultation. Improved voice-recognition techniques would save the time spent in transcribing. As well as filling in the gaps about how to use pattern-recognition software and visual gaze estimation software to capture body language, we need to consider how we might embed logs into active clinical systems so that, for example, the change in length in consultation associated with a new release of software can be automatically measured and potentially investigated.

We also need to explore with a larger sample what are true differences between EPR systems and what is clinician variation. Recording several clinicians using 1 system should enable us to do this.

### Conclusions

We set out to develop tools that would provide objective time stamps of activities within the consultation, allowing their simultaneous viewing and analyzing interactions in detail. The ALFA toolkit allows multiple observations of the consultation to be aggregated, simultaneously navigated, and output into other applications. The output from the ALFA tool should provide the evidence, based on which improved technology and models for the consultation can be developed.

## Multimedia Appendix 1

Technical design of ALFA toolkit

## Multimedia Appendix 2

Comparison of ALFA toolkit features with existing tools

## Multimedia Appendix 3

Sample multichannel video file

## Multimedia Appendix 4

Demonstration video of navigable occurrence graph created by LFA stage

## Multimedia Appendix 5

Source code for LFA tool

## Multimedia Appendix 6

Source code for UAR tool

## Abbreviations

ALFA: activity log file aggregation
BP: blood pressure
CHUI: character user interface
COREC: Central Office for Research Ethics Committees
EPR: electronic patient record
GP: general practitioner
GUI: graphical user interface
GPL: general public license
HCI: human computer interaction
LFA: log files aggregation
ODC: observational data capture
PRS: pattern recognition software
UAR: user action recording
UML: unified modeling language
VAR: voice activity recording
XML: extensible markup language
