# Supplementary material for: The ALFA (Activity Log Files Aggregation) Toolkit: A Method for Precise Observation of the Consultation
Source: J Med Internet Res. 2008 Sep 8;10(4):e27. doi: 10.2196/jmir.1080 (PMC2629369; doi:10.2196/jmir.1080)
Supplement: Supplementary file 1 [file jmir_v10i4e27_app1.pdf]

Activity Log File Aggregation (ALFA) toolkit for computer mediated consultation observation

Technical setup

	Stage of ALFA method	ALFA technique	Output
**1.**	**Observation**		
1.1	Audio visual recording	Multi-channel video (MCV) with 3 camera views and screen capture.	Multi-channel video
1.2	Observational data collection	Coding of multi-channel video with event variables representing multiple aspects of interactions	ObsWin log file with event occurrences and durations
1.3	Computer use	Measurement of keyboard and mouse use with User Action Recording (UAR) tool	Time-stamped keyboard and mouse activity log files
1.4	Verbal interactions	Measuring the verbal interactions using Voice Activation Recording (VAR), combed with transcript	Time-stamped conversation log
1.5	Non-verbal interactions	Motion detection using PRS software. (Not reliably used.)	PRS log with time-stamped motion indicators
1.6	Other inputs	Log File Aggregation (LFA) tool current version accepts up to 10 files	
**2.**	**Unification**	Aggregation of time-stamped logs using LFA.	Aggregated output in XML or CSV format
**3.**	**Analysis**		
3.1	Identifying the sequence and patterns of interactions	Modelling of consultation process with UML Sequence diagrams, UML met-model for consultation.	Unified Modelling Language (UML) process models
3.2	Identifying the process map with activity occurrences and durations	Occurrence graph representation of aggregated observational data in LFA application. Video segments linked to events.	Occurrence graph with video segments.

Biomedical Informatics, Division of Community Health Sciences
Tel + 44 (0)20 8725 5661 Fax + 44 (0)20 8725 3584 Email slusigna@sgul.ac.uk

ALFA stage 1: Observation

1.1 Multi-channel video recording

Objective

Audio-Visual recording of computer mediated consultation activities

Setup and process

Three separate video recordings are made simultaneously with digital cameras placed in positions to capture as much of the face and body of the participants as possible. One camera is positioned to record a wide angle view of both the doctor and patient around the desk. Two further cameras then capture the doctor and patient individually. A screen capture software is used to record the computer screen and data entered into the clinical computer system in real-time. All video cameras are placed avoiding views of the examination couch. The whole upper body of the patient should be filmed as the patient's hand movements are often an important part of non-verbal communication.

The cameras are left stationary and running to enable the researchers to leave the consultation and best ensure that the participants are distracted as little as possible by the recording equipment. Two researchers can to set up the cameras and computer software in less than 15 minutes in a standard consulting room.

The final multi-channel footage is composed by merging and synchronising the audio visual recordings of the three cameras and the output of screen capture software. Currently this is done using Adobe Premier Elements 2.0 Software. The video feeds are synchronised by identifying a common sound element in all three videos timed to 1/25 second. The separate channels were then exported into our chosen format and rendered to AV1 (Audio Video Interleave) and transferred to DVD

Hardware/software requirements

3 x standard digital video cameras (Sony DCR HC45E) and recording media (mini DV)
3 x tripods
Screen recorder software e.g. Techsmith Camtasia studio, AviScreen Portable (USB based free software)
Video editing software e.g. Adobe Premier Elements 2.0, Jahshaka 3.0 (open source)

Biomedical Informatics, Division of Community Health Sciences
Tel + 44 (0)20 8725 5661 Fax + 44 (0)20 8725 3584 Email slusigna@sgul.ac.uk

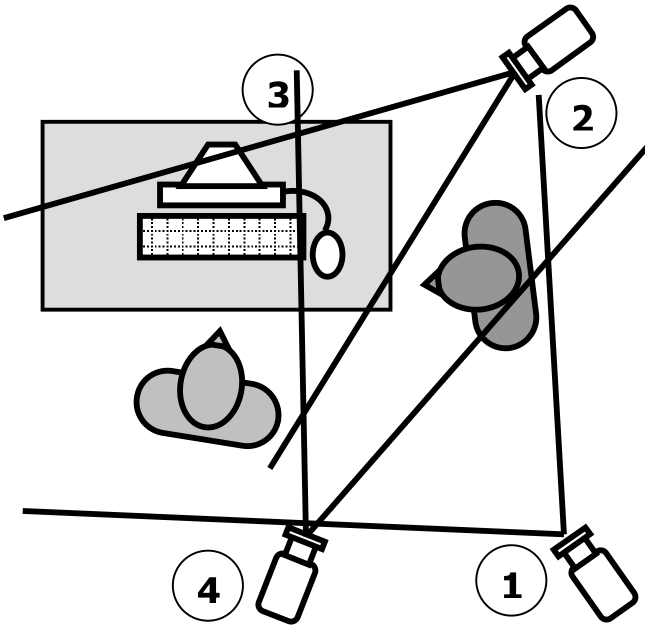

Figure 1: Multi-channel video setup – three camera angles and the screen capture software

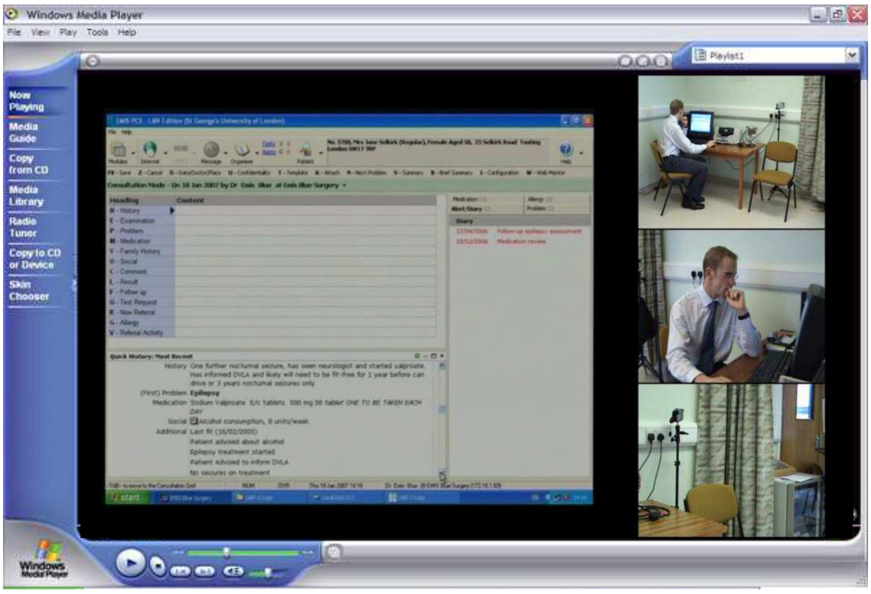

Figure 2: Multi-channel video with combined video and screen capture recordings

1.2 Observational Data Capture (ODC)

Objective

Collection of observational data about doctor-patient and doctor-computer interactions

Setup and process

The multi-channel consultation video is imported into Obswin (an observational data capture tool) in order to measure timings and occurrences of various aspects of the consultation.

First the interactions that need to answer the research question should be identified. If the number of variables is difficult to be measured in a single recording run (n>5), then they should be categorised into groups. Separate recording runs should focus on each group of variables. Each consultation is watched at least once before the actual recording run by each rater, to get familiar with the content of the consultation. When the observation is in progress, the corresponding key is pressed to indicate onset, and pressed again to indicate offset.

Raters receive training about analysing the videos by the use of a written training manual. This should give a more encompassing definition of the variables and sample screen shots to give further clarity to the variables. This reduces the need for an informal teaching process and standardises the training process for all raters. As well as a general training manual, system specific guides will help to familiarise raters with design features unique to the system. The system specific training included a crib sheet for each video which provided a brief summary of the conditions discussed in each consultation.

The results are then combined into one file representing the whole consultation, forming a dataset from which summary statistics and graphs can be produced. Numerical data includes the time interval that a variable occurred over, its percentage interval and the number of it's "on" and "off" sets. After the end of the recordings, the intra class correlation coefficient was then calculated for each variable across all videos. Data can be displayed as occurrence graphs, displaying various activities that occur in the consultation. This linear form of representation shows the proportionate times of specific activities within a consultation and how these relate to each other.

Hardware/software requirements

ObsWin or similar observational data analysis tool

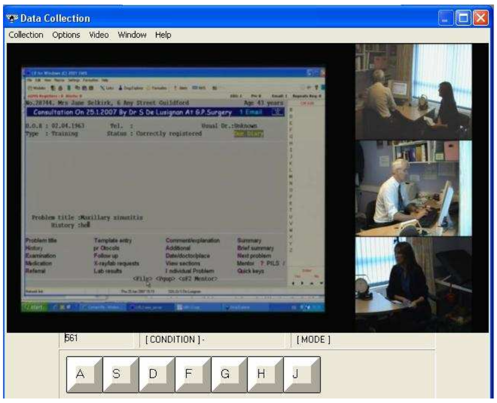

Figure 3: ObsWin rating interface

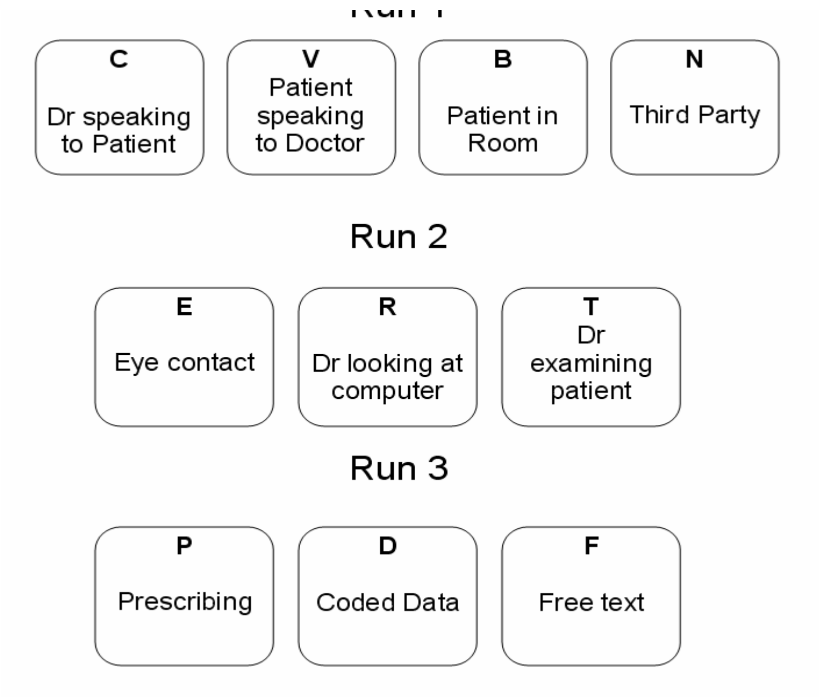

Figure 4: Rating scale used for a pilot study: Variables and their descriptions

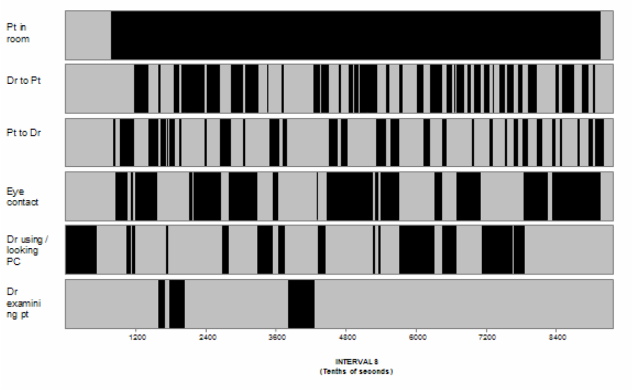

Figure 5: Summary bar chart of a rating

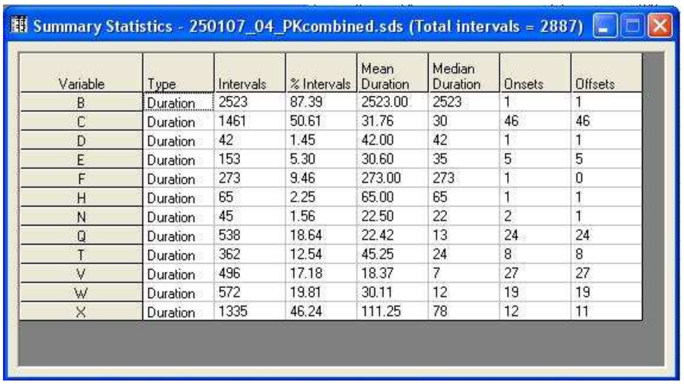

Summary Statistics - 250107_04_PKcombined.sds (Total intervals = 2887)

Variable	Type	Intervals	% Intervals	Mean Duration	Median Duration	Onsets	Offsets
B	Duration	2523	87.39	2523.00	2523	1	1
C	Duration	1461	50.61	31.76	30	46	46
D	Duration	42	1.45	42.00	42	1	1
E	Duration	153	5.30	30.60	35	5	5
F	Duration	273	9.46	273.00	273	1	0
H	Duration	65	2.25	65.00	65	1	1
N	Duration	45	1.56	22.50	22	2	1
Q	Duration	538	18.64	22.42	13	24	24
T	Duration	362	12.54	45.25	24	8	8
V	Duration	496	17.18	18.37	7	27	27
W	Duration	572	19.81	30.11	12	19	19
X	Duration	1335	46.24	111.25	78	12	11

Figure 6: Frequency table a rating

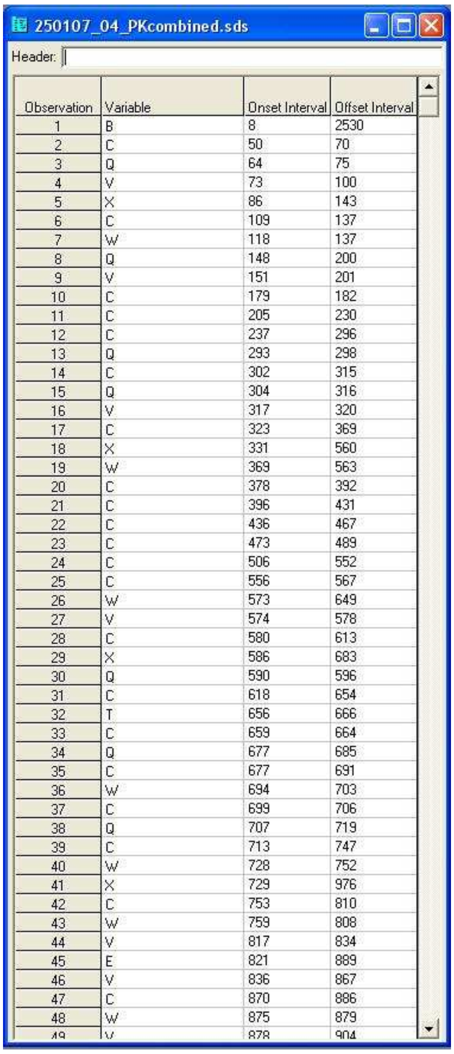

250107_04_PKcombined.sds

Header:

Observation	Variable	Onset Interval	Offset Interval
1	B	8	2530
2	C	50	70
3	Q	64	75
4	V	73	100
5	X	86	143
6	C	109	137
7	W	118	137
8	Q	148	200
9	V	151	201
10	C	179	182
11	C	205	230
12	C	237	296
13	Q	293	298
14	C	302	315
15	Q	304	316
16	V	317	320
17	C	323	369
18	X	331	560
19	W	369	563
20	C	378	392
21	C	396	431
22	C	436	467
23	C	473	489
24	C	506	552
25	C	556	567
26	W	573	649
27	V	574	578
28	C	580	613
29	X	586	683
30	Q	590	596
31	C	618	654
32	T	656	666
33	C	659	664
34	Q	677	685
35	C	677	691
36	W	694	703
37	C	699	706
38	Q	707	719
39	C	713	747
40	W	728	752
41	X	729	976
42	C	753	810
43	W	759	808
44	V	817	834
45	E	821	889
46	V	836	867
47	C	870	886
48	W	875	879
49	V	878	904

Figure 7: Rating table with variables and their onset-offset values

1.3 User Action Recording (UAR)

Objective

To record the clinician's use of computer keyboard and mouse during the consultation

Setup and process

User Action Recorder (UAR) is a data collection tool that has been used for analysis of the consultation process. When activated, this programme captures keystrokes and mouse movements. The value of the pressed key and the co-ordinates of the mouse pointer are written into two separate log files with time-stamps. The software is copied into the doctor's computer before the start of the consultation recording session. It is activated before the first consultation, and left running until the end of the recording session. The two files representing the keyboard (keyboard.txt) and mouse activities (mouse.txt) are then copied for analysis. Used in conjunction with multi-channel video, UAR can be used to accurately calculate the time taken to complete various computer activities. Although observer rating of computer use was unreliable, ObsWin software can be used to play back the consultation videos at variable speeds. UAR is then used to accurately calculate time taken for various computer activities, including coded data entry, free text, prescriptions and referrals.

E.g: Calculating time taken for prescription of medication.

-The consultation video is imported into Obswin software and then scrolled through to the point where prescriptions occur.

-In the instance of prescription, the start of the process was taken as the mouse click / key stroke used to launch the prescription template, and the end of the process taken as the mouse click / keystroke used to exit the template / save the data.

-The time-stamp on the multi-channel video screen is then correlated to the UAR log files.

-The time taken for that particular sequence of mouse clicks and keystrokes can then be calculated from the log file time-stamp.

Use of an Excel Macro makes the calculation process and the identification of the interactions segments efficient. After series of analysis, a time gap of more than 3 seconds between two adjacent entries was identified as an indicator for a possible break in doctor-patient interaction.

Hardware/software requirements

UAR software

Biomedical Informatics, Division of Community Health Sciences
Tel + 44 (0)20 8725 5661 Fax + 44 (0)20 8725 3584 Email slusigna@sgul.ac.uk

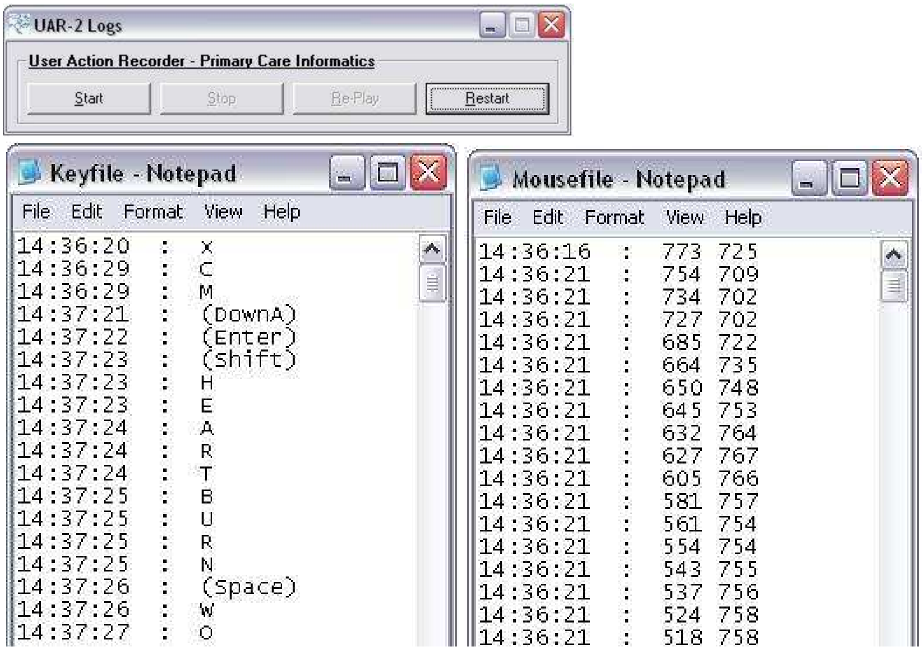

Figure 8: UAR recorder interface and two sample log files created for keyboard and mouse use

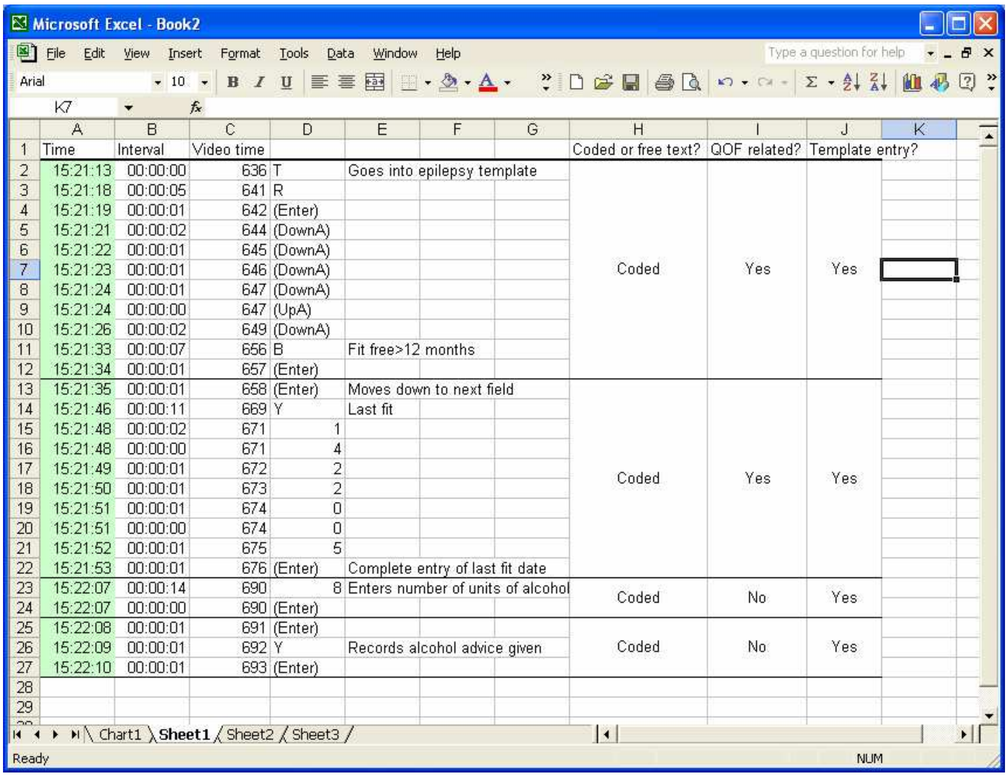

	A	B	C	D	E	F	G	H	I	J	K
1	Time	Interval	Video time					Coded or free text?	QOF related?	Template entry?	
2	15:21:13	00:00:00	636	T	Goes into epilepsy template						
3	15:21:18	00:00:05	641	R							
4	15:21:19	00:00:01	642	(Enter)							
5	15:21:21	00:00:02	644	(DownA)							
6	15:21:22	00:00:01	645	(DownA)							
7	15:21:23	00:00:01	646	(DownA)				Coded	Yes	Yes	
8	15:21:24	00:00:01	647	(DownA)							
9	15:21:24	00:00:00	647	(UpA)							
10	15:21:26	00:00:02	649	(DownA)							
11	15:21:33	00:00:07	656	B	Fit free>12 months						
12	15:21:34	00:00:01	657	(Enter)							
13	15:21:35	00:00:01	658	(Enter)	Moves down to next field						
14	15:21:46	00:00:11	669	Y	Last fit						
15	15:21:48	00:00:02	671	1							
16	15:21:48	00:00:00	671	4							
17	15:21:49	00:00:01	672	2							
18	15:21:50	00:00:01	673	2				Coded	Yes	Yes	
19	15:21:51	00:00:01	674	0							
20	15:21:51	00:00:00	674	0							
21	15:21:52	00:00:01	675	5							
22	15:21:53	00:00:01	676	(Enter)	Complete entry of last fit date						
23	15:22:07	00:00:14	690	8	Enters number of units of alcohol			Coded	No	Yes	
24	15:22:07	00:00:00	690	(Enter)							
25	15:22:08	00:00:01	691	(Enter)							
26	15:22:09	00:00:01	692	Y	Records alcohol advice given			Coded	No	Yes	
27	15:22:10	00:00:01	693	(Enter)							
28											
29											

Figure 9: Analysing of a Keyboard use log file

1.4 Voice Activity Recording (VAR) & Consultation transcripts

Objective

To create a time-stamped transcription of the consultation

Setup and process

The VAR tool monitors the verbal interactions and creates a log file by analyzing the sound level of the recorded video. Certain amount of noise reduction from the environment can be done by adjusting the silence level and gain level. Setting the sample size adjusts the overall sensitivity of the tool to the voice levels. Its output is a log file with time-stamps indicating possible start and end times of verbal interactions. Though we attempted to identify possible software techniques to differentiate doctor's and patient's voices; variations in consultation room layouts, intrusiveness of dedicated voice recording hardware, pre-recording training requirements of software and time constrains in consultation sessions reduced their utility.

VAR log improves the efficiency of consultation transcription process, as it indicates the occurrences of doctor-patient verbal interactions to the transcriber directly. We have found the VAR to provide a precise time-stamp of when speech starts – and the transcribers can easily type the audio output from the consultation against the correct time-stamp. This provides us with text which can be synchronised with any of the other observations.

The VAR also enables us to identify who initiates and terminates silence. We have observed how the clinician sometimes makes purposeless use of the IT to initiate silence to control the consultation. The format of the VAR log is designed to be compatible with most of the transcribing tools, and it is easily customisable. It has header fields which details format of the data recording. We have successfully imported this to the 'Subtitle Worksop' application, which links the VAR entries directly into the video segments.

Hardware/software requirements

Multi-channel video recordings or separate audio recording

VAR software

Transcriber tool or video subtitle editing tool with import-export features

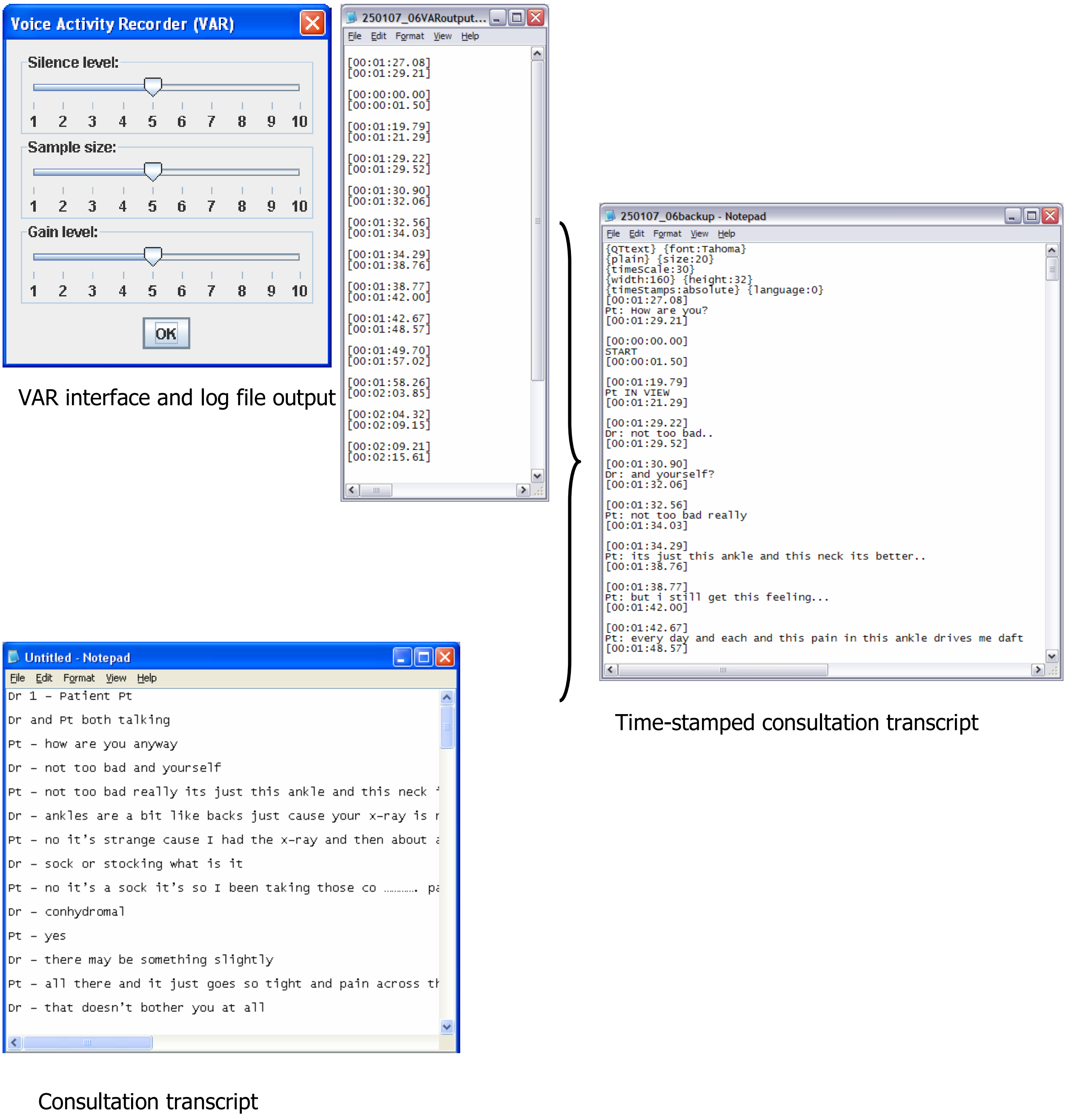

Figure 10: Creation of the time-stamped consultation transcripts

ALFA stage 2: Unification

Objective

To combine the activity log files created by multiple observation techniques into single data file

Setup and process

Unification is done using the Log File Aggregation (LFA) tool. This allows importing of any number of time-stamped log files. It was developed based on Object Oriented Programming (OOP) principles, making it easily customisable to handle different log file formats. This tool enables the researchers to isolate and review doctor-computer-patient interactions efficiently. The number of log files that can be imported are currently set to 10, but this is limited only by the amount of display area available for the aggregated output. Once the number of log files the LFA tool needs to combine is configured, it allows the user to select the data format for each file. Currently this is set to import ObsWin, UAR (User Activity Recording), VAR (Voice Activity Recording), XML and PRS (Pattern Recognition Software) data files. Next is the combination stage, which produces an unified output in the background. User can then either opt to view the aggregated output using number of formats available or export it. Combined output could be saved as text, ObSwin, or XML file formats. The Java XML package facilitates the conversion of aggregated output into XML format. This single output represents the data from multiple log files of different formats as a single transferable data file. This also enables the communication between different XML reader applications. This tool removes the risk of human error involved in manual linkage of observation files or the time taken for comparing observations of different measurements.

Hardware/software requirements

LFA tool

Java Media Framework

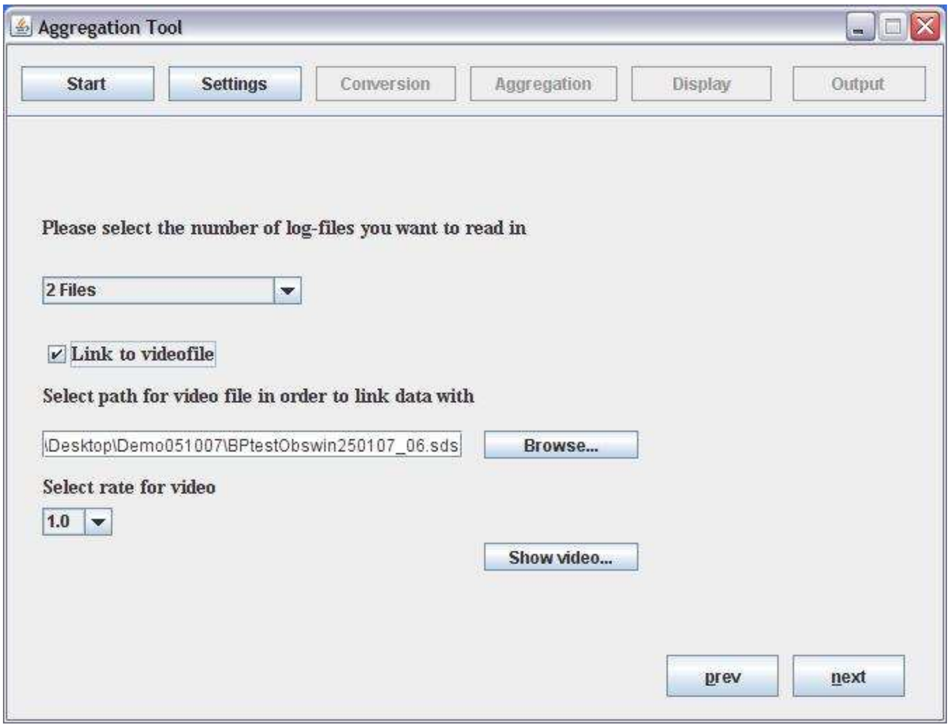

Figure 11: The LFA (Log File Aggregation) tool interface

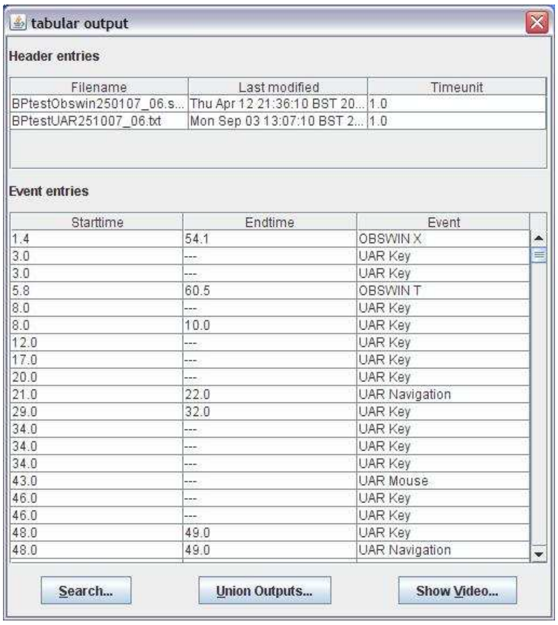

Figure 12: Aggregated output from the LFA tool

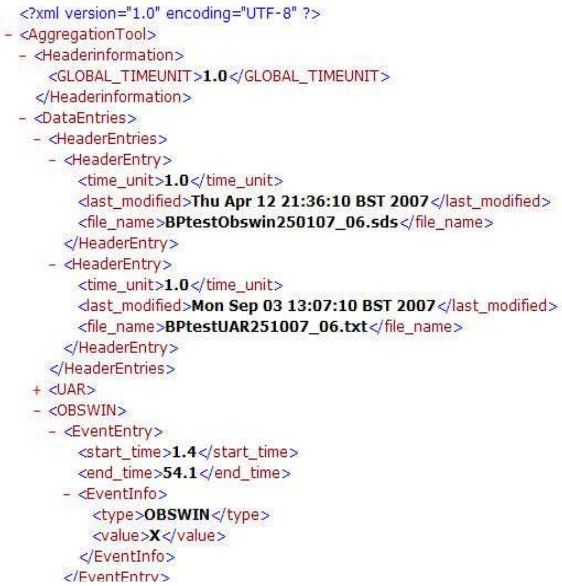

Figure 13: XML output of an aggregation from the LFA tool

ALFA stage 3: Analysis

3.1 Occurrence graph with video segments

Objective

To create a process map with computer mediated consultation activity occurrences and durations

Setup and process

This analysis stage is integrated into the LFA tool. The aggregated output created by the LFA tool can be viewed as histograms representing interaction frequencies or durations or as occurrence graphs in its 'Display' stage. The latter output produces a navigable graphical process map of the consultation. The power of this output in analysis is that, clicking on a rectangle representing a specific variable occurrence in the occurrence graph of any of the files imported, takes the user directly to the appropriate location in the multi-channel video's timeline. This enables users to navigate into any segment in the consultation they wish to study and simultaneously view all the log files relating to that point in time. Histogram output provides an analysable overview of activities that occurred in the consultations and their duration's quantitatively. For the UAR log entries this represents the number of keyboard interactions differentiating them as character key presses, navigation using arrows, corrections or mouse clicks. This also has a search facility, which allows the researcher to identify a particular set of interactions within a specified time segment. Comparing occurrence graphs of multiple consultations clearly indicates the characteristics of each session in the context of both doctor-patient social relationship aspect as well as the doctor-computer information system aspect.

Hardware/software requirements

LFA tool

Java Media Framework

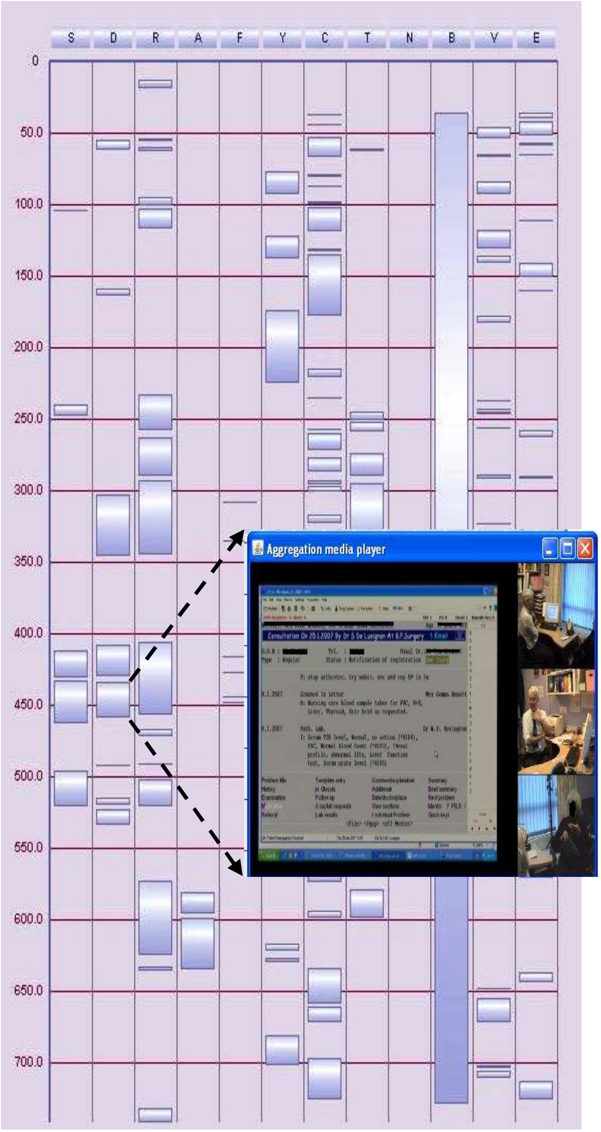

Figure 14: The Occurrence graph output; clicking on an activity rectangle brings up the corresponding video segment

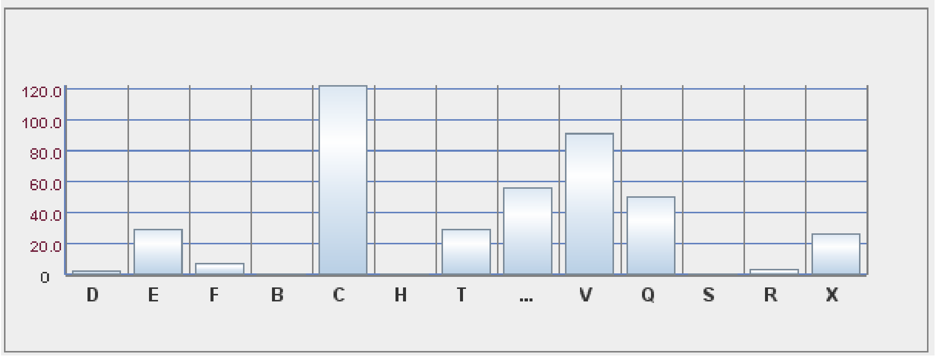

Figure 15: A histogram output produced by LFA tool showing the activity ; clicking on an activity rectangle brings up the corresponding video segment

3.2 Occurrence graph with video segments

Objective

To synthesise the consultation observations into an analysable process model of doctor-computer-patient interactions, with features interpretable by clinical system developers

Setup and process

This analysis stage of ALFA is performed using the 'Sequence diagram' graphical notation of the Unified Modelling Language (UML). We use UML 2.0. This UML process model represents the principal activities within the consultation, complexity of the interactions and how the sequence of activities is progressively defined. We can analyse how the interaction patterns may be dependent on the design of the clinical system and their effect on patient centeredness. The sequence diagram creation is a three stage process. First the basic interaction framework is plotted using the LFA occurrence graph output. This process can be semi-automated with the use of its XML output. This marks the start and end times of doctor-patient and doctor-computer interactions. We plot these findings into the doctor and computer lifelines in the 'Sequence diagram'; this defines their focus of control boxes and the message passing arrows between them. Direction of the arrows indicates the originator of each interaction. We then review the UAR and VAR log files to add precise details about the nature, objectives and relationship of identified verbal interactions of doctor-patient and doctor's use of the keyboard. The times-tamps for these activities are extractable from the XML output. We could then annotate the sequence diagram to explain the data flows associated with each interaction. Further enhancements of the sequence diagram skeleton using the multi-channel video outputs are aimed at informing the interdependence of each interaction. We viewed the computer interface activity of the multi-channel code to identify the characteristics of doctor and computer interactions associated with the clinical system brands..

Hardware/software requirements

UML modelling tool, e.g. Star UML

Aggregated outputs of LFA

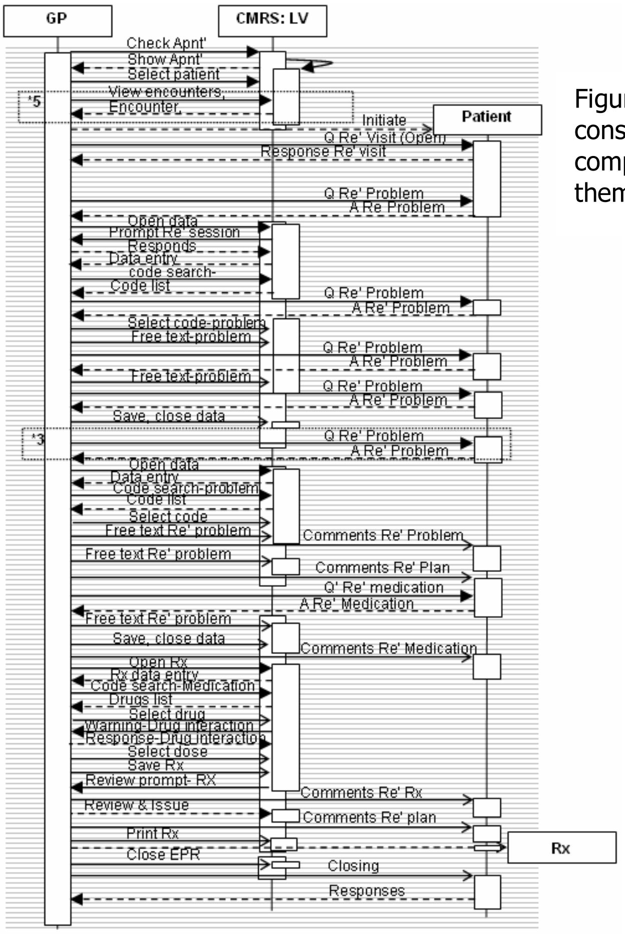

Figure 16: An UML Sequence diagram of a consultation; three life lines represent the doctor, computer and the patient. Arrows passing between them represents the interactions.

Figure 17: UML Sequence diagrams comparing the blood pressure data entry task in four different EPR systems.

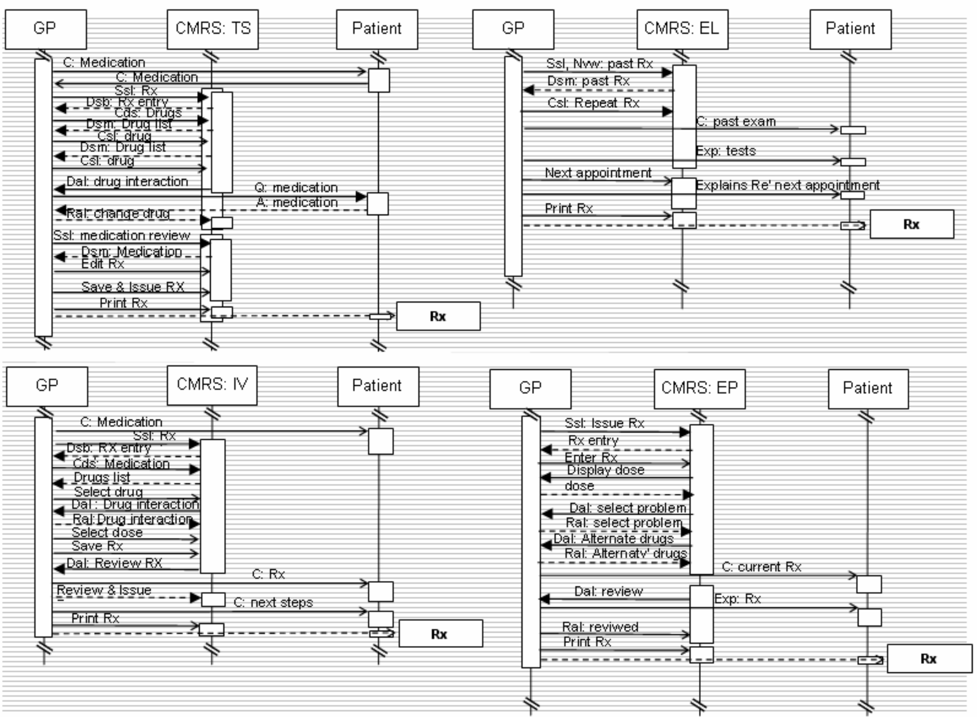

Biomedical Informatics, Division of Community Health Sciences
Tel + 44 (0)20 8725 5661 Fax + 44 (0)20 8725 3584 Email slusigna@sgul.ac.uk
