# Supplementary material for: The ALFA (Activity Log Files Aggregation) Toolkit: A Method for Precise Observation of the Consultation
Source: J Med Internet Res. 2008 Sep 8;10(4):e27. doi: 10.2196/jmir.1080 (PMC2629369; doi:10.2196/jmir.1080)
Supplement: Supplementary file 2 [file jmir_v10i4e27_app2.pdf]

St George's
University of London

Comparison of Activity Log File Aggregation (ALFA) toolkit features with existing tools

Background

The ALFA toolkit supports three steps in consultation analysis. (1) Observation: Recording of doctor-patient and doctor-computer interactions using video and other observational software, (2) Unification: Combining multiple observations into a single analysable format, (3) Analysis: Use of aggregated outputs to study consultation tasks.

The ALFA toolkit was developed because we were unable to identify any other application which would meet our requirements. This document details the other applications we considered.

Objective

To identify whether there are existing applications we could use to capture the detailed interactions in the consultation; and facilitate the creation of UML sequence diagrams.

Method

We first mapped the existing tools to the different stages of the ALFA method. This was then followed by a detailed review of strengths and weaknesses of each approach in the context of the clinical consultation observation. Selected applications are broadly from four categories; Qualitative research, transcription based analysis, usability testing and screen capture based observation. We included widely used applications from each category for this review. After a short introduction of the selected tool, its functionalities are compared under subheadings representing the main modules of the ALFA approach.

Results

This appendix contains descriptive data which compare the ALFA toolkit with other packages: (1) Atlas ti and (2) NVivo – qualitative research applications; (3) Transana - A transcription analysis tool; (4) Morae – A usability application; and (5) Camtasia, Adobe captivate and BB Flash Back – Screen casting software. Their very different functionalities, compared with the ALFA tool are summarised in table 1.

Conclusions:

We were unable to identify an "off the shelf" application with the functionality set out in our specification.

Biomedical Informatics, Division of Community Health Sciences
Tel + 44 (0)20 8725 5661 Fax + 44 (0)20 8725 3584 Email slusigna@sgul.ac.uk

Table 1: Summary of the state of the art review of ALFA toolkit

ALFA element and comparable functionality	Existing tools							ALFA tool kit
	Qualitative research		Transcription & analysis	Usability testing	Screen casting			
	ATLAS.ti	NVivo	Transana	Morae	Camtasia	Adobe Captivate	BB F'Back	
**1. Multi channel Video (MCV) recording**								
Screen capture	N/A	N/A	N/A	Yes	No	No	Yes	Yes
Video capture	N/A	N/A	N/A	1 camera	No	No	1 camera	3 cameras
Audio capture	N/A	N/A	N/A	Yes	Yes	Yes	Yes	Yes
**2. Observational Data Capture (ODC)**								
Multimedia file import	Yes	Yes	Yes	Yes	No	No	No	Yes
Sufficient video display area	Yes	Yes	No	No	N/A	N/A	N/A	Flexible
Video playback controls	Yes	Yes	Yes	Limited	N/A	N/A	N/A	Yes
Direct export of interaction durations	No	No	No	No	N/A	N/A	No	Yes
Method of coding for interactions	Codes, Memos	Codes, Memos, Nodes	Keywords, Comments	Markers	No	No	No	Duration variables
Interaction durations visible	No	Yes	No	Yes	No	No	Yes	Exports directly
**3. User Activity Recording (UAR)**								
Computer keyboard use	N/A	N/A	N/A	Yes	No	No	Yes	Exports directly
Computer mouse use	N/A	N/A	N/A	Yes	Yes	Yes	Yes	Exports directly
Interaction	No	No	No	No	No	No	No	No

durations								
Lightweight to install & run	N/A	N/A	N/A	No	No	No	No	Yes
**4. Voice Activity Recording (VAR) and transcription**								
Indicates voice levels	No	No	Yes	No	No	No	Yes	Yes
Measures verbal interactions	No	Manual	Manual	No	No	No	Manual	Exports directly
Import/create transcriptions	Yes	Yes	Yes	No	No	No	No	Yes
Time stamped transcriptions	No	Yes	Yes	No	No	No	No	Yes.
**5. Log File Aggregation**								
Combine data from different tools	video & transcriptions	video & transcriptions	video & transcriptions	Screen capture & video	No	No	No	Up to 10. Can extend further
Single exportable file	Yes	No	No	No	Yes	Yes	Yes	Yes, many formats
XML output	Yes	No	No	No	No	No	No	Yes
**6. Occurrence graphs**								
Time lines for interaction	No, Network diagrams	Yes, small display area	No, Clips organised with labels	1 timeline	No	1 timeline	mouse, keybrd' & voice	Multiple timelines. Large display area
Interactions mapped to video	Yes	Yes	Yes	Yes	No	No, to screen capture	No, to screen capture	Yes
Interaction durations linked to video segments	No	Yes	No	Yes	No	No, linked to frame	No, linked to frame	No
**7. UML process modelling**								
Use for UML validation	Limited	Limited	No	Limited	No	No	No	No
Indicates	No	Yes,	No	Yes, 1	No	No	Only for	Yes, multiple

interactions and durations in channels		limited by display area		at a time			mouse, keyboard & voice	channels of interactions
Shows interaction type directly	No, Using codes	Yes	No. Using labels	No, Using markers	No	No	Mouse, keyboard & voice	Yes. For all recorded interactions

1.0 ATLAS ti

www.atlasti.com

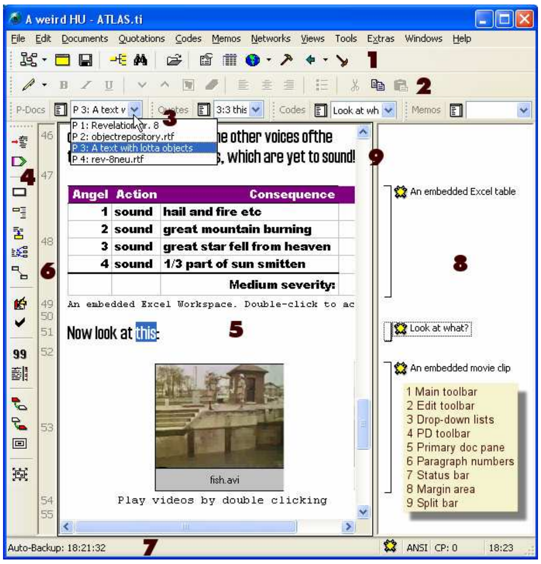

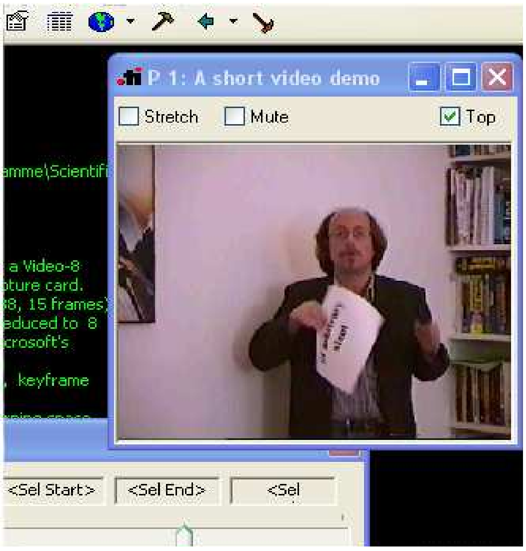

Introduction

The manufacturer describes this as a knowledge workbench software. The main objective is to provide a systematic method of analysing the text and multimedia data. This is particularly useful in gleaning analysable results from large volumes of documents, images, audio or video data. The primary mechanism is locating useful information, coding and annotating them, and facilitation of their organisation in a searchable way. Creation of Network models assists to visualise the conceptual relationships between the observed materials.

1.1 Multi Channel Video recording

This does not have any data recording mechanisms, mainly an analysis tool for collected data objects. Only the combined multi-channel video can be imported as a video file. Interface does not provide large enough space to review the combined video.

1.2 Observational Data Capture

Facilities available may depend on the compression of the video file or the availability of the drivers to support the media format. Frame level navigation may not be available for compressed videos. Overall, the rich set of coding and organising facilities available are more optimised for textual materials. Inconsistent use of video frame and video time for linking of observations makes the analysis unwieldy; i.e. the 'Quotation manager' feature uses video frames as the unit, whereas the 'Media controller' can use either time or frames. While the use of codes can be useful to classify interactions in an organised manner, it is difficult to obtain durations for interactions directly.

1.3 User Action Recording (UAR)

This is not an interaction recording tool. Alternative use would be to observe the log file to categorise computer use patterns. This cannot import and process a computer activity log file directly. However, it might be possible to import this as a textual object and code manually. In ALFA approach this is measured objectively and directly recorded in an analysable format.

1.4 Voice Activity Recording (VAR) and transcription.

No comparable feature to VAR exists. This has facilities to work with both transcriptions and audio files. However, it is not optimised to combine a full transcription to an entire audio file. Instead it's more efficient to link audio segments to fragments of transcriptions using its 'Hypertext' function. This is a tool to analyse transcriptions or audio segments, rather than offering facilities to transcribe.

1.5 Log File Aggregation (LFA)

Comparable function in ATLAS.ti is the grouping of Codes into families. In ALFA, quantitative observational outputs of different formats are aggregated into a single analysable overview. In ATALS, the grouping is done in a conceptual level among similar format of observations. This does not require any conversion functionality since the data origin is within the same application.

1.6 Activity mapping with occurrence graphs

In ALFA, the occurrence graph provides the analysable overview of the observations. In ATLAS.ti the Network diagrams provides a similar overview. But the latter is built by the researcher, based on the concepts visible through codes or memos. ALFA occurrence graph is directly generated from the aggregated outputs. Relationships among measurements are visible directly. The view can be modified by adding or removing measurement variables. ATLAS.ti networks need to be re-drawn to obtain different views. Both approaches are similar in available data export formats; XML, CSV, SPSS etc. Object oriented design in ALFA, enables introduction of new formats with minimum effort.

1.7 UML modelling

ATLAS.ti 'Codes', Memos' and 'Network diagrams' may become useful for creation of the UML sequence diagram. However, ALFA occurrence graph and aggregated logs provide direct visual cues and duration measurements respectively for the UML Sequence diagram generation. Cross validation and modification of the Sequence diagram is easier in ALFA approach.

2.0 NVivo

www.qsrinternational.com/products_NVivo.aspx

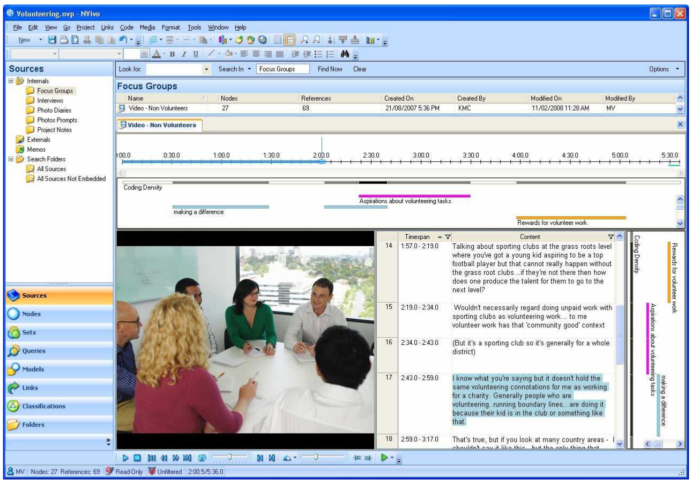

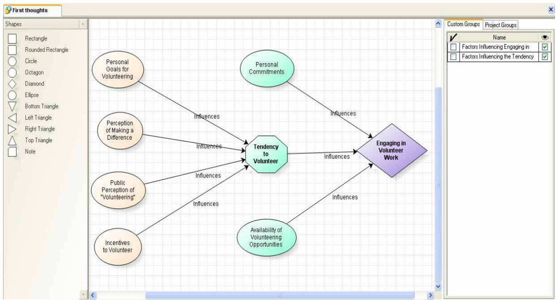

Introduction

NVivo provides facilities to code, classify, sort and organise data to collect information from rich or plain text documents, audio, video files and images. Classification structures maintain coding consistency throughout the project. Annotations and memos link the thought or observations to the materials. Links connect items based on themes. NVivo coding stripes provide visual cues about the project for easy comparison. Nodes provide mechanisms for storing ideas and coding. Its built in search engine provides facility to query coded data. Special functions are available for group based analysis. Models and charts display the projects information, connections and findings.

2.1 Multi Channel Video recording

No recording elements for video data exist. It has a flexible fully featured embedded video player. To get the full potential from this tool, each of the four channels of the MCV would need to be imported separately.

2.2 Observational Data Capture

This has a rich set of observation and coding tools. Similar to the ObsWin approach, codes are linked to the video time-line. These codes can be then organised into 'Nodes' for

summarising the observations. NVivo has more video control facilities than ObsWin. Slow motion playing, looping, skip playing and variable play back speeds exist. The ObSwin coding method using the keyboard key presses is simpler than NVivo.

2.3 User Action Recording (UAR)

No methods exist to import activity log files. Any recording of doctor-computer interactions would need to be done manually.

2.4 Voice Activity Recording (VAR) and transcription.

This can import transcriptions to link with a video or provides facilities to transcribe based on imported video or audio files. Transcriptions are linked to segments of the video shown as 'Timespans'. This is linked to the timeline of the video. This does not provide automated measurements based on audio levels like the VAR tool.

2.5 Log File Aggregation (LFA)

No specific aggregation occurs except linking the transcript to the video. No format differences exist as the data capturing take place within the tool. Importing the external observational data is difficult. No features available to customise data importing routines. LFA can easily be modified to handle different data formats. NVivo does not support XML data export. Each observational item needs to be exported separately or printed.

2.6 Activity mapping with occurrence graphs

NVivo provides Coding strips. This shows the location and duration of the codes linked to the video. However the number of coding strips visible or manageable is fewer than the ALFA approach. This method is not efficient to display shorter duration of interactions.

2.7 UML modelling

This does not provide occurrence or interaction duration data to generate UML diagrams directly. Coding strips may be a useful visual framework to generate the Sequence diagram. However the amount of coding strips needed to identify the interactions and their purposes would be complicated to handle by the NVivo interface.

3.0 Transana

www.transana.org/index.htm

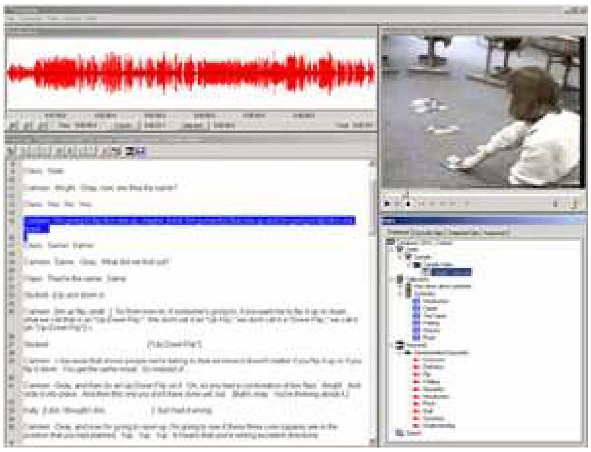

Introduction

This tool's main focus is to facilitate transcription of audio or video clips to support their qualitative analysis in a systematic way. It provides facilities to annotate segments of videos, introduce keywords and to manage them based on file descriptives. Multiple clips or their segments can be grouped together into 'collections'. Codes can be applied for the videos, but this needs to be done at the clip level. Text based reports provide details about each clip such as associated keywords, transcriptions, file descriptors, comments etc. Graphical reports represent the placement of keywords against the clip's time-line. Additional facilities are available to transfer video files in an encrypted format, presentation of analysed videos and ability to support multiple users simultaneously.

3.1 Multi Channel Video recording

Transana does not have a recording element. This is a tool for viewing of pre-recorded videos.

3.2 Observational Data Capture

Researchers can use Transana to import a multi-channel video into its integrated video player. However, any analysis based on time durations cannot be done directly. An

alternative method is to divide the video file into mini clips, and tagging them with keywords, or to attach additional comments. The size of the video player is not sufficient, and not having visual indications about the interactions being studied would effect negatively on the inter-rater reliability. Ability to see the audio level may increase reliability of rating verbal interactions. In ALFA method this is done by a dedicated tool in a semi-automated manner.

3.3 User Action Recording (UAR)

This neither provides facilities to record keyboard, mouse interactions directly nor to import such activity log files. This needs to be done by observing the video clip, and if done so, the result would be a series of labels attached to the clips.

3.4 Voice Activity Recording (VAR) and transcription

This has a voice level indicator based on the audio stream of the imported multimedia file. Main emphasis is on the qualitative assessments of the conversations. It cannot supply a time-stamped transcript. Transana provides ability to include speech characteristics to the transcriptions, e.g. Jeffersonian Notation. This is not currently considered in the ALFA approach.

3.5 Log File Aggregation (LFA)

Not an automated process. Linking the transcripts and labels to video clips is comparable to the 'Unification stage' of the ALFA method. However, in the ALFA method this involves automated aggregation of multiple observation outputs.

3.6 Activity mapping with occurrence graphs

Analysis needs to be done based on the keyword/label based organisation of the clips. It is difficult to analyse interactions within a consultation into a detailed level. There is no comparable output to the LFA occurrence graph or to the histograms. Observers can jump to selected clips based on the clip management structure, but not to segments within the clip.

3.7 UML modelling

Not efficient to use as a data source for UML modelling. Even if done, it would be difficult to validate as the tool does not provide objective measurements for interactions.

4.0 Morae

www.techsmith.com/morae.asp

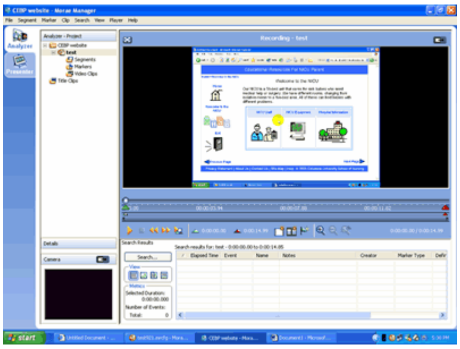

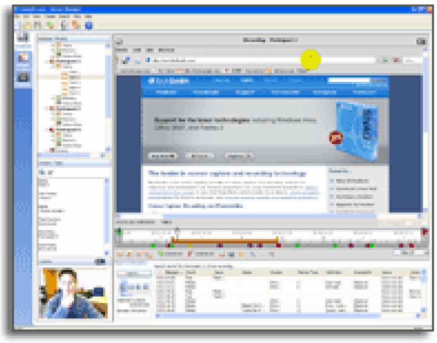

Introduction

Morae is an usability testing suite for software applications and web sites. This consists of three components; (1) 'Morae recorder' for data collection, (2) 'Remote viewer' for observation of participants and their interactions remotely, (3) 'Manager' for presentation of observation in an analysable format. A combination of these three modules means, Morae can be used for live usability testing with or without instructions given by a facilitator.

4.1 Multi Channel Video recording

Morae recorder has audio-video recording elements. This uses screen recorder, single webcam and microphone connected to the user's computer. Output of the video appears in the Morae viewer element together with the view of the test participant's computer screen. This could not be used to observe in multiple camera angles in its live mode. If pre-recorded multi-channel video is uploaded to the viewer module, the amount of observation area is not sufficient for a detailed interpretation.

4.2 Observational Data Capture

The comparable functionality is the adding of event markers to the videos viewed through the Morae viewer. Each marker is attached to the selected video frame. Similar to the duration variables used in the ALFA approach, markers in Morae can be predefined to maintain consistency across multiple observers. However, marker selection needs to be done from a drop down list which would be difficult to do if number of overlapping interactions are

being observed. Output formats of observations are more efficient for qualitative analysis. Can not produce tabular or occurrence graph outputs.

4.3 User Action Recording (UAR)

Morae provides facilities to monitor mouse clicks or keystrokes, text, window events, closing or opening of applications. Mouse recorder captures click events. Mouse positions are displayed visually in the observation view. In ALFA method, UAR provides a facility to replay the mouse movements as well as time-stamped mouse coordinate values enabling further quantifiable analysis, e.g. mouse travel distance, click frequency. Majority of the Morae event capturing mechanisms are optimised for web pages. This is less helpful for the clinical consultation systems, where interfaces are much more complex and no API accessibility to identify application events are available. In Morae, recorded activities are linked to the observation segment. In ALFA method, since the computer interactions and observational data capturing is done in independent separate modules, it is possible to analyse the interactions in a flexible manner according to the research needs.

4.4 Voice Activity Recording (VAR) and transcription.

Transcription is fully manual. It uses the recorded audio stream of the observation session. Amount of information available might not be sufficient to transcribe a conversation between doctor and patient in a measurable format. No facility to link the transcription to the video segments. Alternative method would be to link the transcription with annotations or as 'tasks'.

4.5 Log File Aggregation (LFA)

Recording consist of the video capturing of user activity and the web camera feeding. No separate aggregation takes place since the additional observations are directly coded into the video. This limits the number of parallel observations that can be combined.

4.6 Activity mapping with occurrence graphs

Morae manager a has facility to navigate directly to the video segment by clicking on a marker. This is similar to the ALFA functionality. However, all the markers are positioned in a linear manner, making the number of navigable positions limited. In ALFA occurrence graph this can be done two dimensionally. Both have the zooming functionality for the timelines. LFA tool has a higher level of searching functionality, and also a facility to combine search results. Morae does not generate combined outputs in a self explanatory format like XML, only data tables can be downloaded.

4.7 UML modelling

This needs to be done after an initial round of observation where markers are positioned for each interaction. In ALFA approach, UML modelling and validation are supported by the time-stamped UAR, VAR log files and the LFA occurrence graphs. Using Morae would need more processing time.

Biomedical Informatics, Division of Community Health Sciences
Tel + 44 (0)20 8725 5661 Fax + 44 (0)20 8725 3584 Email slusigna@sgul.ac.uk

4. Camtasia, Adobe Captivate and BB Flash Back

www.techsmith.com/camtasia.asp

www.adobe.com/products/captivate/

www.bbsoftware.co.uk/BBFlashBack.aspx

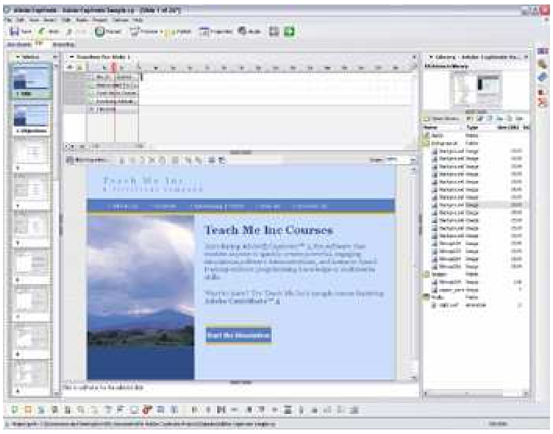

Techsmith Camtasia

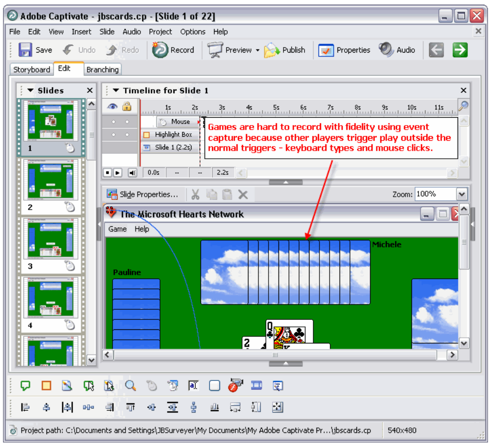

Adobe Captivate

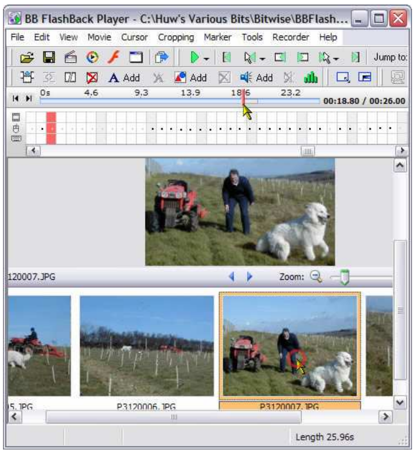

BB FlashBack

Introduction

These are typical examples for popular screen recording software tools. Main objectives of this category of tools are to present screen activities in a format that can be replayed as a movie. Their main applications are software demonstrations, video tutorials, training materials or screencasts. While main functionally is to record the screen sequences, some do have additional

facilities to add annotations, audio streams, display mouse movements and to edit movie segments.

5.1 Multi Channel Video recording

Primary data capture element is the screen recording. In BB FlashBack, single web cam stream can be linked in. In ALFA method, in addition to the screen capture three additional video streams are combined to obtain a multi-channel video stream.

5.2 Observational Data Capture

The only mechanism to rate different segments of the video is by adding text notes (call out boxes) into the video stream. Limited level of clip organising facilities exists. Their main limitation compared to data capture approach in ALFA, is the non-existence of data recording mechanism that runs parallel to the video stream. However, existence of the mouse, keyboard interaction and audio time lines in BB FlashBack may support accurate recording of doctor-computer interactions. This feature is available in the VAR and UAR modules of ALFA.

5.3 User Action Recording (UAR)

All three software capture the mouse movements visually together with the screen activity recording. In BB FlashBack, mouse and keystrokes are available as separate timelines. Measuring the amount of interactions relative to video segment would require manual data collection by looking at these timelines. No features are available to extract the mouse or keyboard interactions for detailed analysis.

5.4 Voice Activity Recording (VAR) and transcription.

No features are provided for transcription. However, availability of voice activity time line in BB Flashback may assist in identifying the start and end time of the verbal interactions to a similar accuracy level to the VAR tool. Unlike VAR, no quantifiable measurements are directly available.

5.5 Log File Aggregation (LFA)

Outputs from all observation techniques available in this category of software, are integrated to the screen recording file. Facilities for customisation of rating scale to suit the research objectives are limited.

5.6 Activity mapping with occurrence graphs

Navigation between interactions across different timelines is possible, but this is limited to a maximum of four measurement categories, i.e. In BBFlashBack, users can select locations between video frame, mouse click, keystroke or voice interaction timelines. In ALFA method this is determined only by the research objectives, number of parallel timelines is unlimited. Data output formats are mostly suitable for publishing or presentation tasks, e.g. PowerPoint, Flash, movie etc. These tools cannot produce externally interpretable formats like XML, CVS etc.

5.7 UML modelling

Information available for UML modelling is limited to the screen recording outputs. Separation of the doctor-patient and doctor-computer interactions in an analysable format is not straightforward. Validation is also difficult due to unavailability of multiple camera angles.

Biomedical Informatics, Division of Community Health Sciences
Tel + 44 (0)20 8725 5661 Fax + 44 (0)20 8725 3584 Email slusigna@sgul.ac.uk
