# Supplementary material for: The ALFA (Activity Log Files Aggregation) Toolkit: A Method for Precise Observation of the Consultation
Source: J Med Internet Res. 2008 Sep 8;10(4):e27. doi: 10.2196/jmir.1080 (PMC2629369; doi:10.2196/jmir.1080)
Supplement: Supplementary file 5 [file jmir_v10i4e27_app5.pdf]

Log File Aggregation Tool

General Copyright statement

Log File Aggregation (LFA) tool V1.1

Copyright (C) 2008 Biomedical Informatics (BMI), St George's University of London. Citations to the toolkit should be as 'de Lusignan S, Kumarapeli P, Chan T, Pflug B, van Vlymen J, Jones B, Freeman GK. The ALFA (activity Log Files Aggregation) toolkit: a method for precise observation of the consultation. <J Med Internet Res 2008; ..>. method for precise observation of the consultation. *<J Med Internet Res 2008; ..>.*

This program is free software: you can redistribute it and/or modify it under the terms of the GNU General Public License as published by the Free Software Foundation, either version 3 of the License, or (at your option) any later version.

This program is distributed in the hope that it will be useful, but WITHOUT ANY WARRANTY; without even the implied warranty of MERCHANTABILITY or FITNESS FOR A PARTICULAR PURPOSE. See the GNU General Public License for more details.

You should have received a copy of the GNU General Public License along with this program. If not, see <http://www.gnu.org/licenses/>.

Any redistributions/public demonstration/publication/use of this software should acknowledge the original developers mentioned above as the Copyright holders. It is also encouraged to inform the original developers about such activities in order to assure the consistency among future software versions and to keep potential users up-to-date.

Source codes

Language: Sun Java 1.6
Platform: Microsoft Windows/Linux/Sun Solaris

[Ctrl + click to follow the following source code items]

Src1: LogAggregationTool.java
Src 2: AbstractGraphDisplay.java
Src 3: AggregationMediaPlayer.java
Src 4 : AggregationPanel.java
Src 5 : ConversionPanel.java
Src 6 : CSVLogConverter.java
Src 7 : CSVOutputConverter.java
Src 8 : DataEntry.java
Src 9 : DisplayPanel.java

Src 10 : errDialogEntry.java
Src 11: ErrorDialog.java
Src 12 : ErrorTableCellRenderer.java
Src 13 : EventEntry.java
Src 14 : GraphDisplayDialog.java
Src 15 : GUISuperclass.java
Src 16 : HeaderEntry.java
Src 17 : Histogram.java
Src 18: LogAggregator.java
Src 19 : LogConverter.java
Src 20 : newColumnGraphPanel.java
Src 21 : ObswinLogConverter.java
Src 22 : OutputConverter.java
Src 23 : OutputDeviceManager.java
Src 24 : OutputPanel.java
Src 25 : PRSLogConverter.java
Src 26 : ScreenImage.java
Src 27 : SDSOutputConverter.java
Src 28 : SearchDialog.java
Src 29 : SettingsPanel.java
Src 30: StartPanel.java
Src 31 : StepPanelInterface.java
Src 32 : TabularDisplayDialog.java
Src 33 : TabularDisplayDialogClosingAdapter.java
Src 34: UARLogConverter.java
Src 35 : UnionDialog.java
Src 36 : VideoSettings.java
Src 37 : WindowClosingAdapter.java
Src 38 : XMLConvertable.java
Src 39: XMLFactory.java
Src 40 : XMLLogConverter.java
Src 41: XMLOutputConverter.java

Src1: LogAggregationTool.java

/**
Main Java Class of Aggregation tool package.
Log File Aggregation (LFA) tool V1.1.

Copyright (C) 2008 Biomedical Informatics (BMI), St George's University of London. Citations to the toolkit should be as 'de Lusignan S, Kumarapeli P, Chan T, Pflug B, van Vlymen J, Jones B, Freeman GK. The ALFA (activity Log Files Aggregation) toolkit: a method for precise observation of the consultation. <J Med Internet Res 2008; ..>.

This program is free software: you can redistribute it and/or modify it under the terms of the GNU General Public License as published by the Free Software Foundation, either version 3 of the License, or (at your option) any later version.

```
This program is distributed in the hope that it will be useful, but WITHOUT ANY WARRANTY;
without even the implied warranty of MERCHANTABILITY or FITNESS FOR A PARTICULAR
PURPOSE.  See the GNU General Public License for more details.

You should have received a copy of the GNU General Public License along with this program.  If
not, see <http://www.gnu.org/licenses/>.

*/


package aggregationtool;

public class LogAggregationTool {

    //remembers the current directory after each time open/save dialog is called
    private static String globalFileDirectory;

    public static void main(String[] args) {

        //TODO set output device
        OutputDeviceManager.setCurrentDirForFileOutput();
        OutputDeviceManager.setOutputDevice(OutputDeviceManager.FILE_OUTPUT);

        //instance gui superclass for displaying main frame
        GUISuperclass tool_instance = new GUISuperclass();
        tool_instance.setVisible(true);
    }

    /**
     * This method returns an instance of the aggregation media player
     * in case the framework is available, otherwise null will be returned
     *
     * The reason to implement this method not in AggregationMediaPlayer itself
     * is because otherwise the compiler tries to load the classes before
     * reaching the method
     * So for any external usage of mediaplayer this method should be used,
     * and check of null value afterwards
     *
     * @return instance of aggMediaPlayer if available, null otherwise
     */
    public static AggregationMediaPlayer getAggregationMediaPlayer() {
        //check for JMF
        if (diagnostics()) {

            return AggregationMediaPlayer.getInstance();
        }
        else {
            return null;
        }
    }
```

Src 2: AbstractGraphDisplay.java

```
/**
This class defines the abstract layout for each graph based display dialog Main Java Class of
Aggregation tool package.
Log File Aggregation (LFA) tool V1.1.

Copyright (C) 2008 Biomedical Informatics (BMI), St George's University of London. Citations to
the toolkit should be as 'de Lusignan S, Kumarapeli P, Chan T, Pflug B, van Vlymen J, Jones B,
Freeman GK. The ALFA (activity Log Files Aggregation) toolkit: a method for precise observation
of the consultation. <J Med Internet Res 2008; ..>.

This program is free software: you can redistribute it and/or modify it under the terms of the
GNU General Public License as published by the Free Software Foundation, either version 3 of the
License, or (at your option) any later version.

This program is distributed in the hope that it will be useful, but WITHOUT ANY WARRANTY;
without even the implied warranty of MERCHANTABILITY or FITNESS FOR A PARTICULAR
PURPOSE.  See the GNU General Public License for more details.

You should have received a copy of the GNU General Public License along with this program.  If
not, see <http://www.gnu.org/licenses/>.

*/

package aggregationtool;

import java.awt.Dimension;

import java.util.ArrayList;

import javax.swing.JPanel;

/**
  * This is needed for the static method "get panel for type" to create a panel by calling the
 * constructor of the subclass depending on the type
 *  * also the getMinimumDisplaySize method must be implemented returning the minimum
 * dimension of the subpanel
 * This size is set in GraphDisplayDialog as minimum Size for the subpanel!
 * @author Bernhard Pflug
 */
public abstract class AbstractGraphDisplay extends JPanel {

    //static for types of subclasses
    //add identifier in case of new subclass
    public static int COLUMN = 0;
    public static int HISTOGRAM = 1;

    protected GraphDisplayDialog gdd;
    /**
     * This constructor takes the GraphDisplayDialog to event if window is changed
     */
```

```
    public AbstractGraphDisplay(GraphDisplayDialog gdd) {
        this.gdd = gdd;
    }

    /**
     * This method must be implemented in each subclass to define the minimum size of the
window
     * @return Dimension with minimum X- and Y- Coordinate
     */
    public abstract Dimension getMinimumDisplaySize();

    /**
     * Must be called each time if window is repainted
     * for example if properties for display are changed
     */
    public void windowChanged() {
        gdd.windowChanged(this);
    }


    /**
     * This function creates the Panel depending on choosen DisplayStyle for the given data
source
     * @param FormatType Format of the style for output - not used now because GraphPanels
doesn't depend on format
     * @param DisplayStyle, types defined above
     * @param Source with dataEntries
     * @return Panel with subclass of AbstractGraphDisplay containing information of Source
     */
    public static AbstractGraphDisplay getPanelForType(GraphDisplayDialog gdd,String
FormatType, int DisplayStyle, ArrayList Source) {

        //column display
        if (DisplayStyle == AbstractGraphDisplay.COLUMN) {
            return new newColumnGraphPanel(gdd,Source);
        }
        //histogram display
        else if (DisplayStyle == AbstractGraphDisplay.HISTOGRAM) {
            return new Histogram(gdd,Source);
        }
        //unknown type
        else {
            System.out.println("AbstractGraphDisplay:getPanelForType: Unknown DisplayStyle
given");
            return null;
        }
    }

    //*************************************************************************
    //**************** method to create media player *************************
    //*************************************************************************

    /**
```

```
    * Method to add times to intitial video settings of settingspanel and
    * create/show aggregation media player for selected times
    * @param initialSettings from settings step
    * @param eventEntry to be displayed
    */
    public static void showMediaPlayer(VideoSettings initialSettings,EventEntry eventEntry) {
        //check whether settings were created in settings step
        if (initialSettings != null) {

            //check whether settings ok
            if(initialSettings.settingsOK()) {

                //set start- and endtime of eventEntry
                if (eventEntry.isDuration()) {
                    initialSettings.setStartEndTimes(eventEntry.StartTime,eventEntry.EndTime);
                }
                else {

initialSettings.setStartEndTimes(eventEntry.StartTime,VideoSettings.NO_CERTAIN_ENDTIME);
                }

                //check whether set start- and endtime are ok
                if (initialSettings.settingsOK()) {
                    AggregationMediaPlayer player =
LogAggregationTool.getAggregationMediaPlayer();

                    if (player != null) {
                        player.setProperties(initialSettings);

                        //In case event is duration, start playing, otherwise only show sceen
                        if (eventEntry.isDuration()) {
                            player.play();
                        }
                    }
                    else {

                        ErrorDialog errDialog = new ErrorDialog(null,"Error opening
MediaPlayer...",true);
                        errDialog.addMessage("Unable to open player!",ErrorDialog.ERROR);
                        errDialog.showErrDialog();
                    }
                }
            }
            else {
                ErrorDialog errDialog = new ErrorDialog(null,"Error using settings...",true);
                errDialog.addMessage("at least one setting doesn't consist requirements, please
redefine settings",ErrorDialog.ERROR);
                errDialog.showErrDialog();
            }
        }
        else {
            ErrorDialog errDialog = new ErrorDialog(null,"Error opening Media Player...",true);
```

```
        errDialog.addMessage("set video settings in settings step to be able to link to
video",ErrorDialog.ERROR);
        errDialog.showErrDialog();
      }
    }
}
```

Src 3: AggregationMediaPlayer.java

```
/**
JFrame containing the mediaplayer, which can show any supported media type
Log File Aggregation (LFA) tool V1.1.

Copyright (C) 2008 Biomedical Informatics (BMI), St George's University of London. Citations to
the toolkit should be as 'de Lusignan S, Kumarapeli P, Chan T, Pflug B, van Vlymen J, Jones B,
Freeman GK. The ALFA (activity Log Files Aggregation) toolkit: a method for precise observation
of the consultation. <J Med Internet Res 2008; ..>.

This program is free software: you can redistribute it and/or modify it under the terms of the
GNU General Public License as published by the Free Software Foundation, either version 3 of the
License, or (at your option) any later version.

This program is distributed in the hope that it will be useful, but WITHOUT ANY WARRANTY;
without even the implied warranty of MERCHANTABILITY or FITNESS FOR A PARTICULAR
PURPOSE.  See the GNU General Public License for more details.

You should have received a copy of the GNU General Public License along with this program.  If
not, see <http://www.gnu.org/licenses/>.

*/
package aggregationtool;

import java.awt.BorderLayout;
import java.awt.Component;

import java.awt.Dimension;
import java.awt.event.WindowAdapter;

import java.awt.event.WindowEvent;

import java.io.IOException;

import java.net.MalformedURLException;
import java.net.URL;

import javax.media.ControllerEvent;
import javax.media.ControllerListener;
import javax.media.EndOfMediaEvent;
import javax.media.Manager;
import javax.media.NoPlayerException;
```

```
import javax.media.Player;

import javax.media.PrefetchCompleteEvent;
import javax.media.RealizeCompleteEvent;
import javax.media.Time;

import javax.swing.JFrame;
import javax.swing.JOptionPane;


/**
* @author Bernhard Pflug
 */
public class AggregationMediaPlayer extends JFrame
implements ControllerListener{

    //******************************
    //***** video settings *********
    //******************************
    Player mplayer;
    VideoSettings videoSettings;

    Component visual = null;
    Component control = null;

    /*************************************************************************************
    //******************************** constructor  ************************************
    //**********************************************************************************/

    /**
     * call superclass with settings for Dialog
     */
    private AggregationMediaPlayer() {
        super("Aggregation media player");
    }

    /*************************************************************************************
    //************************ methods for interaction *******************************
    //**********************************************************************************/
    /**
     * starts playing the media file
     */
    public void play() {
        //check settings
        if (videoSettings.settingsOK()) {
            //in case of correct settings, start playing
            mplayer.start();
        }
    }

    /**
     * stops playing the media file
     */
```

```
public void stop() {
    //check settings
    if (videoSettings.settingsOK()) {
        //stop player
        mplayer.stop();
    }
}

/***************************************************************************
//********************** method for videosettings *************************
//************************************************************************/

/**
 * Sets the property file for the video player
 * Every time calling this method a new player with the VideoSettings is created
 * and the player frame is created
 * Depending on the video this method can take some time
 * @param vs contains all properties for the video player
 */
public void setProperties(VideoSettings vs) {

    videoSettings = vs;

    try {

        URL url = new URL("file:"+videoSettings.getFilePath());

        if (url == null) {
            Fatal("Can't build URL for " + videoSettings.getFilePath());
            videoSettings.setSettingsOK(false);
            return;
        }

        try {
            // Create an instance of a player for this media
            mplayer = Manager.createPlayer(url);

            //remove older components
            getContentPane().removeAll();

            //create layout
            getContentPane().setLayout( new BorderLayout() );

            //add Listener for control properties of player
            mplayer.addControllerListener((ControllerListener) this);

            mplayer.realize();

            //add listener for closing windwo
            this.addWindowListener(new WindowAdapter () {
                    public void windowClosing(WindowEvent event) {
                        mplayer.stop();
                        mplayer.close();
```

```
                }
            }
        );

        //set Window visible
        this.setVisible(true);
    }
    catch (NoPlayerException e) {
        videoSettings.setSettingsOK(false);
        Fatal("Error: " + e.getMessage());
    }
}
catch (MalformedURLException ex) {
    videoSettings.setSettingsOK(false);
    Fatal("Error:" + ex.getMessage());
}
catch (IOException ex) {
    videoSettings.setSettingsOK(false);
    Fatal("Error:" + ex.getMessage());
}
}
/****************************************************************************
//******************** method for controller listener *********************
//*************************************************************************/

 public void controllerUpdate(ControllerEvent ce) {
    if (ce instanceof RealizeCompleteEvent) {
        mplayer.prefetch();
    } else if (ce instanceof PrefetchCompleteEvent) {
        if (visual != null)
            return;

        //check for visual component
        if ((visual = mplayer.getVisualComponent()) != null) {
           //set size
            Dimension size = visual.getPreferredSize();
            videoSettings.videoWidth = size.width;
            videoSettings.videoHeight = size.height;
            getContentPane().add("Center", visual);
        } else {
            videoSettings.videoWidth = 320;
        }

        //check for control component
        if ((control = mplayer.getControlPanelComponent()) != null) {
            videoSettings.controlHeight = control.getPreferredSize().height;
            getContentPane().add("South", control);
        }

        setSize(videoSettings.videoWidth + videoSettings.insetWidth,
                videoSettings.videoHeight + videoSettings.controlHeight +
videoSettings.insetHeight);
```

```
            //repaint method for components
            validate();

            //************ setting all properties wich need a realized player *************

            //setting start- and endtime in case they are set and player is prefetched
            if (videoSettings.startTimeSet()) {
               //check if starttime higher as video duration
               if(videoSettings.getStartTime() > mplayer.getDuration().getSeconds()) {
                  JOptionPane.showMessageDialog(this,"selected start-time is higher than duration
of video!\nStart-time set to default!","start time",JOptionPane.WARNING_MESSAGE);
               } else {
                  mplayer.setMediaTime(new Time(videoSettings.getStartTime()));
               }
            }

            if (videoSettings.endTimeSet()) {

               //check if endtime higher as video duration
               if(videoSettings.getEndTime() > mplayer.getDuration().getSeconds()) {
                  JOptionPane.showMessageDialog(this,"selected end-time is higher than duration of
video!\nEnd-time set to default","end time",JOptionPane.WARNING_MESSAGE);
               } else {
                  mplayer.setStopTime(new Time(videoSettings.getEndTime()));
               }

            }

            //set reate for player
            mplayer.setRate(videoSettings.getVideoRate());

        } else if (ce instanceof EndOfMediaEvent) {
            //TODO in case any action should be performed in case of media end define here
        }
    }

    /****************************************************************************
    //*************************** JMF error method *****************************
    //**************************************************************************/

    /**
     * creates an error output message
     */
    static void Fatal(String s) {
        JOptionPane.showMessageDialog(null,s,"Media-Player
error",JOptionPane.ERROR_MESSAGE);
    }

    /****************************************************************************
    //************************** instance method ******************************
    //**************************************************************************/
    /**
     * Method to get instance of MediaPlayer
```

```
    * SHOULD ONLY BE USED BY LOGAGGREGATIONTOOL TO GET INSTANCE
    * TO GET INSTANCE FOR USAGE CALL FUNCTIONALTIY IN LOGAGGREGATIONTOOL
    * AS IT SUPPORTS JMF DIAGNOSIS
    * @return returns an new instance of the mediaplayer
    */
    @Deprecated
    public static AggregationMediaPlayer getInstance() {

        return new AggregationMediaPlayer();
    }
}
```

Src 4 : AggregationPanel.java

```
/**
Panel for the aggregation option in the aggregation tool workflow
Log File Aggregation (LFA) tool V1.1.
Copyright (C) 2008 Biomedical Informatics (BMI), St George's University of London. Citations to
the toolkit should be as 'de Lusignan S, Kumarapeli P, Chan T, Pflug B, van Vlymen J, Jones B,
Freeman GK. The ALFA (activity Log Files Aggregation) toolkit: a method for precise observation
of the consultation. <J Med Internet Res 2008; ..>.

This program is free software: you can redistribute it and/or modify it under the terms of the
GNU General Public License as published by the Free Software Foundation, either version 3 of the
License, or (at your option) any later version.

This program is distributed in the hope that it will be useful, but WITHOUT ANY WARRANTY;
without even the implied warranty of MERCHANTABILITY or FITNESS FOR A PARTICULAR
PURPOSE.  See the GNU General Public License for more details.

You should have received a copy of the GNU General Public License along with this program.  If
not, see <http://www.gnu.org/licenses/>.

*/

package aggregationtool;

import java.awt.Rectangle;
import java.awt.event.ActionEvent;
import java.awt.event.ActionListener;

import java.awt.event.KeyEvent;

import java.util.ArrayList;

import javax.swing.JButton;
import javax.swing.JCheckBox;
import javax.swing.JLabel;
import javax.swing.JPanel;

/**
```

```
* @author Bernhard Pflug
 */

public class AggregationPanel extends JPanel
implements StepPanelInterface{

    //Reference to superclass
    GUISuperclass superclass;

    //Buttons for navigation
    private JButton next_button = new JButton();
    private JButton prev_button = new JButton();

    //Aggregated Output
    public ArrayList AggregationFile;

    //GUI Elements
    private JLabel jLabel1 = new JLabel();

    private JLabel [] NameLabels;
    private JCheckBox [] CheckBoxes;

    public AggregationPanel(GUISuperclass superclass) {

        this.superclass= superclass;

        try {
            jbInit();
        } catch (Exception e) {
            e.printStackTrace();
        }
    }

    /**
     * Creates GUI depending on the Entries to display
     * @throws Exception
     */
    private void jbInit() throws Exception {
        this.setLayout( null );

        jLabel1.setText("Please select all files you want to aggregate");
        jLabel1.setBounds(new Rectangle(5, 5, 340, 30));

        //For each converted File display one row with checkbutton to aggregate
        ArrayList commonFiles = superclass.conversionpanel.CommonFiles;

        NameLabels = new JLabel[commonFiles.size()];
        CheckBoxes = new JCheckBox[commonFiles.size()];

        for (int i=0; i<commonFiles.size(); i++) {
            //For each row create JLabel with Filename
            //Attention! First entry in list must be HeaderInformation
```

```
            NameLabels[i] = new JLabel("Entry "+(i+1)+": 
"+((HeaderEntry)((ArrayList)commonFiles.get(i)).get(0)).fileName);
            NameLabels[i].setBounds(80,50+i*30,400,20);
            this.add(NameLabels[i]);

            //For each row create checkbox to select
            CheckBoxes[i] = new JCheckBox("",true);
            CheckBoxes[i].setBounds(550,50+i*30,30,20);
            this.add(CheckBoxes[i]);
        }

        //next_button
        next_button.setText("next");
         next_button.setBounds(new Rectangle(550, 380, 80, 30));
         next_button.setMnemonic(KeyEvent.VK_N);
         next_button.setToolTipText("next step");
         next_button.addActionListener(new ActionListener() {
                public void actionPerformed(ActionEvent e) {

                    process_aggregation();
                }
        });

        //prev_button
         prev_button.setText("prev");
         prev_button.setBounds(460,380,80,30);
         prev_button.setMnemonic(KeyEvent.VK_P);
         prev_button.setToolTipText("previous step");
         prev_button.addActionListener(new ActionListener() {
                public void actionPerformed(ActionEvent e) {
                    display_previous();
                }
        });

        //Add to ContentPane
        this.add(jLabel1, null);
        this.add(next_button, null);
        this.add(prev_button,null);
    }

    /**
     * method to process aggregation when clicking next button
     */
    private void process_aggregation() {
        ArrayList aggProcess = new ArrayList();
        this.AggregationFile = new ArrayList();

        //create new List with all files which are selected for aggregation
        for (int i=0; i< superclass.conversionpanel.CommonFiles.size(); i++) {
            //if Checkbox for file is selected, add to aggProcess
            if (CheckBoxes[i].isSelected()) {
                aggProcess.add(superclass.conversionpanel.CommonFiles.get(i));
            }
```

```
        }

        if (!aggProcess.isEmpty()) {
            //in case at least one entry is selected, instance LogAggregator and let it process
aggregation
            LogAggregator logAgg = new LogAggregator(aggProcess);
            AggregationFile = logAgg.process_aggregation();

            //after processing display next panel
            display_next();
        }
        else {
            ErrorDialog errDialog = new ErrorDialog(superclass,"Error at aggregation",true);
            errDialog.addMessage("at least one entry must be selected",ErrorDialog.ERROR);
            errDialog.showErrDialog();
        }
    }

    /**
     * Display next step
     */
    public void display_next() {
        this.setVisible(false);
        superclass.displaypanel= new DisplayPanel(superclass);
        superclass.displayPanel(superclass.displaypanel,5);
    }

    /**
     * Go back to previous step
     */
    public void display_previous(){
        this.setVisible(false);
        superclass.displayPanel(superclass.conversionpanel,3);
    }
}
```

Src 5 : ConversionPanel.java

/**
Panel for the conversion step
Log File Aggregation (LFA) tool V1.1.

Copyright (C) 2008 Biomedical Informatics (BMI), St George's University of London. Citations to the toolkit should be as 'de Lusignan S, Kumarapeli P, Chan T, Pflug B, van Vlymen J, Jones B, Freeman GK. The ALFA (activity Log Files Aggregation) toolkit: a method for precise observation of the consultation. <J Med Internet Res 2008; ..>.

This program is free software: you can redistribute it and/or modify it under the terms of the GNU General Public License as published by the Free Software Foundation, either version 3 of the License, or (at your option) any later version.

This program is distributed in the hope that it will be useful, but WITHOUT ANY WARRANTY; without even the implied warranty of MERCHANTABILITY or FITNESS FOR A PARTICULAR PURPOSE. See the GNU General Public License for more details.

You should have received a copy of the GNU General Public License along with this program. If not, see <http://www.gnu.org/licenses/>.

```
*/

package aggregationtool;

import java.awt.Font;
import java.awt.Rectangle;
import java.awt.event.ActionEvent;
import java.awt.event.ActionListener;

import java.awt.event.KeyEvent;

import java.io.File;

import java.util.ArrayList;

import javax.swing.BorderFactory;
import javax.swing.JButton;
import javax.swing.JComboBox;
import javax.swing.JDialog;
import javax.swing.JFileChooser;
import javax.swing.JLabel;
import javax.swing.JOptionPane;
import javax.swing.JPanel;
import javax.swing.JTextField;
import javax.swing.SwingConstants;
import javax.swing.border.TitledBorder;

public class ConversionPanel extends JPanel
implements StepPanelInterface, ActionListener{

    //instance of supercalss
    private GUISuperclass superclass;

    //number of rows to be displayed to read in files
    private int rowsToShow;

    //GUI elements
    private JComboBox[] types;
    private JTextField[] fields;
    private JButton[] bButtons;
    private JButton[] opButtons;

    private LogConverter [] Converters;
    //contains all Arraylists created by conversion step
    public ArrayList CommonFiles;
```

```
//GUI elements
private JButton next_button = new JButton();
private JButton prev_button = new JButton();
private JLabel jLabel1 = new JLabel();
private JLabel jLabel2 = new JLabel();

//**************************************************************************
//**************************** constructor **********************************
//**************************************************************************

public ConversionPanel(GUISuperclass superclass) {
    this.superclass=superclass;

    //Get information of the number of files from SettingsPanel
    rowsToShow = superclass.settingspanel.getNumberOfFiles();

    //init converters list
    Converters = new LogConverter[rowsToShow];

    //If there is only one row, disable aggregation Step
    if (rowsToShow ==1) {
        superclass.setAggregationEnabled(false);
    }
    else {
        superclass.setAggregationEnabled(true);
    }

    try {
        jbInit();
    } catch (Exception e) {
        e.printStackTrace();
    }
}

//**************************************************************************
//***************************** GUI METHOD **********************************
//**************************************************************************

/**
 * Initialises the layout of the panel
 * @throws Exception
 */
private void jbInit() throws Exception {
    this.setLayout( null );

     //next_button
     next_button.setText("next");
     next_button.setBounds(new Rectangle(550, 380, 80, 30));
     next_button.setMnemonic(KeyEvent.VK_N);
     next_button.setToolTipText("next step");
     next_button.addActionListener(new ActionListener() {
                public void actionPerformed(ActionEvent e) {
```

```
                process_conversion();
            }
    });

    //prev_button
     prev_button.setText("prev");
     prev_button.setBounds(460,380,80,30);
     prev_button.setMnemonic(KeyEvent.VK_P);
     prev_button.setToolTipText("previous step");
     prev_button.addActionListener(new ActionListener() {
            public void actionPerformed(ActionEvent e) {
                display_previous();
            }
    });

    //Labels to display
    jLabel1.setText("Type");
    jLabel1.setBounds(new Rectangle(25, 20, 65, 20));
    jLabel1.setFont(new Font("Times New Roman", 1, 14));
    jLabel1.setHorizontalAlignment(SwingConstants.CENTER);
    jLabel2.setText("Path of the File");
    jLabel2.setBounds(new Rectangle(130, 25, 305, 20));
    jLabel2.setFont(new Font("Times New Roman", 1, 14));
    jLabel2.setHorizontalAlignment(SwingConstants.CENTER);

    //create for each File to select an type combobox, a textfield and a browse_button
    types = new JComboBox[rowsToShow];
    fields = new JTextField[rowsToShow];
    bButtons = new JButton[rowsToShow];
    opButtons = new JButton[rowsToShow];

    for (int i =0; i <rowsToShow; i++) {

        //create Typ ComboBox
        types[i]= new JComboBox();
        types[i].addActionListener(this);

        types[i].setBounds(5,50+i*30,105,20);

        types[i].addItem(LogConverter.OBSWINEVENTTYPE);
        types[i].addItem(LogConverter.UAREVENTTYPE);
        types[i].addItem(LogConverter.PRSEVENTTYPE);
        types[i].addItem(LogConverter.XMLFILETYPE);
        types[i].addItem(LogConverter.CSVFILETYPE);

        setConverter(types[i]);

        this.add(types[i]);

        //create JTextField
        fields[i]= new JTextField();
        fields[i].setBounds(120,50+i*30,320,20);
        this.add(fields[i]);
```

```
            //create browse_button
            bButtons[i]= new JButton("Browse...");
            bButtons[i].setBounds(450,50+i*30,90,20);
            bButtons[i].setToolTipText("Browse to select file");
            bButtons[i].addActionListener(new ActionListener() {
                    public void actionPerformed(ActionEvent e) {

                        JFileChooser fc = new JFileChooser();

                        //if global file directory set, init with remembered dir
                        if (LogAggregationTool.isFileDirSet()) {
                            fc.setCurrentDirectory(new File(LogAggregationTool.getFileDir()));
                        }

                        int returnVal = fc.showOpenDialog(superclass);

                        if (returnVal == JFileChooser.APPROVE_OPTION) {
                            //calculate button number
                            //Attention! if canging the bounds of the rows, also change the calculation!!
                            //TODO dirty, change algorithm to get line number
                            int number = (int)(((JButton)e.getSource()).getBounds().getY()-50)/30;
                            fields[number].setText(fc.getSelectedFile().getPath());
                        }

                        //set global file dir
                        LogAggregationTool.setFileDir(fc.getCurrentDirectory().getPath());
                    }
                }
            );
            this.add(bButtons[i]);

            opButtons[i] = new JButton("Options...");
            opButtons[i].setBounds(550,50+i*30,90,20);
            opButtons[i].setToolTipText("edit options for choosen type");
            opButtons[i].addActionListener(new ActionListener() {
                public void actionPerformed(ActionEvent e) {
                    OptionDialog opDialog = new OptionDialog();
                    int number = (int)(((JButton)e.getSource()).getBounds().getY()-50)/30;
                    //TODO dirty, change algorithm to get line number
                    opDialog.setLogConverter(Converters[number]);
                    opDialog.showOptionDialog();
                }
            });
            this.add(opButtons[i]);
        }

        //add all components to frame
        this.add(jLabel2, null);
        this.add(jLabel1, null);
        this.add(next_button);
        this.add(prev_button);
    }
```

```
//**************************************************************************
//************************ processing methods ******************************
//**************************************************************************

/**
 * Method to set Converter for selected type in comboBox
 */
private void setConverter(JComboBox typeBox) {

    //calculate button number
    //Attention! if canging the bounds of the rows, also change the calculation!!
    //TODO dirty, change algorithm to get line number
    int lineNumber = (int)(typeBox.getBounds().getY()-50)/30;

    //Depending on selected type create LogConverter
    if (typeBox.getSelectedItem().equals(LogConverter.OBSWINEVENTTYPE)) {
        Converters[lineNumber] = new ObswinLogConverter();
    }
    if (typeBox.getSelectedItem().equals(LogConverter.UAREVENTTYPE)) {
        Converters[lineNumber] = new UARLogConverter();
    }
    if (typeBox.getSelectedItem().equals(LogConverter.PRSEVENTTYPE)) {
        Converters[lineNumber] = new PRSLogConverter();
    }
    if (typeBox.getSelectedItem().equals(LogConverter.XMLFILETYPE)) {
        Converters[lineNumber] = new XMLLogConverter();
    }
    if (typeBox.getSelectedItem().equals(LogConverter.CSVFILETYPE)) {
        Converters[lineNumber] = new CSVLogConverter();
    }

}

/**
 * Method called for each registrated component
 * All type comboboxes added to create instance of type depending on selection
 * @param e
 */
public void actionPerformed (ActionEvent e) {

    //Check for source type combo box
    if (e.getSource() instanceof JComboBox) {

        //change converter to new selected type
        setConverter((JComboBox)e.getSource());
    }
}

/**
 * This function converts each file to the common format depending on the type
 * In case of adding new LogConverter Subclass it must also be added here
 */
```

```
    public void process_conversion() {
        CommonFiles = new ArrayList();

        ErrorDialog errDialog = new ErrorDialog(superclass,"output from conversion",true);

        //For each row
        for (int i=0; i< this.rowsToShow; i++) {

            //get converter for line
            LogConverter con= Converters[i];

            //set file path for converter
            con.setFilePath(fields[i].getText());

            //Call method to create converson for each entry
            ArrayList commonContent = con.getDataFromFile();

            //Add all warnings to errorDialog
            errDialog.addMessage("Data source "+(i+1)+":",ErrorDialog.INFO);
            errDialog.addAllMsg(con.getErrorWarnings());

            //add produced common content to ArrayList
            CommonFiles.add(commonContent);
        }

        //if error list is entry, no errors and warnings occurred
        if (errDialog.containsOnlyInfos()) {
            display_next();
        }
        //if error list includes no errors, ask if he want to ignore warnings
        else if (!errDialog.containsErrors()) {
            //show dialog

            errDialog.showErrDialog();

            //Ask if user want to ignore warnings
            int ret = JOptionPane.showConfirmDialog(superclass,"Do you want to ignore these
warnings and continue?","Continue",JOptionPane.YES_NO_OPTION);

            if (ret == JOptionPane.YES_OPTION) {
                display_next();
            }
        }
        //Otherwise show errors
        else {
            errDialog.showErrDialog();
        }
    }

    //**************************************************************************
    //***************************** Navigation ********************************
    //**************************************************************************
```

```
    public void display_next() {
        this.setVisible(false);

        if (superclass.isAggregationEnabled()) {
            superclass.aggregationpanel= new AggregationPanel(superclass);
            superclass.displayPanel(superclass.aggregationpanel,4);
        }
        else {
            superclass.displaypanel = new DisplayPanel(superclass);
            superclass.displayPanel(superclass.displaypanel,5);
        }
        //superclass.displayPanel(superclass.conversionpanel,3);
    }

    public void display_previous(){
        this.setVisible(false);
        superclass.displayPanel(superclass.settingspanel,2);
    }

    //***************************************************************************
    //*************************** Option Dialog ********************************
    //***************************************************************************

    /**
     * Inner class to display option dialog for each selected input type in one row
     *
     * @author Bernhard Pflug
     */
    class OptionDialog extends JDialog {

        private LogConverter logConverter;

        //***** constructor *****
        public OptionDialog () {
            super(superclass,"options for...",true);
        }

        //***** set log converter ****
        public void setLogConverter(LogConverter logConverter) {
            this.logConverter = logConverter;
        }

        //***** show dialog *****
        public void showOptionDialog() {

            this.setLayout (null);

            //create header
            JLabel headerLabel = new JLabel("options for
"+logConverter.getClass().getSimpleName());
            headerLabel.setBounds(10,5,LogConverter.PROPERTY_PANEL_WIDTH,20);
            headerLabel.setAlignmentX(JLabel.CENTER_ALIGNMENT);
            this.add(headerLabel);
```

```
            // create content
            JPanel optionPanel = logConverter.getPropertyPanel();
            TitledBorder titledBorder = BorderFactory.createTitledBorder("options");
            titledBorder.setTitleJustification(TitledBorder.CENTER);
            optionPanel.setBorder(titledBorder);
            optionPanel.setLocation(0,30);

            this.add(optionPanel);

            // close button
            JButton closeButton = new JButton("CLOSE");
            closeButton.setFont(new Font("null",Font.BOLD,11));
            closeButton.setBounds(LogConverter.PROPERTY_PANEL_WIDTH/2-
35,LogConverter.PROPERTY_PANEL_HEIGHT+40,70,20);
            closeButton.addActionListener(new ActionListener () {
               public void actionPerformed(ActionEvent e) {
                  setVisible(false);
               }
            });
            this.add(closeButton);

            // set size

this.setSize(LogConverter.PROPERTY_PANEL_WIDTH,LogConverter.PROPERTY_PANEL_HEIGHT+
100);

            //set visible
            this.setVisible(true);
        }
    }
}
```

Src 6 : CSVLogConverter.java

/**
Subclass of log converter to process CSV filesMain Java Class of Aggregation tool package.
Log File Aggregation (LFA) tool V1.1.

Copyright (C) 2008 Biomedical Informatics (BMI), St George's University of London. Citations to the toolkit should be as 'de Lusignan S, Kumarapeli P, Chan T, Pflug B, van Vlymen J, Jones B, Freeman GK. The ALFA (activity Log Files Aggregation) toolkit: a method for precise observation of the consultation. <J Med Internet Res 2008; ..>.

This program is free software: you can redistribute it and/or modify it under the terms of the GNU General Public License as published by the Free Software Foundation, either version 3 of the License, or (at your option) any later version.

This program is distributed in the hope that it will be useful, but WITHOUT ANY WARRANTY; without even the implied warranty of MERCHANTABILITY or FITNESS FOR A PARTICULAR PURPOSE. See the GNU General Public License for more details.

You should have received a copy of the GNU General Public License along with this program. If not, see <http://www.gnu.org/licenses/>.

```
*/

package aggregationtool;

import java.awt.event.ActionEvent;
import java.awt.event.ActionListener;

import java.util.ArrayList;
import java.util.Date;
import java.util.NoSuchElementException;
import java.util.StringTokenizer;

import javax.swing.JComboBox;
import javax.swing.JLabel;
import javax.swing.JPanel;


public class CSVLogConverter extends LogConverter {

   //****** properties ******

    //selectedable seperators
    private Character [] seperators = {';',',',':'};
    private int selectedSepIndex = 1;

    //****** settings panel *******
    private JComboBox sepCombo = new JComboBox(seperators);

   //**************************************************************************
   //************************** constructor ***********************************
   //**************************************************************************

   public CSVLogConverter () {

   }

   public CSVLogConverter(String filePath) {
      this.FilePath = filePath;
   }

   //**************************************************************************
   //********************** property panel method *****************************
   //**************************************************************************

    /**
     * Method to create properties for specific options on format
     * Should instance property Panel of super class, add all necessary components
     * and read them before processing to get required options
     * In case no property panel is needed leave body without content
```

```
     */
    public void createPropertyPanel() {

        //TODO In case options are necessary uncomment following lines, add components
        //and read them before processing

          //*** Panel ***
          propertyPanel = new JPanel();

          propertyPanel.setLayout( null );

          //Threshold label
          JLabel sepLabel = new JLabel("Choose seperator CSV file is seperated with");
          sepLabel.setBounds(20,30,350,20);

          //Threshold combo box
          sepCombo.setBounds(20,60,100,20);
          sepCombo.setSelectedIndex(selectedSepIndex);
          sepCombo.addActionListener(new ActionListener() {

              public void actionPerformed(ActionEvent e) {
                 //set slectedThresIndex as new selected one in combo box
                 selectedSepIndex = sepCombo.getSelectedIndex();
              }
           });

          //add to panel
          propertyPanel.add(sepLabel);
          propertyPanel.add(sepCombo);
    }

   //**************************************************************************
   //************************* process method *********************************
   //**************************************************************************

   /**
    * implemented method of superclass to interpret content of the read input file string
    *
    * !! time unit of input must fit global time unit of program !!
    * !! Further more first line must include header information, and from second line on data will
be interpreted !!
    */
   public void interpretLogFile() {

       //ArrayList containing the common Context of the file
       CommonContent = new ArrayList();

       //************************* process header ****************************

       //create header entry
       CommonContent.add(new HeaderEntry(LogConverter.GLOBAL_TIMEUNIT,new
Date(this.LastModified),this.FileName));
```

```
        //check only if header information fits data structure, otherwise create warning
        //get internal elements of EventEntry
        Object [] itemNames = EventEntry.complexItemNames();
        String requiredHeaderString="";

        //add all of them to string seperated by selected sep
        for (int i=0; i < itemNames.length-1; i++) {
           requiredHeaderString += (itemNames[i].toString() + seperators[selectedSepIndex]);
        }

        if (itemNames.length >0) {
           requiredHeaderString += itemNames[itemNames.length-1];
        }
        else {
           addErrorWarning(ErrorDialog.ERROR,"Internal structure of EventEntry contains no
elements");
           return;
        }

        //compare string with first line of file
        if (!FileContent.isEmpty() && FileContent.get(0).toString().length() >0) {

           if (!requiredHeaderString.equals(FileContent.get(0))) {
              addErrorWarning(ErrorDialog.WARNING,"header info in file doesn't fit internal
structure, maybe wrong seperator choosen!");
           }

           //after checking header, remove first line
           FileContent.remove(0);
        }
        else {
           addErrorWarning(ErrorDialog.ERROR,"Error reading first line in file, first line must
contain header information");
           return;
        }
        //************************* process content ****************************

        //go through all entries
        for (int i=0; i < FileContent.size(); i++) {
           try {

              //event entry to be created
              EventEntry eventEntry = new EventEntry();

              StringTokenizer st = new
StringTokenizer((String)FileContent.get(i),""+seperators[selectedSepIndex]);

              //counts for while look to detect item
              int entryCounter =0;

              while (st.hasMoreTokens()) {

                 String valueString = st.nextToken();
```

```
/**!!!! This part now depends on internal structure of EventEntry!!! */


//**** starttime ****
if (entryCounter == 0) {
    //check if string contains information
    if (valueString.length() > 0) {

        //set start time - in case no number NumberFormatException is throughn
        eventEntry.setStartTime(Float.parseFloat(valueString));
    }
    else {
        addErrorWarning(ErrorDialog.WARNING,"Unable to create starttime of entry
in line "+i+", set to zero");
        eventEntry.setStartTime(0);
    }
}
//***** endtime *****
else if (entryCounter == 1) {
    //check if string contains information
    if (valueString.length() > 0) {

        //set end time - in case no number NumberFormatException is throughn
        eventEntry.setEndTime(Float.parseFloat(valueString));
    }
    else {
        addErrorWarning(ErrorDialog.WARNING,"Unable to create endtime of entry in
line "+i+", set to zero");
        eventEntry.setEndTime(0);
    }
}
//***** Event type ******
else if (entryCounter == 2) {
    //check if string contains infromation
    if (valueString.length() > 0) {


        //check for event type one of handled eventtypes
        if (!valueString.equals(LogConverter.OBSWINEVENTTYPE) &&
!valueString.equals(LogConverter.PRSEVENTTYPE) &&
!valueString.equals(LogConverter.UAREVENTTYPE)) {
            addErrorWarning(ErrorDialog.INFO,"- LINE "+i);
            addErrorWarning(ErrorDialog.ERROR,"- - Event type is not of type
'"+LogConverter.OBSWINEVENTTYPE+"' or '"+LogConverter.PRSEVENTTYPE+"' or
'"+LogConverter.UAREVENTTYPE+"'");
            addErrorWarning(ErrorDialog.ERROR,"- - Change event type excatly to
one of the values above as other spellings are not supported!");
        }

        //set event type of event
        eventEntry.getEvent().setType(valueString);
```

```
                }
                else {
                    addErrorWarning(ErrorDialog.WARNING,"Unable to create event type of entry
in line "+i+", set to dummy string");
                    eventEntry.getEvent().setType("<unkown type>");
                }
            }
            //***** event value *****
            else if (entryCounter == 3) {
                //check if string contains infromation
                if (valueString.length() > 0) {

                    eventEntry.getEvent().setValue(valueString);
                }
                else {
                    addErrorWarning(ErrorDialog.WARNING,"Unable to create event value of
entry in line "+i+", set to dummy string");
                    eventEntry.getEvent().setValue("<unkown value>");
                }
            }
            else {
                addErrorWarning(ErrorDialog.WARNING,"Line "+i+" contains more entries as
needed for internal structure!");
                break;
            }

            entryCounter++;
        }

        //check if all items where read
        if (entryCounter == 4) {

            //correct number of entries read, add to common content
            CommonContent.add(eventEntry);
        }
        else {
            //if entryCounter is not 3 line doesn't contain information for all needed information
of EventEntry
            addErrorWarning(ErrorDialog.WARNING,"Entry in line "+i+" contains not enough
information to create EventEntry, line ignored");
        }
    }
    catch (NoSuchElementException ex) {
        //should normaly not occur as while loop only loops while existing items

        //Exception will be throughn in case StringTokenizer wants to access unaavailable
item
        addErrorWarning(ErrorDialog.WARNING,"Entry in line "+i+" contains not enough
information to create EventEntry, line ignored");
    }
    catch (NumberFormatException ex2) {
```

```
            addErrorWarning(ErrorDialog.WARNING,"Unable to get either start time or end time
of entry in line "+i+", line ignored");
         }
      }
      //no such element exception
   }
}
```

Src 7 : CSVOutputConverter.java

```
/**
Subclass of OutputConverter to handle CSV conversion output
Log File Aggregation (LFA) tool V1.1.

Copyright (C) 2008 Biomedical Informatics (BMI), St George's University of London. Citations to
the toolkit should be as 'de Lusignan S, Kumarapeli P, Chan T, Pflug B, van Vlymen J, Jones B,
Freeman GK. The ALFA (activity Log Files Aggregation) toolkit: a method for precise observation
of the consultation. <J Med Internet Res 2008; ..>.

This program is free software: you can redistribute it and/or modify it under the terms of the
GNU General Public License as published by the Free Software Foundation, either version 3 of the
License, or (at your option) any later version.

This program is distributed in the hope that it will be useful, but WITHOUT ANY WARRANTY;
without even the implied warranty of MERCHANTABILITY or FITNESS FOR A PARTICULAR
PURPOSE.  See the GNU General Public License for more details.

You should have received a copy of the GNU General Public License along with this program.  If
not, see <http://www.gnu.org/licenses/>.

*/

package aggregationtool;

import java.awt.event.ActionEvent;
import java.awt.event.ActionListener;

import java.util.ArrayList;
import java.util.zip.DataFormatException;

import javax.swing.JComboBox;
import javax.swing.JLabel;
import javax.swing.JPanel;


public class CSVOutputConverter extends OutputConverter {

   //****** properties ******
   //selectedable seperators
   private Character [] seperators = {';',',',':'};
```

```
private int selectedSepIndex=0;

//*** Property Panel components ***
JLabel sepLabel = new JLabel();
JComboBox sepComboBox = new JComboBox(seperators);

//***************************************************************************
//***************** constructor/building methods ***************************
//***************************************************************************

public CSVOutputConverter() {

    super();

    createPropertyPanel();
}

/**
 * creates a JPanel with all required properties for creating an SDS file
 */
protected void createPropertyPanel() {


    //*** Panel ***
    propertyPanel = new JPanel();

    propertyPanel.setLayout( null );

    //seperator label
    sepLabel.setText("select seperator for CSV format");
    sepLabel.setBounds(100,70,200,20);

    //combo box
    sepComboBox.setBounds(300,70,50,20);
    sepComboBox.setSelectedIndex(selectedSepIndex);
    sepComboBox.addActionListener(new ActionListener() {

        public void actionPerformed(ActionEvent e) {
            //set slectedSepIndex as new selected one in combo box
            selectedSepIndex = sepComboBox.getSelectedIndex();
        }
    });

    //add to panel
    propertyPanel.add(sepLabel);
    propertyPanel.add(sepComboBox);

}

//***************************************************************************
//************************* operating methods *****************************
//***************************************************************************
```

```
/**
 * creates output string of data entries depending on SDS format
 * @throws DataFormatException in any case an error occurs
 */
public void convertSource() throws DataFormatException {

    //check for data source
    if (dataSource != null) {

        //initialise output string
        outputString = "";

        //********************** process header ****************************

        //get content of event entry class
        Object [] eventItems = EventEntry.complexItemNames();

        //for all entries without the last add entry with seperator behind
        for (int i=0; i < eventItems.length-1; i++) {
            outputString += eventItems[i].toString() + seperators[selectedSepIndex];
        }

        //add last entry without seperator
        if (eventItems.length > 0) {
            outputString += eventItems[eventItems.length-1] + "\n";
        }

        //********************** process content ***************************

        ArrayList eventEntries = DataEntry.getAllEventEntries(dataSource);

        //go through all event entries
        for (int i=0; i < eventEntries.size(); i++) {

            //get current entry
            EventEntry entry = (EventEntry)eventEntries.get(i);

            //get content of entry
            Object [] entryContent = entry.complexItemsToArray();

            //check if at least one variable is given
            if (entryContent.length > 0) {

                //for all content variables except the last add with seperator behind
                for (int j = 0; j < entryContent.length-1; j++) {

                    outputString += entryContent[j].toString() + seperators[selectedSepIndex];
                }

                //add last entry with line seperator
                outputString += entryContent[entryContent.length-1] + "\n";
            }
        }
```

```
        }
        else {
            throw new DataFormatException("no data source set");
        }
    }
}
```

Src 8 : DataEntry.java

```
/**
Abstract class to define the basic structure of the common file format
Log File Aggregation (LFA) tool V1.1.

Copyright (C) 2008 Biomedical Informatics (BMI), St George's University of London. Citations to
the toolkit should be as 'de Lusignan S, Kumarapeli P, Chan T, Pflug B, van Vlymen J, Jones B,
Freeman GK. The ALFA (activity Log Files Aggregation) toolkit: a method for precise observation
of the consultation. <J Med Internet Res 2008; ..>.

This program is free software: you can redistribute it and/or modify it under the terms of the
GNU General Public License as published by the Free Software Foundation, either version 3 of the
License, or (at your option) any later version.

This program is distributed in the hope that it will be useful, but WITHOUT ANY WARRANTY;
without even the implied warranty of MERCHANTABILITY or FITNESS FOR A PARTICULAR
PURPOSE.  See the GNU General Public License for more details.

You should have received a copy of the GNU General Public License along with this program.  If
not, see <http://www.gnu.org/licenses/>.

*/

package aggregationtool;

import java.util.ArrayList;
import java.util.HashSet;
import java.util.Iterator;

public abstract class DataEntry implements Comparable,XMLConvertable {

    public abstract String toString();

    /**
     * @return all items of the subclass in an object array for displaying
     */
    public abstract Object[] itemsToDisplaystyle();

    /**
     * this method is for sorting an arraylist of dataentries
     * This functionality is needed to call sort function of collections class
     *
```

```
     * @param o object to compare with
     * @return integer which defines difference, 0 if they are equal
     */
    public int compareTo(Object o) {
        //if both are header, there are shown as equal
        if (this instanceof HeaderEntry && o instanceof HeaderEntry) {
            return ((HeaderEntry)this).compareTo((HeaderEntry)o);
        }
        //if this one is header and the other Data, this one has to be first
        else if (this instanceof HeaderEntry && o instanceof EventEntry) {
            return -1;
        }
        //opposite of the one before
        else if (this instanceof EventEntry && o instanceof HeaderEntry) {
            return 1;
        }
        //if both are EventEntry, it depends on the StartTime, then on the Endtime and at least on
the Event
        else if (this instanceof EventEntry && o instanceof EventEntry) {
            return ((EventEntry)this).compareTo((EventEntry)o);
        }
        else {
            System.out.println("DataEntry:compareTo: Instance of wrong classes!");
            return 0;
        }
    }

    /*
     * Methods for getting knowledge of content from ArrayList of DataEntries
     */

     /**
     * Searches for different types of EventEntries
     * @param Source arraylist with DataEntries
     * @return string array with different types of EventEntries
     */
     public static Object[] getUniqueEventTypes(ArrayList Source) {
         HashSet hs = new HashSet();

         Iterator it = Source.iterator();

         while (it.hasNext()) {
             DataEntry de = (DataEntry)it.next();

             if (de instanceof EventEntry) {
                 hs.add(((EventEntry)de).getEvent().getType().toString());
             }
         }

         return hs.toArray();
    }

    /**
```

```
     * Searches all different values of EventEntries
     * @param Source ArrayList with DataEntries
     * @return string array with all different values found in EventEntries
     */
    public static Object[] getUniqueEventValues(ArrayList Source) {
        HashSet hs = new HashSet();

        Iterator it = Source.iterator();

        while (it.hasNext()) {
            DataEntry de = (DataEntry)it.next();

            if (de instanceof EventEntry) {
                hs.add(((EventEntry)de).getEvent().getValue().toString());
            }
        }

        return hs.toArray();
    }

    /**
     * Depending on number of different Eventtypes, Source is splited into ArrayList for each
Eventtype
     * @param Source Arraylist with DataEntries
     * @return Array of Arraylists, separated by EventType
     */
    public static ArrayList[] splitOnEventType(ArrayList Source) {
        Object [] EventTypes = getUniqueEventTypes(Source);

        ArrayList [] splitContent= new ArrayList[EventTypes.length];

        //create for each position an arraylist
        for (int i=0; i< splitContent.length; i++) {
            splitContent[i] = new ArrayList();
        }

        Iterator it = Source.iterator();

        //go through list and add entry to one of arraylists depending on eventtype
        while (it.hasNext()) {
            DataEntry de = (DataEntry)it.next();

            if (de instanceof EventEntry) {
                EventEntry ee = (EventEntry)de;

                //Go through detected Types, and in case of match add to this arraylist
                for (int i=0; i< EventTypes.length; i++) {
                    if (EventTypes[i].equals(ee.getEvent().getType().toString())) {
                        splitContent[i].add(ee);
                    }
                }
            }
        }
```

```
    return splitContent;
}

/**
 * Searches for the highest start- and endtime in the source list
 * @param Source arraylist with DataEntries
 * @return the highest start- (int[0]) and endtime (int[1])
 */
public static float[] getHighestTimes(ArrayList Source) {

    Iterator it = Source.iterator();

    float highestST=0,highestET=0;

    while (it.hasNext()) {
        DataEntry de = (DataEntry)it.next();

        if (de instanceof EventEntry) {
            EventEntry ee = (EventEntry)de;

            if (ee.StartTime > highestST) {
                highestST = ee.StartTime;
            }

            if (ee.EndTime > highestET) {
                highestET = ee.EndTime;
            }
        }
    }

    return new float[] {highestST,highestET};
}

/**
 * Method to return event entries only, without header entries
 * @param Source Arraylist of data entries
 * @return Arraylist with evententries only
 */
public static ArrayList getAllEventEntries (ArrayList Source) {

    ArrayList EventEntries = new ArrayList();

    Iterator it = Source.iterator();

    while (it.hasNext()) {
        DataEntry de = (DataEntry)it.next();

        if (de instanceof EventEntry) {
            EventEntries.add(de);
        }
    }
```

```
        return EventEntries;
    }

    /**
     * Method to return header entries only, without event entries
     * @param Source ArrayList of data entries
     * @return ArrayList with headerentries only
     */
    public static ArrayList getAllHeaderEntries(ArrayList Source) {

        ArrayList HeaderEntries = new ArrayList();

        Iterator it = Source.iterator();

        while (it.hasNext()) {
            DataEntry de = (DataEntry)it.next();

            if (de instanceof HeaderEntry) {
                HeaderEntries.add(de);
            }
        }

        return HeaderEntries;
    }

    /*
     * Methods for test output
     */

    /**
     * This method creates a testoutput for an arraylist of DataEntries
     * @param Content
     */
    @Deprecated
    public static void SystemOutput(ArrayList Content) {
        Iterator it= Content.iterator();

        while (it.hasNext()) {
            System.out.println(it.next());
        }
    }
}
```

Src 9 : DisplayPanel.java

```
/**
Panel for Display step
Log File Aggregation (LFA) tool V1.1.
```

Copyright (C) 2008 Biomedical Informatics (BMI), St George's University of London. Citations to the toolkit should be as 'de Lusignan S, Kumarapeli P, Chan T, Pflug B, van Vlymen J, Jones B, Freeman GK. The ALFA (activity Log Files Aggregation) toolkit: a method for precise observation of the consultation. <J Med Internet Res 2008; ..>.

This program is free software: you can redistribute it and/or modify it under the terms of the GNU General Public License as published by the Free Software Foundation, either version 3 of the License, or (at your option) any later version.

This program is distributed in the hope that it will be useful, but WITHOUT ANY WARRANTY; without even the implied warranty of MERCHANTABILITY or FITNESS FOR A PARTICULAR PURPOSE. See the GNU General Public License for more details.

You should have received a copy of the GNU General Public License along with this program. If not, see <http://www.gnu.org/licenses/>.

```
*/

package aggregationtool;

import java.awt.Rectangle;
import java.awt.event.ActionEvent;
import java.awt.event.ActionListener;

import java.awt.event.KeyEvent;

import java.util.ArrayList;

import javax.swing.ButtonGroup;
import javax.swing.JButton;
import javax.swing.JDialog;
import javax.swing.JLabel;
import javax.swing.JPanel;
import javax.swing.JRadioButton;

public class DisplayPanel extends JPanel
implements StepPanelInterface, ActionListener{

    //manages all open Dialogs for Output
    private static ArrayList openDialogs= new ArrayList();

    //instance for superclass
    private GUISuperclass superclass;

    //data to display
    public ArrayList displayData;

    //Buttongroup for radiobuttons
    private ButtonGroup radioButtonGroup,typeButtonGroup;

    private static String TABULAR_BUTTON= "tabular output";
    private static String GRAPH_BUTTON = "graph output";
    private static String TYPE_COLUMN = "column style";
```

```
    private static String TYPE_HISTO = "histogram";

    private JButton next_button = new JButton();
    private JButton prev_button = new JButton();
    private JButton display_button = new JButton();

    private JLabel jLabel1 = new JLabel();

    private JRadioButton column_type;
    private JRadioButton histo_type;

    public DisplayPanel(GUISuperclass superclass) {

        this.superclass=superclass;

        //Choose datasource depending on the previous panel
        if(superclass.isAggregationEnabled()) {
            displayData = superclass.aggregationpanel.AggregationFile;
        }
        else {
            //When getting information from conversion panel, commonFile contains for each file an
arraylist with the data
            //Because we know that there must be exactly one Entry (otherwise aggregation would
have been called) we can
            //select the first entry in the list
            displayData = (ArrayList)superclass.conversionpanel.CommonFiles.get(0);
        }

        try {
            jbInit();
        } catch (Exception e) {
            e.printStackTrace();
        }
    }

    //*****************************************************************
    //************************ GUI creation ***************************
    //*****************************************************************

    /**
     * method to create GUI
     * @throws Exception
     */
    private void jbInit() throws Exception {
        this.setLayout( null );

        //display_button
         display_button.setText("display");
         display_button.setBounds(new Rectangle(370, 380, 80, 30));
         display_button.setToolTipText("display data source");
         display_button.setMnemonic(KeyEvent.VK_D);
         display_button.addActionListener(new ActionListener() {
                    public void actionPerformed(ActionEvent e) {
```

```
                process_display();
            }
        });

        //next_button
         next_button.setText("next");
         next_button.setBounds(new Rectangle(550, 380, 80, 30));
         next_button.setMnemonic(KeyEvent.VK_N);
         next_button.setToolTipText("next step");
         next_button.addActionListener(new ActionListener() {
            public void actionPerformed(ActionEvent e) {
                display_next();
            }
        });

        //prev_button
         prev_button.setText("prev");
         prev_button.setBounds(460,380,80,30);
         prev_button.setMnemonic(KeyEvent.VK_P);
         prev_button.setToolTipText("previous step");
         prev_button.addActionListener(new ActionListener() {
            public void actionPerformed(ActionEvent e) {
                display_previous();
            }
        });

        //radio buttons
        radioButtonGroup = new ButtonGroup();
        typeButtonGroup = new ButtonGroup();

        JRadioButton tab_button = new JRadioButton(TABULAR_BUTTON,true);
        tab_button.addActionListener(this);
        tab_button.setActionCommand(TABULAR_BUTTON);
        tab_button.setBounds(60,100,200,20);
        radioButtonGroup.add(tab_button);

        JRadioButton graph_button = new JRadioButton(GRAPH_BUTTON);
        graph_button.addActionListener(this);
        graph_button.setActionCommand(GRAPH_BUTTON);
        graph_button.setBounds(60,130,200,20);
        radioButtonGroup.add(graph_button);

        column_type = new JRadioButton(TYPE_COLUMN,true);
        column_type.setEnabled(false);
        column_type.setActionCommand(TYPE_COLUMN);
        column_type.setBounds(80,160,200,20);
        typeButtonGroup.add(column_type);

        histo_type = new JRadioButton(TYPE_HISTO);
        histo_type.setEnabled(false);
        histo_type.setActionCommand(TYPE_HISTO);
        histo_type.setBounds(80,180,200,20);
        typeButtonGroup.add(histo_type);
```

```
        //Label
        jLabel1.setText("Please choose one of the options below for ouput...");
        jLabel1.setBounds(new Rectangle(25, 15, 355, 30));

        //Add all to content pane
        this.add(jLabel1, null);
        this.add(next_button, null);
        this.add(prev_button,null);
        this.add(display_button,null);
        this.add(tab_button);
        this.add(graph_button);
        this.add(column_type);
        this.add(histo_type);
    }

    //*****************************************************************
    //********************** processing methods *************************
    //*****************************************************************

    /**
     * Method to display output dialog depending on choosen type
     */
    public void process_display() {

        //Show Dialog depending on choise
        JDialog displaydialog=null;
        String selectedButton = radioButtonGroup.getSelection().getActionCommand();

        if (selectedButton.equals(TABULAR_BUTTON)) {
            //tabular output
            displaydialog = new TabularDisplayDialog(superclass,"tabular
output",false,displayData,false);

            //add tablur dialog to dialog list for combining later
            DisplayPanel.addDialogToDialogList(displaydialog);
        }
        else if (selectedButton.equals(GRAPH_BUTTON)){
            //graph output
            int dialog_type=AbstractGraphDisplay.COLUMN;

            //column output style
            if (column_type.isSelected()) {
                dialog_type = AbstractGraphDisplay.COLUMN;
            }
            //histogram output style
            else if (histo_type.isSelected()) {
                dialog_type = AbstractGraphDisplay.HISTOGRAM;
            }

            //instance graph display dialog with selected settings
            displaydialog = new GraphDisplayDialog(superclass,"graph
output",false,displayData,dialog_type);
```

```
        }

        displaydialog.setVisible(true);
    }

    /**
     * Is called if one of the registerd radiobutton is clicked
     * in this case en-disable special options
     * @param e
     */
    public void actionPerformed(ActionEvent e) {

        if (e.getSource() instanceof JRadioButton) {

            String command = e.getActionCommand();

            //by clicking graph button disable suboptions
            if (command.equals(GRAPH_BUTTON)) {
                column_type.setEnabled(true);
                histo_type.setEnabled(true);
            }
            //by clicking tabular button disable suboptions of graph button
            else if (command.equals(TABULAR_BUTTON)) {
                column_type.setEnabled(false);
                histo_type.setEnabled(false);
            }
        }
    }

    //*****************************************************************
    //****************** Methods for Navigation *************************
    //*****************************************************************
    /**
     * display next panel
     */
    public void display_next() {

        this.setVisible(false);

        superclass.outputpanel = new OutputPanel(superclass);

        superclass.displayPanel(superclass.outputpanel,6);
    }

    /**
     * display previous panel, depending on previous step
     */
    public void display_previous() {
        this.setVisible(false);

        if (superclass.isAggregationEnabled()) {
            superclass.displayPanel(superclass.aggregationpanel,4);
        }
```

```
        else {
            superclass.displayPanel(superclass.conversionpanel,3);
        }
    }

    //*******************************************************************
    //Methods for Dialoghandling
    //*******************************************************************
     /**
      * Each dialog with output must be added to this list when it is shown
      * @param dialog
      */
     public static void addDialogToDialogList(JDialog dialog) {
         openDialogs.add(dialog);
     }

     /**
      * On closing, the dialog has to remove himself from the list
      * @param dialog
      */
     public static void removeDialogFromDialogList(JDialog dialog) {
         openDialogs.remove(dialog);
     }

     /**
     * @return arraylist with open dialogs
     */
     public static ArrayList getOpenDialogsList() {
         return openDialogs;
     }
}
```

Src 10 : errDialogEntry.java

/**
used by ErrorDialog to contain the entries for the display
Log File Aggregation (LFA) tool V1.1.

Copyright (C) 2008 Biomedical Informatics (BMI), St George's University of London. Citations to the toolkit should be as 'de Lusignan S, Kumarapeli P, Chan T, Pflug B, van Vlymen J, Jones B, Freeman GK. The ALFA (activity Log Files Aggregation) toolkit: a method for precise observation of the consultation. <J Med Internet Res 2008; ..>.

This program is free software: you can redistribute it and/or modify it under the terms of the GNU General Public License as published by the Free Software Foundation, either version 3 of the License, or (at your option) any later version.

This program is distributed in the hope that it will be useful, but WITHOUT ANY WARRANTY; without even the implied warranty of MERCHANTABILITY or FITNESS FOR A PARTICULAR PURPOSE. See the GNU General Public License for more details.

You should have received a copy of the GNU General Public License along with this program. If not, see <http://www.gnu.org/licenses/>.

```
*/


package aggregationtool;

import java.util.ArrayList;
import java.util.Iterator;

public class errDialogEntry {

        //Type of entry (INFO, WARNING, ERROR)
        int type;

        //Text to be display in dialog
        String text;

        public errDialogEntry(int type, String text) {
            this.type=type;
            this.text=text;
        }

        public String toString() {
            return text;
        }

        /**
        * USE ERROR DIALOG FUNCTIONALITY INSTEAD
        * @param Messages
        * @return
        */
        @Deprecated
        public static boolean containsErrors(ArrayList Messages) {
            Iterator it = Messages.iterator();

            while (it.hasNext()) {
                errDialogEntry er = (errDialogEntry)it.next();

                if (er.type == ErrorDialog.ERROR) {
                    return true;
                }
            }
            return false;
        }
    }
```

Src 11: ErrorDialog.java

```
/**
ErrorDialog used for any error, warning or info output from the program,except the mediaplayer output
Log File Aggregation (LFA) tool V1.1.

Copyright (C) 2008 Biomedical Informatics (BMI), St George's University of London. Citations to the toolkit should be as 'de Lusignan S, Kumarapeli P, Chan T, Pflug B, van Vlymen J, Jones B, Freeman GK. The ALFA (activity Log Files Aggregation) toolkit: a method for precise observation of the consultation. <J Med Internet Res 2008; ..>.

This program is free software: you can redistribute it and/or modify it under the terms of the GNU General Public License as published by the Free Software Foundation, either version 3 of the License, or (at your option) any later version.

This program is distributed in the hope that it will be useful, but WITHOUT ANY WARRANTY; without even the implied warranty of MERCHANTABILITY or FITNESS FOR A PARTICULAR PURPOSE.  See the GNU General Public License for more details.

You should have received a copy of the GNU General Public License along with this program.  If not, see <http://www.gnu.org/licenses/>.

*/
package aggregationtool;

import java.awt.BorderLayout;
import java.awt.Color;
import java.awt.Dimension;
import java.awt.Frame;

import java.awt.event.ActionEvent;
import java.awt.event.ActionListener;

import java.awt.event.KeyEvent;
import java.awt.event.KeyListener;

import java.util.ArrayList;

import java.util.Iterator;

import javax.swing.JButton;
import javax.swing.JDialog;
import javax.swing.JLabel;
import javax.swing.JScrollPane;
import javax.swing.JTable;
import javax.swing.JTextArea;
import javax.swing.ListSelectionModel;
import javax.swing.table.DefaultTableModel;

public class ErrorDialog extends JDialog
implements KeyListener{
```

```
//statics for Type definition
public static int ERROR = 1;
public static int WARNING = 2;
public static int INFO = 3;

//Error - Warnings list
private ArrayList errorWarnings = new ArrayList();

//GUI elements
private DefaultTableModel tableModel;
private JTable jTable1 = new JTable(tableModel) {

    //to set all cells editable false
    public boolean isCellEditable(int row,int col) {
        return false;
    }
};

private JButton jButton1 = new JButton();
private JTextArea jTextArea2 = new JTextArea();
private JLabel dummyLabel= new JLabel(" ");
private JLabel dummyLabel2= new JLabel(" ");

//************************************************************************
//*********************** constructors **************************************
//************************************************************************
public ErrorDialog() {
    this(null, "", false);

    addKeyListener(this);
}

public ErrorDialog(Frame parent, String title, boolean modal) {
    super(parent, title, modal);

    try {
        jbInit();
    } catch (Exception e) {
        e.printStackTrace();
    }

    //don't know why key listener only works by adding to jtable, not with dialog
    jTable1.addKeyListener(this);

}
//************************************************************************
//*********************** GUI initialising *********************************
//************************************************************************
/**
 * creates all the GUI elements
 * @throws Exception
 */
```

```
private void jbInit() throws Exception {
    this.setSize(new Dimension(543, 337));
    this.getContentPane().setLayout( new BorderLayout(10,10) );

    //*********
    //create JTable
    jTable1.setDefaultRenderer(Object.class,new ErrorTableCellRenderer());
    jTable1.setSelectionMode(ListSelectionModel.SINGLE_SELECTION);
    jTable1.setCellSelectionEnabled(false);
    //*********

    jButton1.setText("OK");
    jButton1.addActionListener(new ActionListener() {
                public void actionPerformed(ActionEvent e) {
                    jButton1_actionPerformed(e);
                }
            });
    jTextArea2.setText("Following errors occured");
    jTextArea2.setEnabled(false);
    jTextArea2.setEditable(false);
    jTextArea2.setDisabledTextColor(new Color(49, 49, 49));
    this.getContentPane().add(jButton1,BorderLayout.SOUTH);
    this.getContentPane().add(new JScrollPane(jTable1),BorderLayout.CENTER);
    this.getContentPane().add(jTextArea2,BorderLayout.NORTH);
    this.getContentPane().add(dummyLabel,BorderLayout.WEST);
    this.getContentPane().add(dummyLabel2,BorderLayout.EAST);
}
//*************************************************************************
//******************* operate with Error Warnings list *********************
//*************************************************************************

/**
 * Adds a new Message with type and message string to the dialog list
 * @param errMsg text to display
 * @param errorCode defines whether error or warning
 */
public void addMessage(String errMsg, int errorCode) {
    errorWarnings.add(new errDialogEntry(errorCode,errMsg));
}

/**
 * Adds an arraylist containing errDialogEntries
 * Unpurified use because arraylist with errdialog entries must be created outside
 * @param errorList
 */
@Deprecated
public void addAllMsg(ArrayList errorList) {
    errorWarnings.addAll(errorList);
}

/**
 * removes all existing errors and warnings from the list
 */
```

```
public void removeAllMsg() {
    errorWarnings.clear();
}

/**
 * @return true if at least one error is in list, otherwise false
 */
public boolean containsErrors() {
    Iterator it = errorWarnings.iterator();

    while (it.hasNext()) {
        errDialogEntry errEntry = (errDialogEntry)it.next();

        if (errEntry.type == ErrorDialog.ERROR) {
            return true;
        }
    }

    return false;
}

/**
 * @return true if at least one warning is in list, otherwise false
 */
public boolean containsWarnings() {
    Iterator it = errorWarnings.iterator();

    while (it.hasNext()) {
        errDialogEntry errEntry = (errDialogEntry)it.next();

        if (errEntry.type == ErrorDialog.WARNING) {
            return true;
        }
    }

    return false;
}

/**
 * @return false if at least one entry is not from type INFO, otherwise true
 */
public boolean containsOnlyInfos() {
    Iterator it = errorWarnings.iterator();

    while (it.hasNext()) {
        errDialogEntry errEntry = (errDialogEntry)it.next();

        if (errEntry.type != ErrorDialog.INFO) {
            return false;
        }
    }

    return true;
```

```
}

/**
 * @return true if any messages are in queue
 */
public boolean containsAnyMsg() {
    return !errorWarnings.isEmpty();
}

 //**************************************************************************
 //*********************** display dialog **********************************
 //**************************************************************************
 /**
 * Shows the error dialog with all set messages in the error list
 */
public void showErrDialog() {
    tableModel = new DefaultTableModel();

    tableModel.setColumnIdentifiers(new Object[] {"Errors/Warnings/Infos"});
    for (int i=0; i<errorWarnings.size();i++) {
        tableModel.addRow(new Object[] {errorWarnings.get(i)});
    }
    jTable1.setModel(tableModel);
    this.setVisible(true);
}

//**************************************************************************
//*********************** window handling *********************************
//**************************************************************************
/**
 * If ok button is pressed, hide window
 * @param e
 */
private void jButton1_actionPerformed(ActionEvent e) {
    this.setVisible(false);
    this.dispose();
}

//**************************************************************************
//************************** key handling *********************************
//**************************************************************************
public void keyTyped(KeyEvent e) {
    //do nothing
}

public void keyPressed(KeyEvent e) {

    //in case enter pressed, close dialog
    if (e.getKeyCode() == KeyEvent.VK_ENTER) {
        this.setVisible(false);
        this.dispose();
    }
}
```

```
    public void keyReleased(KeyEvent e) {
        //do nothing
    }
}
```

Src 12 : ErrorTableCellRenderer.java

```
/**
Displays the table with errors
Log File Aggregation (LFA) tool V1.1.

Copyright (C) 2008 Biomedical Informatics (BMI), St George's University of London. Citations to the toolkit should be as 'de Lusignan S, Kumarapeli P, Chan T, Pflug B, van Vlymen J, Jones B, Freeman GK. The ALFA (activity Log Files Aggregation) toolkit: a method for precise observation of the consultation. <J Med Internet Res 2008; ..>.

This program is free software: you can redistribute it and/or modify it under the terms of the GNU General Public License as published by the Free Software Foundation, either version 3 of the License, or (at your option) any later version.

This program is distributed in the hope that it will be useful, but WITHOUT ANY WARRANTY; without even the implied warranty of MERCHANTABILITY or FITNESS FOR A PARTICULAR PURPOSE.  See the GNU General Public License for more details.

You should have received a copy of the GNU General Public License along with this program.  If not, see <http://www.gnu.org/licenses/>.

*/

package aggregationtool;

import java.awt.*;
import javax.swing.*;
import javax.swing.table.*;

public class ErrorTableCellRenderer
implements TableCellRenderer
{
  public Component getTableCellRendererComponent(
    JTable table,
    Object value,
    boolean isSelected,
    boolean hasFocus,
    int row,
    int column
  )
 {
    //create Label for cell
    JLabel label = new JLabel(value.toString());
    label.setOpaque(true);
```

```
    //set layout depending on table layout
    label.setFont(table.getFont());
    label.setForeground(table.getForeground());
    //default
    label.setBackground(table.getBackground());

    //get errordialog entry as given value
    errDialogEntry entry = (errDialogEntry) value;

    //Depending on the error Type set background
    if (entry != null ) {
        if (entry.type==ErrorDialog.ERROR) {
            label.setBackground(new Color(250,80,100));
        }
        else if (entry.type==ErrorDialog.WARNING) {
            label.setBackground(Color.YELLOW);
        }
        else if (entry.type==ErrorDialog.INFO) {
            label.setBackground(Color.GREEN);
        }
    }
    else {
         label.setBackground(Color.WHITE);
    }

    return label;
 }
}
```

Src 13 : EventEntry.java

```
/**
Subclass of Data entry which contains one entry of a read and converted file to the common file format
Log File Aggregation (LFA) tool V1.1.

Copyright (C) 2008 Biomedical Informatics (BMI), St George's University of London. Citations to the toolkit should be as 'de Lusignan S, Kumarapeli P, Chan T, Pflug B, van Vlymen J, Jones B, Freeman GK. The ALFA (activity Log Files Aggregation) toolkit: a method for precise observation of the consultation. <J Med Internet Res 2008; ..>.

This program is free software: you can redistribute it and/or modify it under the terms of the GNU General Public License as published by the Free Software Foundation, either version 3 of the License, or (at your option) any later version.

This program is distributed in the hope that it will be useful, but WITHOUT ANY WARRANTY; without even the implied warranty of MERCHANTABILITY or FITNESS FOR A PARTICULAR PURPOSE.  See the GNU General Public License for more details.
```

```
You should have received a copy of the GNU General Public License along with this program.  If
not, see <http://www.gnu.org/licenses/>.

*/

package aggregationtool;

import org.w3c.dom.Document;
import org.w3c.dom.Element;
import org.w3c.dom.NodeList;

public class EventEntry
extends DataEntry{

    //defines value endtime is set in case no duration is set
    public static float ENDTIME_NO_DURATION_VALUE = -1;
    public static String ENDTIME_NO_DURATION_STRING = "---";

    public float StartTime;
    public float EndTime;
    private EventInfo Event;

    /***************************************************************************
     ****************************** constructor ********************************
     **************************************************************************/

    public EventEntry () {
        Event = new EventInfo();
    }

    public EventEntry (float StartTime,float EndTime, String EventType,String EventValue) {
        this.StartTime=StartTime;
        this.EndTime=EndTime;
        this.Event= new EventInfo(EventType,EventValue);
    }

    /***************************************************************************
     ***************** methods for display and operation ***********************
     **************************************************************************/
    public String toString() {
        return "Starttime:"+StartTime+"/Endtime: "+EndTime+"/Event:"+Event;
    }

    /**
     * Compares two EventEntries
     * 1) by StartTime
     * 2) by EndTime
     * 3) by Event
     * @param ev2
     * @return
     */
    public int compareTo (EventEntry ev2) {
```

```
        int st,et;

        if ((st = Float.compare(this.StartTime,ev2.StartTime)) ==0) {
            if ((et = Float.compare(this.EndTime,ev2.EndTime)) == 0) {
                return this.Event.compareTo(ev2.Event);
            }
            else {
                return et;
            }
        }
        else {
            return st;
        }
    }

    /**
     * Get information about evententry whether entry contains duration or only
     * an event without duration
     * @return false in case of no duration, true otherwise
     */
    public boolean isDuration() {

        if (EndTime == ENDTIME_NO_DURATION_VALUE) {
            return false;
        } else {
            return true;
        }
    }

    /**
     * !!! change in case new basic items added !!!
     * Does not contain real values of all variables
     * @return all basic items in an object array or displaying
     */
    public Object[] itemsToDisplaystyle() {
        Object [] array = new Object[3];

        //**** starttime ****
        array[0]= StartTime;

        //**** endtime ****
        //If EndTime is ENDTIME_NO_DURATION there is no duration
        if (EndTime == ENDTIME_NO_DURATION_VALUE) {
            array[1] = ENDTIME_NO_DURATION_STRING;
        }
        else {
            array[1] = EndTime;
        }

        array[2]= Event.toString();

        return array;
    }
```

Biomedical Informatics, Division of Community Health Sciences
Tel + 44 (0)20 8725 5661 Fax + 44 (0)20 8725 3584 Email slusigna@sgul.ac.uk

```
/**
 * !!! change in case new items added in EvenEntry, not in EventInfo!!
 * @return object array with all information of class in with real values
 */
 public Object[] complexItemsToArray() {
     Object [] array = new Object[2 + Event.numberOfItems()];

     //*** starttime ***
     array [0] = this.StartTime;

     //*** endtime ***
     array[1] = this.EndTime;

     //*** event ***
     Object [] eventItems = Event.complexeItemsToArray();

     for (short s =0; s < Event.numberOfItems(); s++) {
         array[s+2] = eventItems[s];
     }

     return array;
 }

/**
 * !!! change in case new items added !!!
 * This method is used for display to combine event information
 * @return the names of basic the items in an object array
 */
public static Object[] itemDisplayNames() {
    return new String[] {"Starttime","Endtime","Event"};
}

/**
 * !!! change in case new items added !!!
 * Currently this method is used for CSV output to detail information
 * @return the name of all items, also subinformation of event
 */
public static Object[] complexItemNames() {
    return new String[] {"Starttime","Endtime","Event type","Event value"};
}

/**************************************************************************
 ******************** accessors for variables ******************************
 *************************************************************************/

public void setStartTime(float startTime) {
    this.StartTime = startTime;
}

public float getStartTime() {
    return StartTime;
}
```

```
    public void setEndTime(float endTime) {
        this.EndTime = endTime;
    }

    public float getEndTime() {
        return EndTime;
    }

    public void setEvent(String Type, String Value) {
        this.Event = new EventInfo(Type,Value);
    }

    public EventEntry.EventInfo getEvent() {
        return Event;
    }

     /*************************************************************************
      ************************* xml functionality ****************************
      ************************************************************************/
    /**
     * create Element with content of all information to be displayed in XML file
     * @param document to create elements
     * @return class element with all added information
    */
    public Element createXMLElement(Document document) {

        Element classElement = document.createElement(this.getClass().getSimpleName());

        //starttime
        Element elemStarttime = document.createElement("start_time");
        elemStarttime.appendChild(document.createTextNode(""+StartTime));
        classElement.appendChild(elemStarttime);

        //endtime
         Element elemEndtime = document.createElement("end_time");
         elemEndtime.appendChild(document.createTextNode(""+EndTime));
         classElement.appendChild(elemEndtime);

        //*** Event ***
        classElement.appendChild(Event.createXMLElement(document));

        return classElement;
    }

    /**
     * Method to set settings by an given element of an xml file
     * @param classElement xml element with data source
     */
     public void setPropertiesByXML(Element classElement) {

         //starttime
         NodeList startL = classElement.getElementsByTagName("start_time");
```

```
        StartTime = Float.parseFloat(startL.item(0).getChildNodes().item(0).getNodeValue());

        //endtime
        NodeList endL = classElement.getElementsByTagName("end_time");
        EndTime = Float.parseFloat(endL.item(0).getChildNodes().item(0).getNodeValue());

        //event
        NodeList eventL =
classElement.getElementsByTagName(EventInfo.class.getSimpleName());
        Event.setPropertiesByXML((Element)eventL.item(0));
    }

    /***************************************************************************
     ********************** inner class for event *****************************
     **************************************************************************/

    public class EventInfo implements XMLConvertable{

        //************* variables ****************
        //unique Eventtype
        private String Type;

        //value of event
        private String Value;

        //****************************************
        //************* constructors **************
        //****************************************
        public EventInfo() {

        }

        public EventInfo(String Type, String Value) {
            this.Type=Type;
            this.Value=Value;
        }

        //****************************************
        //********* basic functionality ***********
        //****************************************
        public String toString() {
            return Type+" "+Value;
        }

        /**
         * Compares two EventInfo
         * 1) by Type
         * 2) by Value
         * @param ei
         * @return
         */
        public int compareTo(EventInfo ei) {
```

```
        //primary sort by type, then by value
        if (this.Type.equals(ei.Type)) {
            return this.Value.compareTo(ei.Value);
        }
        else {
            return this.Type.compareTo(ei.Type);
        }
    }

    /**
     * !! must be changed in case new variables where added !!
     * @return the number of variables this class contains
     */
    public int numberOfItems() {
        return 2;
    }

    /**
     * @return all variables in object array
     */
    public Object[] complexeItemsToArray() {
        Object [] items = new Object[2];

        //Type
        items[0] = Type;

        //Value
        items[1] = Value;

        return items;
    }

    //*****************************************
    //************** accessors ****************
    //*****************************************
    public void setType (String Type) {
        this.Type = Type;
    }

    public String getType () {
        return this.Type;
    }

    public void setValue(String Value) {
        this.Value = Value;
    }

    public String getValue () {
        return this.Value;
    }

    //*****************************************
    //*********** XML support ****************
```

```
        //*****************************************
        public Element createXMLElement(Document document) {

            Element classElement = document.createElement(this.getClass().getSimpleName());

            //type
            Element elemType = document.createElement("type");
            elemType.appendChild(document.createTextNode(getType()));
            classElement.appendChild(elemType);

            //value
             Element elemValue = document.createElement("value");
             elemValue.appendChild(document.createTextNode(getValue()));
             classElement.appendChild(elemValue);

             return classElement;
        }

        public void setPropertiesByXML(Element classElement) {

            //type
            NodeList typeL = classElement.getElementsByTagName("type");
            setType(typeL.item(0).getChildNodes().item(0).getNodeValue());

            //value
            NodeList valueL = classElement.getElementsByTagName("value");
            setValue(valueL.item(0).getChildNodes().item(0).getNodeValue());
        }
    }
}
```

Src 14 : GraphDisplayDialog.java

/**
builds the frame for all graph based output formats
Log File Aggregation (LFA) tool V1.1.

Copyright (C) 2008 Biomedical Informatics (BMI), St George's University of London. Citations to the toolkit should be as 'de Lusignan S, Kumarapeli P, Chan T, Pflug B, van Vlymen J, Jones B, Freeman GK. The ALFA (activity Log Files Aggregation) toolkit: a method for precise observation of the consultation. <J Med Internet Res 2008; ..>.

This program is free software: you can redistribute it and/or modify it under the terms of the GNU General Public License as published by the Free Software Foundation, either version 3 of the License, or (at your option) any later version.

This program is distributed in the hope that it will be useful, but WITHOUT ANY WARRANTY; without even the implied warranty of MERCHANTABILITY or FITNESS FOR A PARTICULAR PURPOSE. See the GNU General Public License for more details.

You should have received a copy of the GNU General Public License along with this program. If not, see <http://www.gnu.org/licenses/>.

```
*/

package aggregationtool;

import java.awt.BorderLayout;
import java.awt.Dimension;
import java.awt.GridBagConstraints;
import java.awt.GridBagLayout;

import java.awt.Insets;

import java.util.ArrayList;

import javax.swing.JDialog;
import javax.swing.JPanel;
import javax.swing.JScrollPane;

public class GraphDisplayDialog extends JDialog
{
    //instance of GUI Superclass
    protected GUISuperclass superclass;

    //combined datasource given by constructor
    private ArrayList DataSource;
    //Array containing all displayed panels depending on split by eventtype
    private AbstractGraphDisplay [] Panels;

    //depending on this type the subclasses of AbstractGraphDisplay are choosen to be displayed
    private int DisplayStyle;

    public GridBagLayout layout;
    public JPanel Content;


    public GraphDisplayDialog(GUISuperclass parent, String title, boolean modal,ArrayList
DataSource,int DisplayStyle) {
        super(parent, title, modal);

        this.superclass = parent;
        this.DataSource = DataSource;
        this.DisplayStyle = DisplayStyle;
        try {
            jbInit();
        } catch (Exception e) {
            e.printStackTrace();
        }
    }

    /**
     * Initialising GUI
```

```
     * @throws Exception
     */
    private void jbInit() throws Exception {

        //Checking different types of Eventtypes, and therefore create Gridlayout with same
number of rows
        Object [] EventTypes = DataEntry.getUniqueEventTypes(DataSource);

        //Split given DataSource by EventTypes into several ArrayLists
        ArrayList [] Sources = DataEntry.splitOnEventType(this.DataSource);

        //Array to keep all the panels for display to be able to change it afterwards
        Panels = new AbstractGraphDisplay[Sources.length];

        //Depending on the number of different types in Arraylist, create rows to display
        this.getContentPane().setLayout(new BorderLayout());
        layout = new GridBagLayout();
        GridBagConstraints gbc;

        Content = new JPanel( layout );


        //For each Eventtype create Panel and display in Layout
        for (int i=0; i< Sources.length; i++) {

            AbstractGraphDisplay panel =
AbstractGraphDisplay.getPanelForType(this,(String)EventTypes[i],DisplayStyle,Sources[i]);

            //store panel in Panel list
            Panels[i] = panel;

            //get minimum size for window
            Dimension minSize = panel.getMinimumDisplaySize();

            gbc =
makegbc(i,0,1,1,GridBagConstraints.BOTH,(int)minSize.getWidth(),(int)minSize.getHeight());

            //set properties to gridbaglayout
            layout.setConstraints(panel,gbc);

            Content.add(panel);
        }

        //at the end add JPanel in content in center with a ScrollPane
        this.getContentPane().add(new JScrollPane(Content),BorderLayout.CENTER);

        pack();
    }

    /**
     * This method is called from a subclass each time a content changed
     * it repaints the whole dialog
     * @param agd
```

```
    */
    public void windowChanged(AbstractGraphDisplay agd) {
        Content.removeAll();
        GridBagConstraints gbc;

        for (int i =0; i< Panels.length; i++) {

                //get minimum size for window
                Dimension minSize = Panels[i].getMinimumDisplaySize();
                gbc =
makegbc(i,0,1,1,GridBagConstraints.BOTH,(int)minSize.getWidth(),(int)minSize.getHeight());

                //set properties to gridbaglayout
                layout.setConstraints(Panels[i],gbc);

                Content.add(Panels[i]);
        }

        pack();
    }


    /**
     * creates the settings for a component which should be displayed in GridBagLayout
     * @param x startcoordinate of component
     * @param y startcoordinate of component
     * @param width how many cells
     * @param height how many cells
     * @return
     */
    private GridBagConstraints makegbc (int x, int y, int width, int height,int fill, int ipadx, int
ipady) {
        GridBagConstraints gbc = new GridBagConstraints();
        gbc.gridx = x;
        gbc.gridy = y;
        gbc.gridwidth = width;
        gbc.gridheight = height;
        gbc.fill = fill;
        gbc.ipadx = ipadx;
        gbc.ipady = ipady;
        //Defines the border of each element
        gbc.insets = new Insets(10, 10, 10, 10);

        return gbc;
    }
}
```

Src 15 : GUISuperclass.java

```
/**
defines the frame for the program, displays navigation on top and panel
```

Log File Aggregation (LFA) tool V1.1.

Copyright (C) 2008 Biomedical Informatics (BMI), St George's University of London. Citations to the toolkit should be as 'de Lusignan S, Kumarapeli P, Chan T, Pflug B, van Vlymen J, Jones B, Freeman GK. The ALFA (activity Log Files Aggregation) toolkit: a method for precise observation of the consultation. <J Med Internet Res 2008; ..>.

This program is free software: you can redistribute it and/or modify it under the terms of the GNU General Public License as published by the Free Software Foundation, either version 3 of the License, or (at your option) any later version.

This program is distributed in the hope that it will be useful, but WITHOUT ANY WARRANTY; without even the implied warranty of MERCHANTABILITY or FITNESS FOR A PARTICULAR PURPOSE. See the GNU General Public License for more details.

You should have received a copy of the GNU General Public License along with this program. If not, see <http://www.gnu.org/licenses/>.

```
*/

package aggregationtool;

import java.awt.Dimension;
import java.awt.Rectangle;
import java.awt.event.ActionEvent;
import java.awt.event.ActionListener;
import javax.swing.JButton;
import javax.swing.JFrame;
import javax.swing.JPanel;
import javax.swing.JSeparator;

public class GUISuperclass extends JFrame
{
    private JSeparator jSeparator1 = new JSeparator();
    private JButton aggregation_button = new JButton();
    private JButton start_button = new JButton();
    private JButton conversion_button = new JButton();
    private JButton display_button = new JButton();
    private JButton settings_button = new JButton();
    private JButton output_button = new JButton();

    //Panel references
    public StartPanel startpanel;
    public SettingsPanel settingspanel;
    public ConversionPanel conversionpanel;
    public AggregationPanel aggregationpanel;
    public DisplayPanel displaypanel;
    public OutputPanel outputpanel;

    //steps which can be disabled
    private boolean aggregation_enabled=true;

    public JPanel aktPanel;
```

```
public GUISuperclass() {
    super("Aggregation Tool");

    try {
        jbInit();

        //basic settings
        this.addWindowListener(new WindowClosingAdapter());

        //when initialising create and display startpanel
        startpanel= new StartPanel(this);
        displayPanel(startpanel,1);

    } catch (Exception e) {
        e.printStackTrace();
    }


}

/**
 * create GUI elements
 * @throws Exception
 */
private void jbInit() throws Exception {

    //basic settings
    this.getContentPane().setLayout( null );
    this.setSize(new Dimension(673, 513));
    this.setResizable(false);

    jSeparator1.setBounds(new Rectangle(0, 50, 670, 10));

    //start button
    start_button.setText("Start");
    start_button.setBounds(new Rectangle(10, 15, 95, 25));
    start_button.addActionListener(new ActionListener() {
                public void actionPerformed(ActionEvent e) {
                    start_button_actionPerformed(e);
                }
            });
    //settings button
    settings_button.setText("Settings");
    settings_button.setBounds(new Rectangle(115, 15, 95, 25));
    settings_button.addActionListener(new ActionListener() {
                public void actionPerformed(ActionEvent e) {
                    settings_button_actionPerformed(e);
                }
            });
    //conversion button
    conversion_button.setText("Conversion");
    conversion_button.setBounds(new Rectangle(220, 15, 100, 25));
```

```
        conversion_button.addActionListener(new ActionListener() {
                    public void actionPerformed(ActionEvent e) {
                        conversion_button_actionPerformed(e);
                    }
                });
        //aggregation button
        aggregation_button.setText("Aggregation");
        aggregation_button.setBounds(new Rectangle(330, 15, 105, 25));
        aggregation_button.addActionListener(new ActionListener() {
                    public void actionPerformed(ActionEvent e) {
                        aggregation_button_actionPerformed(e);
                    }
                });
        //display button
        display_button.setText("Display");
        display_button.setBounds(new Rectangle(445, 15, 100, 25));
        display_button.addActionListener(new ActionListener() {
                    public void actionPerformed(ActionEvent e) {
                        display_button_actionPerformed(e);
                    }
                });
        //output button
        output_button.setText("Output");
        output_button.setBounds(new Rectangle(560, 15, 95, 25));
        output_button.addActionListener(new ActionListener() {
                    public void actionPerformed(ActionEvent e) {
                        output_button_actionPerformed(e);
                    }
                });
        this.getContentPane().add(output_button, null);
        this.getContentPane().add(settings_button, null);
        this.getContentPane().add(display_button, null);
        this.getContentPane().add(conversion_button, null);
        this.getContentPane().add(start_button, null);
        this.getContentPane().add(aggregation_button, null);
        this.getContentPane().add(jSeparator1, null);
    }

    /**
     * This method should be called every time another step should be displayed
     * Therefore the panel which should be called must exist and initialied
     * @param panel to be displayed, must already exist
     * @param Step starting with 1 by startpanel to define which buttons should be disabled
     */
    public void displayPanel(JPanel panel, int Step) {

        //define properties of panel and display it
        panel.setLocation(StepPanelInterface.XLocation,StepPanelInterface.YLocation);
        panel.setSize(new Dimension(StepPanelInterface.WIDE, StepPanelInterface.HIGH));
        panel.setVisible(true);
        this.aktPanel=panel;

        this.getContentPane().add(panel);
```

```
        //Enable-disable buttons
        setButtons(Step);
    }

    /**
     * Method to enable/disable top navigations buttons depending on current step
     * @param Step starting with 1 by startpanel
     */
    public void setButtons(int Step) {
        if (Step<2) {
            settings_button.setEnabled(false);
        }
        else {
            settings_button.setEnabled(true);
        }
        if (Step<3) {
            conversion_button.setEnabled(false);
        }
        else {
            conversion_button.setEnabled(true);
        }
        if (Step<4 || !aggregation_enabled) {
            aggregation_button.setEnabled(false);
        }
        else {
            aggregation_button.setEnabled(true);
        }
        if (Step<5) {
            display_button.setEnabled(false);
        }
        else {
            display_button.setEnabled(true);
        }
        if (Step<6) {
            output_button.setEnabled(false);
        }
        else {
            output_button.setEnabled(true);
        }
    }

    //*****************************************************************************
    //************ Eventhanling for top navigation buttons ********************
    //*****************************************************************************

    private void start_button_actionPerformed(ActionEvent e) {
        aktPanel.setVisible(false);
        displayPanel(this.startpanel,1);
    }

    private void settings_button_actionPerformed(ActionEvent e) {
        aktPanel.setVisible(false);
```

```
        displayPanel(this.settingspanel,2);
    }

    private void conversion_button_actionPerformed(ActionEvent e) {
        aktPanel.setVisible(false);
        displayPanel(this.conversionpanel,3);
    }

    private void aggregation_button_actionPerformed(ActionEvent e) {
        aktPanel.setVisible(false);
        displayPanel(this.aggregationpanel,4);
    }

    private void display_button_actionPerformed(ActionEvent e) {
        aktPanel.setVisible(false);
        displayPanel(this.displaypanel,5);
    }

    private void output_button_actionPerformed(ActionEvent e) {
        aktPanel.setVisible(false);
        displayPanel(this.outputpanel,6);
    }


    //************************************************************************
    //************ methods to handle ability of aggregation step ***************
    //************************************************************************

    /**
     * Method to define whether aggregation mode is enabled
     * @param enable true to enable aggregation button
     */
    public void setAggregationEnabled(boolean enable) {
            aggregation_enabled=enable;
    }

    /**
     * gets information about whether aggregation step is enabled
     * @return
     */
    public boolean isAggregationEnabled() {
        return aggregation_enabled;
    }
}
```

Src 16 : HeaderEntry.java

```
/**
Subclass of DataEntry containig headerinformation of the read file
Log File Aggregation (LFA) tool V1.1.
```

Copyright (C) 2008 Biomedical Informatics (BMI), St George's University of London. Citations to the toolkit should be as 'de Lusignan S, Kumarapeli P, Chan T, Pflug B, van Vlymen J, Jones B, Freeman GK. The ALFA (activity Log Files Aggregation) toolkit: a method for precise observation of the consultation. <J Med Internet Res 2008; ..>.

This program is free software: you can redistribute it and/or modify it under the terms of the GNU General Public License as published by the Free Software Foundation, either version 3 of the License, or (at your option) any later version.

This program is distributed in the hope that it will be useful, but WITHOUT ANY WARRANTY; without even the implied warranty of MERCHANTABILITY or FITNESS FOR A PARTICULAR PURPOSE. See the GNU General Public License for more details.

You should have received a copy of the GNU General Public License along with this program. If not, see <http://www.gnu.org/licenses/>.

```
*/

package aggregationtool;

import java.util.Date;
import org.w3c.dom.Document;
import org.w3c.dom.Element;
import org.w3c.dom.NodeList;


public class HeaderEntry extends DataEntry {

    //timeunit which defines the unit of the start- and endtime of the entries; 1.0 is one second
    public float timeUnit;
    //last modified date of the read file
    public Date lastModified;
    //Name of the read file
    public String fileName;

    public HeaderEntry() {

    }

    public HeaderEntry(float TimeUnit, Date LastModified, String FileName) {
        this.timeUnit=TimeUnit;
        this.lastModified=LastModified;
        this.fileName=FileName;
    }

    public String toString() {
        return "Filename: "+fileName+" TimeUnit: "+timeUnit+" LastModified: "+lastModified;
    }

    /**
     * this method compares two HeaderEntries
     * - 1) Filename
     * - 2) lastModified
```

```
     * - 3) timeUnit
     *
     * @param he2
     * @return
     */
    public int compareTo(HeaderEntry he2) {

        if (this.fileName.equals(he2.fileName)) {
            if (this.lastModified.equals(he2.lastModified)) {
                return Float.compare(this.timeUnit,he2.timeUnit);
            }
            else {
                return this.lastModified.compareTo(he2.lastModified);
            }
        }
        else {
            return this.fileName.compareTo(he2.fileName);
        }
    }

    /**
     * return values of variables
     * must have same order like itemNames
     * @return all items in an object array
     */
    public Object[] itemsToDisplaystyle() {
        Object [] array = new Object[3];
        array[0]= fileName;
        array[1]= lastModified;
        array[2]= new Float(timeUnit);

        return array;
    }

    /**
     * Return strings with names of all variables
     * must have same order like itemsToDisplaysytle
     * @return the names of the items in an object array
     */
    public static Object[] itemDisplayNames() {
        return new String[] {"Filename","Last modified","Timeunit"};
    }

    //***************************************************************************
    //************************* XML functionality ****************************
    //***************************************************************************

    /**
     * create Element with content of all information to be displayed in XML file
     * @param document to create elements
     * @return class element with all added information
     */
    public Element createXMLElement(Document document) {
```

```
        Element classElement = document.createElement(this.getClass().getSimpleName());

        //timeUnit
        Element elemTimeUnit = document.createElement("time_unit");
        elemTimeUnit.appendChild(document.createTextNode(""+timeUnit));
        classElement.appendChild(elemTimeUnit);

        //lastModified
        Element elemLastModified = document.createElement("last_modified");
        elemLastModified.appendChild(document.createTextNode(lastModified.toString()));
        classElement.appendChild(elemLastModified);

        //fileName
        Element elemFileName = document.createElement("file_name");
        elemFileName.appendChild(document.createTextNode(fileName));
        classElement.appendChild(elemFileName);

        return classElement;
    }

    /**
     * Method to set settings by an given element of an xml file
     * !!! attention, read interface to get further information !!!
     * @param classElement xml element with data source
     */
     public void setPropertiesByXML(Element classElement) {

         //timeunit
         NodeList timeUnitList = classElement.getElementsByTagName("time_unit");
         this.timeUnit =
Float.parseFloat(timeUnitList.item(0).getChildNodes().item(0).getNodeValue());

         //Last modified
         lastModified = new Date(System.currentTimeMillis());

         //file name
         NodeList filenameL = classElement.getElementsByTagName("file_name");
         fileName = filenameL.item(0).getChildNodes().item(0).getNodeValue();
     }
}
```

Src 17 : Histogram.java

/**
Displays occurrence of eventvalues either only by occurrence or including duration too
Log File Aggregation (LFA) tool V1.1.

Copyright (C) 2008 Biomedical Informatics (BMI), St George's University of London. Citations to the toolkit should be as 'de Lusignan S, Kumarapeli P, Chan T, Pflug B, van Vlymen J, Jones B, Freeman GK. The ALFA (activity Log Files Aggregation) toolkit: a method for precise observation of the consultation. <J Med Internet Res 2008; ..>.

This program is free software: you can redistribute it and/or modify it under the terms of the GNU General Public License as published by the Free Software Foundation, either version 3 of the License, or (at your option) any later version.

This program is distributed in the hope that it will be useful, but WITHOUT ANY WARRANTY; without even the implied warranty of MERCHANTABILITY or FITNESS FOR A PARTICULAR PURPOSE. See the GNU General Public License for more details.

You should have received a copy of the GNU General Public License along with this program. If not, see <http://www.gnu.org/licenses/>.

```
*/

package aggregationtool;

import java.awt.Color;
import java.awt.Component;
import java.awt.Dimension;
import java.awt.Font;
import java.awt.GridBagConstraints;

import java.awt.Insets;

import java.awt.Rectangle;
import java.awt.event.ActionEvent;
import java.awt.event.ActionListener;

import java.io.File;
import java.io.IOException;

import java.util.ArrayList;

import javax.swing.BorderFactory;
import javax.swing.Icon;
import javax.swing.ImageIcon;
import javax.swing.JButton;
import javax.swing.JColorChooser;
import javax.swing.JComboBox;
import javax.swing.JFileChooser;
import javax.swing.JLabel;
import javax.swing.JOptionPane;
import javax.swing.JPanel;
import javax.swing.JSeparator;
import javax.swing.SwingConstants;

public class Histogram extends AbstractGraphDisplay {

    //**********************
    //**** Data Sources ****
    //**********************

    //Arraylist containing all dataentries for this view
```

```
private ArrayList Source;

//defines the columns for display
private Object [] UniqueValues;

//defines the length of the plot depending on the occurrence of a value and the option
private int [] valueCount;
private int [] valueLength;

//****************************
//** coordinates of panels **
//****************************

//distance from Top to first panel
private int topBorder = 20;
//distance from panels to left border
private int leftBorder = 30;
//distance between panels
private int panelBorder = 10;

//****************************
//****** size of panels ******
//****************************
//define the minimum Coordinations for the whole panel
//private int minimumDiagramX, minimumDiagramY;

private int headerWidth = 80;
private int headerHeight = 30;
private int minOptionWidth = 490;
//calculated - depend on content
private int optionWidth;
private int optionHeight = 40;
//calculated - depend on Content
private int diagramWidth;
//calculated - depend on Content
private int diagramHeight;
//distances between Panel border and diagram content
private int diagramHorizontalBorder=40;
private int diagramVerticalBorder= 50;

//****************************
//****** current panels ******
//****************************

 JPanel headerPanel;
 JPanel optionPanel;
 JPanel diagramPanel;

//***********************
//*** Layout Settings ***
//***********************

//*** Combo Box ***
```

```
private static String ONLY_COUNT = "only count";
private static String INC_DURATION = "including duration";
//defines if only number of occurrence is displayed or duration as well
private String countingOption = ONLY_COUNT;

//Zoom faktor
private float ZF=1;

//Distance between two seperators
private int sepDistance=20;

//max size of one charakter
private int charakterPixel=12;

//defines the highest value for each list
//necessary for defining Y size of window
private int highestCountValue,highestLengthValue;

//distance between two values on x koordinate
private int ValueDistance=40;
private int actualValueDistance;

//*** en-disabled items ***
//horizontal separator and labels
private boolean h_separator=true;
//vertical separator
private boolean v_separator=true;

//**** Colors ****
//horizontal separator color
private static Color default_hs_color = new Color(113, 32, 60);
private Color hs_color;
//vertical separator color;
private static Color default_vs_color = Color.gray;
private Color vs_color;

public Histogram(GraphDisplayDialog gdd,ArrayList Source) {
    super(gdd);

    this.Source = Source;

    //get unique Values for labeling
     UniqueValues = DataEntry.getUniqueEventValues(Source);

    //only called once in constructor to create display information from Source
    analyseData();

    //updates calculated variables
    updateCalculatedVariables();

    createLayout();
}
```

```
    /**
     * This method creates the layout of the whole window
     */
    public void createLayout() {
        this.setLayout( null );

        headerPanel = createHeader();
        optionPanel = createOptionPane();
        diagramPanel = createDiagram();

        //swap diagram
        this.swapYKoord(diagramPanel);

        this.add(headerPanel);
        this.add(optionPanel);
        this.add(diagramPanel);
    }

    /**
     * @return panel for headerinformation
     */
    public JPanel createHeader() {
        JPanel headerPanel = new JPanel();
        headerPanel.setLayout( null );
        headerPanel.setBounds(leftBorder,topBorder,headerWidth,headerHeight);

        JLabel headerLabel = new
JLabel(((EventEntry)Source.get(0)).getEvent().getType().toString());
        headerLabel.setFont(new Font("header",3,15));
        headerLabel.setBounds(0,0,70,30);

        headerPanel.add(headerLabel);

        return headerPanel;
    }

    /**
     * @return panel for option pane
     */
    public JPanel createOptionPane() {
        JPanel optionPanel = new JPanel();
        optionPanel.setLayout( null );
        optionPanel.setBorder(BorderFactory.createLineBorder(Color.gray,1));

optionPanel.setBounds(leftBorder,topBorder+headerHeight+panelBorder,optionWidth,optionHeig
ht);


        //*** Label ***
        JLabel label = new JLabel("OPTIONS");
        label.setFont(new Font("options",3,10));
        label.setBounds(5,7,50,25);
        optionPanel.add(label);
```

```
//*** Zoom ***
//ZoomIn
Icon icon = new ImageIcon ("OptionIcons\\lupe_plus_20.jpg");
JButton ZoomIn = new JButton(icon);
ZoomIn.setBounds(60,7,25,25);
ZoomIn.setToolTipText("Zoom in");
ZoomIn.addActionListener( new ActionListener() {
    public void actionPerformed(ActionEvent e) {
        ZF*=2;
        updateContent();
    }
}
);
optionPanel.add(ZoomIn);

//ZoomOut
icon = new ImageIcon ("OptionIcons\\lupe_minus_20.jpg");
JButton ZoomOut = new JButton(icon);
ZoomOut.setBounds(90,7,25,25);
ZoomOut.setToolTipText("Zoom out");
ZoomOut.addActionListener( new ActionListener() {
    public void actionPerformed(ActionEvent e) {
        ZF/=2;
        updateContent();
    }
}
);
optionPanel.add(ZoomOut);

//ZoomLabel
JLabel zoomLabel = new JLabel(""+ZF);
zoomLabel.setFont(new Font("zooml",3,11));
zoomLabel.setBounds(125,7,25,25);
optionPanel.add(zoomLabel);

//*** Enable - Disable ***
//En- Disable vertical vertical lines
 icon = new ImageIcon ("OptionIcons\\vertical.jpg");
 JButton VerticalLines = new JButton(icon);
 VerticalLines.setBounds(165,7,25,25);
 VerticalLines.setToolTipText("En/Disable vertical lines");
 VerticalLines.addActionListener( new ActionListener() {
     public void actionPerformed(ActionEvent e) {
         v_separator = !v_separator;
         updateContent();
     }
 }
 );
 optionPanel.add(VerticalLines);

//En- Disable horizontal lines
 icon = new ImageIcon ("OptionIcons\\horizontal.jpg");
```

```
        JButton HorizontalLines = new JButton(icon);
        HorizontalLines.setBounds(195,7,25,25);
        HorizontalLines.setToolTipText("En/Disable horizontal lines");
        HorizontalLines.addActionListener( new ActionListener() {
            public void actionPerformed(ActionEvent e) {
                h_separator = !h_separator;
                updateContent();
            }
        }
        );
        optionPanel.add(HorizontalLines);

       //*** Change Colors ***
       //horizontal Color change
       icon = new ImageIcon ("OptionIcons\\horizontal_color.jpg");
       JButton HorizontalColor = new JButton(icon);
       HorizontalColor.setBounds(235,7,25,25);
       HorizontalColor.setToolTipText("Change horizontal lines color");
       HorizontalColor.addActionListener( new ActionListener() {
          public void actionPerformed(ActionEvent e) {
              hs_color = JColorChooser.showDialog(null,"Choose color for horizontal
lines",default_hs_color);
              updateContent();
          }
       }
       );
       optionPanel.add(HorizontalColor);

       //horizontal Color change
       icon = new ImageIcon ("OptionIcons\\vertical_color.jpg");
       JButton VerticalColor = new JButton(icon);
       VerticalColor.setBounds(265,7,25,25);
       VerticalColor.setToolTipText("Change vertical lines color");
       VerticalColor.addActionListener( new ActionListener() {
          public void actionPerformed(ActionEvent e) {
              hs_color = JColorChooser.showDialog(null,"Choose color for vertical
lines",default_hs_color);
              updateContent();
          }
       }
       );
       optionPanel.add(VerticalColor);

       //save to jpeg
       icon = new ImageIcon("OptionIcons\\jpeg_icon.jpg");
       JButton SaveJpeg = new JButton(icon);
       SaveJpeg.setBounds(310,7,25,25);
       SaveJpeg.setToolTipText("Save diagram as jpg");
       SaveJpeg.addActionListener( new ActionListener () {
          public void actionPerformed(ActionEvent e) {

              //create save file dialog with embedded filter for jpeg files
              JFileChooser fc = new JFileChooser();
```

```
        if (LogAggregationTool.isFileDirSet()) {
            fc.setCurrentDirectory(new File(LogAggregationTool.getFileDir()));
        }

        fc.setDialogTitle("select destination ...");

        int returnVal = fc.showSaveDialog(gdd.superclass);

        if (returnVal == JFileChooser.APPROVE_OPTION) {
            File file = fc.getSelectedFile();

            String filePath = file.getAbsolutePath();
            String fileName = file.getName();

            boolean flag1 = true;

            //Check if file extists, and in case ask for overwrite
            if (file.exists()) {
                int returnVal2 = JOptionPane.showConfirmDialog(null,"Overwrite existing
file?","Overwrite",JOptionPane.YES_NO_OPTION);

                if (returnVal2 != JOptionPane.YES_OPTION) {
                    flag1 = false;
                    return;
                }
            }
            else {
                //in case file not exists, check for extention
                //In case no extention given, create standard
                if (fileName.lastIndexOf(".") == -1) {
                    filePath += ".jpg";
                    fileName += ".jpg";
                }
            }

            if (flag1) {

                if (fileName.lastIndexOf(".")==0) {
                    JOptionPane.showMessageDialog(gdd.superclass,"Not allowed to enter empty
file name","Error",JOptionPane.ERROR_MESSAGE);
                    return;
                }


                //check for equal extention of type
                if (fileName.indexOf(".") !=-1 &&
fileName.substring(fileName.lastIndexOf(".")).equalsIgnoreCase(".jpg")) {

                    //process output with correct extention
                    try {
                        ScreenImage.createImage(diagramPanel,filePath);
```

```
                    JOptionPane.showMessageDialog(gdd.superclass,"Saving
sucessfull!","INFO",JOptionPane.INFORMATION_MESSAGE);
                  }
                  catch (IOException ex) {
                    JOptionPane.showMessageDialog(gdd.superclass,"Error creating
image!","Error",JOptionPane.ERROR_MESSAGE);
                  }
                }
                else {
                  JOptionPane.showMessageDialog(gdd.superclass,"Only extention '.jpg'
allowed!","Error",JOptionPane.ERROR_MESSAGE);
                }
              }

            }

            //set global file dir
            LogAggregationTool.setFileDir(fc.getCurrentDirectory().getPath());
          }
        });
        optionPanel.add(SaveJpeg);

        //*** Combo box ***
        JComboBox displayType = new JComboBox(new Object[]
{ONLY_COUNT,INC_DURATION});
        displayType.setBounds(350,7,135,25);
        displayType.setSelectedItem(countingOption);
        displayType.addActionListener( new ActionListener() {
          public void actionPerformed(ActionEvent e) {
            countingOption = (String)((JComboBox)e.getSource()).getSelectedItem();
            updateContent();
          }
        }
        );
        optionPanel.add(displayType);


        return optionPanel;
    }

    /**
     * @return panel with diagramm
     */
    public JPanel createDiagram() {
        JPanel diagramPanel = new JPanel();
        diagramPanel.setBorder(BorderFactory.createLineBorder(Color.GRAY,1));
        diagramPanel.setLayout( null );

diagramPanel.setBounds(leftBorder,topBorder+headerHeight+panelBorder+optionHeight+panelB
order,diagramWidth,diagramHeight);

        //***********************************************************************
        //create Labels for displayed values
```

```
    //*****************************************************************

     for (int i =0; i< UniqueValues.length; i++) {
        JLabel label = new JLabel((String)UniqueValues[i]);
        int XValue = diagramHorizontalBorder+i*actualValueDistance;
        float offset = (actualValueDistance-charakterPixel*((String)UniqueValues[i]).length())/2;

        //In case label length is not larger then Valuedistance, center
        if (offset > 0) {
           XValue += (int)offset;
        }


label.setBounds((int)XValue,diagramVerticalBorder/5*3,charakterPixel*((String)UniqueValues[i]).l
ength(),15);

        diagramPanel.add(label);
     }

    //*****************************************************************
    //create lines depending on option
    //*****************************************************************

     //after counting draw buttons with spezified length
     JButton button;

     //can use for both the same length as they always have the same length
     for (int i=0; i < valueCount.length; i++) {
        button = new JButton();

        if (countingOption.equals(ONLY_COUNT)) {
           button.setBounds((int)(i*actualValueDistance + actualValueDistance/10 +
diagramHorizontalBorder),diagramVerticalBorder,actualValueDistance/10*8,(int)(valueCount[i]*Z
F));

           button.setToolTipText(""+valueCount[i]);
        }
        else if (countingOption.equals(INC_DURATION)) {
           button.setBounds((int)(i*actualValueDistance + actualValueDistance/10 +
diagramHorizontalBorder),diagramVerticalBorder,actualValueDistance/10*8,(int)(valueLength[i]*Z
F));

           button.setToolTipText(""+valueLength[i]);
        }

        diagramPanel.add(button);
     }

    //*****************************************************************
    //create X and Y Koordinates
    //*****************************************************************

    //horizontal Zeroline
```

```
        JSeparator horZeroline = new JSeparator();
        horZeroline.setBounds(diagramHorizontalBorder,diagramVerticalBorder-1,diagramWidth-
diagramHorizontalBorder*2,2);
        horZeroline.setBackground(Color.gray);
        diagramPanel.add(horZeroline);

        //ZeroLabel
        JLabel zeroValueLabel = new JLabel("0");
        zeroValueLabel.setFont(new Font("labeling",1,10));
        zeroValueLabel.setBounds(diagramHorizontalBorder/5*3,diagramVerticalBorder-11,20,20);
        diagramPanel.add(zeroValueLabel);

        //vertical Zeroline
        JSeparator verZeroline = new JSeparator(JSeparator.VERTICAL);
        verZeroline.setBounds(diagramHorizontalBorder-1,diagramVerticalBorder,2,diagramHeight-
diagramVerticalBorder*2);
        verZeroline.setBackground(Color.gray);
        diagramPanel.add(verZeroline);

        //create vertical separators between values
        if (v_separator) {

            for (int i = 0; i< UniqueValues.length; i++) {
                JSeparator v_sep = new JSeparator(JSeparator.VERTICAL);

v_sep.setBounds(diagramHorizontalBorder+(i+1)*(int)actualValueDistance,diagramVerticalBorder
,1,diagramHeight-diagramVerticalBorder*2);

                if (vs_color != null) {
                    v_sep.setForeground(vs_color);
                }
                else {
                    v_sep.setForeground(default_vs_color);
                }

                diagramPanel.add(v_sep);
            }
        }

        if (h_separator) {

            //Counts esspecially the number displayed on the labels
            float labelCounter = 0;

            for (float i = diagramVerticalBorder+sepDistance; i<= diagramHeight-
diagramVerticalBorder; i+=sepDistance) {

                //inclease labelCounter
                labelCounter += sepDistance / ZF;

                //*** separator ***
                JSeparator h_sep = new JSeparator();
```

```
            h_sep.setBounds(diagramHorizontalBorder,(int)i,diagramWidth-
diagramHorizontalBorder*2,1);


            //*** label ***
            JLabel h_label;
            String disValue = ""+labelCounter;
            int pointIndex = disValue.indexOf(".");
            //If . found (means number is float) and there are more then two decial placed, cut at
this pos
            if (pointIndex != -1 && disValue.length() > pointIndex+2) {
               disValue = disValue.substring(0,pointIndex+3);
            }

            h_label = new JLabel(disValue);
            h_label.setHorizontalAlignment(SwingConstants.RIGHT);
            h_label.setFont(new Font("labeling",1,10));
            h_label.setBounds(0,(int)i-11,diagramHorizontalBorder-2,20);

            //set colors
            if (hs_color != null) {
               h_sep.setBackground(hs_color);
               h_label.setForeground(hs_color);
            }
            else {
               h_sep.setBackground(default_hs_color);
               h_label.setForeground(default_hs_color);
            }

            diagramPanel.add(h_sep);
            diagramPanel.add(h_label);
         }
      }

       return diagramPanel;
   }

   //**************************************************************************
   //*********** Methods for calculation and repainting **********************
   //**************************************************************************

   /**
    * in case of changed options some variables must be new calculated
    */
   private void updateCalculatedVariables() {
      //calculate actual Value distance by set value multi. with Zoom faktor
      //TODO if also horizontal zoom is decired, multiplicate with ZF
      actualValueDistance = ValueDistance;

      //check if one header value is longer then actualValueDistance
      for (int i = 0; i< UniqueValues.length; i++) {
         if (UniqueValues[i].toString().length() * charakterPixel > actualValueDistance ) {
            //in that case set value distance as length of string * number of pixel of one charakter
```

```
                actualValueDistance = UniqueValues[i].toString().length() * charakterPixel;
            }
        }

        //set option width like diagram width (so border is shown in better way)
        optionWidth = (diagramHorizontalBorder*2 + UniqueValues.length*actualValueDistance);

        //in case of zoom out or removing diagram can become smaler then minimum option length
        if (optionWidth < minOptionWidth) {
            optionWidth = minOptionWidth;
        }

        //diagram widht is defined as horizontal border on left and right and number of values *
actual value distance
        diagramWidth = (diagramHorizontalBorder*2 + UniqueValues.length*actualValueDistance);

        if (countingOption.equals(ONLY_COUNT)) {
            diagramHeight = (int)(diagramVerticalBorder*2 + highestCountValue*ZF);
        }
        else if (countingOption.equals(INC_DURATION)) {
            diagramHeight = (int)(diagramVerticalBorder*2 + highestLengthValue*ZF);
        }

    }
    /**
     * Is called if compenents have changed to repaint window
     */
    public void updateContent() {
        this.removeAll();

        this.updateCalculatedVariables();;

        this.createLayout();

        this.repaint();

        this.windowChanged();
    }

    public Dimension getMinimumDisplaySize() {

        Dimension minDim = new Dimension();

        //if option pane width higher then diagramwidth, set optionwidth, otherwise diagramwidth
        if (optionWidth > diagramWidth) {
           minDim.width = leftBorder*2 + optionWidth;
        }
        else {
            minDim.width = leftBorder*2 + diagramWidth;
        }

        minDim.height = 2 * topBorder + 2 * panelBorder + headerHeight + optionHeight +
diagramHeight;
```

```
        return minDim;
    }

    /**
     * swaps all koordinations in Y direcation on the given value
     * @param panel where all components are translated
     */
    private void swapYKoord(JPanel panel) {
        //get Height of given panel
        Dimension panelSize = panel.getSize();
        double height = panelSize.getHeight();

        //go through all components in panel
        for (int i =0; i < panel.getComponentCount(); i++) {
            Component com = panel.getComponent(i);

            Rectangle rec = com.getBounds();

            int Ykoord = (int)rec.getMinY();
            int Ylength = (int)(rec.getMaxY()-rec.getMinY());

            int newY = (int)height - (Ykoord + Ylength);

            com.setBounds((int)rec.getMinX(),newY,(int)(rec.getMaxX()-
rec.getMinX()),(int)(rec.getMaxY()-rec.getMinY()));
        }

        repaint();
    }


    /**
     * method to create display info from Source data
     * for each option its own list with content data is created
     */
    private void analyseData() {
        valueCount = new int [UniqueValues.length];
        valueLength = new int [UniqueValues.length];

        //For each Entry in Source draw Rectange
        for (int i=0; i < Source.size(); i++) {
            EventEntry ee = (EventEntry) Source.get(i);

            //Compare entry with all unique Values, in case of match draw at position
            for (int j=0; j< UniqueValues.length; j++) {
                if (UniqueValues[j].equals(ee.getEvent().getValue().toString())) {

                    //only count number of values for option ONLY_COUNT
                    valueCount[j]+= 1;

                    //upate hightest Count value
                    if (valueCount[j] > highestCountValue) {
```

```
                highestCountValue = valueCount[j];
              }

              //Include duration for option INC_DURATION
              if (!ee.isDuration()) {
                valueLength[j] += 1;
              }
              else {
                valueLength[j] += Math.round(ee.EndTime-ee.StartTime);
              }

              if (valueLength[j] > highestLengthValue) {
                highestLengthValue = valueLength[j];
              }

            }
          }

          System.out.println("ValueCount:"+valueCount[2]+" ValueLength:"+valueLength[2]);
        }
    }

    //***************************************************************************
    /**
     * creates the settings for a component which should be displayed in GridBagLayout
     * @param x startcoordinate of component
     * @param y startcoordinate of component
     * @param width how many cells
     * @param height how many cells
     * @return
     */
    private GridBagConstraints makegbc (int x, int y, int width, int height,int fill, int ipadx, int
ipady) {
        GridBagConstraints gbc = new GridBagConstraints();
        gbc.gridx = x;
        gbc.gridy = y;
        gbc.gridwidth = width;
        gbc.gridheight = height;
        gbc.fill = fill;
        gbc.ipadx = ipadx;
        gbc.ipady = ipady;
        //Defines the border of each element
        gbc.insets = new Insets(5, 5, 1, 1);

        return gbc;
    }
}
```

Src 18: LogAggregator.java

```
/**
class to aggregate converted files to one single aggregation file
Log File Aggregation (LFA) tool V1.1.

Copyright (C) 2008 Biomedical Informatics (BMI), St George's University of London. Citations to
the toolkit should be as 'de Lusignan S, Kumarapeli P, Chan T, Pflug B, van Vlymen J, Jones B,
Freeman GK. The ALFA (activity Log Files Aggregation) toolkit: a method for precise observation
of the consultation. <J Med Internet Res 2008; ..>.

This program is free software: you can redistribute it and/or modify it under the terms of the
GNU General Public License as published by the Free Software Foundation, either version 3 of the
License, or (at your option) any later version.

This program is distributed in the hope that it will be useful, but WITHOUT ANY WARRANTY;
without even the implied warranty of MERCHANTABILITY or FITNESS FOR A PARTICULAR
PURPOSE.  See the GNU General Public License for more details.

You should have received a copy of the GNU General Public License along with this program.  If
not, see <http://www.gnu.org/licenses/>.

*/

package aggregationtool;

import java.util.ArrayList;
import java.util.Collections;
import java.util.Iterator;


public class LogAggregator {

    //Arraylist containting all converted files choosen at conversion
    private ArrayList commonFiles;


    //************************************************************************
    //******************* constructor and set method **************************
    //************************************************************************
    public LogAggregator(ArrayList commonFiles) {
        this.commonFiles = commonFiles;
    }

    /**
     * Method to set common file data source
     * @param commonFiles Arraylist containing ArrayLists with DataEntries of each converted file
     */
    public void setCommonFiles(ArrayList commonFiles) {
        this.commonFiles = commonFiles;
    }


    //************************************************************************
    //********************** process method **********************************
```

```
    //**************************************************************************
    /**
     * Method to combine all content of given files from conversion
     * @return arraylist with aggregated data
     */
    public ArrayList process_aggregation() {

        ArrayList aggregationResult = new ArrayList();

        //if more then one file is choosen, start aggregation
        if (commonFiles.size()>1) {
            Iterator it = commonFiles.iterator();

            //Put the whole information of all files together
            while (it.hasNext()) {
                aggregationResult.addAll((ArrayList)it.next());
            }

            //Sort this information as defined in compareTo function in class
            Collections.sort(aggregationResult);
        }
        else {
            //if there is only one file, the output is equal to the input
            aggregationResult.addAll((ArrayList)commonFiles.get(0));
        }

        return aggregationResult;
    }
}
```

Src 19 : LogConverter.java

```
/**
Superclass for all types of log convertes
Log File Aggregation (LFA) tool V1.1.

Copyright (C) 2008 Biomedical Informatics (BMI), St George's University of London. Citations to
the toolkit should be as 'de Lusignan S, Kumarapeli P, Chan T, Pflug B, van Vlymen J, Jones B,
Freeman GK. The ALFA (activity Log Files Aggregation) toolkit: a method for precise observation
of the consultation. <J Med Internet Res 2008; ..>.

This program is free software: you can redistribute it and/or modify it under the terms of the
GNU General Public License as published by the Free Software Foundation, either version 3 of the
License, or (at your option) any later version.

This program is distributed in the hope that it will be useful, but WITHOUT ANY WARRANTY;
without even the implied warranty of MERCHANTABILITY or FITNESS FOR A PARTICULAR
PURPOSE.  See the GNU General Public License for more details.

You should have received a copy of the GNU General Public License along with this program.  If
not, see <http://www.gnu.org/licenses/>.
```

```
*/

package aggregationtool;

import java.awt.BorderLayout;
import java.awt.Font;

import java.io.BufferedReader;
import java.io.File;
import java.io.FileNotFoundException;
import java.io.FileReader;
import java.io.IOException;

import java.util.ArrayList;
import java.util.Iterator;

import javax.swing.JLabel;
import javax.swing.JPanel;

/**
 *
 * HOW TO ADD NEW LOG_CONVERTER SUBCLASSES
 *
 * following steps are necessary to add new subclass:
 * - create class implementing LogConverter
 * - add static string EVENTTYPE to LogConverter
 * - CLASS ConversionPanel
 * -- add EVENTTYPE to combobox for choose
 * -- add code in set converter method to create new instance of subclass
 *
 */
public abstract class LogConverter {

    //static definitions for Eventtypes for all subclasses
    //!!! MUST BE UNIQUE !!!
    public static String OBSWINEVENTTYPE = "OBSWIN";
    public static String UAREVENTTYPE = "UAR";
    public static String PRSEVENTTYPE = "PRS";

    public static String XMLFILETYPE = "XML";
    public static String CSVFILETYPE = "CSV";

    //global defined timeunit IN SECONDS
    public static float GLOBAL_TIMEUNIT = 1.0F;

    //variables to manage content and error
    protected ArrayList FileContent,CommonContent;
    protected ArrayList errorsWarnings;

    //variables for file read in
    private BufferedReader bf;
    protected String FilePath;
```

```
protected String FileName;
protected long LastModified;
private String line;

//variables for property panel

protected JPanel propertyPanel;

//Define Height and Width of property panel
public static int PROPERTY_PANEL_WIDTH = 400;
public static int PROPERTY_PANEL_HEIGHT = 200;

//**************************************************************************
//****************************** constructor  ******************************
//**************************************************************************

public LogConverter () {

}

//**************************************************************************
//****************************** accessors *********************************
//**************************************************************************

/**
 * set method for file path
 * @param filePath
 */
public void setFilePath(String filePath) {
    this.FilePath = filePath;
}

//**************************************************************************
//************************** static methods ********************************
//**************************************************************************

//--------

//**************************************************************************
//************************** abstract methods ******************************
//**************************************************************************

 /**
  * This method is for creating the common log format from the content of the file
  * it should be implemented in each subclass for it's special format
  * @return ArrayList with all warnings at conversion
  */
 protected abstract void interpretLogFile();

/**
 * This method should be implemented to create the property panel for display
 * in case new property panel is required, leave method skeleton without body
 */
```

```
protected abstract void createPropertyPanel();

//***************************************************************************
//************************ implemented methods *****************************
//***************************************************************************

 /**
  * creates JPanel in prefered size of 400/200 which contains all required
  * properties of each output type
  * in case instance is null create default panel with no information
  * @return JPanel with required propertie content
  */
 public JPanel getPropertyPanel() {

    //first call property panel method implemented in each subclass
    createPropertyPanel();

    //after that check if method was implemented
    if (propertyPanel != null) {
        //set panel size to default size
        propertyPanel.setSize(PROPERTY_PANEL_WIDTH,PROPERTY_PANEL_HEIGHT);

        return propertyPanel;
    }
    else {
        //In case no panel is set by subclass default panel is created
        JPanel defaultPanel = new JPanel();
        defaultPanel.setSize(PROPERTY_PANEL_WIDTH,PROPERTY_PANEL_HEIGHT);
        defaultPanel.setLayout(new BorderLayout());
        JLabel defaultLabel = new JLabel("<no properties available for this type>");
        defaultLabel.setFont(new Font("TIMES NEW ROMAN",Font.ITALIC,15));
        defaultPanel.add(defaultLabel,BorderLayout.CENTER);
        return defaultPanel;
    }
 }

/**
 * This method reads the log-file line by line, and saves the content into an arraylist
 */
protected void readLogFile() {

    errorsWarnings = new ArrayList();

    try {

        bf= new BufferedReader(new FileReader(FilePath));

        //get name of the file
        File file = new File(FilePath);
        FileName = file.getName();
        LastModified = file.lastModified();

        FileContent = new ArrayList();
```

```
            while ((line = bf.readLine()) != null) {
                FileContent.add(line);
            }

            //if file content is empty, error
            if (FileContent.isEmpty()) {
                addErrorWarning(ErrorDialog.ERROR,"Empty file!");
            }
        }
        catch (FileNotFoundException ex) {
            addErrorWarning(ErrorDialog.ERROR,"File not found!");
        }
        catch (IOException ex2) {
            addErrorWarning(ErrorDialog.ERROR,"Error reading line!");
        }
    }

    /**
     * This method combines the reading and the interpreting of the logFile
     * and standardise time unit
     * This method should be called from outside
     * @return ArrayList with common Data
     */
    public ArrayList getDataFromFile() {

        readLogFile();

        if (!errDialogEntry.containsErrors(errorsWarnings)) {
            this.interpretLogFile();

            if (!errDialogEntry.containsErrors(errorsWarnings)) {
                //in case no standard timeunit is required only remove this method here
                this.standardiseTimeUnit();
            }
        }

        return this.CommonContent;
    }

    public ArrayList getFileContent() {
        return this.FileContent;
    }

    public ArrayList getCommonContent() {
        return this.CommonContent;
    }

    //***************************************************************************
    //*********************** timeunit handling *********************************
    //***************************************************************************

    /**
```

```
     * Method to convert all time entries to the global timeunit
     * Must be called after each read in to get same timeformat for all files
     */
    public void standardiseTimeUnit() {

        //get header entry
        HeaderEntry headerE = null;

        //get headerentry as first entry of arrarylist, otherwise create errormessage and return
        //!!! always first headerentry is selected
        if (CommonContent.size()>0 && CommonContent.get(0) instanceof HeaderEntry) {
            headerE = (HeaderEntry)CommonContent.get(0);
        }
        else {
            addErrorWarning(ErrorDialog.ERROR,"Unable to convert to global timeunit, no
headerentry found");
            return;
        }

        //define value entries must be multiplied with
        float divValue = headerE.timeUnit/GLOBAL_TIMEUNIT;

        //change timeunit in header to global time unit
        headerE.timeUnit = GLOBAL_TIMEUNIT;

        //go through event entries and change values
        Iterator it = CommonContent.iterator();

        while (it.hasNext()) {
            DataEntry de = (DataEntry)it.next();

            if (de instanceof EventEntry) {
                EventEntry ee = (EventEntry)de;

                //multiply start- and endtime with calculated divValue
                ee.StartTime *= divValue;

                //check whether Entry is no duration
                if (ee.isDuration()) {
                    ee.EndTime *= divValue;
                }
            }
        }
    }

    //***************************************************************************
    //********************** error handling ************************************
    //***************************************************************************

    /**
     * Method to add new error or warning to Error-Warnings List
     * @param type Error or Warning
     * @param text to display
```

```
     */
    public void addErrorWarning(int type, String text) {
        errorsWarnings.add(new errDialogEntry(type,text));
    }

    /**
     * @return Arraylist with errDialogEntries with all errors and warnings occurred
     */
    public ArrayList getErrorWarnings() {
        return errorsWarnings;
    }
}
```

Src 20 : newColumnGraphPanel.java

```
/**
Creats an panel containing column based output for an arraylist of Data entries
Log File Aggregation (LFA) tool V1.1.

Copyright (C) 2008 Biomedical Informatics (BMI), St George's University of London. Citations to
the toolkit should be as 'de Lusignan S, Kumarapeli P, Chan T, Pflug B, van Vlymen J, Jones B,
Freeman GK. The ALFA (activity Log Files Aggregation) toolkit: a method for precise observation
of the consultation. <J Med Internet Res 2008; ..>.

This program is free software: you can redistribute it and/or modify it under the terms of the
GNU General Public License as published by the Free Software Foundation, either version 3 of the
License, or (at your option) any later version.

This program is distributed in the hope that it will be useful, but WITHOUT ANY WARRANTY;
without even the implied warranty of MERCHANTABILITY or FITNESS FOR A PARTICULAR
PURPOSE.  See the GNU General Public License for more details.

You should have received a copy of the GNU General Public License along with this program.  If
not, see <http://www.gnu.org/licenses/>.

*/


package aggregationtool;

import java.awt.Color;

import java.awt.Dimension;
import java.awt.Font;
import java.awt.event.ActionEvent;
import java.awt.event.ActionListener;

import java.io.File;

import java.io.IOException;
```

```
import java.util.ArrayList;

import javax.swing.BorderFactory;
import javax.swing.Icon;
import javax.swing.ImageIcon;
import javax.swing.JButton;
import javax.swing.JColorChooser;
import javax.swing.JComboBox;
import javax.swing.JFileChooser;
import javax.swing.JLabel;
import javax.swing.JOptionPane;
import javax.swing.JPanel;
import javax.swing.JSeparator;
import javax.swing.SwingConstants;

public class newColumnGraphPanel extends AbstractGraphDisplay{

    //****************************
    //******* data source ********
    //****************************

    //Arraylist with Data Source for display
    ArrayList Source;

    //defines the columns for display
    private Object [] UniqueValues;
    private Object [] visibleUniqueValues;

    //saves the highest starttime and highest endtime of data source
    private float highestST,highestET;

    //****************************
    //** coordinates of panels **
    //****************************

    //distance from Top to first panel
    private int topBorder = 20;
    //distance from panels to left border
    private int leftBorder = 30;
    //distance between panels
    private int panelBorder = 10;

    //****************************
    //****** size of panels ******
    //****************************
    private int minHeaderWidth=170;
    //calculated - depend on Content
    private int headerWidth;
    private int headerHeight = 40;
    private int minOptionWidth = 430;
    //calculated - depend on content
    private int optionWidth;
    private int optionHeight = 40;
```

```
//calculated - depend on Content
private int diagramWidth;
//calculated - depend on Content
private int diagramHeight;
//distances between Panel border and diagram content
private int diagramHorizontalBorder=40;
private int diagramVerticalBorder= 40;

//****************************
//****** current panels ******
//****************************

 JPanel headerPanel;
 JPanel optionPanel;
 JPanel diagramPanel;

//****************************
//*** settings for display ***
//****************************

//Zoom faktor
private float ZF = 1;
//Distance between two columns
private int valueDistance = 40;
//-- calculated value
private float actualValueDistance;
//Distance between separators
private int sepDistance = 50;
//-- calculated value
private float actualSepDistance;

//number of pixel a charakter of the label has (approximated)
private int charakterPixel=10;

//*** en-disabled items ***
//horizontal separator and labels
private boolean h_separator=true;
//vertical separator
private boolean v_separator=true;

//**** Colors ****
//horizontal separator color
private static Color default_hs_color = new Color(113, 32, 60);
private Color hs_color;
//vertical separator color;
private static Color default_vs_color = Color.gray;
private Color vs_color;

//*************************************************************************
//****************************** Constructor ******************************
//*************************************************************************

public newColumnGraphPanel(GraphDisplayDialog gdd,ArrayList Source) {
```

```
        super (gdd);

        this.Source = Source;

        analyseData();

        updateCalculatedVariables();

        createLayout();
    }

    //************************************************************************
    //***************** Methods for creating display content *****************
    //************************************************************************
    /**
     * Method to combine all JPanel to the main content Panel
     */
    private void createLayout() {

        this.setLayout( null );

        headerPanel = createHeader();
        optionPanel = createOptionPane();
        diagramPanel = createDiagram();

        this.add(headerPanel);
        this.add(optionPanel);
        this.add(diagramPanel);
    }

    /**
     * @return Panel for Header
     */
    private JPanel createHeader() {
        JPanel headerPanel = new JPanel();
        headerPanel.setLayout( null );

headerPanel.setBounds(leftBorder+diagramHorizontalBorder,topBorder,headerWidth,headerHeigh
t);


        //header label
        JLabel headerLabel = new
JLabel(((EventEntry)Source.get(0)).getEvent().getType().toString());
        headerLabel.setFont(new Font("header",3,15));
        headerLabel.setBounds(0,0,70,25);
        headerPanel.add(headerLabel);

        //video link information
        JLabel videoLinkLabel = new JLabel("Click on entry to link to video");
        videoLinkLabel.setFont(new Font("link",Font.ITALIC,12));
        videoLinkLabel.setBounds(0,30,170,10);
        headerPanel.add(videoLinkLabel);
```

```
        return headerPanel;
    }

    /**
     * @return Panel for Option panel
     */
    private JPanel createOptionPane() {
        //*** OptionPanel ***
        JPanel optionPanel = new JPanel();
        optionPanel.setLayout( null );
        optionPanel.setBorder(BorderFactory.createLineBorder(Color.gray,1));

optionPanel.setBounds(leftBorder+diagramHorizontalBorder,topBorder+headerHeight+panelBord
er,optionWidth,optionHeight);


        //*** Label ***
        JLabel label = new JLabel("OPTIONS");
        label.setFont(new Font("options",3,10));
        label.setBounds(5,7,50,25);
        optionPanel.add(label);

        //*** Zoom ***
        //ZoomIn
        Icon icon = new ImageIcon ("OptionIcons\\lupe_plus_20.jpg");
        JButton ZoomIn = new JButton(icon);
        ZoomIn.setToolTipText("Zoom in");
        ZoomIn.setBounds(60,7,25,25);
        ZoomIn.addActionListener( new ActionListener() {
            public void actionPerformed(ActionEvent e) {
                ZF*=2;
                updateContent();
            }
        }
        );
        optionPanel.add(ZoomIn);

        //ZoomOut
        icon = new ImageIcon ("OptionIcons\\lupe_minus_20.jpg");
        JButton ZoomOut = new JButton(icon);
        ZoomOut.setBounds(90,7,25,25);
        ZoomOut.setToolTipText("Zoom out");
        ZoomOut.addActionListener( new ActionListener() {
            public void actionPerformed(ActionEvent e) {
                ZF/=2;
                updateContent();
            }
        }
        );
        optionPanel.add(ZoomOut);

        //ZoomLabel
```

```
        JLabel zoomLabel = new JLabel(""+ZF);
        zoomLabel.setFont(new Font("zooml",3,11));
        zoomLabel.setBounds(125,7,25,25);
        optionPanel.add(zoomLabel);

        //*** Enable - Disable ***
        //En- Disable vertical vertical lines
         icon = new ImageIcon ("OptionIcons\\vertical.jpg");
         JButton VerticalLines = new JButton(icon);
         VerticalLines.setBounds(165,7,25,25);
         VerticalLines.setToolTipText("En/Disable vertical lines");
         VerticalLines.addActionListener( new ActionListener() {
             public void actionPerformed(ActionEvent e) {
                 v_separator = !v_separator;
                 updateContent();
             }
         }
         );
         optionPanel.add(VerticalLines);

        //En- Disable horizontal lines
         icon = new ImageIcon ("OptionIcons\\horizontal.jpg");
         JButton HorizontalLines = new JButton(icon);
         HorizontalLines.setBounds(195,7,25,25);
         HorizontalLines.setToolTipText("En/Disable horizontal lines");
         HorizontalLines.addActionListener( new ActionListener() {
             public void actionPerformed(ActionEvent e) {
                 h_separator = !h_separator;
                 updateContent();
             }
         }
         );
         optionPanel.add(HorizontalLines);

         //*** Change Colors ***
         //horizontal Color change
         icon = new ImageIcon ("OptionIcons\\horizontal_color.jpg");
         JButton HorizontalColor = new JButton(icon);
         HorizontalColor.setBounds(235,7,25,25);
         HorizontalColor.setToolTipText("Change horizontal lines color");
         HorizontalColor.addActionListener( new ActionListener() {
            public void actionPerformed(ActionEvent e) {
                hs_color = JColorChooser.showDialog(null,"Choose color for horizontal
lines",default_hs_color);
                updateContent();
            }
         }
         );
         optionPanel.add(HorizontalColor);

         //horizontal Color change
         icon = new ImageIcon ("OptionIcons\\vertical_color.jpg");
         JButton VerticalColor = new JButton(icon);
```

```
        VerticalColor.setBounds(265,7,25,25);
        VerticalColor.setToolTipText("Change vertical lines color");
        VerticalColor.addActionListener( new ActionListener() {
          public void actionPerformed(ActionEvent e) {
              hs_color = JColorChooser.showDialog(null,"Choose color for vertical
lines",default_hs_color);
              updateContent();
          }
        }
        );
       optionPanel.add(VerticalColor);

        //save to jpeg
        icon = new ImageIcon("OptionIcons\\jpeg_icon.jpg");
        JButton SaveJpeg = new JButton(icon);
        SaveJpeg.setBounds(310,7,25,25);
        SaveJpeg.setToolTipText("Save diagram as jpg");
        SaveJpeg.addActionListener( new ActionListener () {
          public void actionPerformed(ActionEvent e) {

              //create save file dialog with embedded filter for jpeg files
              JFileChooser fc = new JFileChooser();

              if (LogAggregationTool.isFileDirSet()) {
                 fc.setCurrentDirectory(new File(LogAggregationTool.getFileDir()));
              }

              fc.setDialogTitle("select destination ...");

              int returnVal = fc.showSaveDialog(gdd.superclass);

              if (returnVal == JFileChooser.APPROVE_OPTION) {
                 File file = fc.getSelectedFile();

                 String filePath = file.getAbsolutePath();
                 String fileName = file.getName();

                 boolean flag1 = true;

                 //Check if file extists, and in case ask for overwrite
                 if (file.exists()) {
                    int returnVal2 = JOptionPane.showConfirmDialog(null,"Overwrite existing
file?","Overwrite",JOptionPane.YES_NO_OPTION);

                    if (returnVal2 != JOptionPane.YES_OPTION) {
                       flag1 = false;
                       return;
                    }
                 }
                 else {
                    //in case file not exists, check for extention
                    //In case no extention given, create standard
                    if (fileName.lastIndexOf(".") == -1) {
```

```
                    filePath += ".jpg";
                    fileName += ".jpg";
                }
            }

            if (flag1) {

                if (fileName.lastIndexOf(".")==0) {
                    JOptionPane.showMessageDialog(gdd.superclass,"Not allowed to enter empty
file name","Error",JOptionPane.ERROR_MESSAGE);
                    return;
                }


                //check for equal extention of type
                if (fileName.indexOf(".") !=-1 &&
fileName.substring(fileName.lastIndexOf(".")).equalsIgnoreCase(".jpg")) {

                    //process output with correct extention
                    try {
                        ScreenImage.createImage(diagramPanel,filePath);
                        JOptionPane.showMessageDialog(gdd.superclass,"Saving
sucessfull!","INFO",JOptionPane.INFORMATION_MESSAGE);
                    }
                    catch (IOException ex) {
                        JOptionPane.showMessageDialog(gdd.superclass,"Error creating
image!","Error",JOptionPane.ERROR_MESSAGE);
                    }
                }
                else {
                    JOptionPane.showMessageDialog(gdd.superclass,"Only extention '.jpg'
allowed!","Error",JOptionPane.ERROR_MESSAGE);
                }
            }

        }

        //set global file dir
        LogAggregationTool.setFileDir(fc.getCurrentDirectory().getPath());
      }
    });
    optionPanel.add(SaveJpeg);

    //******* combo box *******
    Object [] disInvisibleItems = new Object [UniqueValues.length -
visibleUniqueValues.length+1];

    //define first entry which is shown at beginning
    disInvisibleItems[0] = "- - -";

    //get invisible items
    Object [] invisibleItems = this.getInvisibleItems();
```

```
        //add items to display list
        for (int i = 1; i < disInvisibleItems.length; i++) {
           disInvisibleItems[i] = invisibleItems[i-1];
        }

        //create combo box
        JComboBox invisibleItemBox = new JComboBox(disInvisibleItems);
        invisibleItemBox.setBounds(345,7,70,25);
        invisibleItemBox.addActionListener( new ActionListener() {
           public void actionPerformed(ActionEvent e) {
               Object selected = ((JComboBox)e.getSource()).getSelectedItem();

               if (!selected.equals("- - -")) {
                //if selected value not initial dummy value, add to visible list and repaint
                addValue(selected);
                updateContent();
               }
           }
        }
        );
        optionPanel.add(invisibleItemBox);

        return optionPanel;
    }

    /**
     * @return Panel for diagram
     */
    private JPanel createDiagram() {

        JPanel diagramPanel = new JPanel();
        diagramPanel.setLayout ( null );

diagramPanel.setBounds(leftBorder,topBorder+headerHeight+panelBorder+optionHeight+panelB
order,diagramWidth,diagramHeight);

        //************************* header labels *****************************
        float xValue=0;
        //float offset;

        //For each Value create HeaderButton
        for (int i=0; i< visibleUniqueValues.length; i++) {
           //TODO out commented text creates labels instead of buttons
           /*
           //calculate x postion of label
           xValue = diagramHorizontalBorder + i*actualValueDistance;
           offset = (actualValueDistance-charakterPixel*((String)visibleUniqueValues[i]).length())/2;

           //Only if offset columnlength is larger then textlength, center
           if (offset >0) {
               xValue+=offset;
           }
```

```
          JLabel label = new JLabel((String)visibleUniqueValues[i]);

label.setBounds((int)xValue,diagramVerticalBorder/5*3,8*((String)visibleUniqueValues[i]).length(
),15);
          //label.setBorder(BorderFactory.createLineBorder(Color.black, 1));
          diagramPanel.add(label);
          */
           xValue = diagramHorizontalBorder + i*actualValueDistance+2;

          JButton labelButton = new JButton((String)visibleUniqueValues[i]);
          labelButton.setToolTipText("remove "+(String)visibleUniqueValues[i]);
          labelButton.setFont(new Font("labelButton",1,10));
          labelButton.setBorder(BorderFactory.createEmptyBorder(0, 0, 0, 0));
          labelButton.setBounds((int)xValue,diagramVerticalBorder/5*2,(int)actualValueDistance-
4,15);
          labelButton.setActionCommand(""+(String)visibleUniqueValues[i]);
          labelButton.addActionListener( new ActionListener() {
            public void actionPerformed(ActionEvent e) {
                removeValue(e.getActionCommand());
                updateContent();
            }
          }
          );
          diagramPanel.add(labelButton);
      }

      //*************************** data content ***************************
       for (int i=0; i < Source.size(); i++) {
           EventEntry ee = (EventEntry) Source.get(i);

           //Compare entry with all unique Values, in case of match draw at position
           for (int j=0; j< visibleUniqueValues.length; j++) {

               if (visibleUniqueValues[j].equals(ee.getEvent().getValue().toString())) {

                   //create special button defined below to contain additional event entry
                   DiagramButton button = new DiagramButton(ee);

                   float widthY=0;

                   //if entry is duration
                   if (ee.isDuration()) {
                       //define Y-length of button
                       widthY = (ee.EndTime-ee.StartTime);
                       button.setToolTipText(ee.StartTime+" - "+ee.EndTime);

                   }
                   else {
                       widthY=1;
                       button.setToolTipText(""+ee.StartTime);

                   }
```

```
                button.setBounds((int)(diagramHorizontalBorder + j*actualValueDistance +
actualValueDistance/10),(int)(diagramVerticalBorder
+ee.StartTime*ZF),(int)(actualValueDistance*8/10),(int)(widthY*ZF));

                diagramPanel.add(button);
                break;
              }
           }
        }
        //**************************** coord- system ***************************

        //horizontal Zeroline
        JSeparator horZeroline = new JSeparator();
        horZeroline.setBounds(diagramHorizontalBorder,diagramVerticalBorder-1,diagramWidth-
diagramHorizontalBorder*2,2);
        horZeroline.setBackground(Color.gray);
        diagramPanel.add(horZeroline);

        //ZeroLabel
        JLabel zeroValueLabel = new JLabel("0");
        zeroValueLabel.setFont(new Font("labeling",1,10));
        zeroValueLabel.setBounds(diagramHorizontalBorder/5*3,diagramVerticalBorder-11,20,20);
        diagramPanel.add(zeroValueLabel);

        //vertical Zeroline
        JSeparator verZeroline = new JSeparator(JSeparator.VERTICAL);
        verZeroline.setBounds(diagramHorizontalBorder-1,diagramVerticalBorder,2,diagramHeight-
diagramVerticalBorder*2);
        verZeroline.setBackground(Color.gray);
        diagramPanel.add(verZeroline);

        //create vertical separators between values
        if (v_separator) {

           for (int i = 0; i< visibleUniqueValues.length; i++) {
              JSeparator v_sep = new JSeparator(JSeparator.VERTICAL);

v_sep.setBounds(diagramHorizontalBorder+(i+1)*(int)actualValueDistance,diagramVerticalBorder
,1,diagramHeight-diagramVerticalBorder*2);

              if (vs_color != null) {
                 v_sep.setForeground(vs_color);
              }
              else {
                 v_sep.setForeground(default_vs_color);
              }

              diagramPanel.add(v_sep);
           }
        }

        if (h_separator) {
```

```
        //Counts esspecially the number displayed on the labels
        float labelCounter = 0;

        for (float i = diagramVerticalBorder+actualSepDistance; i<= diagramHeight-
diagramVerticalBorder; i+=actualSepDistance) {

            //inclease labelCounter
            labelCounter += sepDistance / ZF;

            //*** separator ***
            JSeparator h_sep = new JSeparator();
            h_sep.setBounds(diagramHorizontalBorder,(int)i,diagramWidth-
diagramHorizontalBorder*2,1);

            //*** label ***
            JLabel h_label;
            String disValue = ""+labelCounter;
            int pointIndex = disValue.indexOf(".");
            //If . found (means number is float) and there are more then two decial placed, cut at
this pos
            if (pointIndex != -1 && disValue.length() > pointIndex+2) {
                disValue = disValue.substring(0,pointIndex+3);
            }

            h_label = new JLabel(disValue);
            h_label.setHorizontalAlignment(SwingConstants.RIGHT);
            h_label.setFont(new Font("labeling",1,10));
            h_label.setBounds(0,(int)i-11,diagramHorizontalBorder-2,20);

            //set colors
            if (hs_color != null) {
                h_sep.setForeground(hs_color);
                h_label.setForeground(hs_color);
            }
            else {
                h_sep.setForeground(default_hs_color);
                h_label.setForeground(default_hs_color);
            }

            diagramPanel.add(h_sep);
            diagramPanel.add(h_label);
        }
    }

    return diagramPanel;
  }

  //*************************************************************************
  //*********** Methods for calculation and repainting **********************
  //*************************************************************************

  /**
   * calculates all data information necessary for output from data source
```

```
 */
private void analyseData() {

   //get unique values from data source
   UniqueValues = DataEntry.getUniqueEventValues(Source);

   //at beginning define visible unique values as Unique values
   visibleUniqueValues = cloneArray(UniqueValues);

   //get highest start- and endtime of data source
   float [] times = DataEntry.getHighestTimes(Source);

   highestST = times [0];
   highestET = times[1];
}

/**
 * calculates all variables depend on ZF
 */
private void updateCalculatedVariables() {

    //As vertical zoom only looks better then zoom in both directions, acutialValueDistance is
always same like valueDistance
    //TODO if also horizontal zoom is decired, mulipli with ZF
    actualValueDistance = valueDistance;

    //TODO if desired change sep Distance with ZF
    actualSepDistance = sepDistance;
    //*** calculate diagram height and width ***

    //check if one header value is longer then actualValueDistance
    for (int i = 0; i< visibleUniqueValues.length; i++) {
        if (visibleUniqueValues[i].toString().length() * charakterPixel > actualValueDistance ) {
            //in that case set value distance as length of string * number of pixel of one
charakter
            actualValueDistance = visibleUniqueValues[i].toString().length() * charakterPixel;
        }
    }
    //diagram widht is defined as horizontal border on left and right and number of values *
actual value distance
    diagramWidth = (int)(diagramHorizontalBorder*2 +
visibleUniqueValues.length*actualValueDistance);

    //set option width like diagram width (so border is shown in better way)
    optionWidth = (int)(visibleUniqueValues.length*actualValueDistance);

    //in case of zoom out or removing diagram can become smaler then minimum option
length
    if (optionWidth < minOptionWidth) {
        optionWidth = minOptionWidth;
    }

    //get same length as diagram
```

```
        headerWidth = (int)(visibleUniqueValues.length*actualValueDistance);

        //in case of zoom out or removing diagram can become smaler then minimum header
length
        if (headerWidth < minHeaderWidth) {
            headerWidth = minHeaderWidth;
        }

        //if starttime higher endtime define high with starttime, otherwise with endtime
        //high is calculated with highest time * zoomfaktor + vertical border for top and bottom
        if (highestST > highestET) {
           diagramHeight = (int)(diagramVerticalBorder*2 + highestST*ZF);
        }
        else {
           diagramHeight = (int)(diagramVerticalBorder*2 + highestET*ZF);
        }
    }

    /**
     * update content if attributs changed
     */
    private void updateContent() {

        this.removeAll();

        this.updateCalculatedVariables();;

        this.createLayout();

        this.repaint();

        this.windowChanged();
    }

    /**
    * @return minimum Wigth and Height of this panel
    */
    public Dimension getMinimumDisplaySize() {

        Dimension minDim = new Dimension();

        //if option pane width higher then diagramwidth, set optionwidth, otherwise diagramwidth
        if (optionWidth > diagramWidth) {
           minDim.width = leftBorder*2 + optionWidth + diagramHorizontalBorder;
        }
        else {
            minDim.width = leftBorder*2 + diagramWidth;
        }

        minDim.height = 2 * topBorder + 2 * panelBorder + headerHeight + optionHeight +
diagramHeight;

        return minDim;
```

```
    }

    //***********************************************************************
    //************** Methods to operate with Value Array *********************
    //***********************************************************************
    /**
     * creates a copy of a string! array
     */
    private Object[] cloneArray(Object [] sourceArray) {
        Object [] newArray = new Object[sourceArray.length];

        for (int i=0; i< sourceArray.length; i++) {
            newArray[i] = ""+((String)sourceArray[i]);
        }

        return newArray;
    }

    /**
     * removes given value from visible items array
     */
    private void removeValue(Object value) {
        Object [] newArray = new Object [visibleUniqueValues.length-1];

        int j=0;

        for (int i =0; i< visibleUniqueValues.length; i++) {
            if (!value.equals(visibleUniqueValues[i])) {
                newArray[j] = visibleUniqueValues[i];
                j++;
            }
        }

        visibleUniqueValues = newArray;
    }

    /**
     * adds given value at the end of visible items array
     */
    private void addValue(Object value) {
        Object [] newArray = new Object [visibleUniqueValues.length + 1];

        for (int i =0; i< visibleUniqueValues.length; i++) {
            newArray[i] = visibleUniqueValues[i];
        }

        newArray[newArray.length-1] = value;

        visibleUniqueValues = newArray;
    }

    /**
     * @return array of invisible items
```

```
     */
    private Object[] getInvisibleItems() {

        Object [] invisibleItems = new Object[UniqueValues.length -
visibleUniqueValues.length+1];

        int unvisibleCounter=0;

        for (int i = 0; i < UniqueValues.length; i++) {

            boolean found = false;

            for (int j = 0; j < visibleUniqueValues.length; j++) {
                if (UniqueValues[i].equals(visibleUniqueValues[j])) {
                    found = true;
                }
            }

            if (!found) {
                invisibleItems[unvisibleCounter] = UniqueValues[i];
                unvisibleCounter++;
            }
        }

        return invisibleItems;
    }

    /**
     * This inner class defines the Button to represent an event entry
     * in the diagram
     * Therefore the usual JButton is overloaded to allow saving the event entry
     * additionally
     * It also implements to functionality to display the mediaplayer when clicking on the button
     */
    class DiagramButton extends JButton
    implements ActionListener{

        //Source entry for button
        private EventEntry eventEntry;

        //additional constructor to set event entry
        public DiagramButton(EventEntry eventEntry) {
            super();

            this.eventEntry = eventEntry;

            addActionListener(this);
        }

        /**
         * Sets event entry source of button
         * @param eventEntry as source of button
         */
```

```
        public void setSource( EventEntry eventEntry) {
            this.eventEntry = eventEntry;
        }

        /**
         * get event entry source of button
         * @return event entry as source
         */
        public EventEntry getSource() {
            return eventEntry;
        }

        /**
         * method required by action listener, which is called every time
         * button is pressed
         * In this case media player should be instanced to show video
         * for source entry
         * @param e
         */
        public void actionPerformed(ActionEvent e) {

            //get video settings from settings panel
            VideoSettings vs = gdd.superclass.settingspanel.getInitialVideoSettings();

            //call functionality of abstract graph display to display player for event entry
            AbstractGraphDisplay.showMediaPlayer(vs,eventEntry);
        }
    }
}
```

Src 21 : ObswinLogConverter.java

```
/**
subclass of Log Converter to convert Obswin log-files
Log File Aggregation (LFA) tool V1.1.

Copyright (C) 2008 Biomedical Informatics (BMI), St George's University of London. Citations to
the toolkit should be as 'de Lusignan S, Kumarapeli P, Chan T, Pflug B, van Vlymen J, Jones B,
Freeman GK. The ALFA (activity Log Files Aggregation) toolkit: a method for precise observation
of the consultation. <J Med Internet Res 2008; ..>.

This program is free software: you can redistribute it and/or modify it under the terms of the
GNU General Public License as published by the Free Software Foundation, either version 3 of the
License, or (at your option) any later version.

This program is distributed in the hope that it will be useful, but WITHOUT ANY WARRANTY;
without even the implied warranty of MERCHANTABILITY or FITNESS FOR A PARTICULAR
PURPOSE.  See the GNU General Public License for more details.

You should have received a copy of the GNU General Public License along with this program.  If
not, see <http://www.gnu.org/licenses/>.
```

```
*/

package aggregationtool;

import java.util.ArrayList;
import java.util.Date;
import java.util.Iterator;
import java.util.StringTokenizer;

import javax.swing.JPanel;

public class ObswinLogConverter extends LogConverter {

    //defines timeunit of of all entries in file, NOT the accuracy set in header
    //of file!!!!
    private static float timeUnit = 1.0F;

    private static String DataToken = "/ ,-";
    private String wholeContext;
    private ArrayList hierarchicContent;

    //***********************************************************************
    //************************** constructor *********************************
    //***********************************************************************

    public ObswinLogConverter() {

    }

    public ObswinLogConverter(String FilePath) {
        this.FilePath=FilePath;
    }
    //***********************************************************************
    //********************** property panel method *****************************
    //***********************************************************************

     /**
      * Method to create properties for specific options on format
      * Should instance property Panel of super class, add all necessary components
      * and read them before processing to get required options
      * In case no property panel is needed leave body without content
      */
     public void createPropertyPanel() {

         //TODO In case options are necessary uncomment following lines, add components
         //and read them before processing

          /*
          //*** Panel ***
          propertyPanel = new JPanel();

          propertyPanel.setLayout( null );
```

```
        //TODO add properties
        */
    }
    //***************************************************************************
    //******************* content process methods ****************************
    //***************************************************************************

    /**
     * Method to process file content from read file to create data entries
     */
    public void interpretLogFile() {

        CommonContent = new ArrayList();
        wholeContext= "";
        //float accuracy;

        /********************
         ** process Header **
         ********************/

        try {
            //Get and remove headerinformation (only works in case header is two lines)
            //First line isn't needed
            FileContent.remove(0);

            //NO NEED TO GET ACCURACY DEFINED IN HEADER, if it would be used uncomment
following lines
            /*
            //Second line gives information of the accuracy
            String accurracyString = (String)FileContent.get(0);
            //Remove , at beginning
            accurracyString=accurracyString.substring(1);

            accuracy = Float.parseFloat(accurracyString);
            */
            //After getting information, remove
            FileContent.remove(0);

            //REMOVE Line with total time of duration!!!!!!!!!!!!!!!
            FileContent.remove(FileContent.size()-2);

            this.CommonContent.add(new HeaderEntry(timeUnit,new
Date(this.LastModified),this.FileName));
            //***************
        }
        catch (IndexOutOfBoundsException ex) {
            addErrorWarning(ErrorDialog.ERROR,"Error reading header, maybe wrong file format");
            return;
        }
        catch (NumberFormatException ex2) {
            addErrorWarning(ErrorDialog.ERROR,"Error reading header, maybe wrong file format");
            return;
```

```
        }

        /********************
         ** process Content **
         ********************/

        //put rest of file together, and afterwards separated by tokens
        for (int i = 0; i< FileContent.size();i++) {
            wholeContext+=FileContent.get(i)+" ";
        }

        //Initialise recursive call
        this.hierarchicContent= token_File(wholeContext,0);

        //this.testHierarchicOutput(this.hierarchicContent,0);
        //***************
        //now convert to DataEntries
        //First create Header


        //Run through all entries and add them to the DataEntryList
        Iterator it0= this.hierarchicContent.iterator();

        while (it0.hasNext()) {
            ArrayList al0= (ArrayList) it0.next();

            for (int counter=0; counter<al0.size();counter++)
            {
                //Start of the structure of an entry
                ArrayList al1= (ArrayList) al0.get(counter);

                EventEntry evententry = new EventEntry();

                try {
                    //Get charakter
                    ArrayList tag1=(ArrayList)al1.get(0);

                    evententry.getEvent().setType(LogConverter.OBSWINEVENTTYPE);
                    evententry.getEvent().setValue((String)tag1.get(0));

                    //get times
                    ArrayList tag2=(ArrayList)al1.get(1);

                    //startTime
                    evententry.StartTime= Float.parseFloat((String)tag2.get(0))*timeUnit;

                    if (tag2.size()>1) {
                        //duration
                         evententry.EndTime=
Float.parseFloat(((String)tag2.get(1)).substring(0,((String)tag2.get(1)).length()-1))*timeUnit;
                    }
                    else {
                        evententry.EndTime=EventEntry.ENDTIME_NO_DURATION_VALUE;
```

```
                }


                //Add to Common List
                this.CommonContent.add(evententry);
            }
            catch (IndexOutOfBoundsException ex) {
                addErrorWarning(ErrorDialog.WARNING,this.FileName+"   Unable to convert entry
"+(counter+1)+", entry ignored!");
            }
            catch (ClassCastException ex2) {
                addErrorWarning(ErrorDialog.WARNING,this.FileName+"   Unable to convert entry
"+(counter+1)+", entry ignored!");
            }
            catch (NumberFormatException ex3) {
                addErrorWarning(ErrorDialog.WARNING,this.FileName+"   Unable to convert entry
"+(counter+1)+", entry ignored!");
            }
        }
    }
}

/**
 * recursive method which splits given string by charakter of datatoken depending on depth
 * @param Content to be splitted
 * @param depth of hirarchie
 * @return arraylist containing splitted strings by depth's charakter of data token
 */
private ArrayList token_File (String Content, int depth) {

    ArrayList content = new ArrayList();

    //Whlie not reached the end of DataToken, still continue splitting
    if(depth < DataToken.length()) {

        StringTokenizer st = new StringTokenizer(Content,""+DataToken.charAt(depth));

        while (st.hasMoreTokens()) {
            String tok = st.nextToken();

            if (depth < DataToken.length()-1) {
                content.add(token_File(tok,depth+1));
            }
            else {
                content.add(tok);
            }
        }

    }

    return content;
}
```

```
    /**
     * Method to test hierarchical output in standard output device
     * @param Content hierarchie of arraylists with strings at leafes
     * @param depth for internal recall, start with zero
     */
    @Deprecated
    public void testHierarchicOutput (Object Content,int depth) {
        for (int i=0; i<depth;i++) {
            System.out.print("-");
        }
        if (Content instanceof ArrayList) {
            System.out.println("<hierarchie "+depth+">");
            ArrayList list = (ArrayList)Content;
            Iterator it= list.iterator();

            while (it.hasNext()) {
                testHierarchicOutput(it.next(),depth+1);
            }
        }
        else if (Content instanceof String) {
            System.out.println(Content);
        }
        else {
            System.out.println("<unknown object>");
        }
    }
}
```

Src 22 : OutputConverter.java

/**
defines the abstract layout of each converter creating an output file
Log File Aggregation (LFA) tool V1.1.

Copyright (C) 2008 Biomedical Informatics (BMI), St George's University of London. Citations to the toolkit should be as 'de Lusignan S, Kumarapeli P, Chan T, Pflug B, van Vlymen J, Jones B, Freeman GK. The ALFA (activity Log Files Aggregation) toolkit: a method for precise observation of the consultation. <J Med Internet Res 2008; ..>.

This program is free software: you can redistribute it and/or modify it under the terms of the GNU General Public License as published by the Free Software Foundation, either version 3 of the License, or (at your option) any later version.

This program is distributed in the hope that it will be useful, but WITHOUT ANY WARRANTY; without even the implied warranty of MERCHANTABILITY or FITNESS FOR A PARTICULAR PURPOSE. See the GNU General Public License for more details.

You should have received a copy of the GNU General Public License along with this program. If not, see <http://www.gnu.org/licenses/>.

```
*/

package aggregationtool;

import java.awt.BorderLayout;

import java.awt.Font;

import java.io.BufferedWriter;
import java.io.FileWriter;
import java.io.IOException;

import java.util.ArrayList;
import java.util.zip.DataFormatException;

import javax.swing.JLabel;
import javax.swing.JPanel;

/**
 * HOW TO ADD NEW OUTPUT-CONVERTER SUBLCASS
 *
 * following steps are necessary to add new output converter:
 * - create new class extending output converter
 * OUTPUT-Converter
 * - add static variable
 * - add static to function getSubclassNames
 * - add functionality to getSubclassByName
 *
 * @author Bernhard Pflug
 */
public abstract class OutputConverter {

    //*** STATICS ***
    //must be unique
    public static String SDS = "sds";
    public static String XML = "xml";
    public static String CSV = "csv";

    //Define Height and Width of property panel
    public static int PROPERTY_PANEL_WIDTH = 650;
    public static int PROPERTY_PANEL_HEIGHT = 200;

    //*** VARIABLES ***

    //data source
    ArrayList dataSource;

    //String with whole converted data
    protected String outputString;

    //path the file will be saved
    protected String filePath;
```

```
//panel which contains all settings for the format
protected JPanel propertyPanel;

//**********************************************************************
//************************* constructor ********************************
//**********************************************************************
public OutputConverter() {

}

//**********************************************************************
//************************ abstract methods *****************************
//**********************************************************************

/**
 * method to create specific output
 * @throws DataFormatException in case any error occurs in converting
 */
protected abstract void convertSource() throws DataFormatException;

/**
 * This method should be implemented to create the property panel for display
 * in case new property panel is required, leave method skeleton without body
 */
protected abstract void createPropertyPanel();


//**********************************************************************
//********************** implemented methods ****************************
//**********************************************************************

 /**
  * creates JPanel in prefered size of 650/200 which contains all required
  * properties of each output type
  * in case instance is null create default panel with no information
  * @return JPanel with required propertie content
  */
 public JPanel getPropertyPanel() {

    if (propertyPanel != null) {
        //set panel size to default size
        propertyPanel.setSize(PROPERTY_PANEL_WIDTH,PROPERTY_PANEL_HEIGHT);

        return propertyPanel;
    }
    else {
        //In case no panel is set by subclass default panel is created
        JPanel defaultPanel = new JPanel();
        defaultPanel.setSize(PROPERTY_PANEL_WIDTH,PROPERTY_PANEL_HEIGHT);
        defaultPanel.setLayout(new BorderLayout());
        JLabel defaultLabel = new JLabel("<no properties available for this output type>");
        defaultLabel.setFont(new Font("TIMES NEW ROMAN",Font.ITALIC,15));
        defaultPanel.add(defaultLabel,BorderLayout.CENTER);
```

```
        return defaultPanel;
    }
  }

  /**
   * sets the data source for output
   * @param dataSource Arraylist with dataEntries
   */
  public void setDataSource (ArrayList dataSource) {
     this.dataSource = dataSource;
  }

  /**
   * method to write converted content to a file
   * @throws IOException in case writing process isn't sucessfull
   */
  protected void writeToFile() throws IOException {

     if (filePath != null) {

        BufferedWriter bf = new BufferedWriter(new FileWriter(filePath));


        outputString.replaceAll("\n",""+(char)Character.LINE_SEPARATOR);

        bf.write(outputString);

        bf.close();
     }
     else {
        throw new IOException("No filepath set");
     }
  }

 /**
  * Method to convert Source and write it to file
  */
 public void createOutputFile() throws DataFormatException,IOException {

    //at first convert source
    convertSource();

    //afterwards write to file
    writeToFile();
 }

 /**
  * Method to set filepath for output file
  * @param filePath where converted content will be saved
  */
 public void setFilePath(String filePath) {
    this.filePath = filePath;
 }
```

```
    //**************************************************************************
    //**************************** statics ************************************
    //**************************************************************************
    /**
     * @return all statics of subclasses
     */
    public static String[] getSubclassNames() {
        return new String [] {SDS,XML,CSV};
    }

    /**
     * @param name : String with name of subclass and datasource to create output
     */
    public static OutputConverter getSubclassByName(String name) {

        if (name.equals(SDS)) {
            return new SDSOutputConverter();
        }
        else if (name.equals(XML)) {
            return new XMLOutputConverter();
        }
        else if (name.equals(CSV)) {
            return new CSVOutputConverter();
        }
        else {
            return null;
        }
    }

}
```

Src 23 : OutputDeviceManager.java

```
/**
This class is used to set output properties for unexpected output from any java class
Log File Aggregation (LFA) tool V1.1.

Copyright (C) 2008 Biomedical Informatics (BMI), St George's University of London. Citations to
the toolkit should be as 'de Lusignan S, Kumarapeli P, Chan T, Pflug B, van Vlymen J, Jones B,
Freeman GK. The ALFA (activity Log Files Aggregation) toolkit: a method for precise observation
of the consultation. <J Med Internet Res 2008; ..>.

This program is free software: you can redistribute it and/or modify it under the terms of the
GNU General Public License as published by the Free Software Foundation, either version 3 of the
License, or (at your option) any later version.

This program is distributed in the hope that it will be useful, but WITHOUT ANY WARRANTY;
without even the implied warranty of MERCHANTABILITY or FITNESS FOR A PARTICULAR
PURPOSE.  See the GNU General Public License for more details.
```

```
You should have received a copy of the GNU General Public License along with this program.  If
not, see <http://www.gnu.org/licenses/>.

*/

package aggregationtool;

import java.io.FileNotFoundException;
import java.io.FileOutputStream;
import java.io.PrintStream;


public class OutputDeviceManager {

    //statics to define output device
    public static int FILE_OUTPUT = 1;

    //file path if for file output
    private static String filePath = "C:";

    /**
     * Defines output device for application
     * @param DEVICE value for output device, choose of statics in this class
     */
    public static void setOutputDevice(int DEVICE) {

        //in case file output device is selected
        if (DEVICE == FILE_OUTPUT) {
            try {
                System.setOut(new PrintStream(new FileOutputStream(filePath+"\\msgOut.txt")));
                System.setErr(new PrintStream(new FileOutputStream(filePath+"\\errOut.txt")));

            }
            catch(FileNotFoundException ex) {
                System.out.println("LogAggreationTool: outputfile not found");
            }
        }
    }

    /**
     * In case of file output this method defines the directory
     * @param filePath to create output files
     */
    public static void setFileOutputPath(String filePath) {
        OutputDeviceManager.filePath = filePath;
    }

    /**
     * sets current directory for file output
     */
    public static void setCurrentDirForFileOutput() {
        OutputDeviceManager.filePath = System.getProperty("user.dir");
    }
```

```
}
```

Src 24 : OutputPanel.java

```
/**
Java class to create panel for output step
Log File Aggregation (LFA) tool V1.1.

Copyright (C) 2008 Biomedical Informatics (BMI), St George's University of London. Citations to
the toolkit should be as 'de Lusignan S, Kumarapeli P, Chan T, Pflug B, van Vlymen J, Jones B,
Freeman GK. The ALFA (activity Log Files Aggregation) toolkit: a method for precise observation
of the consultation. <J Med Internet Res 2008; ..>.

This program is free software: you can redistribute it and/or modify it under the terms of the
GNU General Public License as published by the Free Software Foundation, either version 3 of the
License, or (at your option) any later version.

This program is distributed in the hope that it will be useful, but WITHOUT ANY WARRANTY;
without even the implied warranty of MERCHANTABILITY or FITNESS FOR A PARTICULAR
PURPOSE.  See the GNU General Public License for more details.

You should have received a copy of the GNU General Public License along with this program.  If
not, see <http://www.gnu.org/licenses/>.

*/

package aggregationtool;

import java.awt.Rectangle;
import java.awt.event.ActionEvent;
import java.awt.event.ActionListener;

import java.awt.event.KeyEvent;

import java.io.File;

import java.io.IOException;

import java.util.zip.DataFormatException;

import javax.swing.BorderFactory;
import javax.swing.JButton;
import javax.swing.JComboBox;
import javax.swing.JFileChooser;
import javax.swing.JLabel;
import javax.swing.JOptionPane;
import javax.swing.JPanel;
import javax.swing.border.TitledBorder;

 */
public class OutputPanel extends JPanel
```

```
implements StepPanelInterface{

    //instance of superclass
    GUISuperclass superclass;

    //*** GUI elements ***
    private JComboBox fileTypeList;

    private JButton next_button = new JButton();
    private JButton prev_button = new JButton();
    private JButton output_button = new JButton();

    private JPanel propertyPanel;

    //*** converter ***
    OutputConverter converter;
    String filePath;

    //**************************************************************************
    //*************************** constructor *********************************
    //**************************************************************************
    public OutputPanel(GUISuperclass superclass) {

        this.superclass = superclass;

        try {
            jbInit();
        } catch (Exception e) {
            e.printStackTrace();
        }
    }

    private void jbInit() throws Exception {
        this.setLayout( null );

        //***** output type ********
        JLabel fileTypeLabel = new JLabel("Choose file type...");
        fileTypeLabel.setBounds(150,60,100,20);

        fileTypeList = new JComboBox(OutputConverter.getSubclassNames());
        fileTypeList.setBounds(280,60,100,20);
        fileTypeList.addActionListener(new ActionListener() {
                public void actionPerformed(ActionEvent e) {
                    //in case list entry is changed change converter and properties
                    updateFileType();
                }
            }
        );

        //*** save button ***
         //SaveButton
         JButton saveButton = new JButton("Save...");
         saveButton.setBounds(370,380,80,30);
```

```
        saveButton.setMnemonic(KeyEvent.VK_S);
        saveButton.setToolTipText("close application");
        saveButton.addActionListener(new ActionListener() {
              public void actionPerformed(ActionEvent e) {

                 JFileChooser fc = new JFileChooser();

                 if (LogAggregationTool.isFileDirSet()) {
                    fc.setCurrentDirectory(new File(LogAggregationTool.getFileDir()));
                 }

                 fc.setDialogTitle("select destination ...");

                 int returnVal = fc.showSaveDialog(superclass);

                 if (returnVal == JFileChooser.APPROVE_OPTION) {
                    File file = fc.getSelectedFile();

                    filePath = file.getAbsolutePath();
                    String fileName = file.getName();

                    boolean flag1 = true;

                    //Check if file extists, and in case ask for overwrite
                    if (file.exists()) {
                       int returnVal2 = JOptionPane.showConfirmDialog(null,"Overwrite existing
file?","Overwrite",JOptionPane.YES_NO_OPTION);

                       if (returnVal2 != JOptionPane.YES_OPTION) {
                          flag1 = false;
                          return;
                       }
                    }
                    else {
                       //in case file not exists, check for extention
                       //In case no extention given, create standard
                       if (fileName.lastIndexOf(".") == -1) {
                          filePath += "."+fileTypeList.getSelectedItem();
                          fileName += "."+fileTypeList.getSelectedItem();
                       }
                    }

                    if (flag1) {

                       if (fileName.lastIndexOf(".")==0) {
                          JOptionPane.showMessageDialog(null,"Not allowed to enter empty file
name","Error",JOptionPane.ERROR_MESSAGE);
                          return;
                       }


                       //check for equal extention of type
```

```
                        if (fileName.indexOf(".") !=-1 &&
fileName.substring(fileName.lastIndexOf(".")).equalsIgnoreCase("."+fileTypeList.getSelectedItem
())) {

                            //process output with correct extention
                            process_output();
                        }
                        else {
                            int returnVal3 = JOptionPane.showConfirmDialog(null,"Extention differs to
format, save anyway?","different type",JOptionPane.YES_NO_OPTION);

                            if (returnVal3 == JOptionPane.YES_OPTION) {

                                //process output with incorrect extention
                                process_output();
                            }
                        }
                    }

                }

                //set global file dir
                LogAggregationTool.setFileDir(fc.getCurrentDirectory().getPath());
            }
        }
    );

    updateFileType();

    //****** navigation buttons ******
    //prev_button
    prev_button.setText("prev");
    prev_button.setBounds(460,380,80,30);
    prev_button.setMnemonic(KeyEvent.VK_P);
    prev_button.setToolTipText("previous step");
    prev_button.addActionListener(new ActionListener() {
        public void actionPerformed(ActionEvent e) {
            display_previous();
        }
      }
    );

    //next/ finish button
    next_button.setText("finish");
    next_button.setBounds(new Rectangle(550, 380, 80, 30));
    next_button.setMnemonic(KeyEvent.VK_N);
    next_button.setToolTipText("ALT - N");
    next_button.addActionListener(new ActionListener() {
            public void actionPerformed(ActionEvent e) {
                display_next();
            }
    });
```

```
        //add all components to content pane
        this.add(saveButton);
        this.add(fileTypeLabel);
        this.add(fileTypeList);
        this.add(next_button);
        this.add(prev_button);
        this.add(output_button);
    }

    //*********************************************************************
    //***************** create Converter and its properties *******************
    //*********************************************************************

    public void updateFileType() {

        //get instance of converter
        converter =
OutputConverter.getSubclassByName(fileTypeList.getSelectedItem().toString());

        //if propertyPanel already exists disable it
        if (propertyPanel != null) {
            propertyPanel.setVisible(false);
        }

        //draw its properties in panel
        propertyPanel = converter.getPropertyPanel();
        TitledBorder propBorder = BorderFactory.createTitledBorder("properties");
        propBorder.setTitleJustification(TitledBorder.CENTER);
        propertyPanel.setBorder(propBorder);

        propertyPanel.setLocation(0,150);
        this.add(propertyPanel);
        this.repaint();
    }

    //*********************************************************************
    //************************** process output ****************************
    //*********************************************************************

    public void process_output() {

        //set file path of output file
        converter.setFilePath(filePath);
        converter.setDataSource(superclass.displaypanel.displayData);

        try {
            converter.createOutputFile();
            JOptionPane.showMessageDialog(superclass,"Saving successful!");
        }
        catch (DataFormatException ex) {
            ErrorDialog errDialog = new ErrorDialog(superclass,"Errors at output",true);
            errDialog.addMessage(ex.getMessage(),ErrorDialog.ERROR);
```

```
            errDialog.showErrDialog();
        }
        catch (IOException ex2) {
            ErrorDialog errDialog = new ErrorDialog(superclass,"Errors at output",true);
            errDialog.addMessage(ex2.getMessage(),ErrorDialog.ERROR);
            errDialog.showErrDialog();
        }
    }

    //************************************************************************
    //********************** methods for navigation ****************************
    //************************************************************************
    public void display_next() {
        //TODO implement in case further steps should be created
        System.exit(0);
    }

    public void display_previous() {
        this.setVisible(false);
        superclass.displayPanel(superclass.displaypanel,5);
    }
}
```

Src 25 : PRSLogConverter.java

```
/**
Subclass of log converter processing PRS log files
Log File Aggregation (LFA) tool V1.1.

Copyright (C) 2008 Biomedical Informatics (BMI), St George's University of London. Citations to
the toolkit should be as 'de Lusignan S, Kumarapeli P, Chan T, Pflug B, van Vlymen J, Jones B,
Freeman GK. The ALFA (activity Log Files Aggregation) toolkit: a method for precise observation
of the consultation. <J Med Internet Res 2008; ..>.

This program is free software: you can redistribute it and/or modify it under the terms of the
GNU General Public License as published by the Free Software Foundation, either version 3 of the
License, or (at your option) any later version.

This program is distributed in the hope that it will be useful, but WITHOUT ANY WARRANTY;
without even the implied warranty of MERCHANTABILITY or FITNESS FOR A PARTICULAR
PURPOSE.  See the GNU General Public License for more details.

You should have received a copy of the GNU General Public License along with this program.  If
not, see <http://www.gnu.org/licenses/>.

*/

package aggregationtool;

import java.awt.event.ActionEvent;
import java.awt.event.ActionListener;
```

```
import java.util.ArrayList;
import java.util.Date;
import java.util.NoSuchElementException;
import java.util.StringTokenizer;
import java.util.zip.DataFormatException;

import javax.swing.JComboBox;
import javax.swing.JLabel;
import javax.swing.JPanel;

/**
 * structure should look like this
 * - first two lines of file are ignored, containing only unnecessary headerinformatino
 * - after that each line is processed
 * - TIME_THRESHOLD defines in which duration two entries are combined to one single entry
with an duration
 * - DIV_VALUE defines the value all time values must be divided by to get seconds
 *
 * @author Bernhard Pflug
 */
public class PRSLogConverter extends LogConverter {

    //Token for the file
    private static String TOKEN = "\t";

    //Variable which defines the time between two entries to be combined to one duration
    //TO BE DEFINED IN SAME UNIT LIKE FILE (usually MILLISECONDS)
    private Float [] TIME_THRESHOLD = {100F, 500F, 1000F, 2000F, 3000F, 4000F, 5000F};
    private int selectedThresIndex = 2;

    //ArrayList which contains the raw DataEntries after converting, but without combining
    private ArrayList rawEntries;

    //******** property panel components *********
    JComboBox thresholdCombobox = new JComboBox(TIME_THRESHOLD);

    //*************************************************************************
    //************************** constructor ************************************
    //*************************************************************************

    public PRSLogConverter () {

    }

    public PRSLogConverter(String filePath) {
        this.FilePath = filePath;

    }
    //*************************************************************************
    //********************** property panel method ****************************
    //*************************************************************************
```

```
    /**
     * Method to create properties for specific options on format
     * Should instance property Panel of super class, add all necessary components
     * and read them before processing to get required options
     * In case no property panel is needed leave body without content
     */
    public void createPropertyPanel() {

        //TODO In case options are necessary uncomment following lines, add components
        //and read them before processing


        //*** Panel ***
        propertyPanel = new JPanel();

        propertyPanel.setLayout( null );

        //Threshold label
        JLabel thresholdLabel = new JLabel("Choose threshold between two values to get
combined");
        thresholdLabel.setBounds(20,30,350,20);

        //Threshold combo box
        thresholdCombobox.setBounds(20,60,100,20);
        thresholdCombobox.setSelectedIndex(selectedThresIndex);
        thresholdCombobox.addActionListener(new ActionListener() {

            public void actionPerformed(ActionEvent e) {
                //set slectedThresIndex as new selected one in combo box
                selectedThresIndex = thresholdCombobox.getSelectedIndex();
            }
         });

        //add to panel
        propertyPanel.add(thresholdLabel);
        propertyPanel.add(thresholdCombobox);
    }
   //**************************************************************************
   //******************** content process methods *****************************
   //**************************************************************************
   /**
    * Method to interpret read file to create Data entries
    */
   public void interpretLogFile() {

       //ArrayList containing the common Context of the file
       CommonContent = new ArrayList();

       rawEntries = new ArrayList();

       /********************
        ** process Header **
        ********************/
```

```
        //create HeaderEntry
         CommonContent.add(new HeaderEntry(0.001F,new
Date(this.LastModified),this.FileName));

        //remove first two colums
        FileContent.remove(0);
        FileContent.remove(0);

         /********************
          ** process Content **
          ********************/

        //split each row by TOKEN
        StringTokenizer st;

        for (int i =0; i< FileContent.size(); i++) {
            try {
                st = new StringTokenizer((String)FileContent.get(i),TOKEN);

                //First token must be time in milliseconds
                float timeMillis = Float.parseFloat(st.nextToken());

                if (timeMillis < 0) {
                    this.addErrorWarning(ErrorDialog.WARNING,"Negative value found in entry
"+(i+1)+", entry processed");
                }

                //After undefinded number of TOKEN, Entry with number of rectange must follow
                String rectString="";

                while (rectString.equals("") && st.hasMoreTokens()) {
                    rectString = st.nextToken();
                }

                //after that rectString must contain the number of the rectangle
                if (!rectString.equals("")) {
                    //This parse wound not be necessary for the save in EventEntry, but is for
controlling if value is number
                    int rectNumber = Integer.parseInt(rectString);

                    //Now create EventEntry in rawEntries list
                    rawEntries.add(new EventEntry(timeMillis,-
1,LogConverter.PRSEVENTTYPE,""+rectNumber));
                }
                else {
                    throw new DataFormatException("number for rectangle missing in entry
"+(i+1)+", entry ignored!");
                }
            }
            catch (NumberFormatException ex) {
                this.addErrorWarning(ErrorDialog.WARNING,"Unable to create numbers of entry
"+(i+1)+", entry ignored!");
            }
```

```
            catch (DataFormatException ex2) {
                this.addErrorWarning(ErrorDialog.WARNING,ex2.getMessage());
            }
            catch (NoSuchElementException ex3) {
                this.addErrorWarning(ErrorDialog.ERROR,"Error in file format, maybe wrong file
format!");
                break;
            }
        }

        //**********************************************************************

        //After creating rawContent entries will be combined
        createCommonContentFromRawEntries();
    }

    /**
     * After interpretation all EventEntries are saved in RawEntries
     * this function combines all entries which starttime is less then the defined
     * value to one duration entry
     * The result is saved in commonContent
     */
    private void createCommonContentFromRawEntries() {

        while (!rawEntries.isEmpty()) {

            EventEntry entry = (EventEntry) rawEntries.get(0);

            float highestDifference = searchHighestDiff(entry);

            //After searching through list and removing all entries in intervall (including entry it's
own),
            //add entry with duration or as event, if no other entries were found
            if (highestDifference >0) {
                CommonContent.add(new
EventEntry(entry.StartTime,(entry.StartTime+highestDifference),LogConverter.PRSEVENTTYPE,e
ntry.getEvent().getValue()));
            }
            else {
                CommonContent.add(new
EventEntry(entry.StartTime,EventEntry.ENDTIME_NO_DURATION_VALUE,LogConverter.PRSEVEN
TTYPE,entry.getEvent().getValue()));
            }

            //after searching an entry, remove from list
            //rawEntries.remove(entry);
        }

    }

    /**
     * This function is called recursiv to search through list and combine all
     * entries in specified intervall
```

```
     * recursion is needed because in case finding an entry in interval also it's
     * entries in interval must be searched and combined
     * @param entry to search for
     * @return highest difference given value and all other values in list (in case of queue of some values last value given)
     */
    private float searchHighestDiff(EventEntry entry) {

        // before processing with list delete value it self to get no dead look
        rawEntries.remove(entry);

        //defines the highest difference between given object and all other objects in list in interval
        float highestDifference = -1;

        //Defines the difference between to given object and compared object in list
        float currDiff;

        //go through list from back to front and search for entries in intervall
        for (int i =rawEntries.size()-1; i>=0; i--) {
            EventEntry toCompare = (EventEntry)rawEntries.get(i);

            //only compare times if entries have same value
            if (entry.getEvent().getValue().equals(toCompare.getEvent().getValue())) {

                //check whether compared entries starttime is between starttime and starttime+Threshold
                if (toCompare.StartTime >= entry.StartTime && toCompare.StartTime <= (entry.StartTime+(TIME_THRESHOLD[selectedThresIndex]).floatValue())) {

                    //create diffence of given value and compare to
                    currDiff = toCompare.StartTime - entry.StartTime;

                    //search for the highestValue of current differene, already compared values (highestDifference) and recursiv search
                    //recursive call searches for other values in intervall of compare to
                    highestDifference = Math.max(highestDifference,Math.max(currDiff,searchHighestDiff(toCompare)+currDiff));


                }
            }
        }

        return highestDifference;
    }
}
```

Src 26 : ScreenImage.java

```
/**
```

class library to create images of GUI components
Log File Aggregation (LFA) tool V1.1.

Copyright (C) 2008 Biomedical Informatics (BMI), St George's University of London. Citations to the toolkit should be as 'de Lusignan S, Kumarapeli P, Chan T, Pflug B, van Vlymen J, Jones B, Freeman GK. The ALFA (activity Log Files Aggregation) toolkit: a method for precise observation of the consultation. <J Med Internet Res 2008; ..>.

This program is free software: you can redistribute it and/or modify it under the terms of the GNU General Public License as published by the Free Software Foundation, either version 3 of the License, or (at your option) any later version.

This program is distributed in the hope that it will be useful, but WITHOUT ANY WARRANTY; without even the implied warranty of MERCHANTABILITY or FITNESS FOR A PARTICULAR PURPOSE. See the GNU General Public License for more details.

You should have received a copy of the GNU General Public License along with this program. If not, see <http://www.gnu.org/licenses/>.

```
*/

package aggregationtool;

import java.awt.*;
import java.awt.event.*;
import java.awt.image.*;
import java.io.*;
import javax.imageio.*;
import javax.swing.*;


public class ScreenImage
{
	/*
	 *  Create a BufferedImage for Swing components.
	 *  The entire component will be captured to an image.
	 *
	 *  @param       component Swing component to create image from
	 *  @param       fileName name of file to be created or null
	 *  @return      image the image for the given region
	 *  @exception IOException if an error occurs during writing
	*/
	public static BufferedImage createImage(JComponent component, String fileName)
		throws IOException
	{
		Dimension d = component.getSize();

		if (d.width == 0)
		{
			d = component.getPreferredSize();
			component.setSize( d );
		}
```

```
		Rectangle region = new Rectangle(0, 0, d.width, d.height);
		return ScreenImage.createImage(component, region, fileName);
	}

	/*
	 *  Create a BufferedImage for Swing components.
	 *  All or part of the component can be captured to an image.
	 *
	 *  @param       component Swing component to create image from
	 *  @param       region The region of the component to be captured to an image
	 *  @param       fileName name of file to be created or null
	 *  @return      image the image for the given region
	 *  @exception IOException if an error occurs during writing
	*/
	public static BufferedImage createImage(JComponent component, Rectangle region,
String fileName)
		throws IOException
	{
		boolean opaqueValue = component.isOpaque();
		component.setOpaque( true );
		BufferedImage image = new BufferedImage(region.width, region.height,
BufferedImage.TYPE_INT_RGB);
		Graphics2D g2d = image.createGraphics();
		g2d.setClip( region );
		component.paint( g2d );
		g2d.dispose();
		component.setOpaque( opaqueValue );
		ScreenImage.writeImage(image, fileName);
		return image;
	}

	/*
	 *  Create a BufferedImage for AWT components.
	 *
	 *  @param       component AWT component to create image from
	 *  @param       fileName name of file to be created or null
	 *  @return      image the image for the given region
	 *  @exception AWTException see Robot class constructors
	 *  @exception IOException if an error occurs during writing
	*/
	public static BufferedImage createImage(Component component, String fileName)
		throws AWTException, IOException
	{
		Point p = new Point(0, 0);
		SwingUtilities.convertPointToScreen(p, component);
		Rectangle region = component.getBounds();
		region.x = p.x;
		region.y = p.y;
		return ScreenImage.createImage(region, fileName);
	}

	/**
	 *  Convenience method to create a BufferedImage of the desktop
```

```
 *
 * @param       fileName name of file to be created or null
 * @return      image the image for the given region
 * @exception AWTException see Robot class constructors
 * @exception IOException if an error occurs during writing
 */
public static BufferedImage createDesktopImage(String fileName)
        throws AWTException, IOException
{
        Dimension d = Toolkit.getDefaultToolkit().getScreenSize();
        Rectangle region = new Rectangle(0, 0, d.width, d.height);
        return ScreenImage.createImage(region, fileName);
}

/**
 * Create a BufferedImage from a rectangular region on the screen.
 *
 * @param       region region on the screen to create image from
 * @param       fileName name of file to be created or null
 * @return      image the image for the given region
 * @exception AWTException see Robot class constructors
 * @exception IOException if an error occurs during writing
 */
public static BufferedImage createImage(Rectangle region, String fileName)
        throws AWTException, IOException
{
        BufferedImage image = new Robot().createScreenCapture( region );
        ScreenImage.writeImage(image, fileName);
        return image;
}

/**
 * Write a BufferedImage to a File.
 *
 * @param       image image to be written
 * @param       fileName name of file to be created
 * @exception IOException if an error occurs during writing
*/
public static void writeImage(BufferedImage image, String fileName)
        throws IOException
{
        if (fileName == null) return;

        int offset = fileName.lastIndexOf( "." );
        String type = offset == -1 ? "jpg" : fileName.substring(offset + 1);

        ImageIO.write(image, type, new File( fileName ));
}

public static void main(String args[])
        throws Exception
{
        final JFrame frame = new JFrame();
```

```
		final JTextArea textArea = new JTextArea(30, 60);
		final JScrollPane scrollPane = new JScrollPane( textArea );
		frame.getContentPane().add( scrollPane );

		JMenuBar menuBar = new JMenuBar();
		frame.setJMenuBar( menuBar );
		JMenu menu = new JMenu( "File" );
		ScreenImage.createImage(menu, "menu.jpg");
		menuBar.add( menu );
		JMenuItem menuItem = new JMenuItem( "Frame Image" );
		menu.add( menuItem );
		menuItem.addActionListener( new ActionListener()
		{
			public void actionPerformed(ActionEvent e)
			{
				//  Let the menu close and repaint itself before taking the image

				new Thread()
				{
					public void run()
					{
						try
						{
							Thread.sleep(50);
							System.out.println("Creating
frame.jpg");
							frame.repaint();
							ScreenImage.createImage(frame,
"frame.jpg");
						}
						catch(Exception exc) { System.out.println(exc);
}
					}
				}.start();
			};
		});

		final JButton button = new JButton("Create Images");
		button.addActionListener( new ActionListener()
		{
			public void actionPerformed(ActionEvent e)
			{
				try
				{
					System.out.println("Creating desktop.jpg");
					ScreenImage.createDesktopImage( "c:\\desktop.jpg" );
					System.out.println("Creating frame.jpg");
					ScreenImage.createImage(frame, "c:\\frame.jpg");
					System.out.println("Creating scrollpane.jpg");
					ScreenImage.createImage(scrollPane,
"c:\\scrollpane.jpg");
					System.out.println("Creating textarea.jpg");
					ScreenImage.createImage(textArea, "c:\\textarea.jpg");
```

```
                        System.out.println("Creating button.jpg");
                        ScreenImage.createImage(button, "c:\\button.jpg");
                        button.setText("button refreshed");
                        button.paintImmediately(button.getBounds());
                        System.out.println("Creating refresh.jpg");
                        ScreenImage.createImage(button, "c:\\refresh.jpg");
                        System.out.println("Creating region.jpg");
                        Rectangle r = new Rectangle(0, 0, 100, 16);
                        ScreenImage.createImage(textArea, r, "c:\\region.png");
                    }
                    catch(Exception exc) { System.out.println(exc); }
                }
            });
            frame.getContentPane().add(button, BorderLayout.SOUTH);

            try
            {
                FileReader fr = new FileReader( "ScreenImage.java" );
                BufferedReader br = new BufferedReader(fr);
                textArea.read( br, null );
                br.close();
            }
            catch(Exception e) {}

            frame.setDefaultCloseOperation(JFrame.EXIT_ON_CLOSE);
            frame.pack();
            frame.setLocationRelativeTo( null );
            frame.setVisible(true);
    }
}
```

Src 27 : SDSOutputConverter.java

```
/**
Subclass of OutputConverter to handle the SDS format
Log File Aggregation (LFA) tool V1.1.

Copyright (C) 2008 Biomedical Informatics (BMI), St George's University of London. Citations to
the toolkit should be as 'de Lusignan S, Kumarapeli P, Chan T, Pflug B, van Vlymen J, Jones B,
Freeman GK. The ALFA (activity Log Files Aggregation) toolkit: a method for precise observation
of the consultation. <J Med Internet Res 2008; ..>.

This program is free software: you can redistribute it and/or modify it under the terms of the
GNU General Public License as published by the Free Software Foundation, either version 3 of the
License, or (at your option) any later version.

This program is distributed in the hope that it will be useful, but WITHOUT ANY WARRANTY;
without even the implied warranty of MERCHANTABILITY or FITNESS FOR A PARTICULAR
PURPOSE.  See the GNU General Public License for more details.
```

```
You should have received a copy of the GNU General Public License along with this program.  If
not, see <http://www.gnu.org/licenses/>.

*/

package aggregationtool;

import java.util.Iterator;
import java.util.zip.DataFormatException;


public class SDSOutputConverter extends OutputConverter{

   //*** Property Panel components ***
   //TODO add all components to be displayed in property panel

   //*************************************************************************
   //***************** constructor/building methods ***************************
   //*************************************************************************

   public SDSOutputConverter() {

      super();

      createPropertyPanel();
   }

   /**
    * creates a JPanel with all required properties for creating an SDS file
    */
   protected void createPropertyPanel() {

      //Uncomment code below in case to add settings for output
      /*
      //*** Panel ***
      propertyPanel = new JPanel();

      propertyPanel.setLayout( null );

      //TODO add properties
      */
   }

   //*************************************************************************
   //************************* operating methods ******************************
   //*************************************************************************

   /**
    * creates output string of data entries depending on SDS format
    * @throws DataFormatException in any case an error occurs
    */
   public void convertSource() throws DataFormatException {
```

Biomedical Informatics, Division of Community Health Sciences
Tel + 44 (0)20 8725 5661 Fax + 44 (0)20 8725 3584 Email slusigna@sgul.ac.uk

```
if (dataSource != null) {

    //******** process header *********
    //define unique values
    outputString = "Timed ";
    Object [] uniqueValues = DataEntry.getUniqueEventValues(dataSource);

    for (int i =0; i < uniqueValues.length-2; i++) {
        outputString += uniqueValues[i]+" ";
    }

    if (uniqueValues.length>0) {
        outputString += uniqueValues[uniqueValues.length-1];
    }

    outputString += ";\n";

    //add timeunit
    //!! global timeumit is set, in case timeunits would differ, change also this definition
    outputString +=  ","+LogConverter.GLOBAL_TIMEUNIT+"\n";

    //******** process content ********
    Iterator it = dataSource.iterator();
    int entryInLineCounter=0;

    while (it.hasNext()) {
        DataEntry de = (DataEntry)it.next();

        if (de instanceof EventEntry) {
            EventEntry ee = (EventEntry)de;

            entryInLineCounter++;

            String entryString;

            //add value
            entryString = ee.getEvent().getValue()+",";

            //add time(s) depending on duration or event
            if (ee.isDuration()) {
                entryString += ee.StartTime + "-" + ee.EndTime+")";
            }
            else {
                entryString += ee.StartTime;
            }

            //in case more then five entries in one line, create newline, otherwise space
            if (entryInLineCounter >=5) {
                entryString += "\n";
                entryInLineCounter=0;
            }
            else {
                entryString += " ";
```

```
            }

            //add entry string to whole output string
            outputString +=entryString;
          }
        }

        //******** process end ********

        //in case last charakter wosn't already a new line
        if (entryInLineCounter != 0) {
          outputString += "\n";
        }

        //include highest time at the end
        float [] highestTimes = DataEntry.getHighestTimes(dataSource);

        //if highest starttime is higher then highest endtime
        if (highestTimes[0] > highestTimes[1]) {
          outputString += ","+highestTimes[0]+")\n";
        }
        else {
          outputString += ","+highestTimes[1]+")\n";
        }

        //add slash at the end
        outputString += "/";
      }
      else {
        throw new DataFormatException("no data source set");
      }
    }
  }
```

Src 28 : SearchDialog.java

```
/**
This class is used by Tabuluar output dialog to search for specified attributes
Log File Aggregation (LFA) tool V1.1.

Copyright (C) 2008 Biomedical Informatics (BMI), St George's University of London. Citations to
the toolkit should be as 'de Lusignan S, Kumarapeli P, Chan T, Pflug B, van Vlymen J, Jones B,
Freeman GK. The ALFA (activity Log Files Aggregation) toolkit: a method for precise observation
of the consultation. <J Med Internet Res 2008; ..>.

This program is free software: you can redistribute it and/or modify it under the terms of the
GNU General Public License as published by the Free Software Foundation, either version 3 of the
License, or (at your option) any later version.
```

This program is distributed in the hope that it will be useful, but WITHOUT ANY WARRANTY; without even the implied warranty of MERCHANTABILITY or FITNESS FOR A PARTICULAR PURPOSE. See the GNU General Public License for more details.

You should have received a copy of the GNU General Public License along with this program. If not, see <http://www.gnu.org/licenses/>.

```
*/

package aggregationtool;

import java.awt.Dimension;
import java.awt.Font;

import java.awt.Rectangle;

import java.awt.event.ActionEvent;
import java.awt.event.ActionListener;

import java.awt.event.KeyEvent;

import java.util.ArrayList;

import java.util.Iterator;

import javax.swing.ButtonGroup;
import javax.swing.JButton;
import javax.swing.JCheckBox;
import javax.swing.JDialog;
import javax.swing.JLabel;
import javax.swing.JRadioButton;
import javax.swing.JSeparator;
import javax.swing.JTextField;

public class SearchDialog extends JDialog
implements ActionListener{

    private ArrayList DataSource;
    private ArrayList SearchResult;

    //instance of errorDialog which is shown any time errors occur
    private ErrorDialog errDialog;

    private GUISuperclass superclass;

    //This variable summerize all displayed results for the title of the Dialog
    private static int searchResultCounter;

    //**********GUI ELEMENTS*************
    //Starttime
    private JCheckBox starttime_check = new JCheckBox();
    private JSeparator jSeparator1 = new JSeparator();
    private JLabel starttime_label = new JLabel();
```

```
    private JTextField starttime_text = new JTextField();
    private ButtonGroup starttime_endvaluegroup = new ButtonGroup();
    private JRadioButton starttime_no_duration = new JRadioButton("EXACT TIME - NO
DURATION",true);
    private JRadioButton starttime_onwards = new JRadioButton("FROM STARTTIME
ONWARDS");
    private JRadioButton starttime_backwards = new JRadioButton("FROM STARTTIME
BACKWARDS");
    private JRadioButton starttime_endvalue = new JRadioButton("ENDVALUE:");
    private JTextField starttime_endvaluetext = new JTextField();

    //Endtime
    private JCheckBox endtime_check = new JCheckBox();
    private JSeparator jSeparator2 = new JSeparator();
    private JLabel endtime_label = new JLabel();
    private JTextField endtime_text = new JTextField();
    private ButtonGroup endtime_endvaluegroup = new ButtonGroup();
    private JRadioButton endtime_no_duration = new JRadioButton ("EXACT TIME - NO
DURATION",true);
    private JRadioButton endtime_onwards = new JRadioButton("FROM ENDTIME ONWARDS");
    private JRadioButton endtime_backwards = new JRadioButton("FROM ENDTIME
BACKWARDS");
    private JRadioButton endtime_endvalue = new JRadioButton("ENDVALUE:");
    private JTextField endtime_endvaluetext = new JTextField();

    //Event
    private JCheckBox event_check = new JCheckBox();
    private JLabel event_label = new JLabel();
    private JTextField event_text = new JTextField();
    private ButtonGroup event_buttongroup = new ButtonGroup();
    private JRadioButton event_completeMatch = new JRadioButton("COMPLETE MATCH",true);
    private JRadioButton event_partialMatch = new JRadioButton("PARTIAL MATCH");
    private ButtonGroup event_buttongroup_2 = new ButtonGroup();
    private JRadioButton event_SearchBoth = new JRadioButton("SEARCH BOTH",true);
    private JRadioButton event_SearchType = new JRadioButton("SEARCH IN TYPE");
    private JRadioButton event_SearchValue = new JRadioButton("SEARCH IN VALUE");

    //Search buttons
    private JButton search_button = new JButton();
    private JButton clear_button = new JButton();
    private JSeparator jSeparator3 = new JSeparator();


    public SearchDialog(GUISuperclass parent, String title, boolean modal,ArrayList DataSource) {
        super(parent, title, modal);

        superclass = parent;

        this.DataSource = DataSource;

        this.errDialog = new ErrorDialog(superclass,"output of search dialog",true);

        try {
```

```
            jbInit();
        } catch (Exception e) {
            e.printStackTrace();
        }
    }

    /**
     * initialise GUI
     * @throws Exception
     */
    private void jbInit() throws Exception {
        this.setSize(new Dimension(620, 500));
        this.getContentPane().setLayout( null );

        //*********
        //STARTTIME
        //*********
        starttime_check.setText("START TIME");
        starttime_check.setActionCommand("start");
        starttime_check.setSelected(true);
        starttime_check.setBounds(new Rectangle(15, 15, 110, 35));
        starttime_check.setFont(new Font("Tahoma", 1, 12));
        starttime_check.addActionListener(this);

        jSeparator1.setBounds(new Rectangle(0, 135, 645, 15));

        starttime_label.setText("ENTER STARTVALUE");
        starttime_label.setBounds(new Rectangle(155, 10, 135, 25));

        starttime_text.setBounds(new Rectangle(155, 40, 100, 20));

        //Radiobuttons
        starttime_no_duration.setActionCommand("no_dur");
        starttime_onwards.setActionCommand("onwards");
        starttime_backwards.setActionCommand("backwards");
        starttime_endvalue.setActionCommand("value");
        starttime_endvaluegroup.add(starttime_no_duration);
        starttime_endvaluegroup.add(starttime_onwards);
        starttime_endvaluegroup.add(starttime_backwards);
        starttime_endvaluegroup.add(starttime_endvalue);
        starttime_no_duration.setBounds(350,15,200,20);
        starttime_onwards.setBounds(350,45,200,20);
        starttime_backwards.setBounds(350,75,200,20);
        starttime_endvalue.setBounds(350,105,90,20);

        //Endvalue textfield
        starttime_endvaluetext.setBounds(new Rectangle(440, 105, 100, 20));

        this.getContentPane().add(starttime_text, null);
        this.getContentPane().add(starttime_label, null);
        this.getContentPane().add(jSeparator1, null);
        this.getContentPane().add(starttime_check, null);
        this.getContentPane().add(starttime_no_duration);
```

```
this.getContentPane().add(starttime_onwards);
this.getContentPane().add(starttime_backwards);
this.getContentPane().add(starttime_endvalue);
this.getContentPane().add(starttime_endvaluetext);
this.getContentPane().add(endtime_text, null);

//*******
//ENDTIME
//*******
endtime_check.setText("END TIME");
endtime_check.setActionCommand("end");
endtime_check.setSelected(true);
endtime_check.setBounds(new Rectangle(15, 150, 110, 35));
endtime_check.setFont(new Font("Tahoma", 1, 12));
endtime_check.addActionListener(this);

jSeparator2.setBounds(new Rectangle(0, 270, 645, 15));

endtime_label.setText("ENTER ENDVALUE");
endtime_label.setBounds(new Rectangle(155, 145, 135, 25));

endtime_text.setBounds(new Rectangle(155, 175, 100, 20));

//Radiobuttons
endtime_no_duration.setActionCommand("no_dur");
endtime_onwards.setActionCommand("onwards");
endtime_backwards.setActionCommand("backwards");
endtime_endvalue.setActionCommand("value");
endtime_endvaluegroup.add(endtime_no_duration);
endtime_endvaluegroup.add(endtime_onwards);
endtime_endvaluegroup.add(endtime_backwards);
endtime_endvaluegroup.add(endtime_endvalue);
endtime_no_duration.setBounds(350, 150, 200, 20);
endtime_onwards.setBounds(350, 180, 200, 20);
endtime_backwards.setBounds(350, 210, 200, 20);
endtime_endvalue.setBounds(350, 240, 90, 20);

//Endvalue textfield
endtime_endvaluetext.setBounds(new Rectangle(440, 240, 100, 20));

this.getContentPane().add(endtime_label, null);
this.getContentPane().add(jSeparator2, null);
this.getContentPane().add(endtime_check, null);
this.getContentPane().add(endtime_no_duration);
this.getContentPane().add(endtime_onwards);
this.getContentPane().add(endtime_backwards);
this.getContentPane().add(endtime_endvalue);

//*****
//EVENT
//*****
this.getContentPane().add(endtime_endvaluetext);
event_check.setText("EVENT");
```

```
event_check.setActionCommand("event");
event_check.setSelected(true);
event_check.setBounds(new Rectangle(15, 280, 110, 35));
event_check.setFont(new Font("Tahoma", 1, 12));
event_check.addActionListener(this);

event_label.setBounds(155,290,200,20);
event_label.setText("TEXT");

event_label.setBounds(new Rectangle(155, 280, 135, 20));
event_text.setBounds(155,310,100,20);

event_completeMatch.setBounds(300,285,150,20);
event_completeMatch.setActionCommand("complete");
event_buttongroup.add(event_completeMatch);

event_partialMatch.setBounds(300,315,150,20);
event_partialMatch.setActionCommand("partial");
event_buttongroup.add(event_partialMatch);

event_SearchBoth.setBounds(460,285,150,20);
event_SearchBoth.setActionCommand("both");
event_buttongroup_2.add(event_SearchBoth);

event_SearchType.setBounds(460,315,150,20);
event_SearchType.setActionCommand("type");
event_buttongroup_2.add(event_SearchType);

event_SearchValue.setBounds(460,345,150,20);
event_SearchValue.setActionCommand("value");
event_buttongroup_2.add(event_SearchValue);

this.getContentPane().add(event_check);
this.getContentPane().add(event_label);
this.getContentPane().add(event_text);
this.getContentPane().add(event_completeMatch);
this.getContentPane().add(event_partialMatch);
this.getContentPane().add(event_SearchBoth);
this.getContentPane().add(event_SearchType);
this.getContentPane().add(event_SearchValue);

//**************
//search options
//**************

this.getContentPane().add(jSeparator3, null);
this.getContentPane().add(search_button, null);
this.getContentPane().add(clear_button);
search_button.setText("Start search...");
search_button.setBounds(new Rectangle(145, 420, 115, 25));
search_button.setMnemonic(KeyEvent.VK_S);
search_button.setActionCommand("search");
search_button.addActionListener(this);
```

```
        jSeparator3.setBounds(new Rectangle(0, 390, 645, 2));

        clear_button.setText("Default");
        clear_button.setBounds(300, 370, 105, 25);
        clear_button.setMnemonic(KeyEvent.VK_D);
        clear_button.setActionCommand("clear");
        clear_button.setBounds(new Rectangle(370, 420, 105, 25));
        clear_button.addActionListener(this);


    }

    /**
     * Is called for each component of the Dialog, for en-disable actions and search actions
     * @param e
     */
    public void actionPerformed(ActionEvent e) {

        String command = e.getActionCommand();

        //Checkbox clicked - ENABLE und DISABLE ELEMENTS
        if (e.getSource() instanceof JCheckBox) {

            if (command.equals("start")) {
                //En- disable all items depending on the checkbox
                boolean enabled = ((JCheckBox)e.getSource()).isSelected();

                starttime_label.setEnabled(enabled);
                starttime_text.setEnabled(enabled);
                starttime_no_duration.setEnabled(enabled);
                starttime_onwards.setEnabled(enabled);
                starttime_backwards.setEnabled(enabled);
                starttime_endvaluetext.setEnabled(enabled);
                starttime_endvalue.setEnabled(enabled);
            }
            else if (command.equals("end")) {
                //En- disable all items depending on the checkbox
                boolean enabled = ((JCheckBox)e.getSource()).isSelected();

                endtime_label.setEnabled(enabled);
                endtime_text.setEnabled(enabled);
                endtime_no_duration.setEnabled(enabled);
                endtime_onwards.setEnabled(enabled);
                endtime_backwards.setEnabled(enabled);
                endtime_endvaluetext.setEnabled(enabled);
                endtime_endvalue.setEnabled(enabled);
            }
            else if (command.equals("event")) {
                //En- disable all items depending on the checkbox
                boolean enabled = ((JCheckBox)e.getSource()).isSelected();

                event_label.setEnabled(enabled);
```

```
            event_text.setEnabled(enabled);
            event_completeMatch.setEnabled(enabled);
            event_partialMatch.setEnabled(enabled);
            event_SearchBoth.setEnabled(enabled);
            event_SearchType.setEnabled(enabled);
            event_SearchValue.setEnabled(enabled);
        }
        else {
            System.out.println("SearchDialog:actionPerformed:JCheckBox: wrong command");
        }
    }
    //One of the buttons were clicked
    else if (e.getSource() instanceof JButton) {
        if (command.equals("search")) {

            process_search();

            //No errors ocurred, display new Result
            if (errDialog.containsOnlyInfos()) {
                //close this search dialog
                this.setVisible(false);

                //increase searchResult counter
                searchResultCounter++;
                //create new tabular display dialog
                TabularDisplayDialog displaydialog = new TabularDisplayDialog(superclass,"search
result "+searchResultCounter,false,this.SearchResult,true);

                //add dialog to dialoglist in display panel
                DisplayPanel.addDialogToDialogList(displaydialog);

                //display new dialog
                displaydialog.setVisible(true);
            }
            else {
                errDialog.showErrDialog();
            }
        }
        else if (command.equals("clear")) {
            //Set all to default values

            starttime_check.setSelected(true);
            //dirty
            actionPerformed(new
ActionEvent(starttime_check,0,starttime_check.getActionCommand()));
            starttime_text.setText("");
            starttime_no_duration.setSelected(true);
            starttime_endvaluetext.setText("");

            endtime_check.setSelected(true);
            //dirty
            actionPerformed(new
ActionEvent(endtime_check,0,endtime_check.getActionCommand()));
```

```
            endtime_text.setText("");
            endtime_no_duration.setSelected(true);
            endtime_endvaluetext.setText("");

            event_check.setSelected(true);
            //dirty
            actionPerformed(new
ActionEvent(event_check,0,event_check.getActionCommand()));
            event_text.setText("");
            event_completeMatch.setSelected(true);
        }
    }
}

//*********************************
//***** methods for processing *****
//*********************************

/**
 * This method processes the search
 */
private void process_search() {

    //initialise result list with all data from source
    SearchResult = new ArrayList();

    //remove all errorDialog content
    errDialog.removeAllMsg();

    //check all selected options

    //search for starttime
    if (starttime_check.isSelected()) {

        errDialog.addMessage("STARTTIME...",ErrorDialog.INFO);

        float starttime_startvalue;

        try {
            //get value for starttime
            starttime_startvalue = Float.parseFloat(this.starttime_text.getText());

            String starttime_action =
starttime_endvaluegroup.getSelection().getActionCommand();

            //Go throw list
            Iterator it = DataSource.iterator();

            //if endvalue is selected, get Endvalue from textfield
            float starttime_endvalue=0;

            if (starttime_action.equals("value")) {
                starttime_endvalue = Float.parseFloat(this.starttime_endvaluetext.getText());
```

```
                //if starttime is higher or equal endtime, throw exception
                if (starttime_startvalue >= starttime_endvalue) {
                    throw new Exception();
                }
            }

            while (it.hasNext()) {

                DataEntry de = (DataEntry)it.next();

                //If entry is headerinformation, add it anyway
                if (de instanceof HeaderEntry) {
                    SearchResult.add(de);
                }
                else if (de instanceof EventEntry) {

                    EventEntry ee = (EventEntry)de;

                    //depending on choosen option compare starttime of entry with value from GUI
                    if (starttime_action.equals("no_dur")) {

                        //compare starttime with given value
                        if (ee.StartTime==starttime_startvalue) {
                            SearchResult.add(ee);
                        }
                    }
                    else if (starttime_action.equals("onwards")) {
                        if (ee.StartTime >= starttime_startvalue) {
                            SearchResult.add(ee);
                        }
                    }
                    else if (starttime_action.equals("backwards")) {
                        if (ee.StartTime <= starttime_startvalue) {
                            SearchResult.add(ee);
                        }
                    }
                    else if (starttime_action.equals("value")) {
                        if (ee.StartTime <= starttime_endvalue && ee.StartTime >=
starttime_startvalue) {
                            SearchResult.add(ee);
                        }
                    }
                }
                else {
                    //Wrong class in list
                    errDialog.addMessage("Entry in Result not instance of Header or Event info,
Entry ignorend",ErrorDialog.WARNING);
                }
            }
        }
        catch (NumberFormatException ex) {
            errDialog.addMessage("value in textfield is not a number",ErrorDialog.ERROR);
```

```
            }
            catch (Exception ex2) {
                errDialog.addMessage("startvalue is higher or equal to endvalue",ErrorDialog.ERROR);
            }
        }

        //search for endtime
        if (endtime_check.isSelected()) {

            errDialog.addMessage("ENDTIME...",ErrorDialog.INFO);

            ArrayList Source;
            //This arraylist saves the information of the endresult, afterwards it overwrites the
SearchResult
            ArrayList endtimeResult = new ArrayList();

            float endtime_startvalue;

            //Depending on whether starttime was already calculated, the source for the search is
defined
            if (starttime_check.isSelected()) {
                Source = SearchResult;
            }
            else {
                Source = DataSource;
            }

            try {
                //get value for endtime
                endtime_startvalue = Float.parseFloat(endtime_text.getText());

                String endtime_action = endtime_endvaluegroup.getSelection().getActionCommand();

                //Go throw list
                Iterator it = Source.iterator();

                //if endvalue is selected, get Endvalue from textfield
                float endtime_endvalue=0;

                if (endtime_action.equals("value")) {
                    endtime_endvalue = Float.parseFloat(endtime_endvaluetext.getText());

                    //if starttime is higher or equal endtime, throw exception
                    if (endtime_startvalue >= endtime_endvalue) {
                        throw new Exception();
                    }
                }

                while (it.hasNext()) {

                    DataEntry de = (DataEntry)it.next();

                    //if entry is instance of headerinformation, add it anyway
```

```
                if (de instanceof HeaderEntry) {
                    endtimeResult.add(de);
                }
                else if (de instanceof EventEntry) {

                    EventEntry ee = (EventEntry)de;

                    //depending on choosen option compare dataentry value with value from GUI
                    if (endtime_action.equals("no_dur")) {

                        //compare starttime with given value
                        if (ee.EndTime==endtime_startvalue) {
                            endtimeResult.add(ee);
                        }
                    }
                    else if (endtime_action.equals("onwards")) {
                        if (ee.EndTime >= endtime_startvalue) {
                            endtimeResult.add(ee);
                        }
                    }
                    else if (endtime_action.equals("backwards")) {
                        if (ee.EndTime <= endtime_startvalue) {
                            endtimeResult.add(ee);
                        }
                    }
                    else if (endtime_action.equals("value")) {
                        if (ee.EndTime <= endtime_endvalue && ee.EndTime >=
endtime_startvalue) {
                            endtimeResult.add(ee);
                        }
                    }
                }
                else {
                    //Wrong class in list
                    errDialog.addMessage("Entry in Result not instance of Header or Event info,
Entry ignorend",ErrorDialog.WARNING);
                }
            }

            //Now the new results are stored in endtimeResult, so they have to be copied to
SearchResult
            //Before the SearchResult must be cleared
            SearchResult.clear();
            SearchResult.addAll(endtimeResult);
        }
        catch (NumberFormatException ex) {
            errDialog.addMessage("value in textfield is not a number",ErrorDialog.ERROR);
        }
        catch (Exception ex2) {
            errDialog.addMessage("startvalue is higher or equal to endvalue",ErrorDialog.ERROR);
        }
    }
```

```
        //search for Event
        if (event_check.isSelected()) {

            errDialog.addMessage("EVENT...",ErrorDialog.INFO);

            ArrayList Source;
            ArrayList eventSearch = new ArrayList();

            //if one of them is selected, use as source the result of there output
            if (starttime_check.isSelected() || endtime_check.isSelected()) {
                Source = SearchResult;
            }
            else {
                //Otherwise us the orignial dataSource
                Source = DataSource;
            }

            try {
                String eventText = event_text.getText();

                if (eventText.length()==0) {
                    throw new Exception("Error at Event: event text is empty");
                }

                Iterator it = Source.iterator();

                while (it.hasNext()) {

                    DataEntry de = (DataEntry)it.next();

                    //if entry instance of Headerentry, add it anyway
                    if (de instanceof HeaderEntry) {
                        eventSearch.add(de);
                    }
                    else if (de instanceof EventEntry) {
                        EventEntry ee = (EventEntry)de;

                        String event_action =
this.event_buttongroup.getSelection().getActionCommand();
                        String event_searchIn =
this.event_buttongroup_2.getSelection().getActionCommand();

                        //depending on choosen option
                        if (event_action.equals("complete")) {
                            if (event_searchIn.equals("both")) {
                                //If event completely matches searchstring, add
                                if (ee.getEvent().toString().equalsIgnoreCase(eventText)) {
                                    eventSearch.add(ee);
                                }
                            }
                            else if (event_searchIn.equals("type")) {
                                //If event type compeletly matches searchstring,add
                                if (ee.getEvent().getType().toString().equalsIgnoreCase(eventText)) {
```

```
                        eventSearch.add(ee);
                    }
                }
                else if (event_searchIn.equals("value")) {
                    //If event value compeletly matches searchstring,add
                    if (ee.getEvent().getValue().toString().equalsIgnoreCase(eventText)) {
                        eventSearch.add(ee);
                    }
                }
            }
            else if (event_action.equals("partial")) {
                if (event_searchIn.equals("both")) {
                    if
(ee.getEvent().toString().toLowerCase().indexOf(eventText.toLowerCase()) !=-1) {
                        eventSearch.add(ee);
                    }
                }
                else if (event_searchIn.equals("type")) {
                     if
(ee.getEvent().getType().toString().toLowerCase().indexOf(eventText.toLowerCase()) !=-1) {
                         eventSearch.add(ee);
                     }
                }
                else if (event_searchIn.equals("value")) {
                     if
(ee.getEvent().getValue().toString().toLowerCase().indexOf(eventText.toLowerCase()) !=-1) {
                         eventSearch.add(ee);
                     }
                }
            }
        }
        else {
            errDialog.addMessage("Entry in Result not instance of Header or Event info,
Entry ignorend",ErrorDialog.WARNING);
        }
    }

    //Same like endtime, remove all previous results and add new
    SearchResult.clear();
    SearchResult.addAll(eventSearch);
   }
   catch (Exception ex) {
    errDialog.addMessage(ex.getMessage(),ErrorDialog.ERROR);
   }
  }
 }
}
```

Src 29 : SettingsPanel.java

```
/**
```

```
defines the initial step by choosing the number of files to read in and optional define a data
source for video file to link with
Log File Aggregation (LFA) tool V1.1.

Copyright (C) 2008 Biomedical Informatics (BMI), St George's University of London. Citations to
the toolkit should be as 'de Lusignan S, Kumarapeli P, Chan T, Pflug B, van Vlymen J, Jones B,
Freeman GK. The ALFA (activity Log Files Aggregation) toolkit: a method for precise observation
of the consultation. <J Med Internet Res 2008; ..>.

This program is free software: you can redistribute it and/or modify it under the terms of the
GNU General Public License as published by the Free Software Foundation, either version 3 of the
License, or (at your option) any later version.

This program is distributed in the hope that it will be useful, but WITHOUT ANY WARRANTY;
without even the implied warranty of MERCHANTABILITY or FITNESS FOR A PARTICULAR
PURPOSE.  See the GNU General Public License for more details.

You should have received a copy of the GNU General Public License along with this program.  If
not, see <http://www.gnu.org/licenses/>.

*/

package aggregationtool;

import java.awt.Font;
import java.awt.Rectangle;
import java.awt.event.ActionEvent;
import java.awt.event.ActionListener;

import java.awt.event.KeyEvent;

import java.io.File;

import javax.swing.JButton;
import javax.swing.JCheckBox;
import javax.swing.JComboBox;
import javax.swing.JFileChooser;
import javax.swing.JLabel;
import javax.swing.JPanel;
import javax.swing.JTextField;


public class SettingsPanel extends JPanel
implements StepPanelInterface{

    //Reference to superclass
    GUISuperclass superclass;

    //settings for the video file
    private VideoSettings vs;

    //Buttons for navigation
    private JButton next_button = new JButton();
```

```
    private JButton prev_button = new JButton();

    //Label with headerinfo
    private JLabel jLabel1 = new JLabel();

    //combo box for selecting the number of files
    private JComboBox jComboBox1 = new JComboBox();

    //CheckBox whether video file should be linked
    private JCheckBox videoCheck = new JCheckBox();

    //Label for videopath
    private JLabel jLabel2 = new JLabel();

    //Textfield for filepath
    private JTextField videoPathField = new JTextField();

    //browse button
    private JButton browseButton = new JButton();

    //show video button
    private JButton showVideo = new JButton();

    //Label for rate setting
    private JLabel rateLabel = new JLabel();

    //Combo box for selection
    private JComboBox rateCombo = new JComboBox();


    public SettingsPanel(GUISuperclass superclass) {
        this.superclass=superclass;

        try {
            jbInit();
        } catch (Exception e) {
            e.printStackTrace();
        }
    }

    /**
     * Initialises the layout of the panel
     * @throws Exception
     */
    private void jbInit() throws Exception {
        this.setLayout( null );

         //next_button
         next_button.setText("next");
         next_button.setBounds(new Rectangle(550, 380, 80, 30));
         next_button.setMnemonic(KeyEvent.VK_N);
         next_button.setToolTipText("next step");
         next_button.addActionListener(new ActionListener() {
```

```
            public void actionPerformed(ActionEvent e) {
                display_next();
            }
});

//prev_button
 prev_button.setText("prev");
 prev_button.setBounds(460,380,80,30);
 prev_button.setMnemonic(KeyEvent.VK_P);
 prev_button.setToolTipText("previous step");
 prev_button.addActionListener(new ActionListener() {
            public void actionPerformed(ActionEvent e) {
                display_previous();
            }
});

//Label with headerinfo
jLabel1.setText("Please select the number of log-files you want to read in");
jLabel1.setBounds(new Rectangle(15, 60, 375, 30));
jLabel1.setFont(new Font("Times New Roman", 1, 14));

//Combo Box
jComboBox1.setBounds(new Rectangle(15, 110, 185, 20));

for (int i=1; i <= 10; i++)
   jComboBox1.addItem(new ComboItem(i));

//Checkbox for videofile
videoCheck.setSelected(false);
videoCheck.setText("Link to videofile");
videoCheck.setFont(new Font("Times New Roman", 1, 14));
videoCheck.setBounds(15,150,150,30);
videoCheck.addActionListener(new ActionListener() {
        public void actionPerformed(ActionEvent e) {
            jLabel2.setEnabled(videoCheck.isSelected());
            videoPathField.setEnabled(videoCheck.isSelected());
            browseButton.setEnabled(videoCheck.isSelected());
            showVideo.setEnabled(videoCheck.isSelected());
            rateLabel.setEnabled(videoCheck.isSelected());
            rateCombo.setEnabled(videoCheck.isSelected());
        }
    }
);

//Label for videopath
jLabel2.setText("Select path for video file in order to link data with");
jLabel2.setBounds(new Rectangle(15, 180, 375, 30));
jLabel2.setFont(new Font("Times New Roman", 1, 14));
jLabel2.setEnabled(videoCheck.isSelected());

//TextField for videopath
videoPathField.setBounds(15,220,300,20);
videoPathField.setEnabled(videoCheck.isSelected());
```

```
        //BrowseButton
        browseButton.setBounds(330,220,110,20);
        browseButton.setText("Browse...");
        browseButton.setEnabled(videoCheck.isSelected());
        browseButton.addActionListener(new ActionListener() {
              public void actionPerformed(ActionEvent e) {

                 JFileChooser fc = new JFileChooser();

                 //if global file directory set, init with remembered dir
                 if (LogAggregationTool.isFileDirSet()) {
                    fc.setCurrentDirectory(new File(LogAggregationTool.getFileDir()));
                 }


                 fc.setFileFilter(new VideoFilter());

                 int returnVal = fc.showOpenDialog(superclass);

                 if (returnVal == JFileChooser.APPROVE_OPTION) {

                    videoPathField.setText(fc.getSelectedFile().getPath());
                 }

                 //set global file dir
                 LogAggregationTool.setFileDir(fc.getCurrentDirectory().getPath());
              }
           }
        );

        //showVideo
        showVideo.setBounds(330,300,110,20);
        showVideo.setText("Show video...");
        showVideo.setEnabled(videoCheck.isSelected());
        showVideo.addActionListener(new ActionListener() {
              public void actionPerformed(ActionEvent e) {
                 //set properties
                  vs =
VideoSettings.createSettings(videoPathField.getText(),((Float)rateCombo.getSelectedItem()).float
Value());

                  if (vs.settingsOK()) {
                    //instance player
                    AggregationMediaPlayer amp =
LogAggregationTool.getAggregationMediaPlayer();

                    if (amp != null) {
                       amp.setProperties(vs);
                       amp.play();
                    }
                    else {
                       ErrorDialog er = new ErrorDialog(superclass,"MediaPlayer error",true);
```

```
                    er.addMessage("Unable to open player!",ErrorDialog.ERROR);
                    er.showErrDialog();
                }
            }
            else {
                ErrorDialog er = new ErrorDialog(superclass,"settings errors",true);
                er.addMessage("errors in videosettings, please resolve to
proceed",ErrorDialog.ERROR);
                er.showErrDialog();
            }
        }
    }
    );

    //rateLabel
    rateLabel.setText("Select rate for video");
    rateLabel.setBounds(new Rectangle(15,250,200,20));
    rateLabel.setFont(new Font("Times New Roman", 1, 14));
    rateLabel.setEnabled(videoCheck.isSelected());

    //rateCombo
    rateCombo.setBounds(15,275,50,20);

    for (int i =1; i<=20; i++) {
        rateCombo.addItem(new Float((float)i/10));
    }

    rateCombo.setSelectedIndex(9);
    rateCombo.setEnabled(videoCheck.isSelected());

    //add to contentPane
    this.add(jLabel2, null);
    this.add(videoCheck,null);
    this.add(showVideo,null);
    this.add(videoPathField,null);
    this.add(browseButton,null);
    this.add(rateLabel,null);
    this.add(rateCombo,null);
    this.add(jComboBox1, null);
    this.add(jLabel1, null);
    this.add(next_button, null);
    this.add(prev_button,null);
}

/**
 * @return the number of files selected in the combobox
 */
public int getNumberOfFiles() {
    return ((ComboItem)this.jComboBox1.getSelectedItem()).getValue();
}

/**
 * Method to get access to video settings from other panels
```

```
     * @return VideoSettings instance if set, null otherwise
     */
    public VideoSettings getInitialVideoSettings() {
        return vs;
    }

    /**
     * Display next step
     */
    public void display_next() {

        boolean proceed = true;

        //in case video checkbox is selected create settings from values of components
        if (videoCheck.isSelected()) {

            vs =
VideoSettings.createSettings(videoPathField.getText(),((Float)rateCombo.getSelectedItem()).float
Value());
            proceed = vs.settingsOK();
        }
        //all settings must be correct in order to proceed
        if (proceed) {
            this.setVisible(false);
            superclass.conversionpanel= new ConversionPanel(superclass);
            superclass.displayPanel(superclass.conversionpanel,3);
        }
        else {
            ErrorDialog er = new ErrorDialog(superclass,"settings errors",true);
            er.addMessage("errors in videosettings, please resolve to proceed",ErrorDialog.ERROR);
            er.showErrDialog();
        }
    }

    /**
     * Go back to previous step
     */
    public void display_previous(){
        this.setVisible(false);
        superclass.displayPanel(superclass.startpanel,1);
    }

    /**
     * Inner class for filtering only mpeg video files in video file browse dialog
     */
    private class VideoFilter extends javax.swing.filechooser.FileFilter{

        String [] filters = new String [] {"mpeg","mpg"};

            public VideoFilter() {
            }

            public boolean accept(File f) {
```

```
            if (f.isDirectory()) {
                return true;
            }
            else {
                for (int i =0; i< filters.length; i++) {
                    if (f.getName().toLowerCase().endsWith("."+filters[i])) {
                        return true;
                    }
                }
                return false;
            }
        }

        public String getDescription() {
            String des = "";

            for (int i =0; i< filters.length-1; i++) {
                des += "."+filters[i] + ",";
            }
            des += "."+filters[filters.length-1];

            return des;
        }
    }

    /**
     * Inner class for representing the items for selecting number of files in the comboBox
     * It makes it easier to get the selected number afterwards
     */
    public class ComboItem {
        public int i;

        public ComboItem(int i) {
            this.i=i;
        }

        public String toString(){
            if (i==1) {
                return "1 File";
            }
            else {
                return i+" Files";
            }
        }

        public int getValue() {
            return i;
        }
    }
}
```

Src 30: StartPanel.java

```
/**
First panel shown at program start to display basic information about the program
Log File Aggregation (LFA) tool V1.1.

Copyright (C) 2008 Biomedical Informatics (BMI), St George's University of London. Citations to
the toolkit should be as 'de Lusignan S, Kumarapeli P, Chan T, Pflug B, van Vlymen J, Jones B,
Freeman GK. The ALFA (activity Log Files Aggregation) toolkit: a method for precise observation
of the consultation. <J Med Internet Res 2008; ..>.

This program is free software: you can redistribute it and/or modify it under the terms of the
GNU General Public License as published by the Free Software Foundation, either version 3 of the
License, or (at your option) any later version.

This program is distributed in the hope that it will be useful, but WITHOUT ANY WARRANTY;
without even the implied warranty of MERCHANTABILITY or FITNESS FOR A PARTICULAR
PURPOSE.  See the GNU General Public License for more details.

You should have received a copy of the GNU General Public License along with this program.  If
not, see <http://www.gnu.org/licenses/>.

*/

package aggregationtool;

import java.awt.Color;

import java.awt.Font;
import java.awt.Rectangle;

import java.awt.event.ActionEvent;
import java.awt.event.ActionListener;

import java.awt.event.KeyEvent;

import javax.swing.JButton;
import javax.swing.JLabel;
import javax.swing.JPanel;
import javax.swing.JTextArea;


public class StartPanel extends JPanel
implements StepPanelInterface{

    //Reference to superclass
    private GUISuperclass superclass;

    //Button which is shown on bottom to go to next step
    private JButton next_button = new JButton();

    //Headerlabel
```

```
    private JLabel jLabel1 = new JLabel();

    //Area for some basic information
    private JTextArea jTextArea1 = new JTextArea();

    public StartPanel(GUISuperclass superclass) {
        this.superclass=superclass;

        try {
            jbInit();
        } catch (Exception e) {
            e.printStackTrace();
        }
    }

    /**
     * Initialises the layout of the panel
     * @throws Exception
     */
    private void jbInit() throws Exception {
        this.setLayout( null );

        //next_button
        next_button.setText("next");
        next_button.setBounds(new Rectangle(550, 380, 80, 30));
        next_button.setMnemonic(KeyEvent.VK_N);
        next_button.setToolTipText("next step");
        next_button.addActionListener(new ActionListener() {
                    public void actionPerformed(ActionEvent e) {
                        display_next();
                    }
                });


        //Define the layout and the content of the Label and the text area
        jLabel1.setText("Aggregation tool");
        jLabel1.setBounds(new Rectangle(15, 15, 200, 40));
        jLabel1.setFont(new Font("Tahoma", 1, 20));
        jTextArea1.setBounds(new Rectangle(20, 65, 610, 305));
        jTextArea1.setEnabled(false);
        jTextArea1.setText("Welcome to aggregation tool!\n\n" +
        "This tool allows you to convert files of different formats to one common format and file,\n"
+
        "to display the output in several ways and to save it to a new file in another format.\n" +
        "Further more it opens the possiblility to link the entries to a video source." +
        "\n\n" +
        "This tool is divided to several steps as shown on top.\n" +
        "In order to go to the next step, press the next button on the bottom of the screen.\n" +
        "Also it is possible to go back to each done step by clicking the prev button\n" +
        "or by pressing the button on top for the step you want to go for" +
        "\n\n\n" +
        "This software is produced by\n" +
        "Bernhard Pflug\n" +
```

```
        "UMIT (www.umit.at) - Austria\n" +
        "at Biomedical informatics department\n" +
        "St George's University of London");
        jTextArea1.setDisabledTextColor(Color.black);
        jTextArea1.setFont(new Font("Tahoma", 0, 13));

        //Add all components to the frame
        this.add(jTextArea1, null);
        this.add(jLabel1, null);
        this.add(next_button, null);
    }

    /**
     * Displays the panel of the next step
     */
    public void display_next() {
        this.setVisible(false);
        superclass.settingspanel= new SettingsPanel(superclass);
        superclass.displayPanel(superclass.settingspanel,2);
    }

    public void display_previous() {
        //Nothing to do
    }
}
```

***/**

Src 31 : StepPanelInterface.java

/**
interface for step panels to define coordinates and distances as well as necessary methods for navigation
Log File Aggregation (LFA) tool V1.1.

Copyright (C) 2008 Biomedical Informatics (BMI), St George’s University of London. Citations to the toolkit should be as ‘de Lusignan S, Kumarapeli P, Chan T, Pflug B, van Vlymen J, Jones B, Freeman GK. The ALFA (activity Log Files Aggregation) toolkit: a method for precise observation of the consultation. <J Med Internet Res 2008; ..>.

This program is free software: you can redistribute it and/or modify it under the terms of the GNU General Public License as published by the Free Software Foundation, either version 3 of the License, or (at your option) any later version.

This program is distributed in the hope that it will be useful, but WITHOUT ANY WARRANTY; without even the implied warranty of MERCHANTABILITY or FITNESS FOR A PARTICULAR PURPOSE. See the GNU General Public License for more details.

You should have received a copy of the GNU General Public License along with this program. If not, see <http://www.gnu.org/licenses/>.

```
*/

package aggregationtool;

public interface StepPanelInterface {
    public static int HIGH=420;
    public static int WIDE=650;
    public static int XLocation=10;
    public static int YLocation=55;

    public void display_next();

    public void display_previous();
}
```

***/**

Src 32 : TabularDisplayDialog.java

/**
Display output to show aggregated data in form of a table with a column for each attribute
Log File Aggregation (LFA) tool V1.1.

Copyright (C) 2008 Biomedical Informatics (BMI), St George's University of London. Citations to the toolkit should be as 'de Lusignan S, Kumarapeli P, Chan T, Pflug B, van Vlymen J, Jones B, Freeman GK. The ALFA (activity Log Files Aggregation) toolkit: a method for precise observation of the consultation. <J Med Internet Res 2008; ..>.

This program is free software: you can redistribute it and/or modify it under the terms of the GNU General Public License as published by the Free Software Foundation, either version 3 of the License, or (at your option) any later version.

This program is distributed in the hope that it will be useful, but WITHOUT ANY WARRANTY; without even the implied warranty of MERCHANTABILITY or FITNESS FOR A PARTICULAR PURPOSE. See the GNU General Public License for more details.

You should have received a copy of the GNU General Public License along with this program. If not, see <http://www.gnu.org/licenses/>.

```
*/

package aggregationtool;

import java.awt.Dimension;

import java.awt.GridBagConstraints;

import java.awt.GridBagLayout;
```

```
import java.awt.Insets;

import java.awt.event.ActionEvent;
import java.awt.event.ActionListener;

import java.awt.event.KeyEvent;

import java.util.ArrayList;

import javax.swing.JButton;
import javax.swing.JDialog;
import javax.swing.JLabel;
import javax.swing.JScrollPane;
import javax.swing.JTable;
import javax.swing.JTextField;
import javax.swing.ListSelectionModel;
import javax.swing.table.DefaultTableModel;


public class TabularDisplayDialog extends JDialog
{

    //Parent
    GUISuperclass superclass;

    //ArrayList with DataEntries
    public ArrayList DataSource;

    //Boolean if dialog is shown for a searchResult
    boolean isSearchResult;

    //JTables
    private DefaultTableModel dataTableModel;
    private JTable dataTable = new JTable(){
        //overwrite to set all cells editable false
        public boolean isCellEditable(int row,int col) {
            return false;
        }
    };

    private DefaultTableModel headerTableModel;
    private JTable headerTable = new JTable() {
        //overwrite to set all cells editable false
        public boolean isCellEditable(int row,int col) {
            return false;
        }
    };

    //Textfield for searchText
    JTextField searchText;

    //Instance of GridBagLayout
    private GridBagLayout layout;
```

```
    public TabularDisplayDialog(GUISuperclass parent, String title, boolean modal,ArrayList
DataSource,boolean isSearchResult) {
        super(parent, title, modal);

        this.superclass = parent;
        this.DataSource = DataSource;
        this.isSearchResult = isSearchResult;

        //add DisplayClosingAdapter
        this.addDisplayDialogClosingAdapter();

        try {
            //Initialise GUI
            jbInit();

            //fill tables with given data
            this.fillTables();

        } catch (Exception e) {
            e.printStackTrace();
        }
    }

    /**
     * initialise GUI
     * @throws Exception
     */
    private void jbInit() throws Exception {
        this.setSize(new Dimension(511, 570));
        this.layout = new GridBagLayout();
        this.setLayout( layout );

        //*********************************************
        //Create all Items for display
        //*********************************************
        //Instance for settings
        GridBagConstraints gbc;

        //HeaderLabel
        JLabel headerTableLabel = new JLabel("Header entries");
        gbc = makegbc(0,1,3,1,GridBagConstraints.BOTH);
        gbc.weightx=100;
        gbc.weighty=5;
        layout.setConstraints(headerTableLabel,gbc);

        //HeaderTable
        headerTable.setSelectionMode(ListSelectionModel.SINGLE_SELECTION);
        headerTable.setRowSelectionAllowed(true);
        headerTable.setColumnSelectionAllowed(false);
        JScrollPane headerScrollPane = new JScrollPane(headerTable);
        gbc = makegbc(0,2,3,1,GridBagConstraints.BOTH);
```

```
gbc.weightx=100;
gbc.weighty=20;
layout.setConstraints(headerScrollPane,gbc);

//DataLabel
JLabel dataTableLabel = new JLabel("Event entries");
gbc = makegbc(0,3,3,1,GridBagConstraints.BOTH);
gbc.weightx=100;
gbc.weighty=5;
layout.setConstraints(dataTableLabel,gbc);

//DataTable
dataTable.setSelectionMode(ListSelectionModel.SINGLE_SELECTION);
dataTable.setRowSelectionAllowed(true);
dataTable.setColumnSelectionAllowed(false);

JScrollPane dataScrollPane = new JScrollPane(dataTable);
gbc = makegbc(0,4,3,1,GridBagConstraints.BOTH);
gbc.weightx=100;
gbc.weighty=100;
layout.setConstraints(dataScrollPane,gbc);

//SearchButton
JButton searchButton = new JButton("Search...");
searchButton.setMnemonic(KeyEvent.VK_S);
searchButton.addActionListener(new ActionListener() {
        public void actionPerformed(ActionEvent e) {
            process_search();
        }
});
gbc = makegbc(0,5,1,1,GridBagConstraints.NONE);
gbc.weightx=100;
gbc.weighty=5;
layout.setConstraints(searchButton,gbc);

//Union button
JButton unionButton = new JButton("Union Outputs...");
unionButton.setMnemonic(KeyEvent.VK_U);
unionButton.addActionListener(new ActionListener() {
        public void actionPerformed(ActionEvent e) {
            process_union();
        }
});
gbc = makegbc(1,5,1,1,GridBagConstraints.NONE);
gbc.weightx=100;
gbc.weighty=5;
layout.setConstraints(unionButton,gbc);

//show video
JButton videoButton = new JButton("Show Video...");
videoButton.setMnemonic(KeyEvent.VK_V);
videoButton.addActionListener(new ActionListener() {
        public void actionPerformed(ActionEvent e) {
```

```
                process_showVideo();
            }
        });
        gbc = makegbc(2,5,1,1,GridBagConstraints.NONE);
        gbc.weightx=100;
        gbc.weighty=5;
        layout.setConstraints(videoButton,gbc);

        //add to content
        this.add(dataTableLabel);
        this.add(headerTableLabel);
        this.add(dataScrollPane);
        this.add(headerScrollPane);
        this.add(searchButton);
        this.add(unionButton);
        this.add(videoButton);

    }

    /**
     * Puts the data from the ArrayList into the JTables depending on the type
     */
    public void fillTables() {

        //create TableModels
        headerTableModel = new DefaultTableModel();
        headerTable.setModel(headerTableModel);
        dataTableModel = new DefaultTableModel();
        dataTable.setModel(dataTableModel);

        //Define colums identifiers
        headerTableModel.setColumnIdentifiers(HeaderEntry.itemDisplayNames());
        dataTableModel.setColumnIdentifiers(EventEntry.itemDisplayNames());

        //Go throw list and put entry in table depending on type
        for (int i=0; i<DataSource.size(); i++) {

            DataEntry dataentry = (DataEntry)DataSource.get(i);

            if (dataentry instanceof HeaderEntry) {
                //if it is headerinformation, put it in headerTable
                headerTableModel.addRow(dataentry.itemsToDisplaystyle());
            }
            else if (dataentry instanceof EventEntry) {
                //if it is datainformation, put it in dataTable
                dataTableModel.addRow(dataentry.itemsToDisplaystyle());
            }
            else {
                System.out.println("TabularDisplayDialog:fillTables: unknown class in ArrayList
found");
            }
        }
    }
```

```
    /**
     * Method to process search option if search button is pressed
     */
    private void process_search() {
        SearchDialog sd = new SearchDialog(superclass, "Search",false,this.DataSource);
        sd.setVisible(true);
    }

    /**
     * Method to process union operation in case union button pressed
     */
    private void process_union() {
        UnionDialog.showUnionDialog(superclass);
    }

    /**
     * Method to process linking selected row to video
     * !!! contains dirty code !!!
     */
    private void process_showVideo() {

        ErrorDialog errDialog = new ErrorDialog(superclass,"Error showing the video",true);

        VideoSettings vs = superclass.settingspanel.getInitialVideoSettings();

        //check for set properties
        if (vs != null) {

            //check for correct settings
            if (vs.settingsOK()) {
                //get selected row
                int selRow = dataTable.getSelectedRow();

                if (selRow != -1) {

                    /*!!!! displayed evententries must have some order like event entries after
                     calling function getAllEventEntries from DataEntrie, otherwise
                     wrong entry will be shown in video !!!!! */

                    //get EventEntry depending on selected row
                    EventEntry ee =
(EventEntry)DataEntry.getAllEventEntries(DataSource).get(selRow);


                    //call functionality of abstract-graph-display to display media player
                    AbstractGraphDisplay.showMediaPlayer(vs,ee);
                }
                else {
                    errDialog.addMessage("Select entry in event table to link!",ErrorDialog.ERROR);
                    errDialog.showErrDialog();
                }
            }
```

```
            else {
                errDialog.addMessage("video settings not correct!",ErrorDialog.ERROR);
                errDialog.showErrDialog();
            }
        }
        else {
            errDialog.addMessage("Please define video source in settings step to link with
video",ErrorDialog.ERROR);
            errDialog.showErrDialog();
        }
    }

    /**
     * Adds a windowlistener which is created for this dialog to remove himself from
     * the the dialoglist in displaypanel
     */
    public void addDisplayDialogClosingAdapter() {
        this.addWindowListener(new
TabularDisplayDialogClosingAdapter(superclass,isSearchResult));
    }

    /**
     * closes the dialog
     */
    public void close () {
        //disable display
        this.setVisible(false);
        this.dispose();

        //remove from list in DisplayPanel
         DisplayPanel.removeDialogFromDialogList(this);
    }
    /**
     * creates the settings for a component which should be displayed in GridBagLayout
     * @param x startcoordinate of component
     * @param y startcoordinate of component
     * @param width how many cells
     * @param height how many cells
     * @return
     */
    private GridBagConstraints makegbc (int x, int y, int width, int height,int fill) {
        GridBagConstraints gbc = new GridBagConstraints();
        gbc.gridx = x;
        gbc.gridy = y;
        gbc.gridwidth = width;
        gbc.gridheight = height;
        gbc.fill = fill;
        //Defines the border of each element
        gbc.insets = new Insets(2, 2, 2, 2);

        return gbc;
    }
}
```

***/**

Src 33 : TabularDisplayDialogClosingAdapter.java

/**
Used by each tabular display output dialog to remove on closing from static list of dialogs in display panel
Log File Aggregation (LFA) tool V1.1.

Copyright (C) 2008 Biomedical Informatics (BMI), St George's University of London. Citations to the toolkit should be as 'de Lusignan S, Kumarapeli P, Chan T, Pflug B, van Vlymen J, Jones B, Freeman GK. The ALFA (activity Log Files Aggregation) toolkit: a method for precise observation of the consultation. <J Med Internet Res 2008; ..>.

This program is free software: you can redistribute it and/or modify it under the terms of the GNU General Public License as published by the Free Software Foundation, either version 3 of the License, or (at your option) any later version.

This program is distributed in the hope that it will be useful, but WITHOUT ANY WARRANTY; without even the implied warranty of MERCHANTABILITY or FITNESS FOR A PARTICULAR PURPOSE. See the GNU General Public License for more details.

You should have received a copy of the GNU General Public License along with this program. If not, see <http://www.gnu.org/licenses/>.

*/

***/**

Src 34: UARLogConverter.java

/**
Subclass of log converter processing UAR log files
Log File Aggregation (LFA) tool V1.1.

Copyright (C) 2008 Biomedical Informatics (BMI), St George's University of London. Citations to the toolkit should be as 'de Lusignan S, Kumarapeli P, Chan T, Pflug B, van Vlymen J, Jones B, Freeman GK. The ALFA (activity Log Files Aggregation) toolkit: a method for precise observation of the consultation. <J Med Internet Res 2008; ..>.

This program is free software: you can redistribute it and/or modify it under the terms of the GNU General Public License as published by the Free Software Foundation, either version 3 of the License, or (at your option) any later version.

This program is distributed in the hope that it will be useful, but WITHOUT ANY WARRANTY; without even the implied warranty of MERCHANTABILITY or FITNESS FOR A PARTICULAR PURPOSE. See the GNU General Public License for more details.

```
You should have received a copy of the GNU General Public License along with this program.  If
not, see <http://www.gnu.org/licenses/>.

*/

package aggregationtool;


import java.awt.event.ActionEvent;
import java.awt.event.ActionListener;

import java.text.ParsePosition;
import java.text.SimpleDateFormat;

import java.util.ArrayList;
import java.util.Date;
import java.util.NoSuchElementException;
import java.util.StringTokenizer;

import javax.swing.JComboBox;
import javax.swing.JLabel;
import javax.swing.JPanel;


public class UARLogConverter extends LogConverter {

    //Token for the file
    private static String TOKEN = " ";
    private static String ENTRYSEPARATOR = ":";

    //Format of the Time in the file
    public static String TIMEFORMAT = "kk:mm:ss";

    //ArrayList which contains the raw DataEntries after converting, but without combining
    private ArrayList rawEntries;

    //list of variable which defines the time between two entries to be combined to one duration
    //TO BE DEFINED IN SAME UNIT LIKE FILE (usually MILLISECONDS)
    private Float [] TIME_THRESHOLD = {100F, 500F, 1000F, 2000F, 3000F, 4000F, 5000F};
    private int selectedThresIndex = 2;

    //******** property panel components *********
    JComboBox thresholdCombobox = new JComboBox(TIME_THRESHOLD);

    //Value the timevalues of the file must be divided by
    //because dateDiff function returnes milliseconds
    private static int divValue = 1000;

    //************************************************************************
    //************************* constructor ***********************************
    //************************************************************************
```

```
    public UARLogConverter () {

    }

    public UARLogConverter(String filePath) {
        this.FilePath = filePath;
    }
    //***********************************************************************
    //********************** property panel method ****************************
    //***********************************************************************

      /**
       * Method to create properties for specific options on format
       * Should instance property Panel of super class, add all necessary components
       * and read them before processing to get required options
       * In case no property panel is needed leave body without content
       */
      public void createPropertyPanel() {

          //TODO In case options are necessary uncomment following lines, add components
          //and read them before processing

            //*** Panel ***
            propertyPanel = new JPanel();

            propertyPanel.setLayout( null );

            //Threshold label
            JLabel thresholdLabel = new JLabel("Choose threshold between two values to get
combined");
            thresholdLabel.setBounds(20,30,350,20);

            //Threshold combo box
            thresholdCombobox.setBounds(20,60,100,20);
            thresholdCombobox.setSelectedIndex(selectedThresIndex);
            thresholdCombobox.addActionListener(new ActionListener() {

                public void actionPerformed(ActionEvent e) {
                    //set slectedThresIndex as new selected one in combo box
                    selectedThresIndex = thresholdCombobox.getSelectedIndex();
                }
             });

            //add to panel
            propertyPanel.add(thresholdLabel);
            propertyPanel.add(thresholdCombobox);
      }
    //***********************************************************************
    //******************* content process methods ****************************
    //***********************************************************************
    /**
     * Method to interpret read file to create Data entries
     */
```

```
    public void interpretLogFile() {

        //Arraylist containing the common content of the file
        CommonContent = new ArrayList();

        rawEntries = new ArrayList();

         /********************
          ** process Header **
          ********************/

        //Headerinformation
        CommonContent.add(new HeaderEntry(1.0F,new Date(this.LastModified),this.FileName));

        //First time in logfile is taken as zerotime
        Date ZeroTime = null;

        //Defines the format for the time
        SimpleDateFormat sdf = new SimpleDateFormat(TIMEFORMAT);

        //Parse the whole first line to Zerotime
        ZeroTime = sdf.parse((String)FileContent.get(0),new ParsePosition(0));

        if (ZeroTime == null) {
            addErrorWarning(ErrorDialog.ERROR,"Unable to convert first entry to time!");
            return;
        }

        //after processing zerotime, delete from file content
        FileContent.remove(0);

        //************************

         /********************
          ** process Content **
          ********************/
        StringTokenizer st;

        //for each row get date and event
        for (int i = 0; i < FileContent.size(); i++) {
            //because the separtor between time and value and the separtor between time are
simular,
            //the file must be splitted by space
            st = new StringTokenizer((String)FileContent.get(i),TOKEN);

            /*now the Tokenizer must create a format like this
             * "HH:MM:SS"
             * "" (empty token)
             * ":" (separator between time and value)
             * "" (empty token)
             * "<value>"
             */
```

```
        try {
            //*********** Time ***********
            String TimeString = st.nextToken();
            Date EntryTime = sdf.parse(TimeString,new ParsePosition(0));

            //check whether Entrytime is not null
            if (EntryTime == null) {
                throw new NumberFormatException("Unable to create time for line "+(i+1));
            }

            long difference = this.getDiffBetweenDates(EntryTime,ZeroTime);

            //Check if difference below zero
            if (difference <0) {
                addErrorWarning(ErrorDialog.WARNING,"Info: line "+(i+1)+" contains a time
before starttime!");
            }

            //*********** Separator ***********
            //as long as next token is empty or separator, go to next token without any action
            String value="";
            while (value.equals("") || value.equals(ENTRYSEPARATOR)) {
                value = st.nextToken();
            }

            //After finding first part of a value, add the rest of the line to the value
            while (st.hasMoreTokens()) {
                value += st.nextToken();
            }

            //*********** add to raw entry list ************
            rawEntries.add(new EventEntry(difference,-1,LogConverter.UAREVENTTYPE,value));
        }
        catch (NumberFormatException ex) {
            addErrorWarning(ErrorDialog.WARNING,ex.getMessage()+", entry ignored");
        }
        catch (NoSuchElementException ex2) {
            addErrorWarning(ErrorDialog.WARNING,"Error in line "+(i+1)+", unable to split time
from value, entry ignored");
        }
    }
    //***********************

     //After creating rawContent entries will be combined
     createCommonContentFromRawEntries();
  }

  /**
   * this function compares the times of two dates and return the difference in seconds
   */
  private long getDiffBetweenDates (Date d1, Date d2) {
     return (d1.getTime()-d2.getTime());
  }
```

```
    /**
     * After interpretation all EventEntries are saved in RawEntries
     * this function combines all entries which starttime is less then the defined
     * value to one duration entry
     * The result is saved in commonContent
     */
    private void createCommonContentFromRawEntries() {

        while (!rawEntries.isEmpty()) {
            EventEntry entry = (EventEntry) rawEntries.get(0);
            float highestDifference = searchHighestDiff(entry);

            //After searching through list and removing all entries in intervall (including entry it's
own),
            //add entry with duration or as event, if no other entries were found
            if (highestDifference == -1) {
                CommonContent.add(new
EventEntry(entry.StartTime/divValue,highestDifference,LogConverter.UAREVENTTYPE,getGroupn
ameForValue(entry.getEvent().getValue())));
            }
            else {
                CommonContent.add(new
EventEntry(entry.StartTime/divValue,(entry.StartTime+highestDifference)/divValue,LogConverter.
UAREVENTTYPE,getGroupnameForValue(entry.getEvent().getValue())));
            }

            //after searching an entry, remove from list
            rawEntries.remove(entry);
        }
    }

    /**
     * This function is called recursiv to search through list and combine all
     * entries in specified intervall
     * recursion is needed because in case finding an entry in interval also it's
     * entries in interval must be searched and combined
     * @param entry to search for
     * @return highest difference given value and all other values in list (in case of queue of some
values last value given)
     */
    private float searchHighestDiff(EventEntry entry) {

        //defines the highest difference between given object and all other objects in list in interval
        float highestDifference = -1;

        //Defines the difference between to given object and compared object in list
        float currDiff;

        //go through list from back to front and search for entries in intervall
        for (int i =rawEntries.size()-1; i>=0; i--) {
            EventEntry toCompare = (EventEntry)rawEntries.get(i);
```

```
            //only compare times if entries have same value
            if
(getGroupnameForValue(entry.getEvent().getValue()).equals(getGroupnameForValue(toCompare.
getEvent().getValue()))) {

                //check whether compared entries starttime is between starttime and
starttime+Threshold
                if (toCompare.StartTime > entry.StartTime && toCompare.StartTime <=
(entry.StartTime+TIME_THRESHOLD[selectedThresIndex])) {

                    //create diffence of given value and compare to
                    currDiff = toCompare.StartTime - entry.StartTime;

                    //search for the highestValue of current differene, already compared values
(highestDifference) and recursiv search
                    //recursive call searches for other values in intervall of compare to
                    highestDifference =
Math.max(highestDifference,Math.max(currDiff,searchHighestDiff(toCompare)+currDiff));

                    //after comparing with an entry in intervall, this entry must be deleted from list
                    rawEntries.remove(i);
                }
            }
        }

        return highestDifference;
    }

    /**
     * Used by createCommonContentFromRawEntries
     * @param value of the event entry
     * @return int to which the value fits
     */
    private String getGroupnameForValue(String value) {

        if (value.equals("(DownA)") || value.equals("(UpA)") || value.equals("(LeftA)") ||
value.equals("(RightA)")) {
            return "Navigation";
        }
        //compare without space because after tokenize all info is put together without spaces
between
        else if (value.equals("(MouseClick)")) {
            return "Mouse";
        }
        else {
            return "Key";
        }
    }
}
```

***/**

Src 35 : UnionDialog.java

```
/**
Used by tabular search dialogs to combine several output results to one dialog
Log File Aggregation (LFA) tool V1.1.

Copyright (C) 2008 Biomedical Informatics (BMI), St George's University of London. Citations to
the toolkit should be as 'de Lusignan S, Kumarapeli P, Chan T, Pflug B, van Vlymen J, Jones B,
Freeman GK. The ALFA (activity Log Files Aggregation) toolkit: a method for precise observation
of the consultation. <J Med Internet Res 2008; ..>.

This program is free software: you can redistribute it and/or modify it under the terms of the
GNU General Public License as published by the Free Software Foundation, either version 3 of the
License, or (at your option) any later version.

This program is distributed in the hope that it will be useful, but WITHOUT ANY WARRANTY;
without even the implied warranty of MERCHANTABILITY or FITNESS FOR A PARTICULAR
PURPOSE.  See the GNU General Public License for more details.

You should have received a copy of the GNU General Public License along with this program.  If
not, see <http://www.gnu.org/licenses/>.

*/

package aggregationtool;

import java.awt.Dimension;
import java.awt.Frame;

import java.awt.Rectangle;

import java.awt.event.ActionEvent;
import java.awt.event.ActionListener;

import java.awt.event.KeyEvent;

import java.util.ArrayList;

import java.util.Collections;
import java.util.Iterator;

import javax.swing.JButton;
import javax.swing.JCheckBox;
import javax.swing.JDialog;
import javax.swing.JLabel;

public class UnionDialog extends JDialog {

    //singleton instance
    private static UnionDialog instance;

    GUISuperclass superclass;
```

```
//Arraylist with all open Dialogs
ArrayList openDialogs;

//Labels for dialog_names
JLabel [] dialogNames;

//checkboxes for dialogs
JCheckBox [] dialogCheck;

//counter for unionDialog
private static int unionCounter;

private JLabel header_label = new JLabel();

/**
 * This class is only allowed to be showed once, therefore it is singleton
 * That means that all constructors are privat, the only way to work with this class is
 * to call this method
 * @param parent
 */
public static void showUnionDialog(GUISuperclass parent) {

    instance = new UnionDialog(parent,"Union search results",true);
    instance.setVisible(true);

}

private UnionDialog(GUISuperclass parent, String title, boolean modal) {
    super(parent, title, modal);

    this.superclass = superclass;

    //get all open dialogs with tabuluar data source
    this.openDialogs = DisplayPanel.getOpenDialogsList();

    try {
        jbInit();
    } catch (Exception e) {
        e.printStackTrace();
    }
}

private void jbInit() throws Exception {
    this.getContentPane().setLayout( null );

    //headerlabel
    header_label.setText("Select all open dialogs you want to combine...");
    header_label.setBounds(new Rectangle(20, 15, 335, 25));
    this.getContentPane().add(header_label, null);

    //for each open dialog create entry with its name and a checkbox
    dialogNames = new JLabel[openDialogs.size()];
```

```
        dialogCheck = new JCheckBox[openDialogs.size()];

        for (int i=0; i < openDialogs.size(); i++) {
            TabularDisplayDialog dialog = (TabularDisplayDialog)openDialogs.get(i);

            //label
            dialogNames[i] = new JLabel(dialog.getTitle());
            dialogNames[i].setBounds(20,60+30*i,300,25);
            this.getContentPane().add(dialogNames[i]);

            //checkbox
            dialogCheck[i] = new JCheckBox("");
            dialogCheck[i].setBounds(330,60+30*i,30,25);
            this.getContentPane().add(dialogCheck[i]);
        }

        //Combine button
        JButton combineButton = new JButton("Combine");
        combineButton.setBounds(20,80+30*openDialogs.size(),105,20);
        combineButton.setMnemonic(KeyEvent.VK_C);
        combineButton.addActionListener(new ActionListener() {
         public void actionPerformed(ActionEvent e) {
             process_combine();
         }
        }
        );
        this.getContentPane().add(combineButton);

        this.setSize(new Dimension(370,140+30*openDialogs.size()));
    }

    public void process_combine() {

        ArrayList combinedData = new ArrayList();

        //go throw all checkboxes, and combine selected
        for (int i = 0; i < openDialogs.size(); i++) {

            if (dialogCheck[i].isSelected()) {
                combinedData.addAll(((TabularDisplayDialog)openDialogs.get(i)).DataSource);

                ((TabularDisplayDialog)openDialogs.get(i)).close();
                i--;
            }
        }
        //Sort entries
        Collections.sort(combinedData);

        //Display new dialog mit combined data
        unionCounter++;

        TabularDisplayDialog combinDialog = new TabularDisplayDialog(superclass,"combined
output "+unionCounter,false,combinedData,true);
```

```
        DisplayPanel.addDialogToDialogList(combinDialog);
        combinDialog.setVisible(true);

        //set this Invisbile
        this.setVisible(false);
    }
}
```

***/**

Src 36 : VideoSettings.java

```
/**
used by the aggregation media player to set and get all required video settings for the video
source
Log File Aggregation (LFA) tool V1.1.

Copyright (C) 2008 Biomedical Informatics (BMI), St George's University of London. Citations to
the toolkit should be as 'de Lusignan S, Kumarapeli P, Chan T, Pflug B, van Vlymen J, Jones B,
Freeman GK. The ALFA (activity Log Files Aggregation) toolkit: a method for precise observation
of the consultation. <J Med Internet Res 2008; ..>.

This program is free software: you can redistribute it and/or modify it under the terms of the
GNU General Public License as published by the Free Software Foundation, either version 3 of the
License, or (at your option) any later version.

This program is distributed in the hope that it will be useful, but WITHOUT ANY WARRANTY;
without even the implied warranty of MERCHANTABILITY or FITNESS FOR A PARTICULAR
PURPOSE.  See the GNU General Public License for more details.

You should have received a copy of the GNU General Public License along with this program.  If
not, see <http://www.gnu.org/licenses/>.

*/

package aggregationtool;

import java.io.File;

public class VideoSettings {

    //defines whether settings are allowed to use
    private boolean settingsOK;

    //**** player information ***
    //source path of the videofile
    private String filePath;

    //starttime for the player
    private double startTime;
    private double endTime;
```

```
//values to be set for start- and endtime if no value should be set
public static double NO_CERTAIN_STARTTIME = -2;
public static double NO_CERTAIN_ENDTIME = -1;

//rate the player is playes the video, as set 1.0 is default
private float videoRate = 1.0F;

public int videoWidth = 0;
public int videoHeight = 0;
public int controlHeight = 30;
public int insetWidth = 10;
public int insetHeight = 30;
public boolean firstTime = true;

private VideoSettings () {

}

/**
 * This method should always be checked before using any settings
 * @return true in case all settings are ok, false otherwise
 */
public boolean settingsOK() {
    return settingsOK;
}

/**
 * Method to set OK flag for settings from outside
 * @param settingsOK
 */
public void setSettingsOK(boolean settingsOK) {
    this.settingsOK = settingsOK;
}

/**
 * @return filePath of data source
 */
public String getFilePath() {
    return filePath;
}

/**
 * gives information if certain starttime to start the video is set
 * @return true if specific starttime is set, false otherwise
 */
public boolean startTimeSet() {
    return !(startTime == NO_CERTAIN_STARTTIME);
}

/**
 * gives information if certain endtime to end the video is set
 * @return true if specific endtime is set, false otherwise
 */
```

```
    public boolean endTimeSet() {
        return !(endTime == NO_CERTAIN_ENDTIME);
    }

    /**
     * @return starttime for video if set, NO_CERTAIN_STARTTIME otherwise
     */
    public double getStartTime() {
        return startTime;
    }

    /**
     * @return endtime for video if set, NO_CERTAIN_ENDTIME otherwise
     */
    public double getEndTime() {
        return endTime;
    }

    /**
     * Sets start- and endtime for video in case video shouldn't be played the whole length
     * to set to wohle length define start- and end time as NO_CERTAIN_STARTTIME and
NO_CERTAIN_ENDTIME
     * @param startTime the video should start from
     * @param endTime the video should end
     */
    public void setStartEndTimes(double startTime,double endTime) {
        //check if starttime smaller then endtime
        if ((startTime < endTime) || (startTime != NO_CERTAIN_STARTTIME && endTime ==
NO_CERTAIN_ENDTIME)) {
            //set start- and end time
            this.startTime = startTime;
            this.endTime = endTime;
        }
        else {
            this.settingsOK=false;
        }
    }

    /**
     * Sets video rate the video should be played with
     * @param rate
     */
    public void setVideoRate(float rate) {
        this.videoRate = rate;
    }

    /**
     * @return video rate
     */
    public float getVideoRate() {
        return videoRate;
    }
```

```
    @Deprecated
    public void printSettings() {
        System.out.println("------- video settings -----");
        System.out.println("FilePath: "+filePath);
        System.out.println("starttime: "+startTime);
        System.out.println("endtime: "+startTime);
    }

    /**
     * Same functionality like method with all parameters, except setting not given
     * params to default values
     * @param filePath of source file
     * @return instance of videoSettings with set parameters
     */
    public static VideoSettings createSettings(String filePath) {

        return createSettings(filePath,NO_CERTAIN_STARTTIME,NO_CERTAIN_ENDTIME,1.0F);
    }

    /**
     * Same functionality like method with all parameters, except setting not given
     * params to default values
     * @param filePath of source file
     * @param videoRate rate the video should be played with
     * @return instance of videoSettings with set parameters
     */
    public static VideoSettings createSettings(String filePath, float videoRate) {

        return
createSettings(filePath,NO_CERTAIN_STARTTIME,NO_CERTAIN_ENDTIME,videoRate);
    }

    /**
     * Same functionality like method with all parameters, execpt defining
     * videorate as default to 1.0
     * @param filePath of the source file
     * @param startTime the video should start from
     * @param endTime the video should end
     * @return intance of videoSettings with set parameters
     */
    public static VideoSettings createSettings(String filePath,double startTime, double endTime) {

        return createSettings(filePath,startTime,endTime,1.0F);
    }

    /**
     * static method to create an instance of videosettings
     * In case at least one setting is not ok variable settingsOK is set to false
     * @param filePath of the source file
     * @param startTime the video should start from
     * @param endTime the video should end
     * @param videoRate defines rate video should be played (only values between 0.1 and 2)
     * @return intance of videoSettings with set parameters
```

```
     */
    public static VideoSettings createSettings(String filePath,double startTime, double endTime,
float videoRate) {

        VideoSettings curSettings = new VideoSettings();

        //check if sourcefile is a file
        File sourceFile = new File(filePath);
        curSettings.settingsOK = sourceFile.exists();

        if (curSettings.settingsOK) {
            curSettings.filePath = filePath;

            //set start- and end time
            curSettings.setStartEndTimes(startTime,endTime);

            if (curSettings.settingsOK) {
                if (videoRate >=0.1F && videoRate<=2.0F) {
                    curSettings.setVideoRate(videoRate);
                }
                else {
                    curSettings.setSettingsOK(false);
                }
            }
        }

        return curSettings;
    }
}
```

Src 37 : WindowClosingAdapter.java

```
/**
used by main frame to close application on pushing the window close button
Log File Aggregation (LFA) tool V1.1.

Copyright (C) 2008 Biomedical Informatics (BMI), St George's University of London. Citations to
the toolkit should be as 'de Lusignan S, Kumarapeli P, Chan T, Pflug B, van Vlymen J, Jones B,
Freeman GK. The ALFA (activity Log Files Aggregation) toolkit: a method for precise observation
of the consultation. <J Med Internet Res 2008; ..>.

This program is free software: you can redistribute it and/or modify it under the terms of the
GNU General Public License as published by the Free Software Foundation, either version 3 of the
License, or (at your option) any later version.

This program is distributed in the hope that it will be useful, but WITHOUT ANY WARRANTY;
without even the implied warranty of MERCHANTABILITY or FITNESS FOR A PARTICULAR
PURPOSE.  See the GNU General Public License for more details.

You should have received a copy of the GNU General Public License along with this program.  If
not, see <http://www.gnu.org/licenses/>.
```

```
*/

package aggregationtool;

import java.awt.event.*;


public class WindowClosingAdapter
extends WindowAdapter{

   public WindowClosingAdapter() {
   }

   public void windowClosing(WindowEvent event)
   {
      event.getWindow().setVisible(false);
      event.getWindow().dispose();

      System.exit(0);
   }
}
```

Src 38 : XMLConvertable.java

```
/**
This interface defines method to create DOM elements of a class
Log File Aggregation (LFA) tool V1.1.

Copyright (C) 2008 Biomedical Informatics (BMI), St George's University of London. Citations to
the toolkit should be as 'de Lusignan S, Kumarapeli P, Chan T, Pflug B, van Vlymen J, Jones B,
Freeman GK. The ALFA (activity Log Files Aggregation) toolkit: a method for precise observation
of the consultation. <J Med Internet Res 2008; ..>.

This program is free software: you can redistribute it and/or modify it under the terms of the
GNU General Public License as published by the Free Software Foundation, either version 3 of the
License, or (at your option) any later version.

This program is distributed in the hope that it will be useful, but WITHOUT ANY WARRANTY;
without even the implied warranty of MERCHANTABILITY or FITNESS FOR A PARTICULAR
PURPOSE.  See the GNU General Public License for more details.

You should have received a copy of the GNU General Public License along with this program.  If
not, see <http://www.gnu.org/licenses/>.

*/

package aggregationtool;

import org.w3c.dom.Document;
import org.w3c.dom.Element;
```

```
public interface XMLConvertable {

    /**
     * create element for it self and add elements for each content of class
     * @param document
     */
    public Element createXMLElement(Document document);

    /**
     * Method to set properties of class by an given element of XML structure
     *
     * !!!!
     * Attention!
     * To get the text values of the variables always the first text element
     * is accessed at the implementation of the function
     * so if there would be no text or more than one text entry the program would
     * not work correctly as well as if there would be more entries with the same
     * identifier, in this case always the first one is choosen
     * !!!
     * @param classElement xml element with content for this class
     * @return
     */
    public void setPropertiesByXML(Element classElement);
}
```

Src 39: XMLFactory.java

```
/**
This class contains the whole XML functionality of the aggregation tool
Log File Aggregation (LFA) tool V1.1.

Copyright (C) 2008 Biomedical Informatics (BMI), St George's University of London. Citations to
the toolkit should be as 'de Lusignan S, Kumarapeli P, Chan T, Pflug B, van Vlymen J, Jones B,
Freeman GK. The ALFA (activity Log Files Aggregation) toolkit: a method for precise observation
of the consultation. <J Med Internet Res 2008; ..>.

This program is free software: you can redistribute it and/or modify it under the terms of the
GNU General Public License as published by the Free Software Foundation, either version 3 of the
License, or (at your option) any later version.

This program is distributed in the hope that it will be useful, but WITHOUT ANY WARRANTY;
without even the implied warranty of MERCHANTABILITY or FITNESS FOR A PARTICULAR
PURPOSE.  See the GNU General Public License for more details.

You should have received a copy of the GNU General Public License along with this program.  If
not, see <http://www.gnu.org/licenses/>.

*/

package aggregationtool;
```

```
//JAXP APIs
import javax.xml.parsers.DocumentBuilder;
import javax.xml.parsers.DocumentBuilderFactory;
import javax.xml.parsers.ParserConfigurationException;

//exceptions
import org.xml.sax.SAXException;

//file packages
import java.io.File;
import java.io.IOException;

//DOM libs
import org.w3c.dom.*;

//not XML packages
import java.util.ArrayList;

//for XML output
import java.util.Collections;

import java.util.zip.DataFormatException;

import javax.xml.transform.Transformer;
 import javax.xml.transform.TransformerFactory;
 import javax.xml.transform.TransformerException;
 import javax.xml.transform.TransformerConfigurationException;

 import javax.xml.transform.dom.DOMSource;

 import javax.xml.transform.stream.StreamResult;


public class XMLFactory {

    //static instance for singlton access
    private static XMLFactory instance;

    //static instance for BuilderFactory to create document builder
    private static DocumentBuilderFactory factory = DocumentBuilderFactory.newInstance();

    private XMLFactory() {
    }

    //*************************************************************************
    //******************* methods for converting ******************************
    //*************************************************************************

    /**
     * This method supports the conversion of an XML file
     * to an ArrayList of DataEntries
     * @param filePath path of file
```

```
     * @throws DataFormatException if any catched error occurs
     * @return sorted Arraylist of Data Entries
     */
    public ArrayList convertXMLToDataEntries(String filePath)
    throws DataFormatException{

        //arraylist to save data entries in
        ArrayList dataEntries = new ArrayList();

        try {

            //create document of document builder to create XML elements
            DocumentBuilder builder = factory.newDocumentBuilder();

            //parse to whole XML content to DOM hirarchie
            Document document = builder.parse( new File (filePath) );

            //******* create header entries *******
            //get all header entries in XML file
            NodeList headerNodes =
document.getDocumentElement().getElementsByTagName(HeaderEntry.class.getSimpleName());

            //go through them and instance for each a new header entry
            for (int i=0; i< headerNodes.getLength(); i++) {
                HeaderEntry headerE = new HeaderEntry();
                headerE.setPropertiesByXML((Element)headerNodes.item(i));

                dataEntries.add(headerE);
            }

            //******** create event entries ********
            //do the same for event entries like the header entries
            NodeList eventNodes =
document.getDocumentElement().getElementsByTagName(EventEntry.class.getSimpleName());

            for (int i=0; i < eventNodes.getLength(); i++) {
                EventEntry eventE = new EventEntry();
                eventE.setPropertiesByXML((Element)eventNodes.item(i));

                dataEntries.add(eventE);
            }

            //sort (should normally not be necessary as created output from the program is sorted
already)
            Collections.sort(dataEntries);

            return dataEntries;

        } catch (SAXException sxe) {
           // Error generated during parsing
           Exception  x = sxe;
           if (sxe.getException() != null)
               x = sxe.getException();
```

```
            throw new DataFormatException(sxe.getMessage());

        } catch (ParserConfigurationException pce) {
            // Parser with specified options can't be built
             throw new DataFormatException(pce.toString());

        } catch (IOException ioe) {
            // I/O error
             throw new DataFormatException(ioe.toString());
        }
    }

    /**
     * Method to create a XML file for given ArrayList of Data Entries
     *
     * it contains following information
     * -root entry
     * - - Headerinformation
     * - - DataEntries
     * - - - HeaderEntries
     * - - - EventEntries
     *
     * @throws DataFormatException if any error occurs
     */
    public void convertDataEntriesToXMLFile(ArrayList dataSource, String filePath)
    throws DataFormatException{

        try {

            //parse to whole XML content to DOM hirarchie
            DocumentBuilder builder = factory.newDocumentBuilder();

            //create a new document to store data in
            Document document = builder.newDocument();

            document.setXmlStandalone(true);

            //******************** root node ***********************************
            //root node is necessary, because hirarchie only allows one root node maximum
            Element rootNode = document.createElement("AggregationTool");

            //******************** process header ******************************
            //global timeunit
            Element headerInfo = document.createElement("Headerinformation");

            Element globalTimeUnit = document.createElement("GLOBAL_TIMEUNIT");

globalTimeUnit.appendChild(document.createTextNode(""+LogConverter.GLOBAL_TIMEUNIT));
            headerInfo.appendChild(globalTimeUnit);

            rootNode.appendChild(headerInfo);
```

Biomedical Informatics, Division of Community Health Sciences
Tel + 44 (0)20 8725 5661 Fax + 44 (0)20 8725 3584 Email slusigna@sgul.ac.uk

```
        //******************** process content ******************************
        Element dataEntries = document.createElement("DataEntries");

        //*** Header Entries ***
        Element elemHeaderEntries = document.createElement("HeaderEntries");
        //get all headerentries of data source
        ArrayList headerEntries = DataEntry.getAllHeaderEntries(dataSource);

        //for each call functionality to create XML element
        for (int i=0; i<headerEntries.size(); i++) {

elemHeaderEntries.appendChild(((XMLConvertable)headerEntries.get(i)).createXMLElement(docu
ment));
        }

        dataEntries.appendChild(elemHeaderEntries);

        //*** Event Entries ***
        //get all eventtypes of source and there content
        Object [] eventTypes = DataEntry.getUniqueEventTypes(dataSource);
        ArrayList [] eventValues = DataEntry.splitOnEventType(dataSource);

        //for each event type create element...
        for (int i=0; i< eventTypes.length; i++) {

            Element eventType = document.createElement(""+eventTypes[i]);
            ArrayList values = eventValues[i];

            //... and add all it's entry to it
            for (int j=0; j<values.size(); j++) {

eventType.appendChild(((XMLConvertable)values.get(j)).createXMLElement(document));
            }

            dataEntries.appendChild(eventType);
        }

        rootNode.appendChild(dataEntries);


        //add root node to document
        document.appendChild(rootNode);

        //****** Write File ******
        //to write a DOM Model a transformer is needed which is defined below
        try {
            TransformerFactory tFactory =
                TransformerFactory.newInstance();
              Transformer transformer = tFactory.newTransformer();

              DOMSource source = new DOMSource(document);
              StreamResult result = new StreamResult(new File(filePath));
              transformer.transform(source, result);
```

```
            }catch (TransformerConfigurationException tce) {
               // Error generated by the parser
                throw new DataFormatException(tce.toString());

            } catch (TransformerException te) {
               // Error generated by the parser
                throw new DataFormatException(te.toString());
            }

        } catch (ParserConfigurationException pce) {
           // Parser with specified options can't be built
            throw new DataFormatException(pce.toString());
        }
    }

    //*****************************************************************************
    //******************* methods for converting *******************************
    //*****************************************************************************

    public static XMLFactory getInstance() {

        if (instance == null) {
            instance = new XMLFactory();
        }

        return instance;
    }
}
```

Src 40 : XMLLogConverter.java

/**
Subclass of log converter processing XML log files
Log File Aggregation (LFA) tool V1.1.

Copyright (C) 2008 Biomedical Informatics (BMI), St George's University of London. Citations to the toolkit should be as 'de Lusignan S, Kumarapeli P, Chan T, Pflug B, van Vlymen J, Jones B, Freeman GK. The ALFA (activity Log Files Aggregation) toolkit: a method for precise observation of the consultation. <J Med Internet Res 2008; ..>.

This program is free software: you can redistribute it and/or modify it under the terms of the GNU General Public License as published by the Free Software Foundation, either version 3 of the License, or (at your option) any later version.

This program is distributed in the hope that it will be useful, but WITHOUT ANY WARRANTY; without even the implied warranty of MERCHANTABILITY or FITNESS FOR A PARTICULAR PURPOSE. See the GNU General Public License for more details.

```
You should have received a copy of the GNU General Public License along with this program.  If
not, see <http://www.gnu.org/licenses/>.

*/

package aggregationtool;

import java.util.ArrayList;
import java.util.zip.DataFormatException;

import javax.swing.JPanel;

public class XMLLogConverter extends LogConverter {


    //*************************************************************************
    //************************** constructor ***********************************
    //*************************************************************************

    public XMLLogConverter() {

    }

    public XMLLogConverter(String filePath) {
        this.FilePath = filePath;
    }
    //*************************************************************************
    //********************** property panel method ****************************
    //*************************************************************************

     /**
      * Method to create properties for specific options on format
      * Should instance property Panel of super class, add all necessary components
      * and read them before processing to get required options
      * In case no property panel is needed leave body without content
      */
     public void createPropertyPanel() {

         //TODO In case options are necessary uncomment following lines, add components
         //and read them before processing

          /*
          //*** Panel ***
          propertyPanel = new JPanel();

          propertyPanel.setLayout( null );

          //TODO add properties
          */
     }
    //*************************************************************************
    //******************* content process methods ****************************
    //*************************************************************************
```

```
    /**
     * This method is overwritten as the XML factory reads the file as it's self
     * @return ArrayList with common Data
     */
    public ArrayList getDataFromFile() {

        //as done ususally in read file define errorWarning list
        errorsWarnings = new ArrayList();

        this.interpretLogFile();

        if (!errDialogEntry.containsErrors(errorsWarnings)) {
            //in case no standard timeunit is required only remove this method here
            this.standardiseTimeUnit();
        }

        return this.CommonContent;
    }

    /**
     * In this subclass this method only uses the functionality of XML factory
     */
    public void interpretLogFile() {

        //in case any error occurs add the error to error warnings list
        try {
            //now only thing to do is call functionality of XML factory
            CommonContent = XMLFactory.getInstance().convertXMLToDataEntries(FilePath);

            if (CommonContent.isEmpty()) {
                addErrorWarning(ErrorDialog.ERROR,"No header- and event-ntries found in XML
File");
            }
        }
        catch (DataFormatException ex) {
            addErrorWarning(ErrorDialog.ERROR,ex.getMessage());
        }
    }
}
```

Src 41: XMLOutputConverter.java

```
/**
This subclass of Outputconverter allows to handle the XML output
Log File Aggregation (LFA) tool V1.1.

Copyright (C) 2008 Biomedical Informatics (BMI), St George’s University of London. Citations to
the toolkit should be as ‘de Lusignan S, Kumarapeli P, Chan T, Pflug B, van Vlymen J, Jones B,
Freeman GK. The ALFA (activity Log Files Aggregation) toolkit: a method for precise observation
of the consultation. <J Med Internet Res 2008; ..>.
```

This program is free software: you can redistribute it and/or modify it under the terms of the GNU General Public License as published by the Free Software Foundation, either version 3 of the License, or (at your option) any later version.

This program is distributed in the hope that it will be useful, but WITHOUT ANY WARRANTY; without even the implied warranty of MERCHANTABILITY or FITNESS FOR A PARTICULAR PURPOSE. See the GNU General Public License for more details.

You should have received a copy of the GNU General Public License along with this program. If not, see <http://www.gnu.org/licenses/>.

```
*/

package aggregationtool;

import java.io.IOException;
import java.util.zip.DataFormatException;

public class XMLOutputConverter extends OutputConverter {


    //*** Property Panel components ***


    //**************************************************************************
    //***************** constructor/building methods ***************************
    //**************************************************************************
    public XMLOutputConverter() {

        super();

        createPropertyPanel();
    }

    /**
     * creates a JPanel with all required properties for creating an SDS file
     */
    protected void createPropertyPanel() {

        //Uncomment code below in case to add settings to output
        /*
        //*** Panel ***
        propertyPanel = new JPanel();

        propertyPanel.setLayout( null );

        //TODO add properties
        */
    }

    //**************************************************************************
    //************************* operating methods ******************************
    //**************************************************************************
```

```
    /**
     * Overloaded method from OutputConverter because file is already written
     * by XML Factory
     * @throws DataFormatException
     * @throws IOException
     */
    public void createOutputFile() throws DataFormatException,IOException {

        //only call this method
        convertSource();
    }

    /**
     * Method which access to XML factory to produce xml file from data entries
     * @throws DataFormatException
     */
    public void convertSource() throws DataFormatException {

        XMLFactory.getInstance().convertDataEntriesToXMLFile(dataSource,filePath);
    }
}
```
