# Supplementary material for: The ALFA (Activity Log Files Aggregation) Toolkit: A Method for Precise Observation of the Consultation
Source: J Med Internet Res. 2008 Sep 8;10(4):e27. doi: 10.2196/jmir.1080 (PMC2629369; doi:10.2196/jmir.1080)
Supplement: Supplementary file 6 [file jmir_v10i4e27_app6.pdf]

User Activity Recording (UAR) Tool

General Copyright statement

User Activity Recording (UAR) Tool V1.0

Copyright (C) 2008 Biomedical Informatics (BMI), St George's University of London. Citations to the toolkit should be as ‘de Lusignan S, Kumarapeli P, Chan T, Pflug B, van Vlymen J, Jones B, Freeman GK. The ALFA (activity Log Files Aggregation) toolkit: a method for precise observation of the consultation. *<J Med Internet Res 2008; ..>.*

This program is free software: you can redistribute it and/or modify it under the terms of the GNU General Public License as published by the Free Software Foundation, either version 3 of the License, or (at your option) any later version.

This program is distributed in the hope that it will be useful, but WITHOUT ANY WARRANTY; without even the implied warranty of MERCHANTABILITY or FITNESS FOR A PARTICULAR PURPOSE. See the GNU General Public License for more details.

You should have received a copy of the GNU General Public License along with this program. If not, see <http://www.gnu.org/licenses/>.

Any redistributions/public demonstration/publication/use of this software should acknowledge the original developers mentioned above as the Copyright holders. It is also encouraged to inform the original developers about such activities in order to assure the consistency among future software versions and to keep potential users up-to-date.

Source codes

Language: Microsoft Visual Basic 6.0
Platform: Microsoft Windows

Src1: frmMouse.frm

```
‘  VB form for the recorder interface and functionality
‘  User Action Recodering (UAR) tool V1.0

‘  Copyright (C) 2008 Biomedical Informatics (BMI), St George's University of London. Citations to
‘  the toolkit should be as ‘de Lusignan S, Kumarapeli P, Chan T, Pflug B, van Vlymen J, Jones B,
‘  Freeman GK. The ALFA (activity Log Files Aggregation) toolkit: a method for precise
‘ observation of the consultation. <J Med Internet Res 2008; ..>.

‘  This program is free software: you can redistribute it and/or modify it under the terms of the
‘  GNU General Public License as published by the Free Software Foundation, either version 3 of
‘  the License, or (at your option) any later version.

‘  This program is distributed in the hope that it will be useful, but WITHOUT ANY WARRANTY;
‘  without even the implied warranty of MERCHANTABILITY or FITNESS FOR A PARTICULAR
```

Biomedical Informatics, Division of Community Health Sciences
Tel + 44 (0)20 8725 5661 Fax + 44 (0)20 8725 3584 Email slusigna@sgul.ac.uk

```
`  PURPOSE.  See the GNU General Public License for more details.

`  You should have received a copy of the GNU General Public License along with this program.  If
`  not, see <http://www.gnu.org/licenses/>.

Option Explicit

'declare and initiate required objects
Dim fs As FileSystemObject
Dim ts As TextStream
Dim st, et As Date

Private Declare Function GetCursorPos Lib "user32" (lpPoint As POINTAPI) As Long
Private Declare Function SetCursorPos Lib "user32" (ByVal X As Long, ByVal Y As Long) As Long
Private Declare Sub Sleep Lib "kernel32" (ByVal dwMilliseconds As Long)

Private Type POINTAPI
        X As Long
        Y As Long
End Type
Private i As Long
Private sMouseArray() As String

Private Sub cmdNew_Click()
cmdStop.Enabled = False
tmrMouse.Enabled = False
cmdRecord.Enabled = True
cmdPlay.Enabled = False

i = 0
End Sub

Private Sub cmdPlay_Click()
Dim J As Long
Dim lPlay As Long
Dim lX As Long
Dim lY As Long
Dim sSplit() As String
lPlay = i

For J = 1 To lPlay
    sSplit = Split(sMouseArray(J - 1))
    lX = CLng(sSplit(0))
    lY = CLng(sSplit(1))
    Sleep hscrlSpeed.Value
    SetCursorPos lX, lY
Next J

End Sub

Private Sub cmdRecord_Click()
frmKey.Cls
frmKey.Create
```

```
cmdStop.Enabled = True
tmrMouse.Enabled = True
cmdRecord.Enabled = False
cmdNew.Enabled = False
Create

st = DateTime.Time
End Sub

Private Sub cmdStop_Click()
et = DateTime.Time
cmdStop.Enabled = False
tmrMouse.Enabled = False
cmdPlay.Enabled = True
cmdNew.Enabled = True
LastRec
Unload frmKey
'
End Sub

Private Function RecordMouse() As String
Dim mouse As POINTAPI
GetCursorPos mouse
RecordMouse = mouse.X & " " & mouse.Y
End Function

Private Sub Frame1_DragDrop(Source As Control, X As Single, Y As Single)

End Sub

Private Sub hscrlSpeed_Change()
lblSpeed.Caption = "Speed: " & hscrlSpeed.Value
End Sub

Private Sub hscrlSpeed_Scroll()
lblSpeed.Caption = "Speed: " & hscrlSpeed.Value
End Sub

Private Sub tmrMouse_Timer()
ReDim Preserve sMouseArray(i)
sMouseArray(i) = RecordMouse
Set fs = New FileSystemObject
Set ts = fs.OpenTextFile("C:\Mousefile.txt", ForAppending, True)
If i = 0 Then
ts.WriteLine DateTime.Time & "  :  " & sMouseArray(i)
Else
If sMouseArray(i) <> sMouseArray(i - 1) Then

ts.WriteLine DateTime.Time & "  :  " & sMouseArray(i)
'ts.WriteLine "IkkaVB Forums"
'ts.WriteLine "IkkaVB Forums"

End If
```

```
End If
ts.Close
i = i + 1

End Sub

Private Sub Create()
Set fs = New FileSystemObject


'To write
Set ts = fs.OpenTextFile("C:\Mousefile.txt", ForWriting, True)

'ts.WriteLine "I Love 555"
'ts.WriteLine "VB Forums6666678888"
ts.Close
End Sub

Private Sub LastRec()
Set fs = New FileSystemObject
Set ts = fs.OpenTextFile("C:\Mousefile.txt", ForAppending, True)
ts.WriteLine "Time Taken in H:M:S : " & DateTime.Second(et - st)
ts.Close
End Sub
```

Src2: frmKey.frm

```
`  VB form for key stroke capturing
`  User Action Recodering (UAR) tool V1.0

`  Copyright (C) 2008 Biomedical Informatics (BMI), St George's University of London. Citations to
`  the toolkit should be as `de Lusignan S, Kumarapeli P, Chan T, Pflug B, van Vlymen J, Jones B,
`  Freeman GK. The ALFA (activity Log Files Aggregation) toolkit: a method for precise
` observation of the consultation. <J Med Internet Res 2008; ..>.

`  This program is free software: you can redistribute it and/or modify it under the terms of the
`  GNU General Public License as published by the Free Software Foundation, either version 3 of
`  the License, or (at your option) any later version.

`  This program is distributed in the hope that it will be useful, but WITHOUT ANY WARRANTY;
`  without even the implied warranty of MERCHANTABILITY or FITNESS FOR A PARTICULAR
`  PURPOSE.  See the GNU General Public License for more details.

`  You should have received a copy of the GNU General Public License along with this program.  If
`  not, see <http://www.gnu.org/licenses/>.

Dim result As Integer
Dim fs As FileSystemObject
Dim ts As TextStream
```

```
Dim Tx As String

Private Declare Function GetAsyncKeyState Lib "user32" (ByVal vKey As Long) As Integer

Public Sub Form_Initialize()
Create
End Sub


Private Sub Form_KeyDown(KeyCode As Integer, Shift As Integer)

End Sub

Private Sub Form_Load()

End Sub

Private Sub Timer1_Timer()
'
For i = 1 To 128 '1 To 255
result = 0
result = GetAsyncKeyState(i)
Tx = ""


If result = -32767 Then
If i = 32 Then 'Chr$(i) = " " Then
'Text1.Text = Text1.Text +
Tx = "(Space)" 'i:" & i
Else
If i = 13 Then
'Text1.Text = Text1.Text +
Tx = "(Enter)"
Else
If i = 1 Then
'Text1.Text = Text1.Text +
Tx = "(Mouse Click)"
Else
If i = 37 Then
'Text1.Text = Text1.Text +
Tx = "(LeftA)"
Else
If i = 38 Then
'Text1.Text = Text1.Text +
Tx = "(UpA)"
Else
If i = 39 Then
'Text1.Text = Text1.Text +
Tx = "(RightA)"
Else
If i = 40 Then
'Text1.Text = Text1.Text +
Tx = "(DownA)"
```

```
Else
If i = 8 Then
'Text1.Text = Text1.Text +
Tx = "(BackSpace)"
Else
If i = 16 Then
'Text1.Text = Text1.Text +
Tx = "(Shift)"
Else
If i = 46 Then
'Text1.Text = Text1.Text +
Tx = "(Delete)"
Else
If i = 20 Then
'Text1.Text = Text1.Text +
Tx = "(Caps)"
Else
If i = 9 Then
'Text1.Text = Text1.Text +
Tx = "(Tab)"
Else
'Text1.Text = Text1.Text + Chr$(i) '+ "(Enter)i:" & i
Tx = Chr$(i)

End If
End If
End If
End If
End If
End If
End If
End If
End If
End If
End If
End If
End If


Set fs = New FileSystemObject
'To write
Set ts = fs.OpenTextFile("C:\Keyfile.txt", ForAppending, True)
If Tx <> "" Then
ts.WriteLine DateTime.Time & "  :  " & Tx

End If
ts.Close

Next i

End Sub
Public Sub Create()
Set fs = New FileSystemObject
```

```
Set ts = fs.OpenTextFile("C:\Keyfile.txt", ForWriting, True)
ts.Close
End Sub
```

Biomedical Informatics, Division of Community Health Sciences
Tel + 44 (0)20 8725 5661 Fax + 44 (0)20 8725 3584 Email slusigna@sgul.ac.uk
